# Establishing reference intervals for 25 common biochemical analytes in Tibetans living at very high altitude

**DOI:** 10.1515/med-2025-1285

**Published:** 2026-01-19

**Authors:** Bai Ci, Yangzong Suona, Zhuoga Danzeng, Zhijuan Liu, Ju Huang, Rui Zhang, Shensong Li, Zhuoma Ciren, Chunyan Yuan, Luobu Gesang

**Affiliations:** High Altitude Medical Research Institute, Tibet Autonomous Region People’s Hospital, Key Laboratory of Translational Medicine for Human Adaptation to the High-altitu, Lhasa, China; High Altitude Medical Research Institute, Tibet Autonomous Region People’s Hospital, Lhasa, China; Department of Clinical Laboratory Medicine, Tibet Autonomous Region People’s Hospital, Lhasa, China; Department of Mountain Sickness and Cardiology, Tibet Autonomous Region People’s Hospital, Lhasa, China; Department of Pediatrics, Tibet Autonomous Region People’s Hospital, Lhasa, China

**Keywords:** biochemicals, high altitude medicine, reference intervals, very high altitude

## Abstract

**Objectives:**

High altitude poses extreme living environment for humans, impacting human physiology and leading to physiological adaptations, including higher hemoglobin levels in highlanders. However, further understanding is required regarding the medical reference ranges at very high altitudes (>4,500 m). Therefore, we conducted a study involving 1,656 healthy individuals from the “Health Improvement at Very High Altitude (HI-VHA)” population to establish a reference range for 25 biochemical analyses in this population residing at very high altitudes.

**Methods:**

The HI-VHA project sampled 3,564 individuals from Tibet Autonomous Region above 4,500 m. After strict exclusion criteria, 1,656 healthy individuals were included to establish age and sex stratified reference intervals (RIs) for 25 biochemical analytes using serum samples.

**Results:**

RIs were generated following the statistical guidelines outlined in CLSI C28-A3. Among the 25 biochemical analytes studied, the levels of ALT, GLB, CREA, UA, HDLC, and HCY showed significant variations by gender, while ALB, AG, CHOL, and DBIL were influenced by age. LDLC was the only analyte affected by both gender and age. AST, TP, TBIL, IBIL, Glu, TG, LDLC, CRP, K, Na, Cl, Ca, Mg, and P, comprising 14 analytes, were not influenced by gender or age.

**Conclusions:**

We established the RIs for 25 biochemical analytes in the very high-altitude population. These RIs are crucial for disease diagnosis and health management of individuals living at very high altitudes. Moreover, an accurate identification of diseases commonly observed at very high altitudes provides insights into the biological adaptation mechanisms of humans residing in such environments.

## Introduction

The physiological well-being of the human body is influenced by the high-altitude environment [1]. Altitudes of 4,500 m or above are classified as very high-altitude which is an extreme living environment for humans [2], 3]. The Tibetan population has a long history of residing in high-altitude areas, dating back 30,000 years [4]. They are considered one of the ethnic groups best adapted to high altitudes [5]. However, living in such extreme high-altitude conditions still presents challenges. Generations of natural selection have resulted in the accumulation of adaptive genetic variations to high altitude [6], 7]. Additionally, physiological adaptations have been acquired, including lower hemoglobin levels and a blunted hypoxic ventilatory response [8], [9], [10]. These factors significantly influence These factors may significantly influence physiological concentrations of many blood constituents in humans. However, there is limited knowledge about the medical reference range for populations residing at very high altitudes. Therefore, the development of specific reference intervals for this population is necessary.

We concluded that the reference range of many analytes indicates significant fluctuation compared to the population living in the sea-levels, most of the analytes are partitioned by gender and age. The result would further reveal the physiological characteristics of human survival at high altitudes and has significant value in high-altitude medicine and adaptation [11], 12].

## Materials and methods

### Selection of reference population

**Figure 1:**
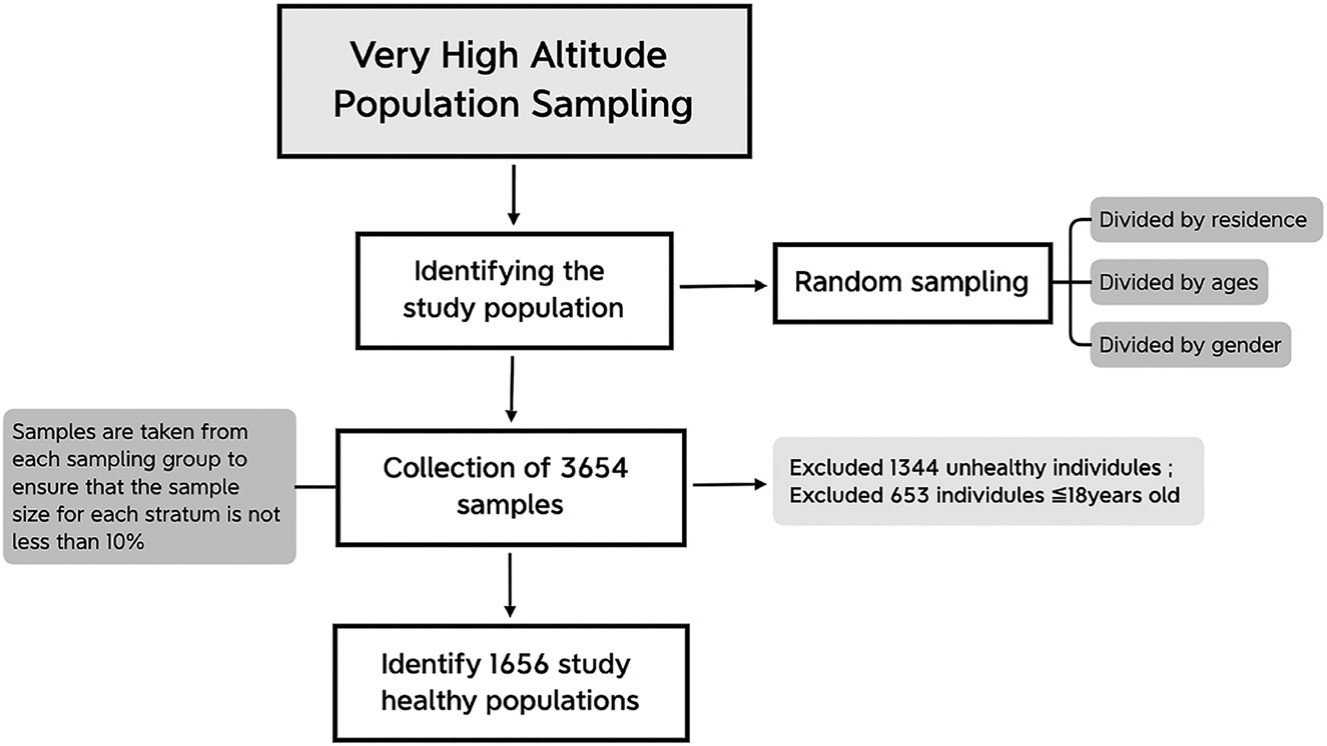
Selection of the reference population.

On June 24, 2021, we initiated the Health Improvement of the Very high-altitude [HI-VHA] project (ChiCTR2100047945). Through stratified sampling, we recruited 3,564 individuals from the Tibet Autonomous Region (TAR), specifically from three counties: Shuanghu County [at an altitude of 4,700 m], Nima County [at an altitude of 4,530 m], and Amdo County [at an altitude of 4,570 m]. These counties encompass 8 townships and 51 villages situated at elevations exceeding 4,500 m above sea level. To ensure representative sampling, the population was divided into 36 strata based on place of residence [three counties], age groups [7–20, 20–30, 30–40, 40–50, 50–60, and ≥60 years old], and gender [male and female]. Random sampling was conducted within each stratum to select the study population. After implementing strict inclusion criteria, a total of 1,656 healthy individuals from the HI-VHA population were included in this study (Figure 1).

### Selection of healthy group

As part of the HI-VHA project, a cohort of healthy individuals was established. All participants were in good physical condition and were aged 18 years or older, representing a balanced gender distribution and encompassing various Tibetan ethnicities. Prior to the project, it was ensured that participants had no history of long-term medication use. Exclusion criteria were applied to individuals with a history of diabetes, cardiovascular disease, excessive erythrocytosis, anemia, chronic renal disease, chronic liver disease (defined as a history of chronic liver disease or abnormal liver function indicated by elevated levels of TBIL, alanine aminotransferase [ALT], and aspartate aminotransferase [AST]) or obesity. Diabetes mellitus was defined as fasting plasma glucose levels ≥7 mmol/L, while obesity was determined by a body mass index [BMI] ≥30 kg/m^2^.

Cardiovascular disease was characterized by the presence of one or more of the following conditions: a history of myocardial infarction or heart failure, echocardiography indicating congenital heart disease (such as atrial septal defect, ventricular septal defect, patent ductus arteriosus, or valvular heart disease). Excessive erythrocytosis was identified as hemoglobin levels ≥210 g/L in males and ≥190 g/L in females, while anemia was defined as hemoglobin levels <130 g/L in males and <120 g/L in females. Chronic renal disease was determined by abnormal blood levels of creatinine and eGFR<60, and/or urine protein 2+ and above.

Chronic liver disease or renal disease. i.e. liver function TBIL>85.5 and ALT≥150 (3 times normal) and or AST≥120 [3 times normal], renal function eGFR<60 and or urine protein 2+ and above.

### Laboratory analysis

#### Sample collection and traceability

Blood sampling and storage: Blood samples were collected from local residents residing in three counties, eight townships, and fifty-one villages in Nyima, Shuanghu, and Amdo counties within the Nagqu region of TAR. The sample collection spanned over one month, from 24th June 2021 to 25th July 2021. Under basal conditions, blood was drawn between 7:00 am and 10:00 am after an overnight fast, following the recommended protocol. Participants refrained from engaging in strenuous muscle exercise for three consecutive days prior to sampling and avoided night work. To prevent postural changes in test results, participants remained seated for 5–10 min before venipuncture. A total of 20 mL of blood was drawn, distributed into three evacuated serum separator tubes (BD-Vacutainer): two non-anticoagulant tubes for biochemical and immunological tests, and one vacutainer tube containing K2-EDTA for routine blood tests. Anthropometric measurements, including weight, height, BMI, waist circumference, and blood pressure, were recorded on the day of sampling.

After collection, the blood samples were centrifuged at 3,500 rpm at room temperature. The resulting serum samples were then divided into five 2 mL sealed frozen tubes (Thermo Fisher) and immediately stored at −80 °C until further group measurements. The frozen samples were transported to the referral laboratory at Centre Lab, maintaining their frozen state. Sample analysis was conducted between 28th June 2021 and 20th July 2021. The purpose of preparing multiple aliquots of serum was to facilitate the testing of additional analytes using immunoassay techniques and to store them for future examination of new markers.

#### Laboratory analysis and quality control

Our central laboratory operates under ISO 15189, an international standard that outlines the requirements for quality and competence in medical laboratories. A C16,000 automatic biochemical analyzer (Abbott, USA) and its reagents and calibrators were used for tCO2 measurements using the phosphoenolpyruvate carboxylase method. The parameter settings, calibration, and detection procedures were conducted in strict accordance with the standard operating procedures provided in the instructions book. Liquid assayed multiqual quality control products (Item No.: 45,862 and 45,863, Bio-Rad, Hercules, CA, USA) were used for quality control. The total imprecision (coefficient of variation) of the tCO2 measurement assay was 4.353 % (for No. 45,862 at a mean value of 16.292 mmol/L) and 4.379 % [for No. 45,863 at a mean value of 21.274 mmol/L) The tCO2 results were qualified according to an external quality assessment by the Clinical Laboratory Center of the National Health Commission (China).

#### Statistics

In accordance with the Ichihara method (IFCC Document), sources of variation were assessed using 2-level ANOVA and MRA. Briefly, 2N-ANOVA, which accounts for multiple factors, was employed to determine whether partitioning reference values based on age or gender was necessary. The magnitude of components of the standard deviation (SD) was calculated using 2N-ANOVA and expressed as the SD ratio (SDR) for between-age (SDRage) and between-sex (SDRsex), representing the ratio of the SD between subgroups to the SD between individuals. For instance, SDRage=SD_Age/SD_individual. Following the IFCC/C-RIDL protocol, an SDR greater than 0.3 was considered as an indication to consider partitioning the reference values by sex or age. Multiple regression analysis (MRA) was employed to identify significant factors contributing to variations in reference interval (RI) results, while accounting for potential confounding relationships among factors such as BMI, blood type, ethyl alcohol consumption, smoking, and sedentariness. A standardized partial regression coefficient greater than 0.20 for a variable indicated its practical importance.

In the secondary exclusion procedure, latent abnormal values exclusion (LAVE), a multivariate-based method for identifying extreme values, was employed to remove participants with potential subclinical disease/latent abnormal values (e.g., liver dysfunction or inflammation). In summary, RIs were initially established using both parametric methods (mean±1.96 SD or after Gaussian transformation, if applicable) and nonparametric methods (2.5th – 97.5th percentile range). Individuals with two or more results outside the RIs derived from the previous computation were excluded during the LAVE procedure. RIs were then recalculated for the remaining participants. This process was repeated six times, until the RIs reached a state of stability.

For comparison purposes, RIs were determined using both parametric and nonparametric methods before and after applying the LAVE procedure, as described by Ichihara et al. [13], 14]. Ultimately, in determining the final value of the reference interval, values are rounded to make them more practical and easier for clinicians to understand and apply. For example, if the upper reference limit is calculated to be 10.23, it may be rounded to 10 or 10.2 to facilitate clinical use. Moreover, merging two or more reference interval, which means that in some cases, multiple similar reference intervals are merged if they can be reasonably combined. Such combinations may be based on clinical significance or statistical properties of the data. For example, if two age reference intervals are very similar, they may be combined into a single interval to simplify clinical application.

### Ethics and participant informed consent

The medical research study described in this paper was performed according to the Declaration of Helsinki https://www.wma.net/what-we-do/medical-ethics/declaration-of-helsinki/] and approved by the Medical Ethics Committee of Tibet Autonomous Region People’s Hospital, with a reference number ID ME-TBHP-21-028. Prior to enrollment, all participants provided informed consent by signing the informed consent form.

#### Questionnaire

The questionnaire includes essential items such as BMI, special diet, records of regularly taken medicines and/or supplements, menstrual status, smoking habits, weekly alcohol consumption (approximate grams of ethanol), and sleeping hours per day. This information will be used to analyze sources of variation in test results and determine the need for a secondary exclusion.

## Results

### Demographics of the population

The study cohort consisted of 1,656 healthy individuals from three different regions of TAR. Among them, 769 were males with a median age of 39 years, and a median BMI of 22.7 kg/m^2^, and 887 females were at same median age of males, and with a median BMI of 22.5 kg/m^2^. The proportion of participants who smoked and consumed alcohol was 7.3 % (n=1,656) and 4.1 % (n=1,656) of the study cohort, respectively. There was a statistically significant difference in smoking and drinking habits between men and women. However, no significant differences were observed in terms of age, BMI, and the distribution of participants by region between the sexes (Table 1).

**Table 1:** Clinical characteristic.

Characteristic	Total=1,656^a^	Male=769^a^	Female=887^a^	p-Value^b^
**Age**	39 (31, 49)	39 (32, 48)	39 (31, 50)	0.7
**BMI**	22.6 (20.1, 25.7)	22.7 (20.3, 25.8)	22.5 (20.0, 25.5)	0.1
**Region**				0.5
Nyma	590 (36 %)	280 (36 %)	310 (35 %)	
ShuangHu	452 (27 %)	215 (28 %)	237 (27 %)	
Anduo	613 (37 %)	274 (36 %)	339 (38 %)	
**Wrist**	76 (68, 86)	80 (70, 89)	74 (66, 83)	<0.001
**Smoking**				<0.001
No	1,536 (93 %)	671 (87 %)	865 (98 %)	
20/day	81 (4.9 %)	64 (8.3 %)	17 (1.9 %)	
<10/day	33 (2.0 %)	28 (3.6 %)	5 (0.6 %)	
<20/day	6 (0.4 %)	6 (0.8 %)	0 (0 %)	
**Drinking**				<0.001
No	1,584 (96 %)	706 (92 %)	878 (99 %)	
Yes	68 (4.1 %)	61 (8.0 %)	7 (0.8 %)	

^a^n (%); Median (IQR). ^b^Pearson’s Chi-squared test; Wilcoxon rank sum test; Fisher’s exact test.

### Source of variation and correlation among analytes

Multivariate regression analysis (MRA) was conducted independently for each gender, using a fixed set of explanatory variables (source of variations) including region, age, BMI, smoking (in four levels), and drinking, to define the reference intervals (RIs) for each analyte as the objective variable (Supplementary Table S1). Standardized partial regression coefficients (rp). was above 0.30, considered to be a significant effect size. In males, we observed a moderate decrease with age for ALB similar to trends reported in sea-level populations, sea-level adult ALB upper limits ∼48–55 g/L per CLSI/IFCC; Indian low-altitude reference 35–48 g/L [19], suggesting that this decrease may be a general physiological aging effect rather than altitude-specific (rp=−0.36) and AG (−0.32). Among females, only UA (rp=0.32) showed an increase with BMI, while CHOL (0.35) and LDLC (0.34) exhibited a substantial increase with age. Other analytes were not indicating significant association with these sources of variation.

### SDR as a guide for partitioning RIs

**Figure 2:**
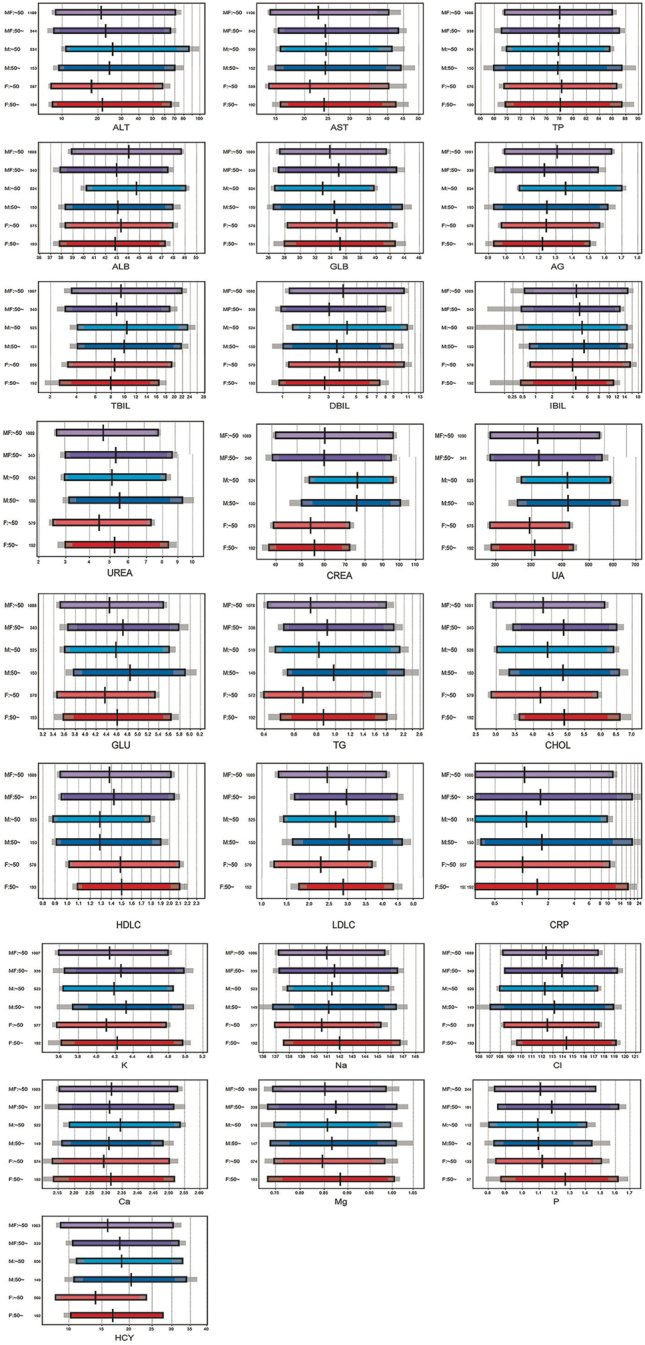
Reference intervals [RI] for analytes stratified by sex and age groups.

Table 2 presents the magnitude of between-sex differences, expressed as SDRsex, derived through two-level nested ANOVA with age as a covariate. SDR≥0.3 indicating significant differences. Regarding sex-partioning, SDRsex≥0.3 was observed for CREA, UA, HCY, HDLC, AG, ALT, Alb, and GLB, in descending order of magnitude. Regarding age-partioning, AG, CHOL, and LDLC were identified as partitioning indicators. Furthermore, SDRage specific to each gender was derived as SDRageM or SDRageF through one-way ANOVA. For males, significant SDRageM values were observed for ALB, AG, CHOL, and LDLC, while for females, significant SDRageF values were observed only for DBIL, UREA, CHOL, and LDLC (Figure 2).

**Table 2:** Standard deviation ratio (SDR) by sex and age of analytes.

ItemName	SDR-sex	SDR-age	SDR-age M	SDR-age F
ALT	0.366	0.273	0.294	0.254
AST	0.15	0.14	0.112	0.165
TP	0.051	0.058	0	0.08
ALB	0.336	0.266	0.389	0.128
GLB	0.302	0.206	0.241	0.174
AG	0.436	0.3	0.376	0.204
TBIL	0.296	0.129	0.134	0.121
DBIL	0.107	0.271	0.229	0.302
IBIL	0.133	0	0.096	0
UREA	0.298	0.25	0.175	0.313
CREA	1.429	0.105	0.119	0.073
UA	1.125	0.13	0.132	0.126
GLU	0.281	0.282	0.275	0.288
TG	0.21	0.24	0.188	0.28
CHOL	0	0.45	0.381	0.513
HDLC	0.551	0	0.045	0
LDLC	0.298	0.44	0.374	0.509
CRP	0	0.113	0.163	0.046
K	0.165	0.146	0.107	0.176
Na	0.098	0.174	0	0.239
Cl	0.093	0.262	0.228	0.288
Ca	0.238	0.177	0.237	0.105
Mg	0	0.125	0	0.19
P	0.298	0.207	0	0.275
HCY	0.597	0.258	0.23	0.292

SDR≥0.3 was considered as an indicator to consider partitioning of Ris. ALB, albumin; ALT, alanine aminotransferase; AST, aspartate aminotransferase; Ca, calcium; Cl, chloride; CREA, creatinine; Glu, Glucose; HDL-C, high-density lipoprotein-cholesterol; IP, inorganic phosphate; K, potassium; LAVE, latent abnormal values exclusion; LDL-C, low-density lipoprotein-cholesterol; Mg, magnesium; Na, sodium; SDR, standard deviations ratio; TBIL, total bilirubin; DBIL, direct bilirubin; TC, total cesterol; TG, triglycerides; TP, total protein; UA, uric acid.

### Derivation of reference intervals

RIs were calculated using four approaches: parametric (P) and nonparametric (NP) methods, with and without the application of the latent abnormal values exclusion (LAVE) method. For potential exclusion of abnormal values, ALT, AST, and TBIL were selected as indicator parameters to identify individuals who may have subclinical diseases such as chronic liver disease or inflammation. LAVE (+) was chosen as the indicator parameter. Histograms and probability plots before and after Box-Cox transformation were examined to assess whether the data conformed to a Gaussian distribution with thresholds used to quantitatively determine normality after transformation: skewness (<−1 or >1) and kurtosis (<−2 or >2). Consistent with a Gaussian distribution, RIs were calculated using a parametric approach, with mean±1.96 standard deviation defining the upper and lower limits of the transformed RIs. Not conforming to a Gaussian distribution, RIs were calculated using a nonparametric method with 2.5 % and 97.5 % of their distributions as the final upper and lower RIs limits. Gaussian distribution was observed for all analytes except for AST, AG, IBIL, GLU, K, Ca, and Mg, leading to the selection of the parametric method (P) (Supplementary File S1). Supplementary Table S2 displays comparisons of RIs for all analytes. In Supplementary File S2 we presented the effect of LAVE procedure and Table 3 illustrated a list of RIs adopted for all analytes is presented. In deriving RIs by sex and age or not, we primarily considered SDRsex and SDRage, but also the bias of the RI limits (LL and UL), as detailed in Table 2, 3, 4 listed the finalised RIs used for all analytes (Table 5).

**Table 3:** The list of RIs stratified by sex and age group.

Item	LAVE	Paramatric	Sex	Age	n	Lower L limit	Upper limit
ALT	LAVE (+)	p	M		703	10.8	86.9
			F		821	8.1	62.7
AST	LAVE (+)	np	MF		1,532	14	53.2
TP	LAVE (−)	p	MF		1,645	69.37	86.43
ALB	LAVE (−)	p	M	19–49	593	39.73	48.94
		p	M	≥50	171	37.25	47.51
		p	F		884	37.72	47.72
GLB	LAVE (−)	p	M		766	26.55	41.16
		p	F		882	28.47	42.57
AG	LAVE (−)	np	M	19–49	596	1	1.7
		np	M	≥50	173	0.9	1.6
		np	F		887	0.9	1.5
TBIL	LAVE (+)	p	MF		1,463	3.52	20.95
DBIL	LAVE (+)	p	M		696	1.18	10.29
		p	F	19–49	586	1.25	10.8
		p	F	≥50	200	0.89	7.3
IBIL	LAVE (+)	np	MF		1,489	0.96	14.1
UREA	LAVE (−)	p	M		767	2.873	8.469
		p	F	19–49	659	2.425	7.272
		p	F	≥50	224	2.964	8.175
CREA	LAVE (−)	p	M		764	51.7	96.5
		p	F		882	38.7	72.1
UA	LAVE (−)	p	M		768	265.6	593.5
		p	F		882	190.9	438.4
GLU	LAVE (−)	np	MF		1,656	3.5	5.6
TG	LAVE (−)	p	MF		1,650	0.416	1.901
CHOL	LAVE (−)	p	M	19–49	596	2.984	6.427
		p	M	≥50	173	3.213	6.581
		p	F	19–49	660	2.812	5.931
		p	F	≥50	224	3.61	6.44
HDLC	LAVE (−)	p	M		769	0.876	1.808
		p	F		883	1.019	2.129
LDLC	LAVE (−)	p	MF	19–49	1,257	1.305	4.234
		p	MF	≥50	397	1.649	4.415
CRP	LAVE (−)	p	MF		1,614	0.254	12.848
K	LAVE (−)	np	MF		1,656	3.6	4.9
Na	LAVE (−)	np	MF		1,656	137	146
Cl	LAVE (−)	np	MF		1,656	108	118
Ca	LAVE (−)	np	MF		1,656	2.11	2.51
Mg	LAVE (−)	np	MF		1,654	0.72	0.98
IP	LAVE (−)	p	MF		400	0.817	1.509
HCY	LAVE (−)	p	M		744	11.063	34.184
		p	F		866	9.311	27.296

ALB, albumin; ALT, alanine aminotransferase; AST, aspartate aminotransferase; Ca, calcium; Cl, chloride; CREA, creatinine; Glu, Glucose; HDL-C, high-density lipoprotein-cholesterol; IP, inorganic phosphate; K, potassium; LAVE, latent abnormal values exclusion; LDL-C, low-density lipoprotein-cholesterol; Mg, magnesium; Na, sodium; SDR, standard deviations ratio; TBIL, total bilirubin; DBIL, direct bilirubin; TC, total cesterol; TG, triglycerides; TP, total protein; UA, uric acid.

**Table 4:** RIs derived by a parametric method with or without LAVE and comparison with those from reagent inserts and other studies.

	HI-VHA					Health occupational standard of China			Saudi Arabia’s study			Indian-study				African-study (18–70 y)		
Analyte	LAVE	Age	M+F	M	F	M+F	M	F	M+F	M	F	Age	M+F	M	F	M+F	M	F
						Healthy people (n=720) from six regions (northeast, north, northwest, south, southwest) of China.			Healthy individuals (n=826) aged≥18 years were recruited			Healthy individuals (n=500) aged 18–65 years were recruited				Healthy individuals (n=804) aged 18–65 years were recruited		
ALT	LAVE (+)			10.8–86.9	8.1–62.7		9–50	7–40		7.9–29.6	3.7–26.0			15–74	10–37		9–57	7–27
AST	LAVE (+)		14–53.2				15–40	13–35		11–28	10–24			20–53	17–39		20–43	18–32
TP	LAVE (−)		69.37–86.43			65–85			62–77									
ALB	LAVE (−)	19–49		39.73–48.94	37.72–47.72	40–55			39–50			18–65			36–47			
		≥50		37.25–47.51								<45		39–52				
												≥45		37–49				
GLB	LAVE (−)			26.55–41.16	28.47–42.57	20–40												
AG	LAVE (−)	19–49		1–1.7	0.9–1.5	1.2–2.4										8.5–21.1		
		≥50		0.9–1.6														
TBIL	LAVE (+)		3.52–20.95				≤26.0	≤21.0		3.6–22.4	2.2–15.5			6.2–23.7	4.0–17.3		7–36	5–24
DBIL	LAVE (+)	19–49		1.18–10.29	1.25–10.8	0–3.4											1.4–7.0	1.0–4.8
		≥50			0.89–7.3													
IBIL	LAVE (+)		0.96–14.1															
UREA	LAVE (−)	19–49		2.873–8.469	2.425–7.272		3.1–8.0	2.6–7.5						2.2–6.0				
												<45			1.9–5.1			
		≥50			2.964–8.175							≥45			2.4–6.7			
CREA	LAVE (−)			51.7–96.5	38.7–72.1		57–97	41–73		66–111	50–74			58–95	35–74			
UA	LAVE (−)			265.6–593.5	190.9–438.4		208–428	155–357		223–444	148–321			248–509	159–404		229–467	147–347
GLU	LAVE (−)		3.5–5.6		3.5–5.6													
TG	LAVE (−)		0.416–1.901		0.416–1.901	<1.7				0.50–3.58	0.39–1.60			0.6–2.7	0.5–2.1		0.42–2.08	0.40–1.57
CHOL	LAVE (−)	19–49		2.984–6.427	2.812–5.931	<5.2												
		≥50		3.213–6.581	3.61–6.44													
HDLC	LAVE (−)			0.876–1.808	1.019–2.129	≥1.04				0.74–1.76	0.98–2.19					0.92–2.18		
LDLC	LAVE (−)	19–49	1.305–4.234			<3.4			1.80–4.34				1.7–4.4				1.97–5.15	2.14–4.07
		≥50	1.649–4.415															
CRP	LAVE (−)		0.254–12.848			≤5											0.17–4.93	0.44–6.75
K	LAVE (−)		3.6–4.9			3.50–5.30			3.7–4.9				3.8–5.0			3.5–5.0		
Na	LAVE (−)		137–146			137–147			135–144				135–146			136–143		
Cl	LAVE (−)		108–118			99–110	1		101–111				102–113			99–108		
Ca	LAVE (−)		2.11–2.51			2.11–2.52			2.11–2.56				2.10–2.44			2.17–2.51		
Mg	LAVE (−)		0.72–0.98			0.75–1.02							0.77–1.07			0.72–0.97		
P	LAVE (−)		0.817–1.509			0.85–1.51												
HCY	LAVE (−)			11.063–34.184	9.311–27.296	≤15												

ALB, albumin; ALT, alanine aminotransferase; AST, aspartate aminotransferase; Ca, calcium; Cl, chloride; CREA, creatinine; Glu, Glucose; HDL-C, high-density lipoprotein-cholesterol; IP, inorganic phosphate; K, potassium; LAVE, latent abnormal values exclusion; LDL-C, low-density lipoprotein-cholesterol; Mg, magnesium; Na, sodium; SDR, standard deviations ratio; TBIL, total bilirubin; DBIL, direct bilirubin; TC, total cesterol; TG, triglycerides; TP, total protein; UA, uric acid.

**Table 5:** Comparison of reference intervals (RIs).

Analyte	High-altitude RI	Normal range (sea level)	Difference
Creatinine, mg/dL	51.7–96.5 (M), 38.7–72.1 (F)	57–97 (M), 41–73 (F)	Lower at high altitude
LDL-C, mg/dL	1.305–4.234 (19–49), 1.649–4.415 (≥50)	<3.4	Lower at high altitude
ALT, U/L	10.8–86.9 (M), 8.1–62.7 (F)	7–40	Higher at high altitude
AST, U/L	14–53.2	13–35	Higher at high altitude
Uric acid, mg/dL	265.6–593.5 (M), 190.9–438.4 (F)	208–428 (M), 155–357 (F)	Elevated at high altitude
Hemoglobin, g/dL	Varies significantly	13.5–17.5 (M), 12.0–15.5 (F)	Elevated at high altitude

## Discussion

This study has not only endeavored to find differences in the reference intervals between high altitude and plains areas for various analytes. Still, it has also identified differences between altitudinal gradients at high altitudes for some indicators. We expect this study to enhance the accuracy of clinical decision-making and health management for individuals living at high altitudes.

Previous studies in high-altitude populations have indicated lower creatinine levels and higher uric acid levels compared to populations residing at lower elevations, suggesting potential metabolic adjustments during high-altitude acclimatization [15]. In males, we observed a moderate age-related decrease in ALB, consistent with trends reported in sea-level populations. For example, sea-level adult ALB upper limits are typically ∼48–55 g/L according to CLSI/IFCC recommendations, while Indian low-altitude populations show reference ranges of 35–48 g/L [19]. These findings suggest that the decrease in ALB may reflect a general physiological aging effect rather than an altitude-specific phenomenon (rp=−0.36 for ALB and −0.32 for AG). In contrast, previous high-altitude studies have shown higher creatinine levels in high-altitude populations compared to sea-level populations [16]. However, this study was conducted at an altitude of 3,400 m and demonstrated that creatinine levels do not change after a certain altitude. However, it did not demonstrate whether creatinine levels continue to stabilize or decrease at higher altitudes (above 3,400 m) Therefore, more altitude gradient studies are needed to demonstrate further whether there is a decrease in creatinine levels at very high altitudes, even affecting other renal function indices. On the other hand, uric acid levels in the very high-altitude population were nearly 100 units higher in both genders compared to the upper and lower limits of the sea-levels reference range (Table 4). Our result further confirming the impact of the very high-altitude environment on various laboratory analytes related to renal function.

We found differences between different altitude gradients for the same ethnic groups from the results of lipid levels. Prior research into lipid profiles among populations living at high altitudes indicates that these individuals typically exhibit lower levels of total cholesterol and LDL-C, along with higher levels of HDL-C, likely due to environmental factors specific to highland regions [15]. The standardized partial regression coefficient (rp) was above 0.30, indicating a significant effect size. In males, ALB showed a moderate age-related decrease, consistent with trends reported in sea-level populations. At sea level, adult ALB upper limits are typically ∼48–55 g/L according to CLSI/IFCC guidelines, whereas Indian low-altitude populations show reference values of 35–48 g/L [19]. These findings suggest that the decline in ALB may reflect a general physiological aging effect rather than an altitude-specific pattern (rp=−0.36 for ALB; −0.32 for AG). Moreover, in the case of lipid levels, another study on high-altitude populations and lipid levels concluded that mean HDL-C levels increased and mean LDL-C levels decreased, while mean total cholesterol levels did not change significantly [17]. However, our results showed a different trend, which is the upper limits of total cholesterol and HDL-C were higher than the plain reference range for both sexes. LDL-C levels were also generally higher than the plain reference range and were influenced by age and gender (Table 4). The inconsistency in this finding, which we attribute to the difference in lipid levels between the two high-altitude areas, is because the subjects in both studies were of Tibetan descent. However, the subjects in the previously mentioned study were of because in the city of Lhasa, which is located at an altitude of 3,660 m above sea level, whereas our participants resided at an altitude of 4,500 m above sea level. The levels of these indicators are not only different from those at high altitudes vs. those in the plains but there is also a difference between high altitudes and higher altitudes. Therefore, developing special reference intervals is critical to be considered for higher altitudes like very high altitudes above 4,500, so that accurately treat populations at very high altitudes.

Liver function analytes may exhibit variations in individuals living at high altitudes [18]. Serum alanine aminotransferase (ALT) and serum aspartate aminotransferase (AST), which are indicators of liver function, may be elevated in individuals residing at high altitudes compared to those living in plains (Table 4). In our study, the reference value range for ALT in the very high-altitude population was approximately 20 units higher than the normal value range in both males and females in plains. The reference range for AST was also higher than the plains reference range, but it was not influenced by gender. Notably, a study conducted on 500 healthy individuals in Mumbai (Table 4), India, at an altitude of 400 m reported ALT levels consistent with our findings, although the upper limit for men in the Indian population was higher than in our very high-altitude population [19].

In our study, the reference interval for homocysteine (HCY) exhibited significant fluctuations when compared to the plains reference interval. Overall, HCY levels in the very high-altitude population were substantially higher than typical sea-level reference values. In sea-level populations, mean HCY concentrations are usually around 6.0 μmol/L, whereas the upper limits in our cohort were nearly twice as high.

Various factors, including genetic factors, age, gender, diet, lifestyle, etc., can influence HCY levels [20], [21], [22]. In line with our findings, a study investigating homocysteine levels and their association with altitude concluded that homocysteine levels were significantly elevated in high-altitude residents [23]. These studies collectively indicate higher homocysteine levels in individuals residing at high altitudes and their association with cardiovascular disease-related risk factors such as blood pressure and lipid levels [24].

## Supplementary Material

Supplementary Material

Supplementary Material

Supplementary Material

Supplementary Material

Supplementary Material
