# Supplementary material for: Establishing reference intervals for 25 common biochemical analytes in Tibetans living at very high altitude
Source: Open Med (Wars). 2026 Jan 19;21(1):20251285. doi: 10.1515/med-2025-1285 (PMC12917553; doi:10.1515/med-2025-1285)

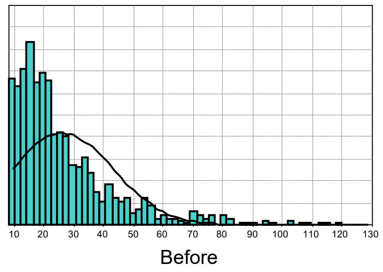

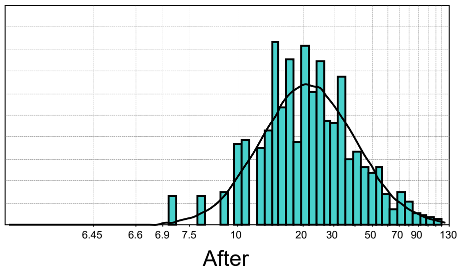

ALT MF n=1,469
Para: 8.61 ~ 21.68 ~ 71.77
Nonpara: 8.25 ~ 21.20 ~ 80.90
Pow=0.173 TPos=6.423
Kurt=-0.242 Skew=0.199
K-S test for normality: 0.04298

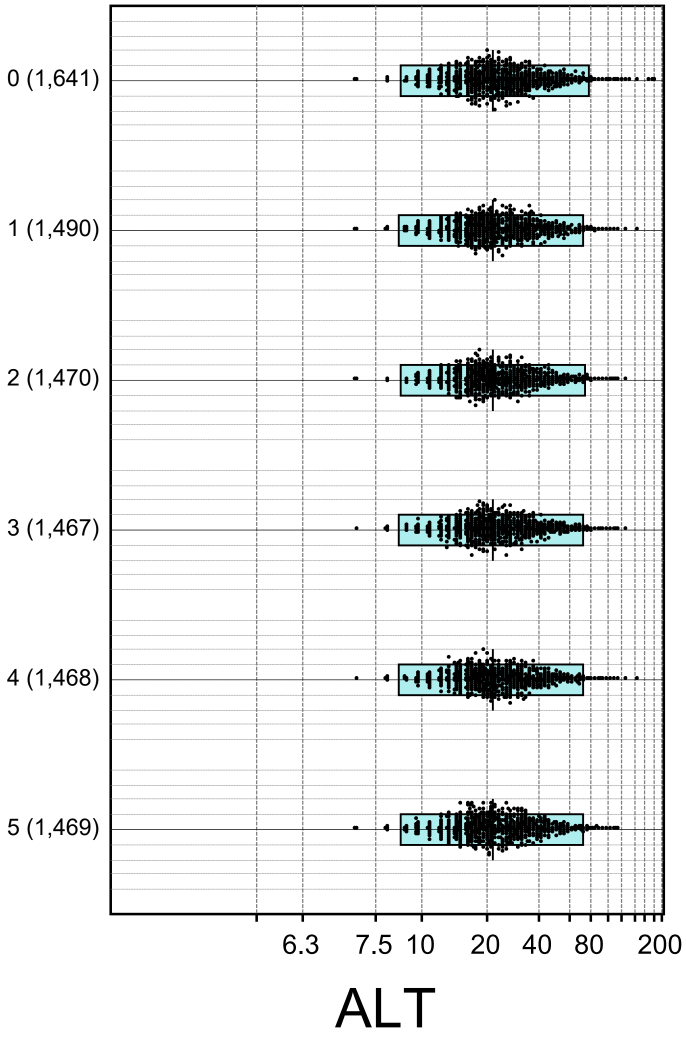

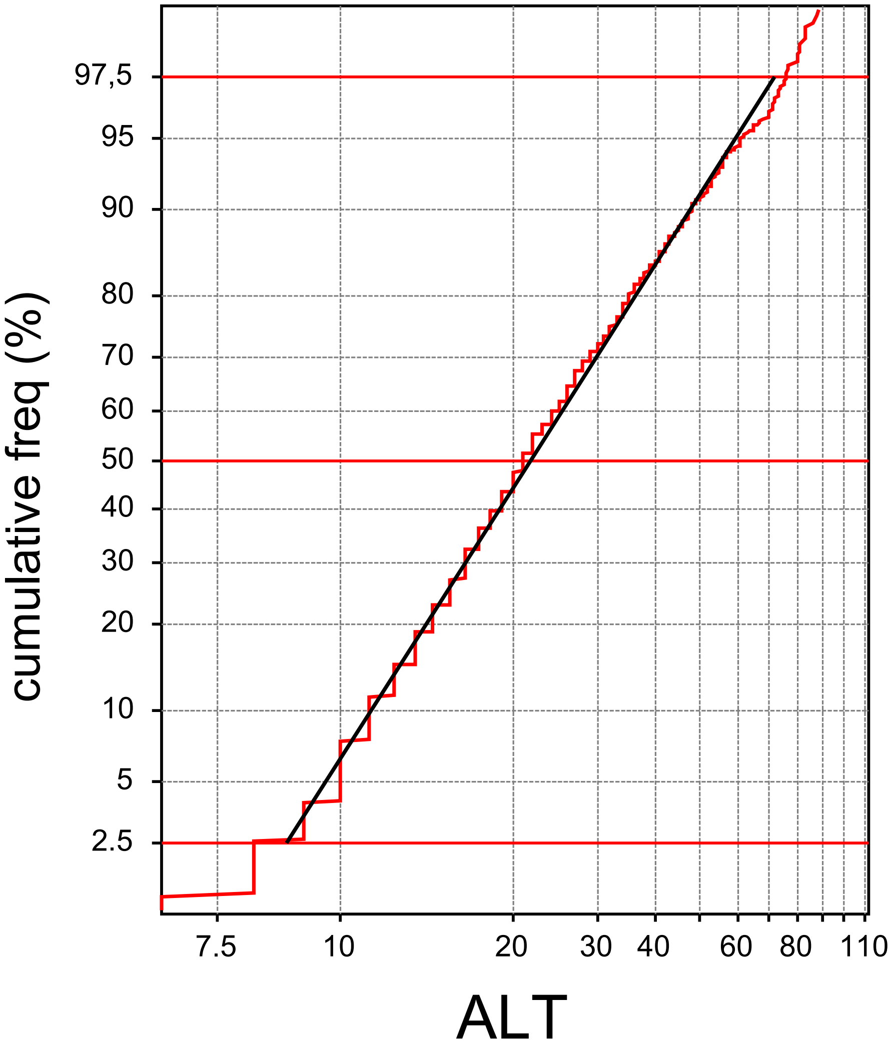

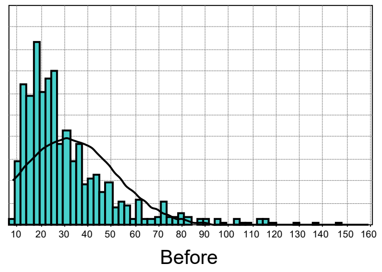

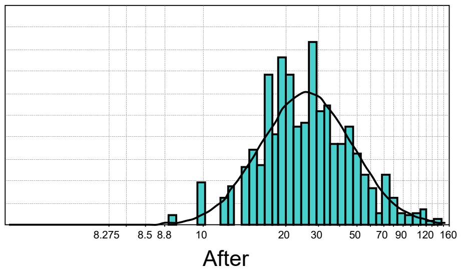

ALT M n=686
Para: 10.75 ~ 25.82 ~ 84.02
Nonpara: 10.15 ~ 25.80 ~ 99.43
Pow=0.171 TPos=8.209
Kurt=-0.192 Skew=0.073
K-S test for normality: .415 (NS)

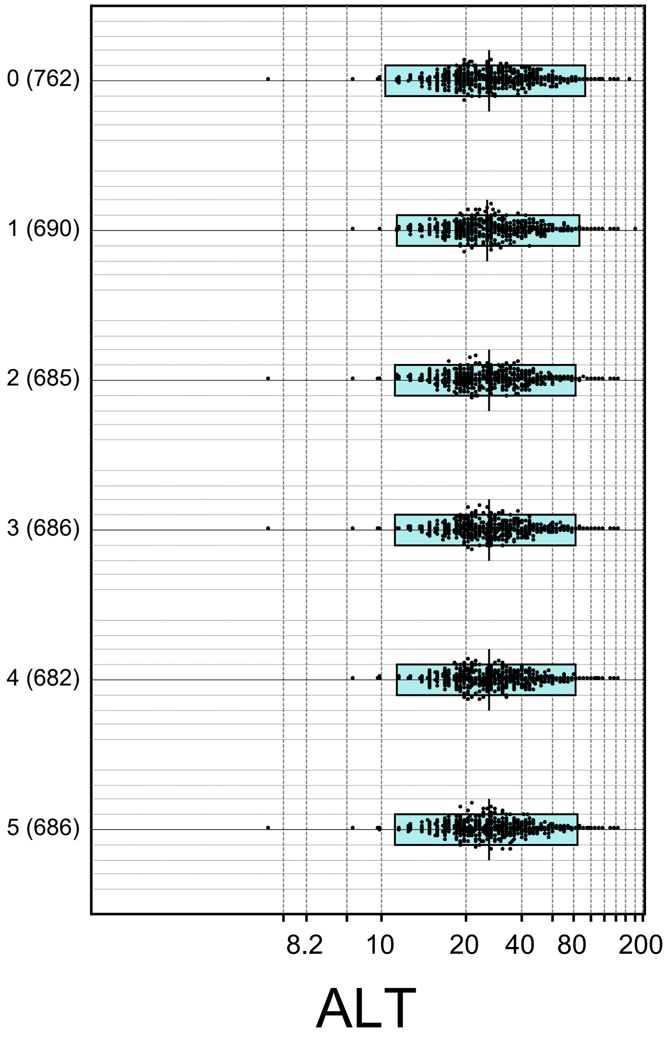

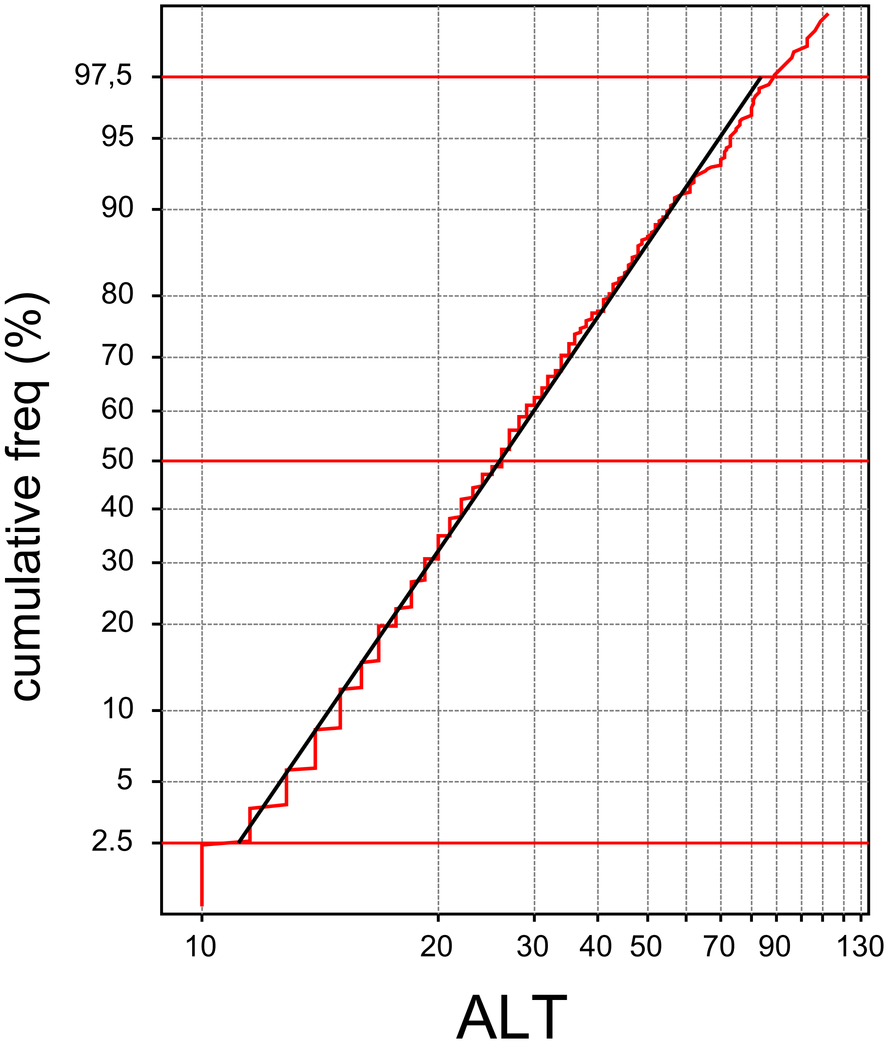

ALT F n=791
Para: 8.04 ~ 18.53 ~ 60.98
Nonpara: 7.60 ~ 18.40 ~ 69.97
Pow=0.143 TPos=6.17
Kurt=-0.047 Skew=0.063
K-S test for normality: .084 (NS)

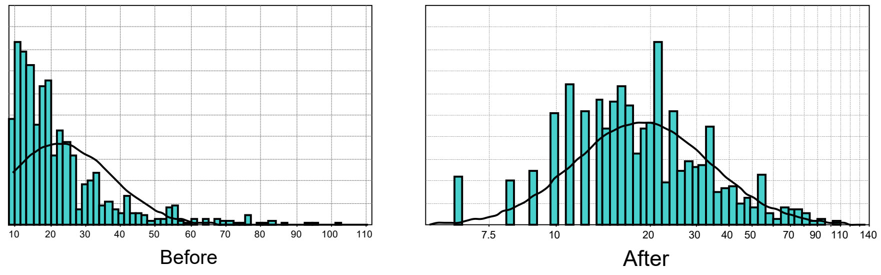

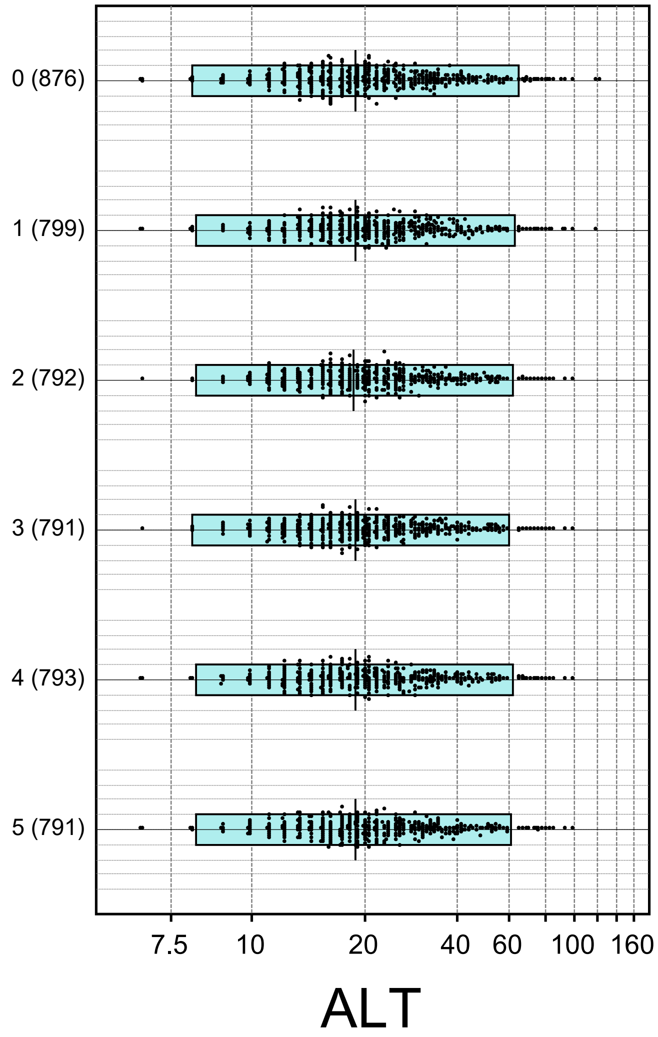

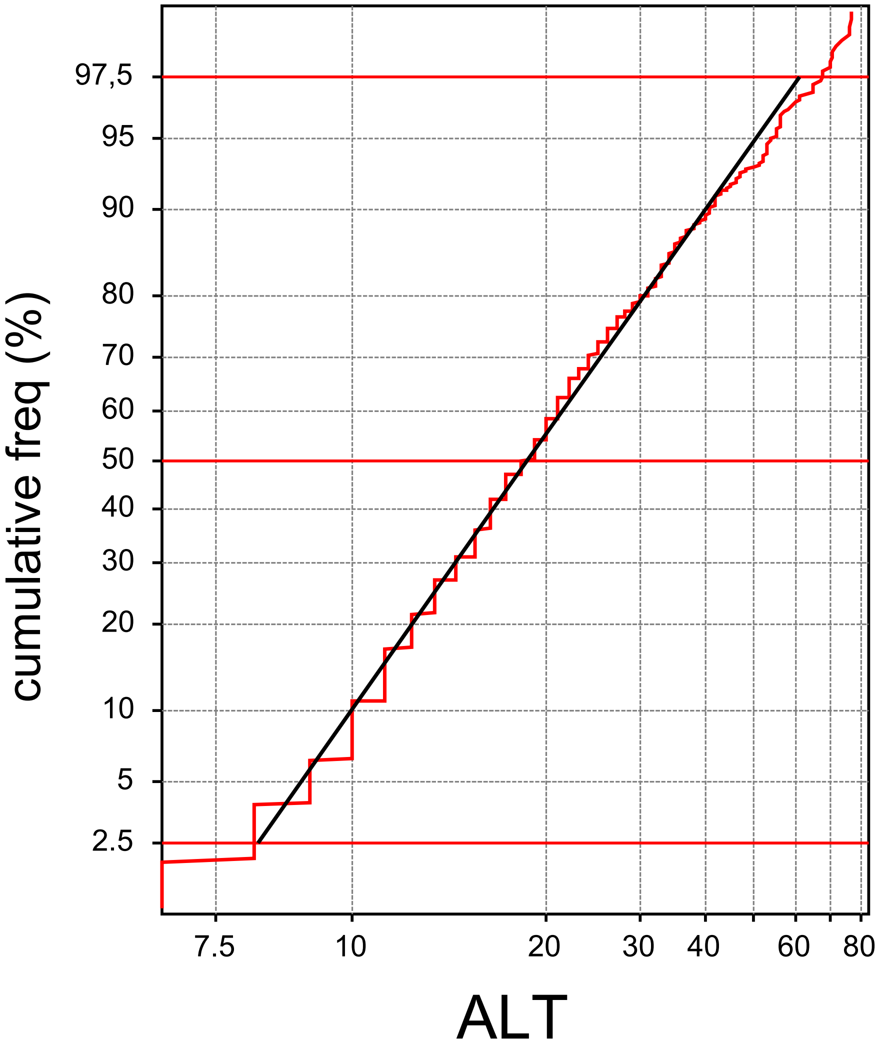

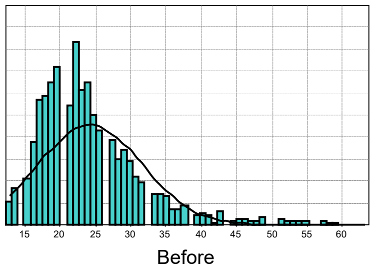

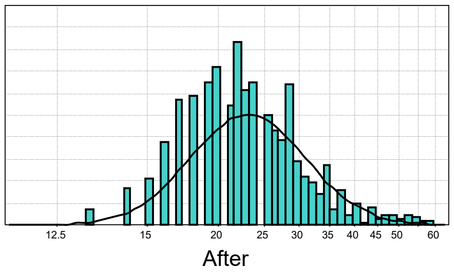

AST MF n=1,458
Para: 14.42 ~ 23.11 ~ 41.72
Nonpara: 14.19 ~ 22.90 ~ 53.40
Pow=0.384 TPos=12.304
Kurt=-0.063 Skew=0.117
K-S test for normality: 0.00021

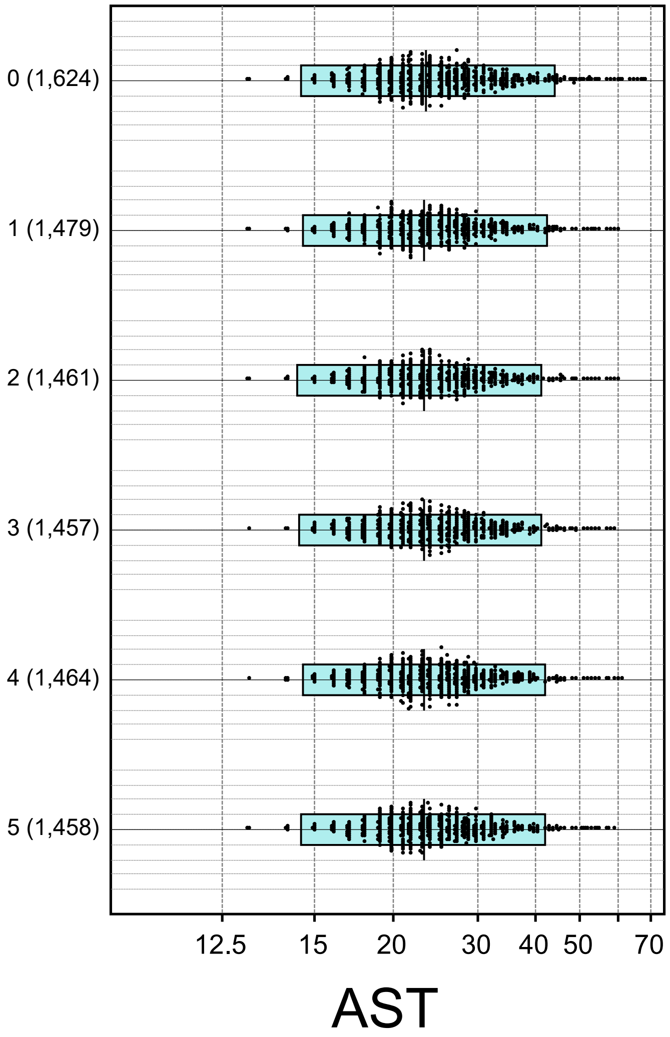

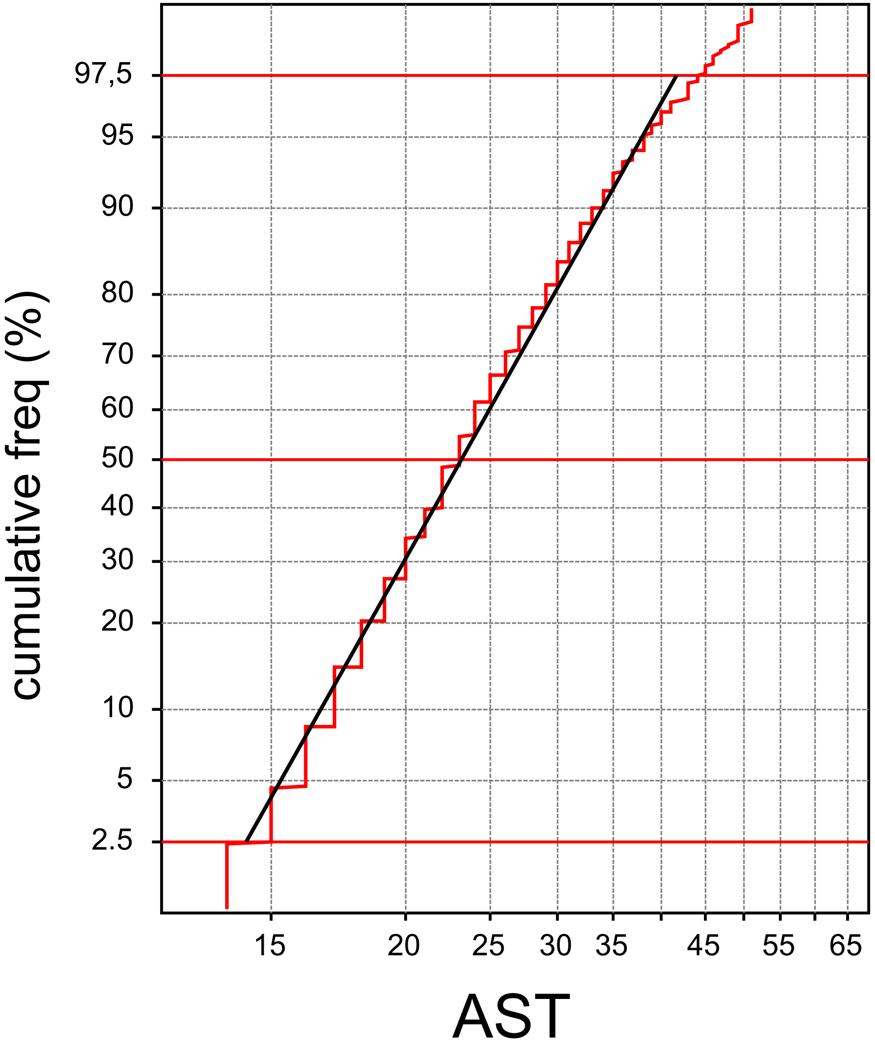

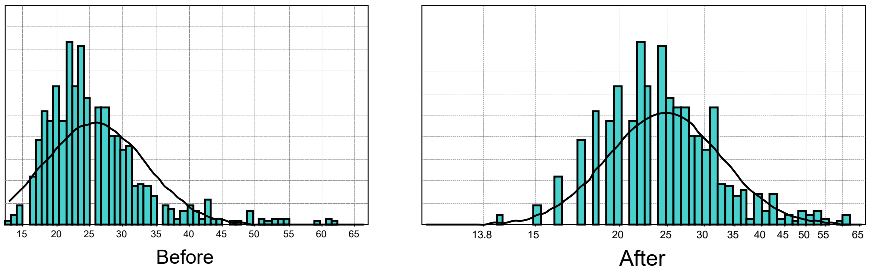

AST M n=682
Para: 15.73 ~ 24.50 ~ 43.10
Nonpara: 15.80 ~ 24.20 ~ 57.13
Pow=0.382 TPos=13.501
Kurt=-0.062 Skew=0.183
K-S test for normality: 0.0385

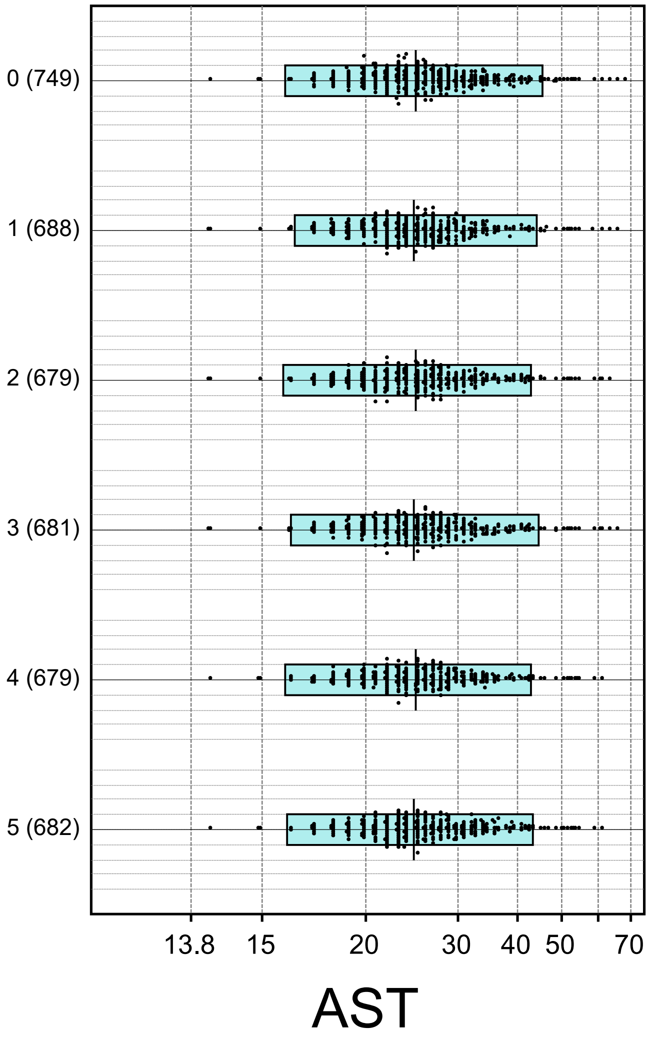

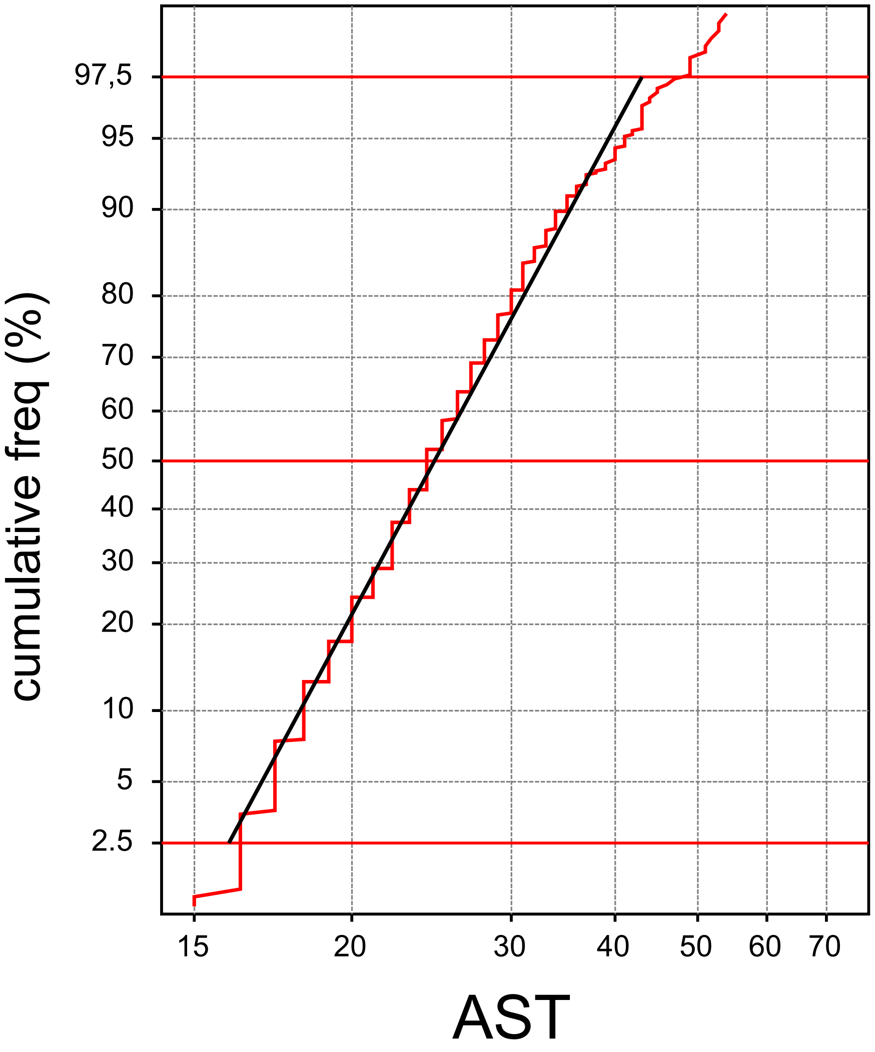

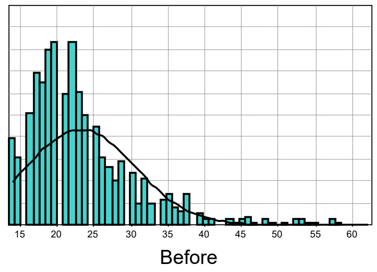

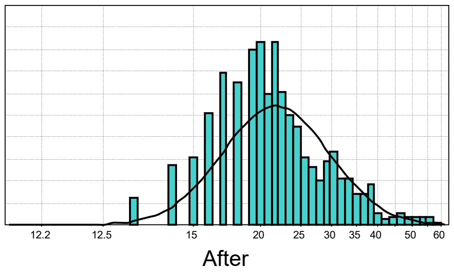

AST F n=788
Para: 14.01 ~ 21.75 ~ 41.39
Nonpara: 13.93 ~ 21.80 ~ 51.99
Pow=0.295 TPos=12.187
Kurt=-0.146 Skew=0.076
K-S test for normality: 0.00702

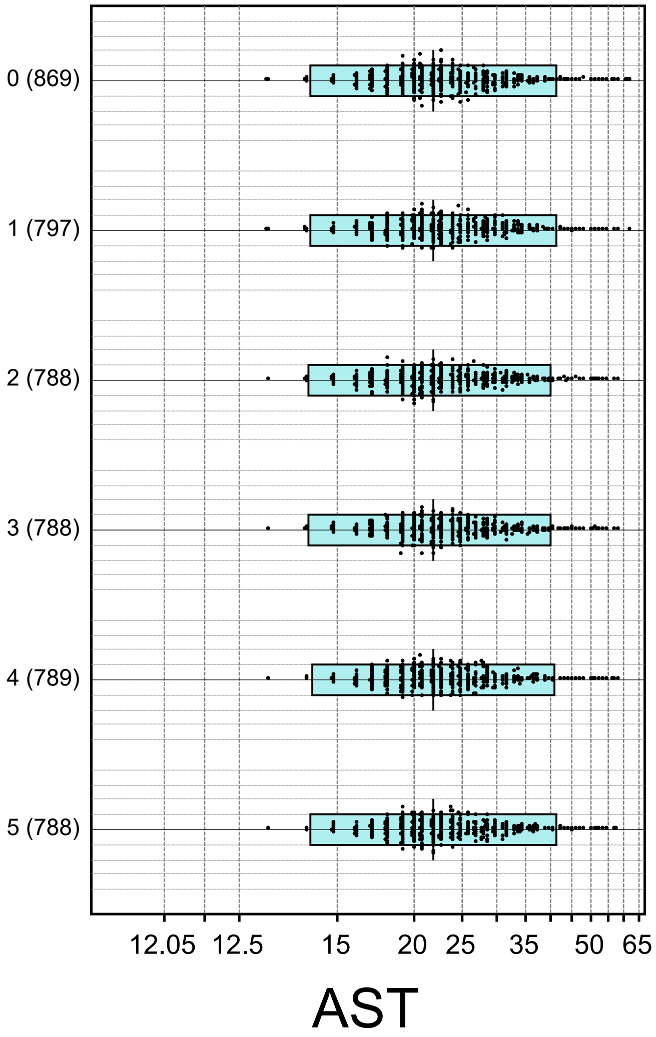

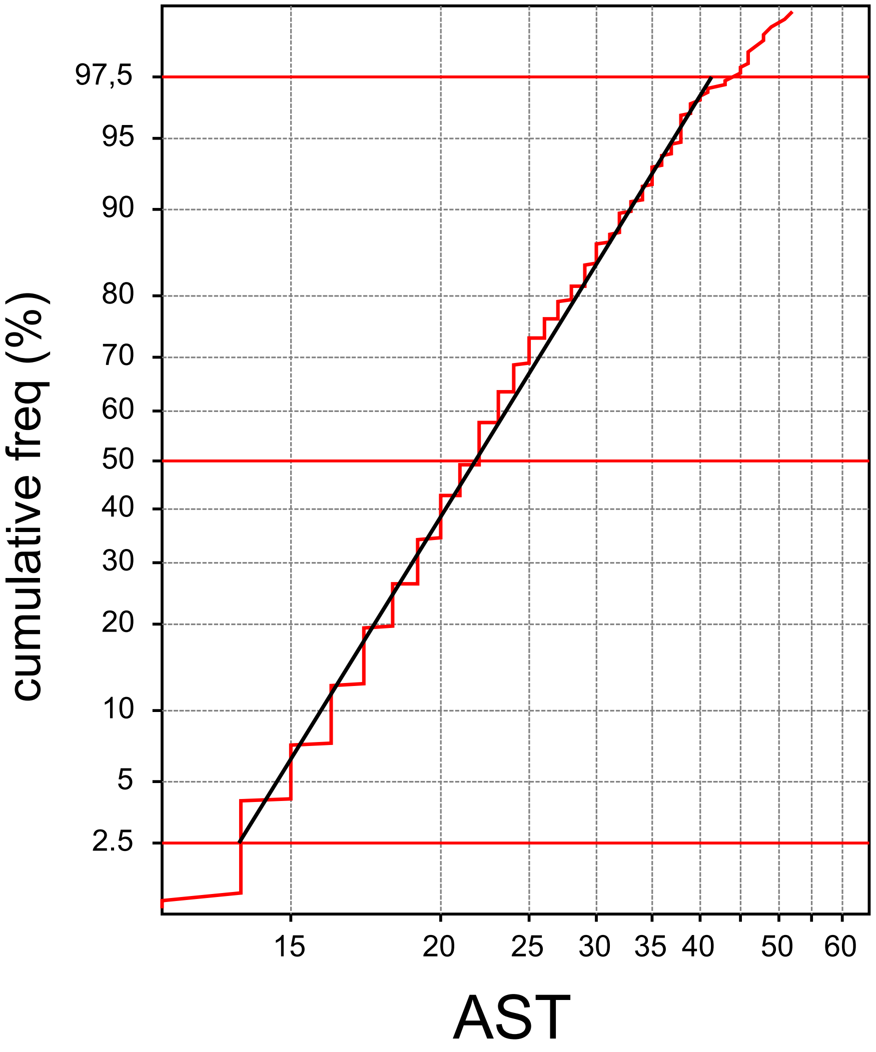

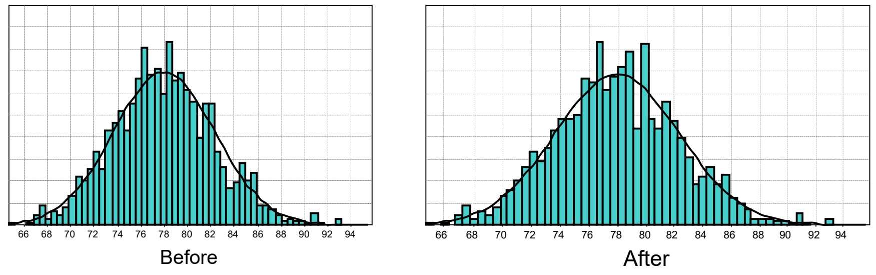

TP MF n=1,440
Para: 69.6 ~ 78.0 ~ 86.5
Nonpara: 69.4 ~ 77.9 ~ 86.8
Pow=0.978 TPos=64.683
Kurt=-0.247 Skew=0.031
K-S test for normality: .441 (NS)

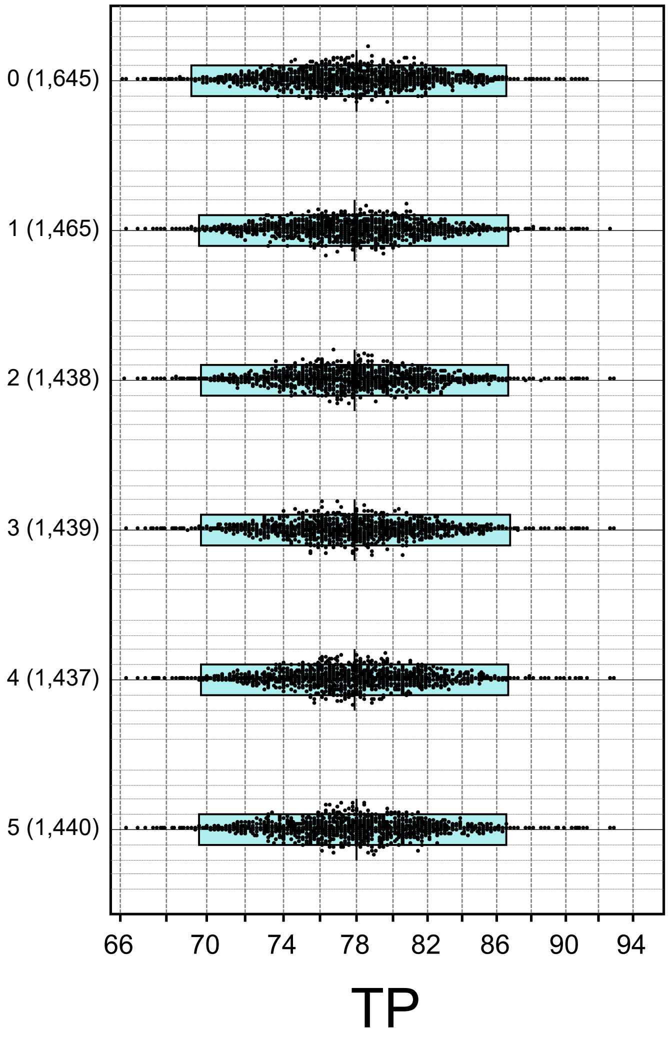

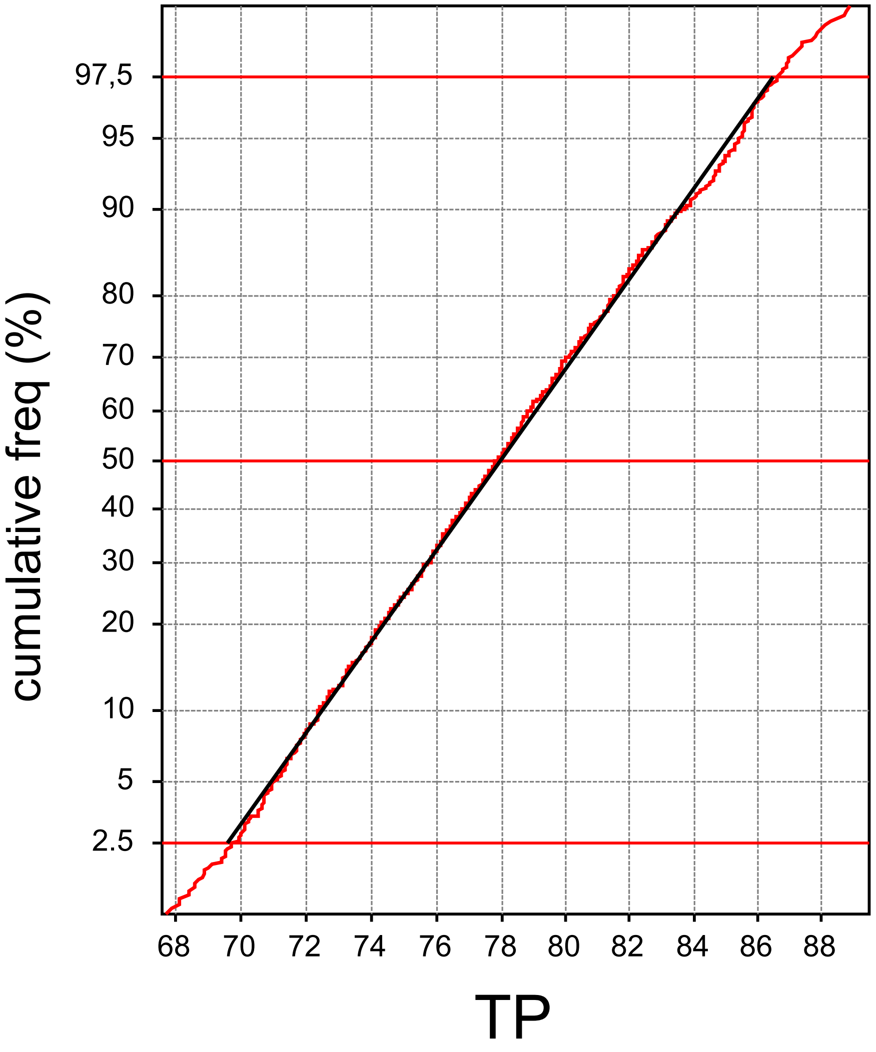

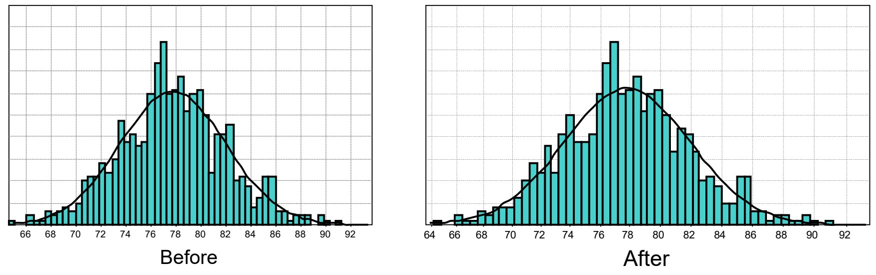

TP M n=671
Para: 69.3 ~ 77.8 ~ 85.9
Nonpara: 69.5 ~ 77.7 ~ 86.4
Pow=1.071 TPos=63.391
Kurt=-0.267 Skew=0.058
K-S test for normality: .6 (NS)

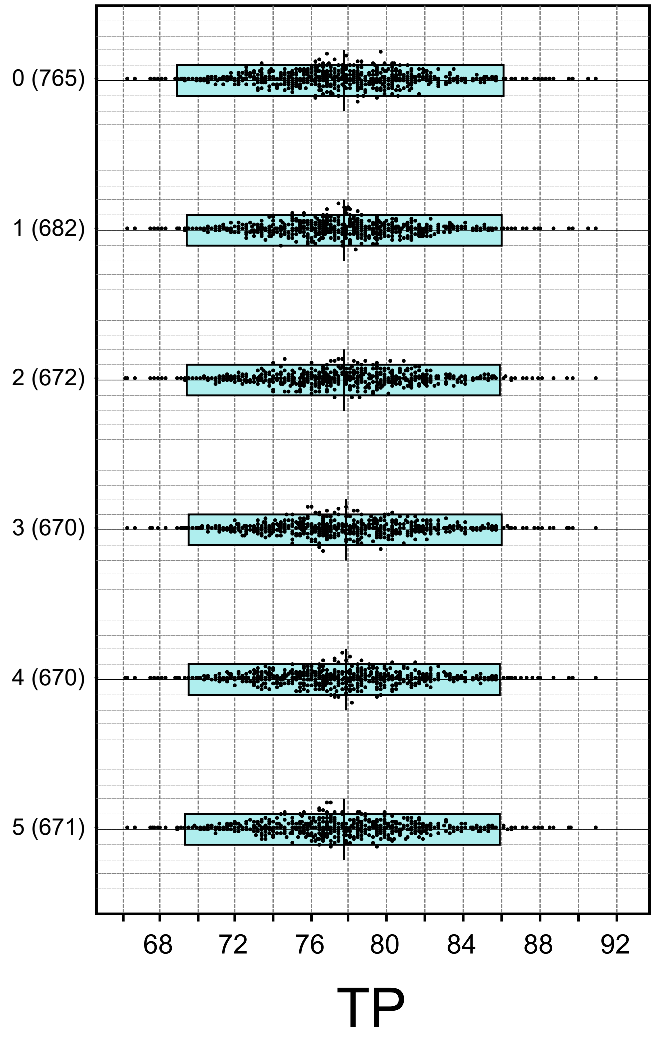

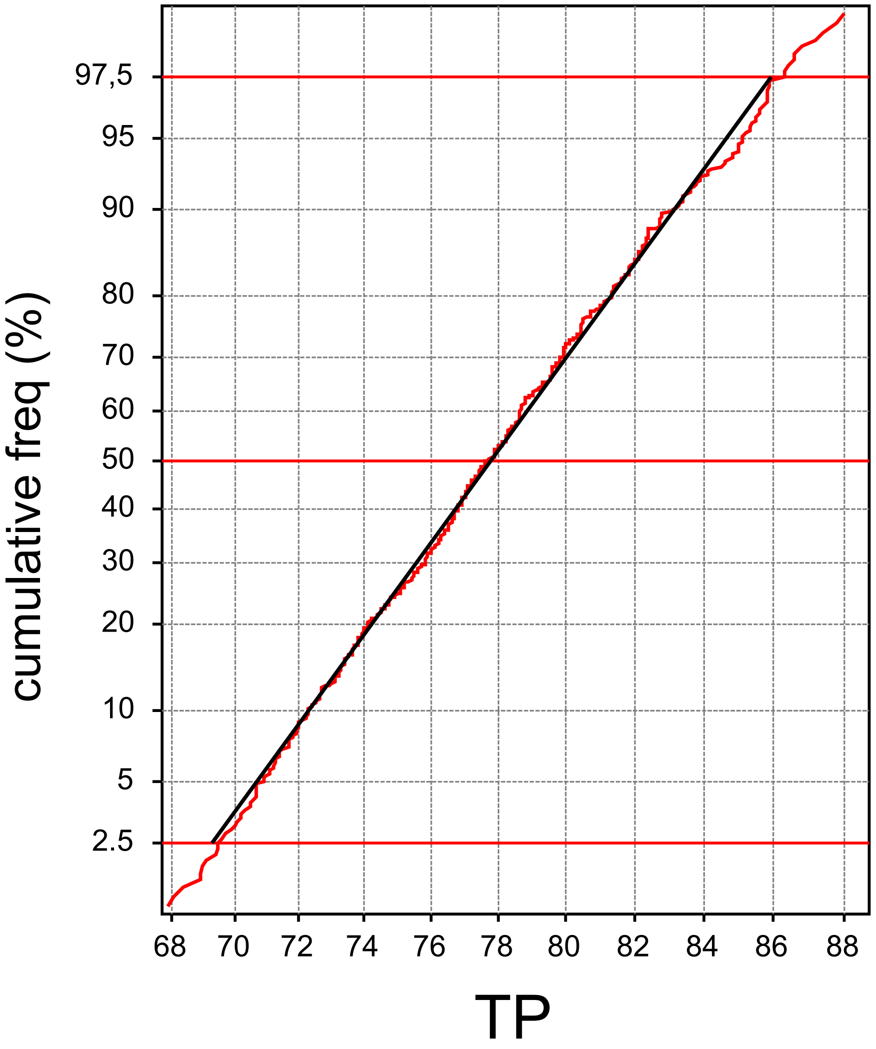

TP F n=774
Para: 69.7 ~ 78.2 ~ 86.9
Nonpara: 69.3 ~ 78.1 ~ 86.9
Pow=0.983 TPos=64.438
Kurt=-0.271 Skew=0.043
K-S test for normality: P≈1.00 (N.S.)

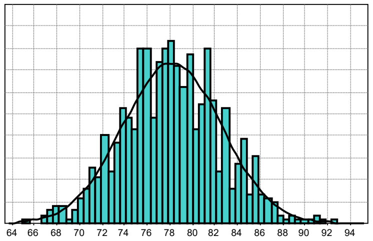

Before

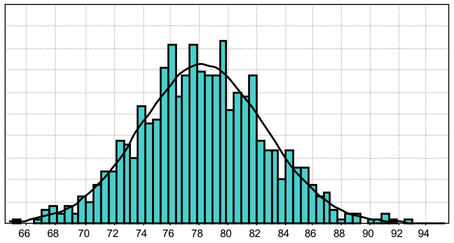

After

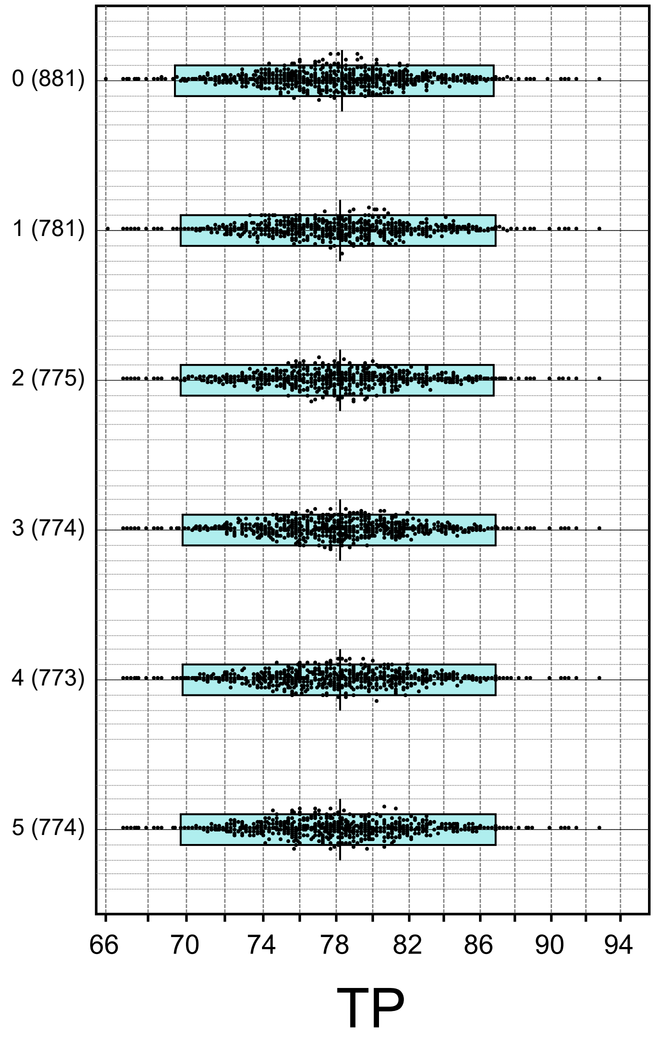

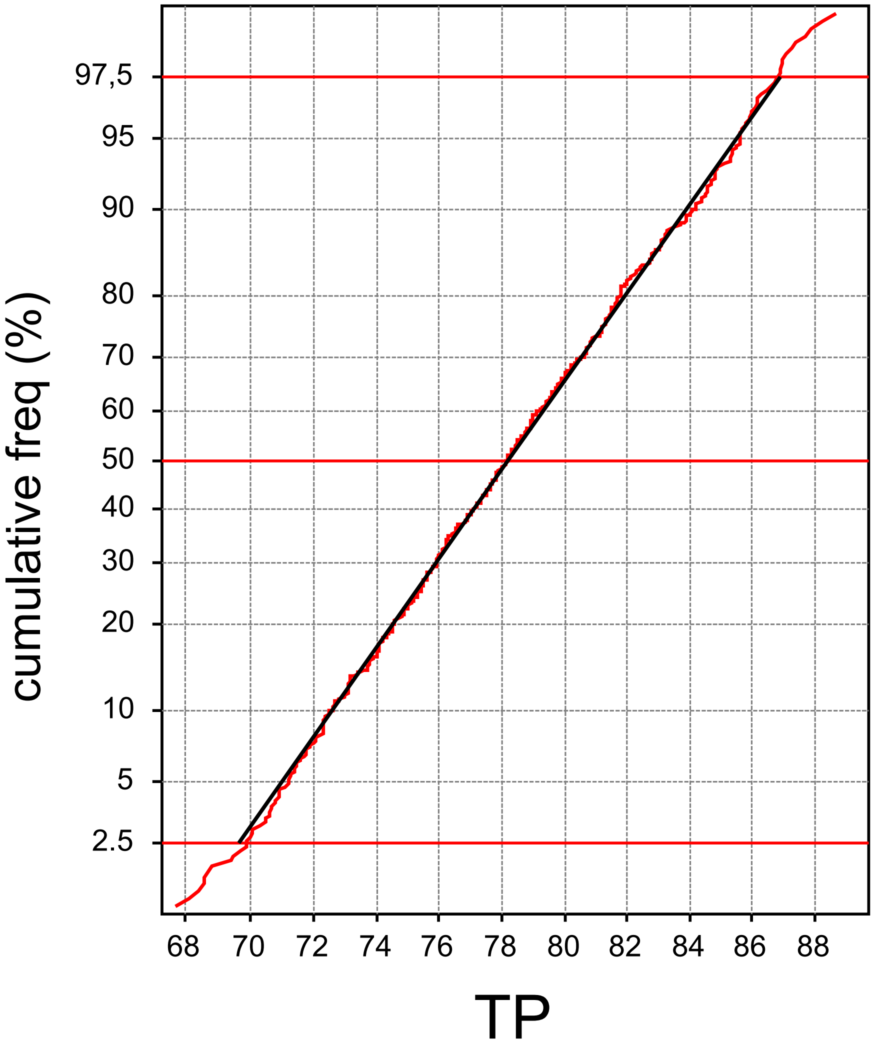

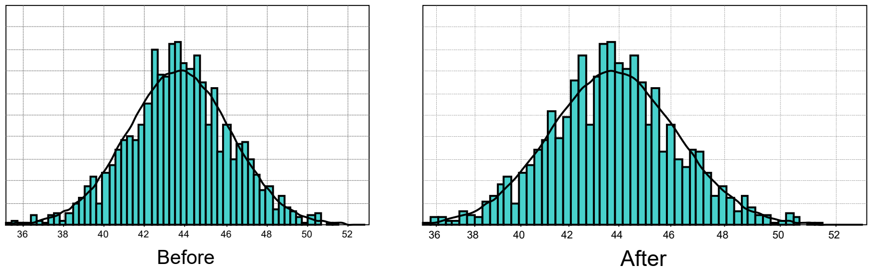

ALB MF n=1,445
Para: 38.70 ~ 43.79 ~ 48.43
Nonpara: 38.57 ~ 43.71 ~ 48.65
Pow=1.161 TPos=35.163
Kurt=-0.277 Skew=0.069
K-S test for normality: .277 (NS)

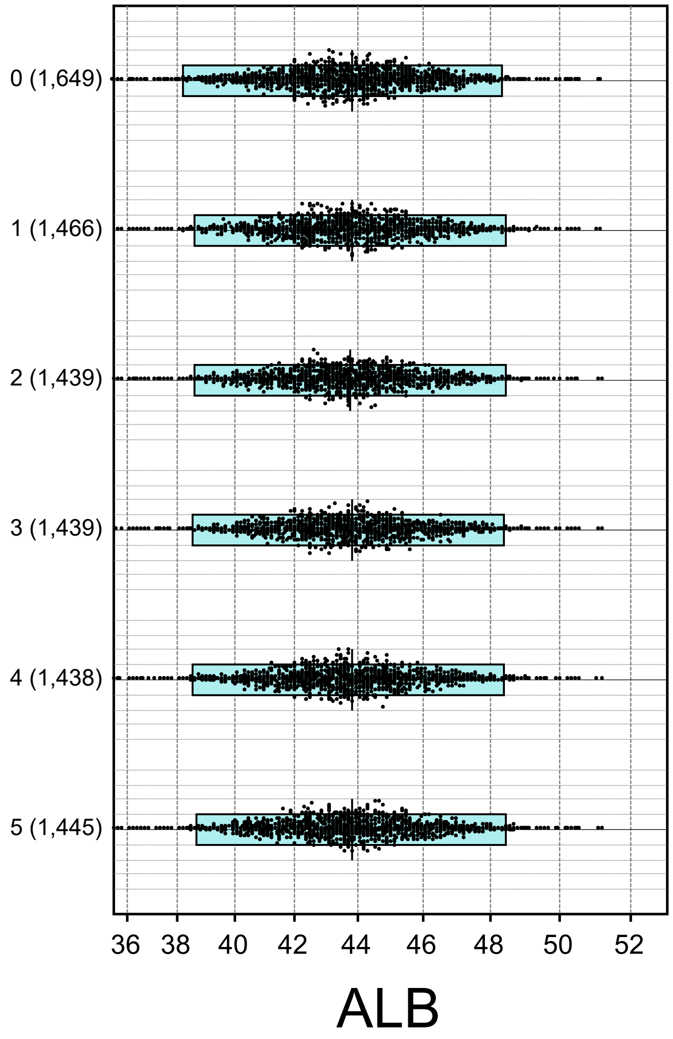

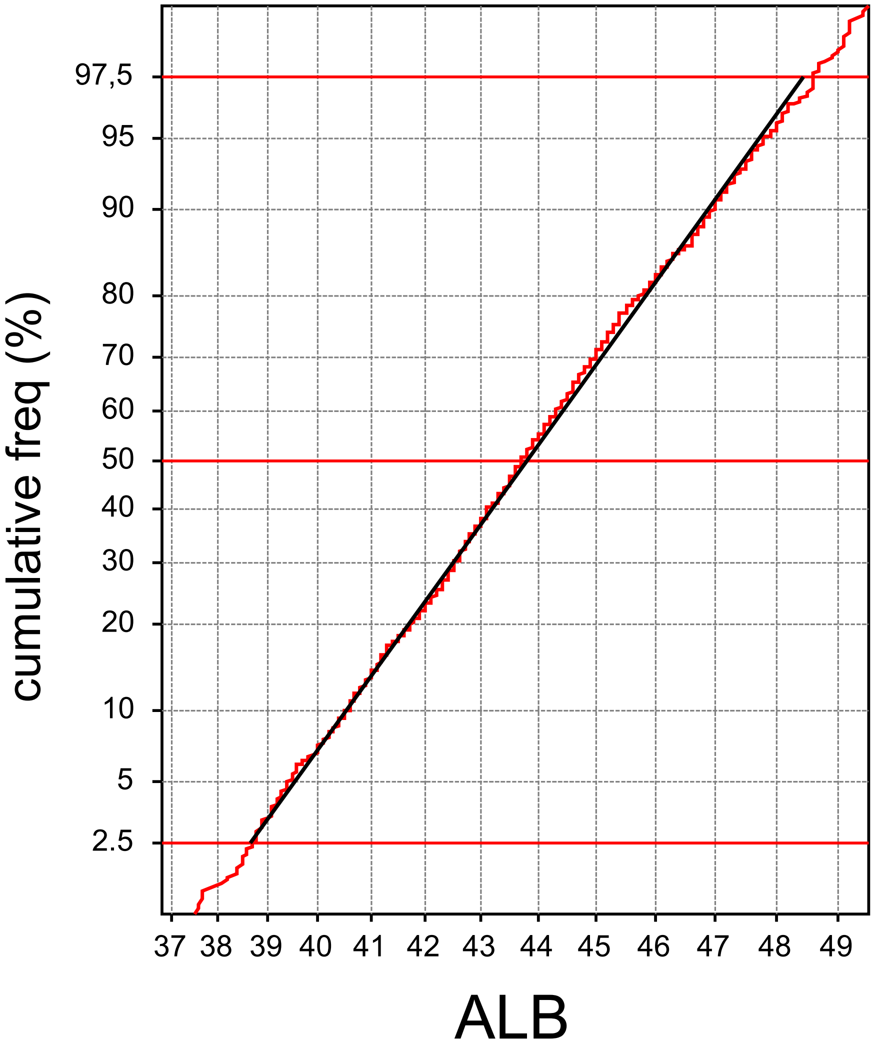

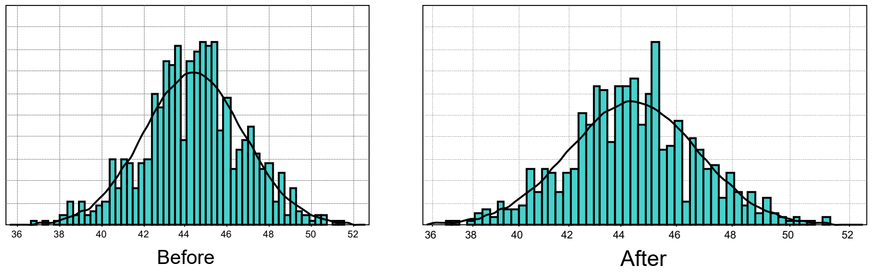

ALB M n=673
Para: 39.58 ~ 44.47 ~ 48.96
Nonpara: 39.28 ~ 44.44 ~ 49.06
Pow=1.177 TPos=35.167
Kurt=-0.219 Skew=0.03
K-S test for normality: .226 (NS)

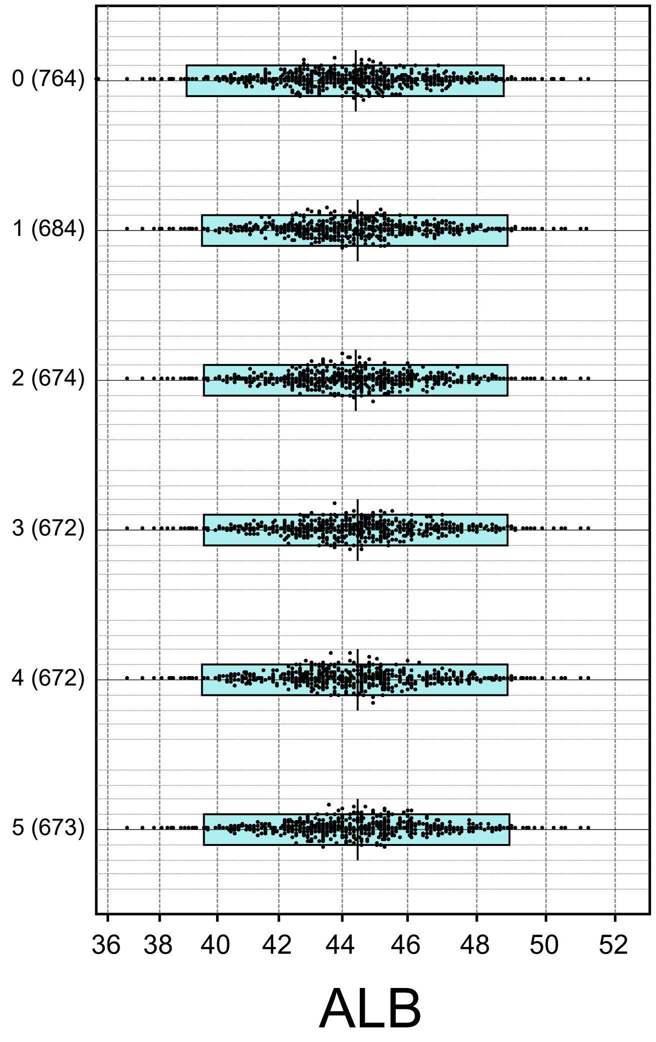

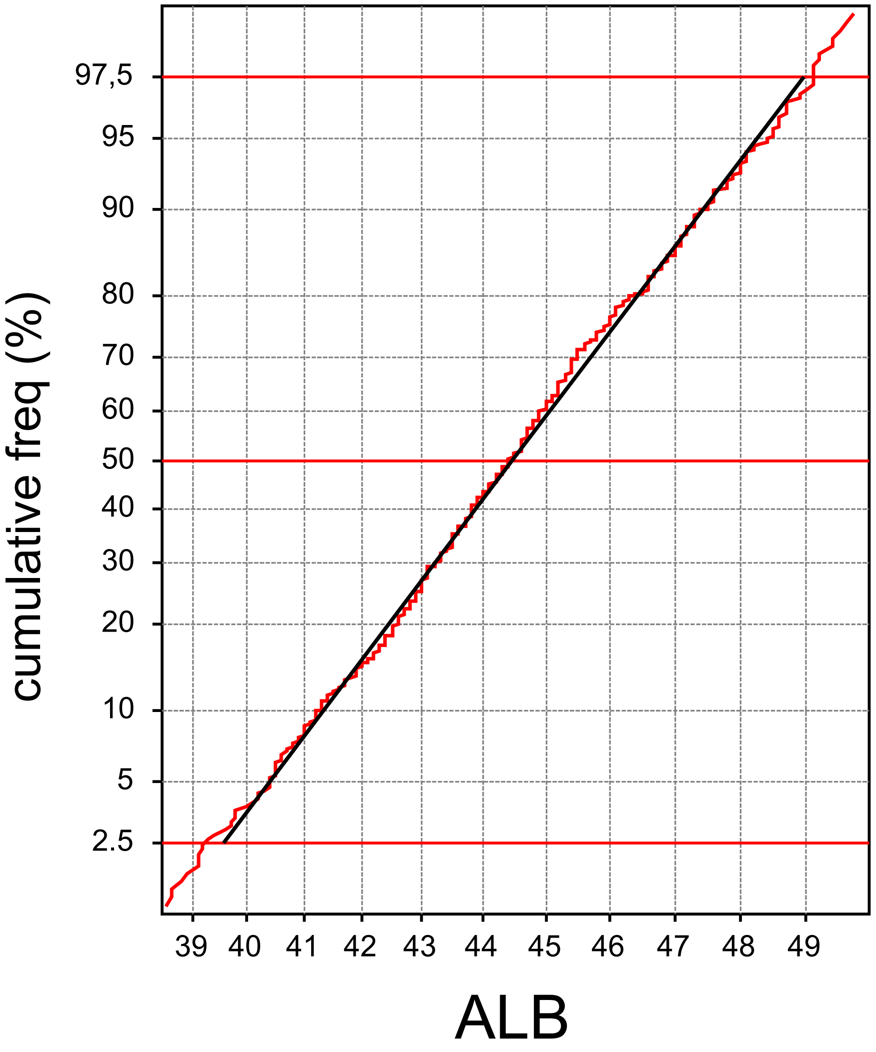

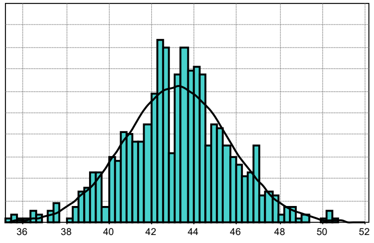

Before

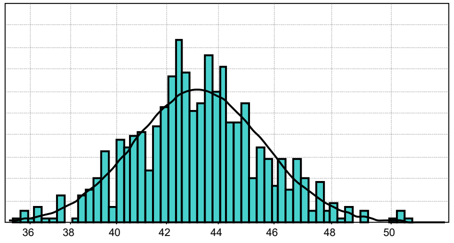

After

ALB F n=777
Para: 38.09 ~ 43.27 ~ 47.81
Nonpara: 37.92 ~ 43.20 ~ 47.93
Pow=1.233 TPos=34.164
Kurt=-0.268 Skew=0.059
K-S test for normality: .377 (NS)

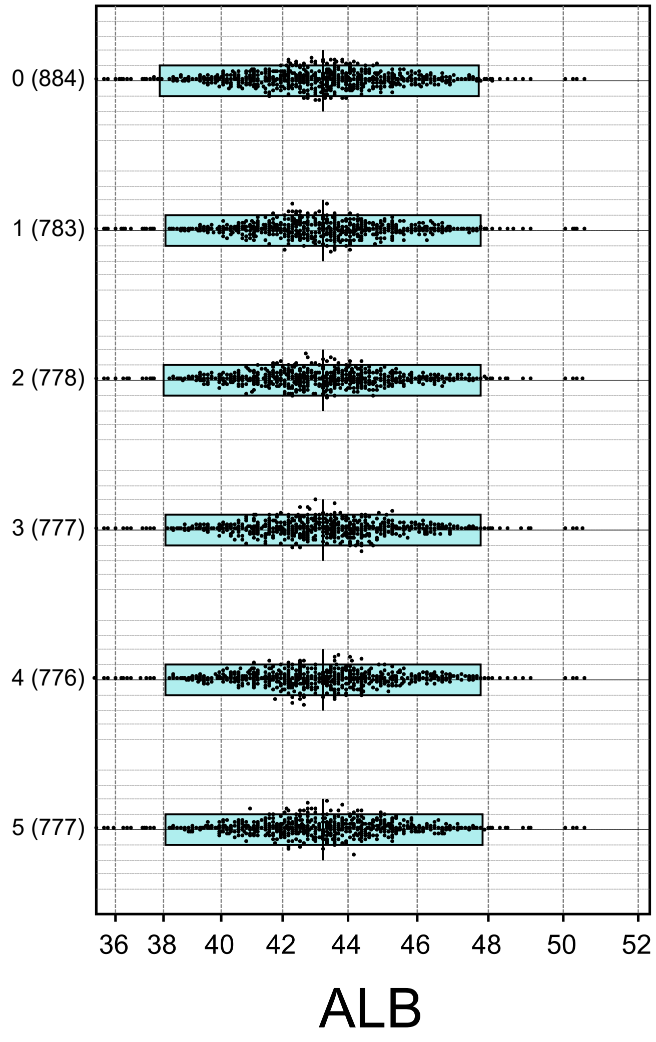

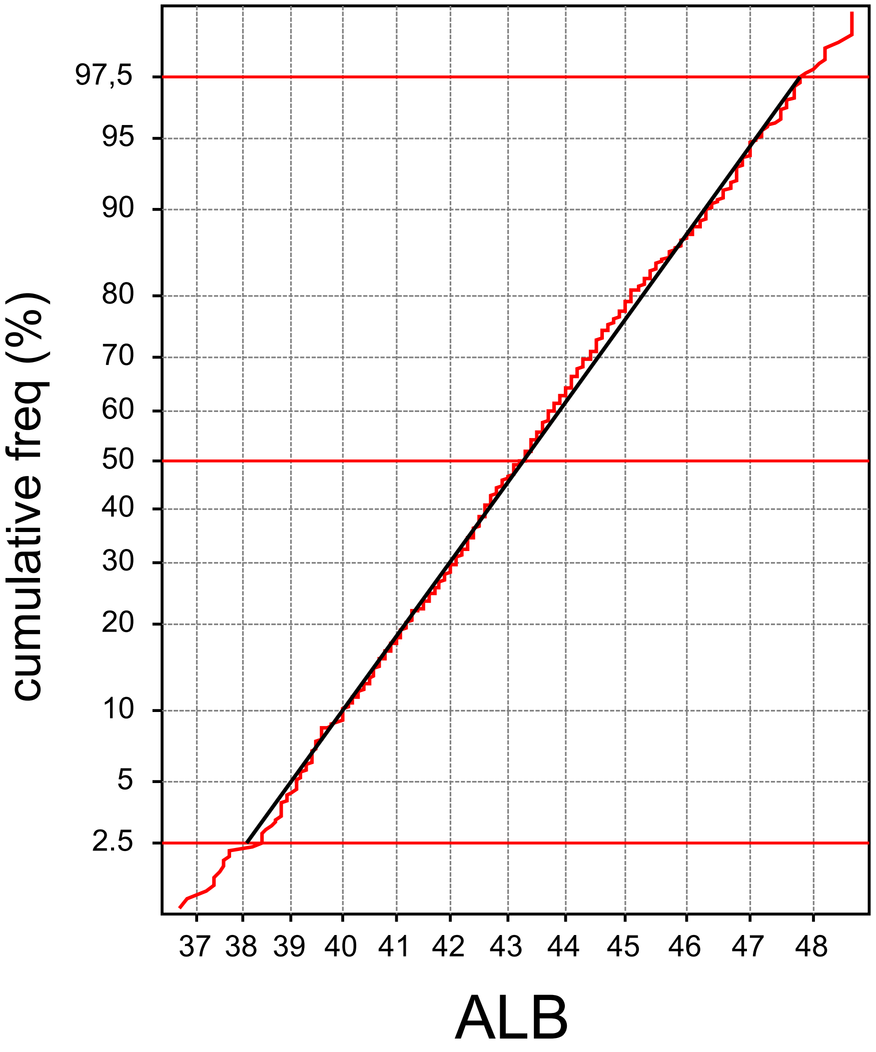

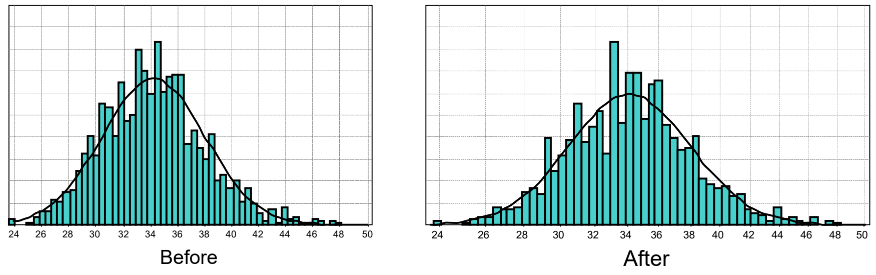

GLB MF n=1,442
Para: 27.25 ~ 34.10 ~ 41.86
Nonpara: 27.13 ~ 34.15 ~ 42.49
Pow=0.838 TPos=23.564
Kurt=-0.286 Skew=-0.048
K-S test for normality: .328 (NS)

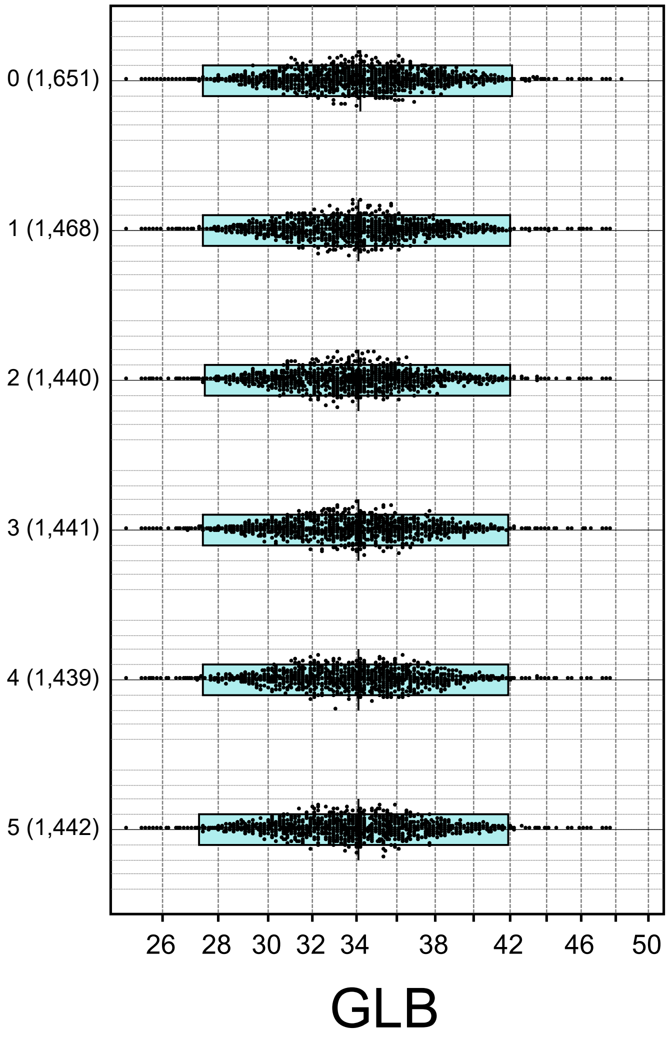

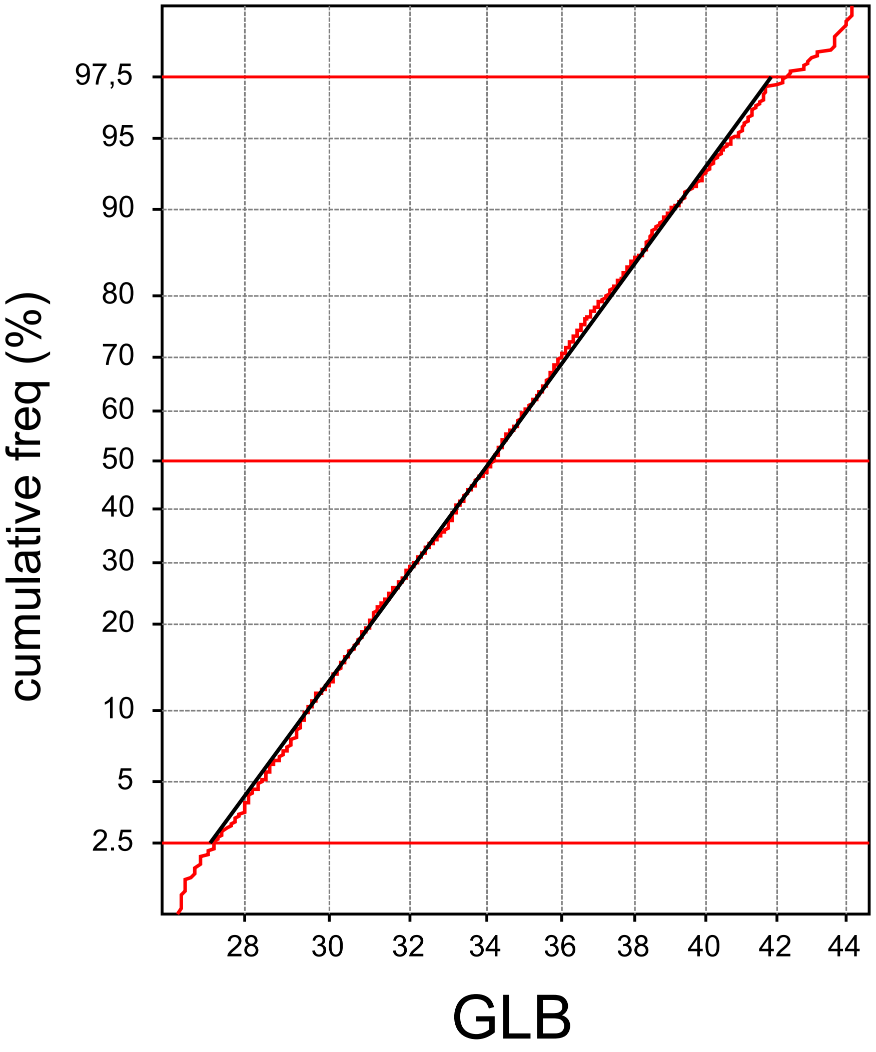

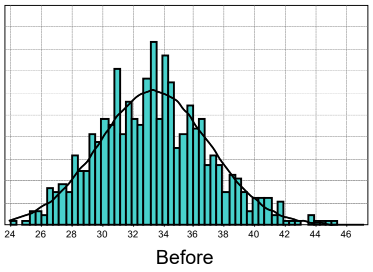

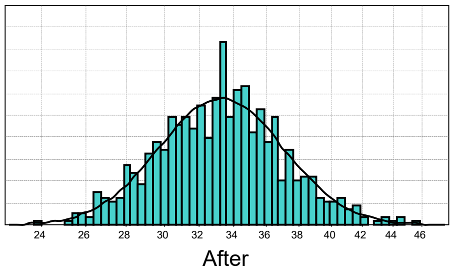

GLB M n=672
Para: 26.62 ~ 33.23 ~ 40.81
Nonpara: 26.74 ~ 33.27 ~ 41.30
Pow=0.809 TPos=21.936
Kurt=-0.307 Skew=-0.001
K-S test for normality: .938 (NS)

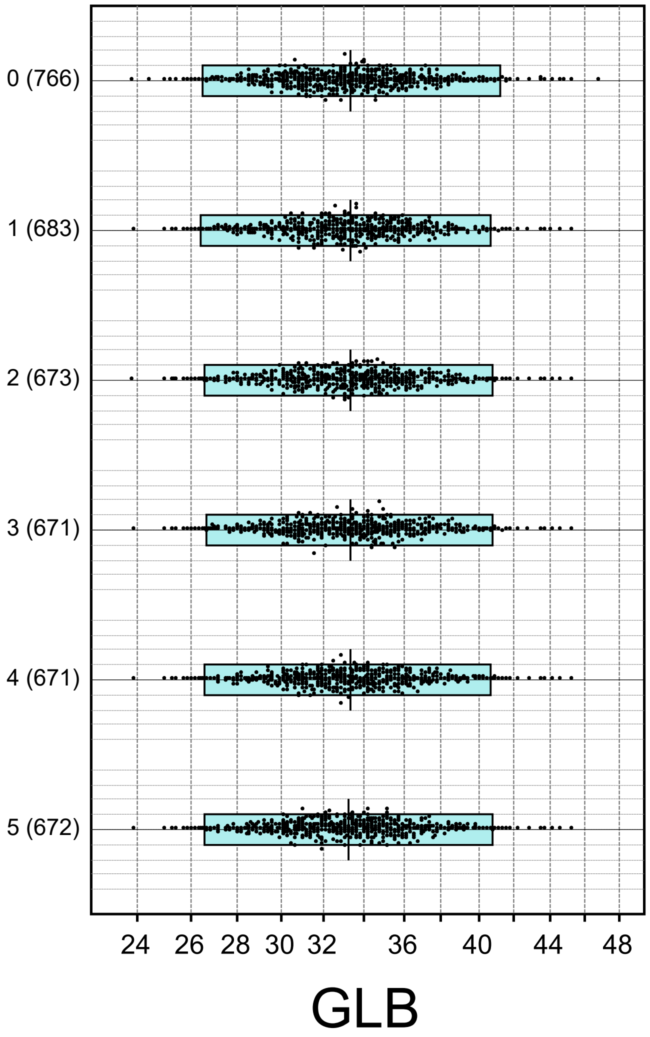

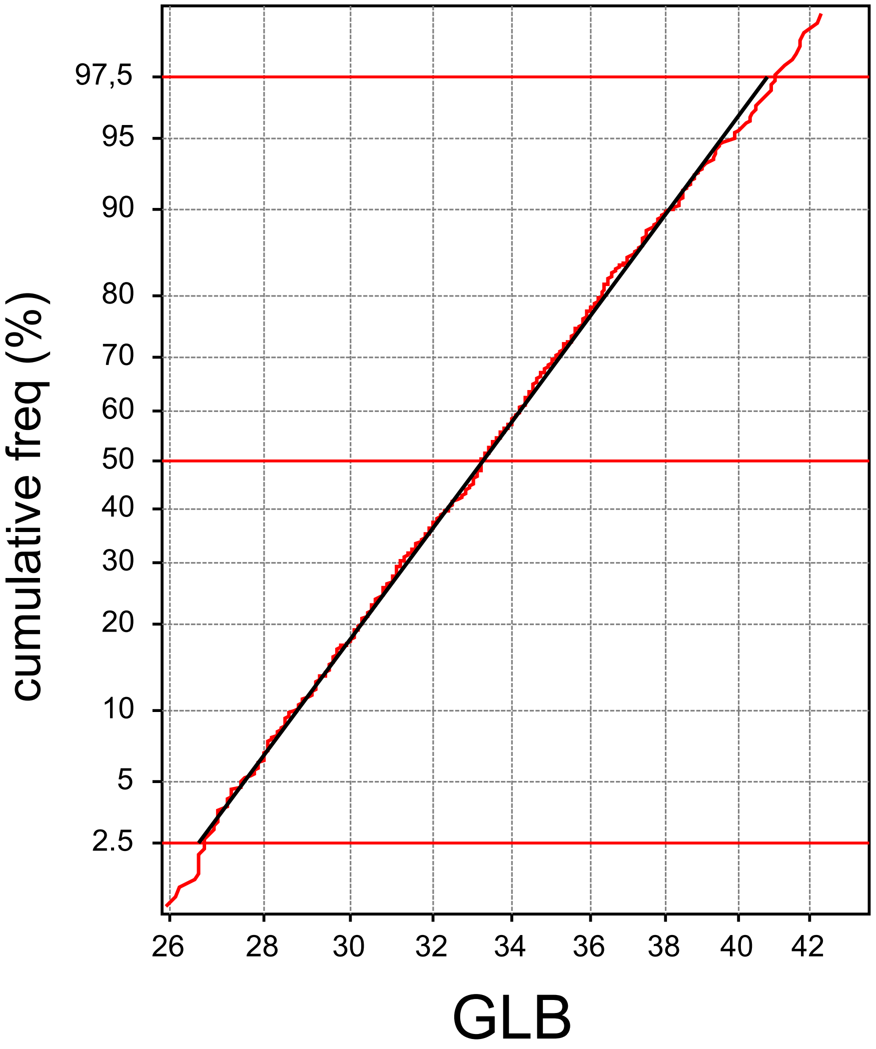

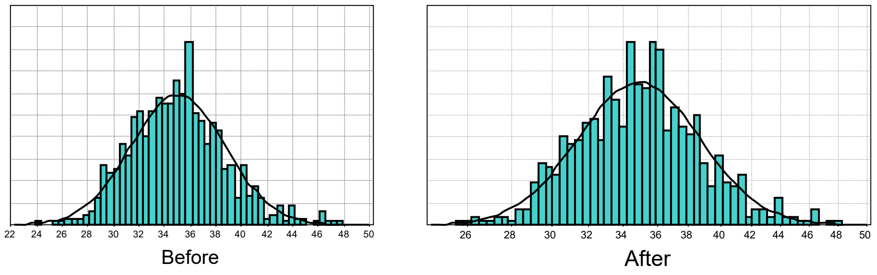

GLB F n=775
Para: 28.37 ~ 34.94 ~ 42.56
Nonpara: 28.56 ~ 34.93 ~ 43.48
Pow=0.804 TPos=24.729
Kurt=-0.226 Skew=0.039
K-S test for normality: .57 (NS)

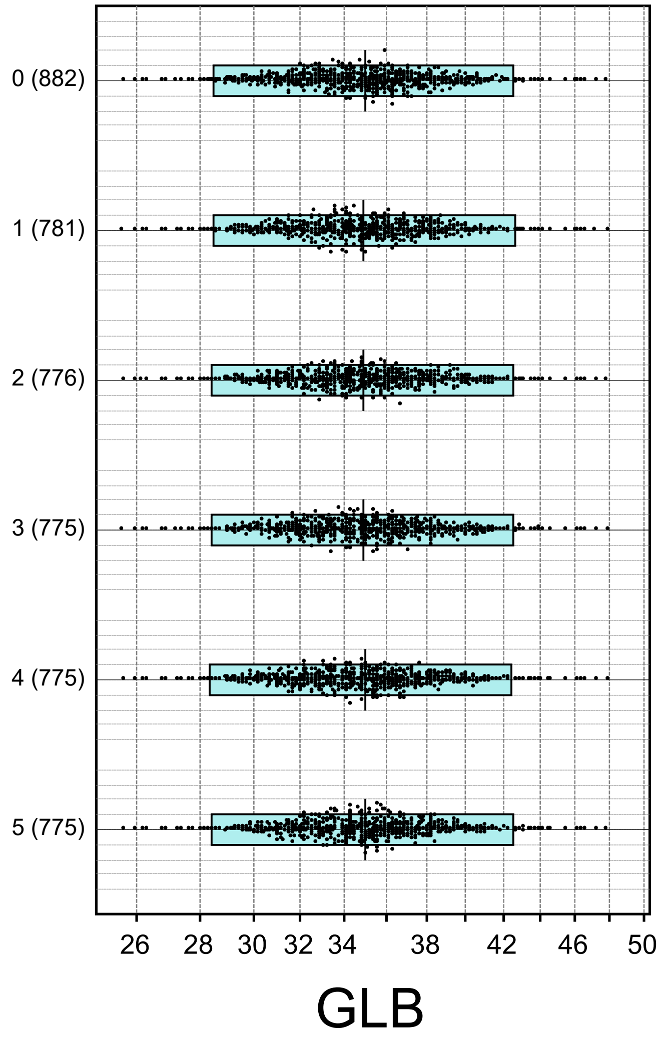

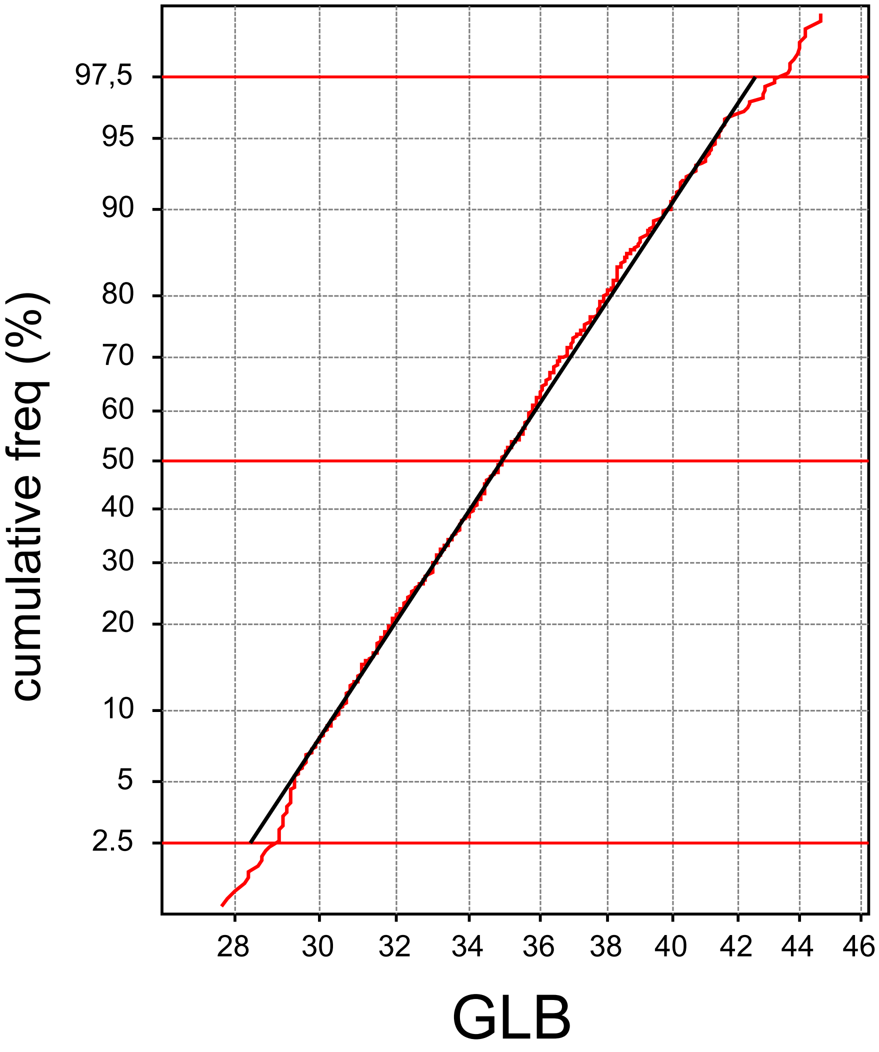

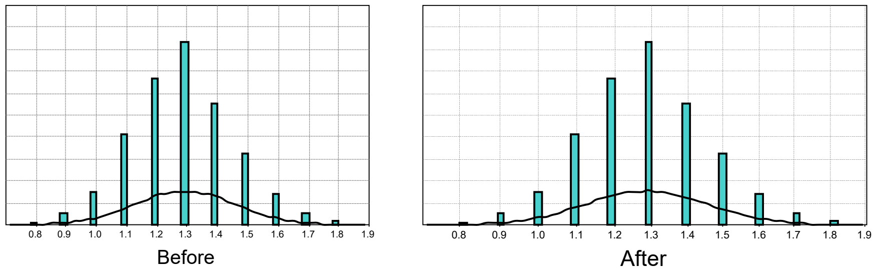

AG MF n=1,444
Para: 0.969 ~ 1.289 ~ 1.623
Nonpara: 0.997 ~ 1.300 ~ 1.620
Pow=0.931 TPos=0.693
Kurt=-0.32 Skew=0.113
K-S test for normality: 0

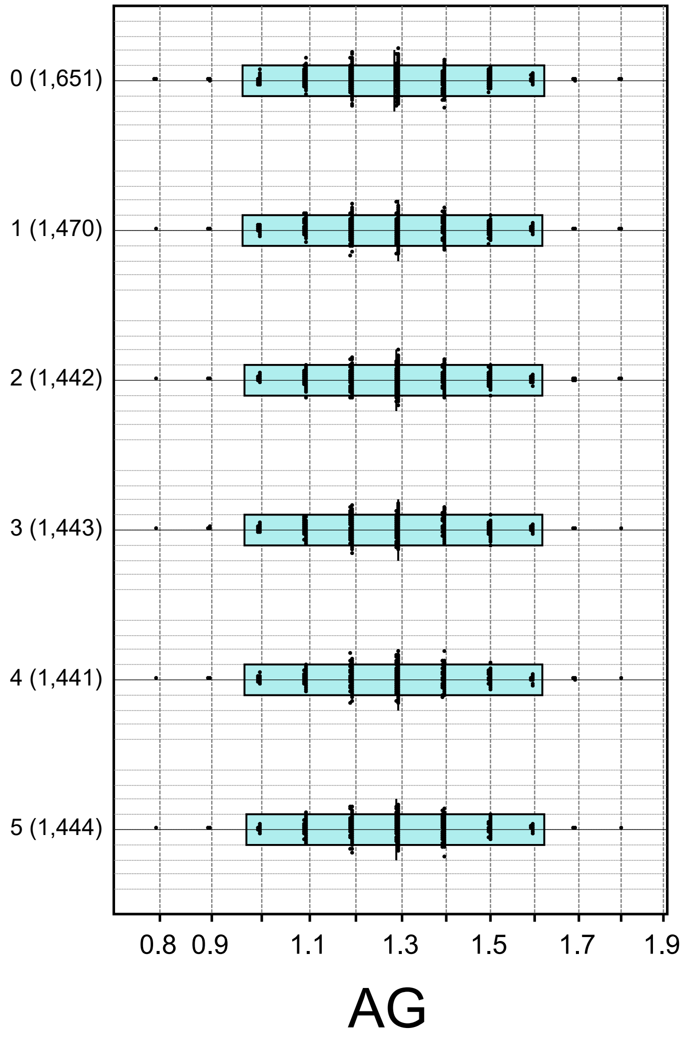

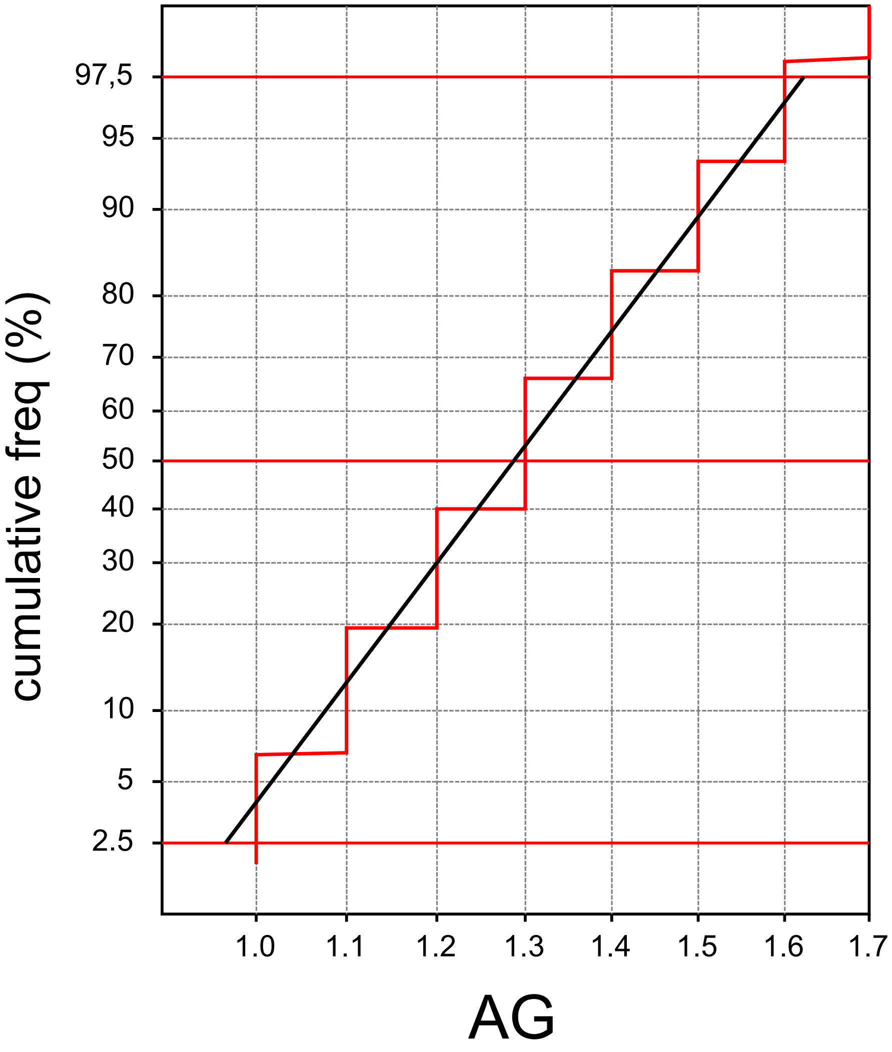

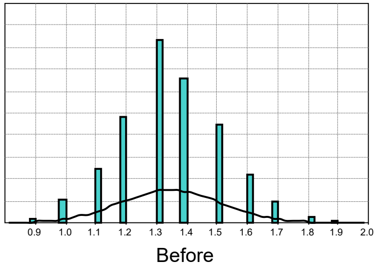

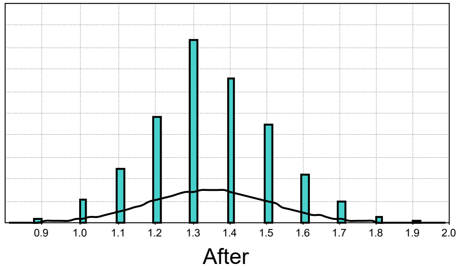

AG M n=673
Para: 1.020 ~ 1.344 ~ 1.678
Nonpara: 1.000 ~ 1.300 ~ 1.700
Pow=0.967 TPos=0.769
Kurt=-0.113 Skew=0.131
K-S test for normality: 0

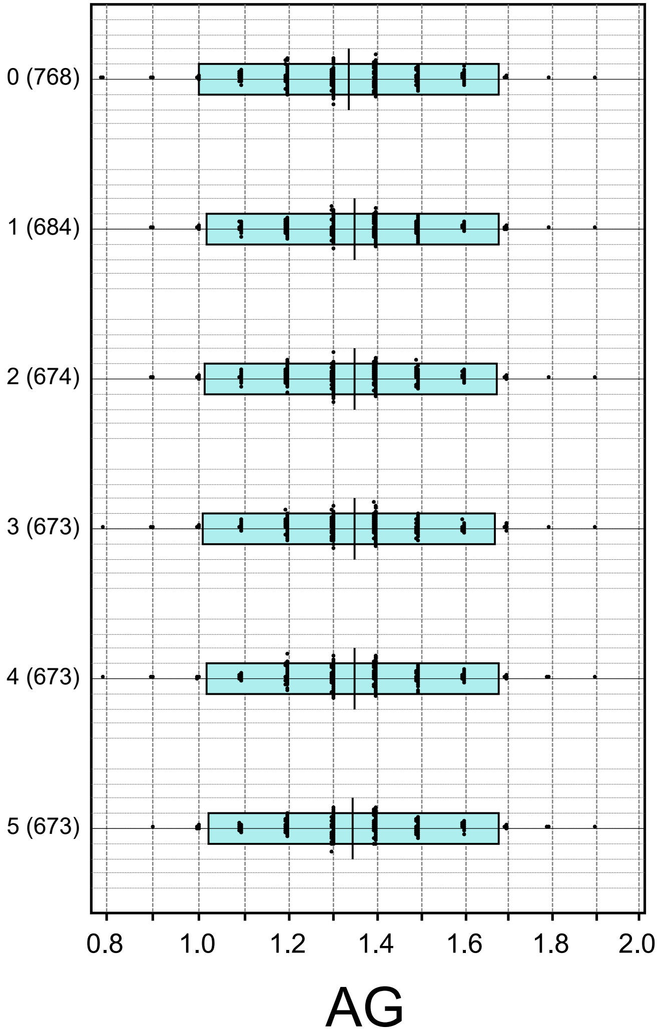

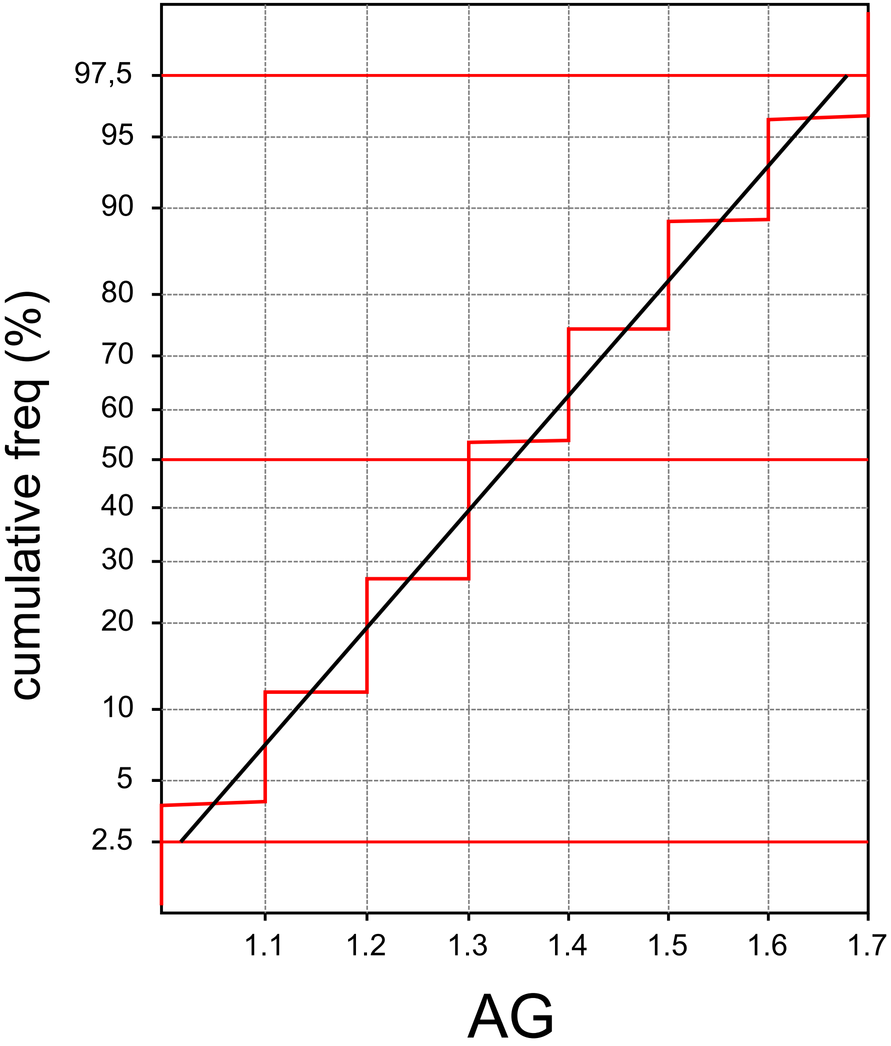

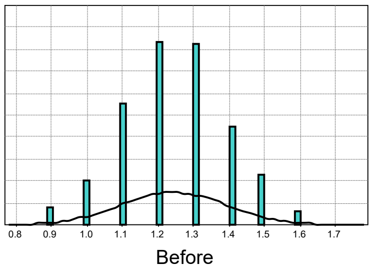

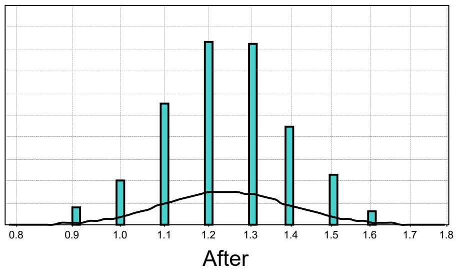

AG F n=777
Para: 0.960 ~ 1.240 ~ 1.552
Nonpara: 0.937 ~ 1.210 ~ 1.543
Pow=0.867 TPos=0.78
Kurt=-0.165 Skew=-0.104
K-S test for normality: 0

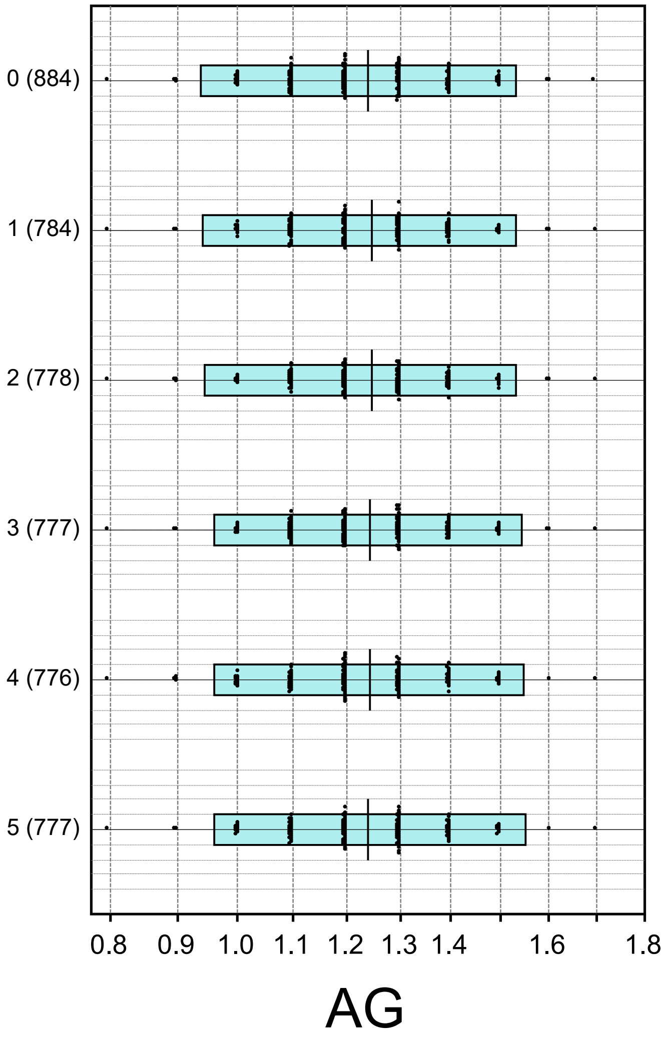

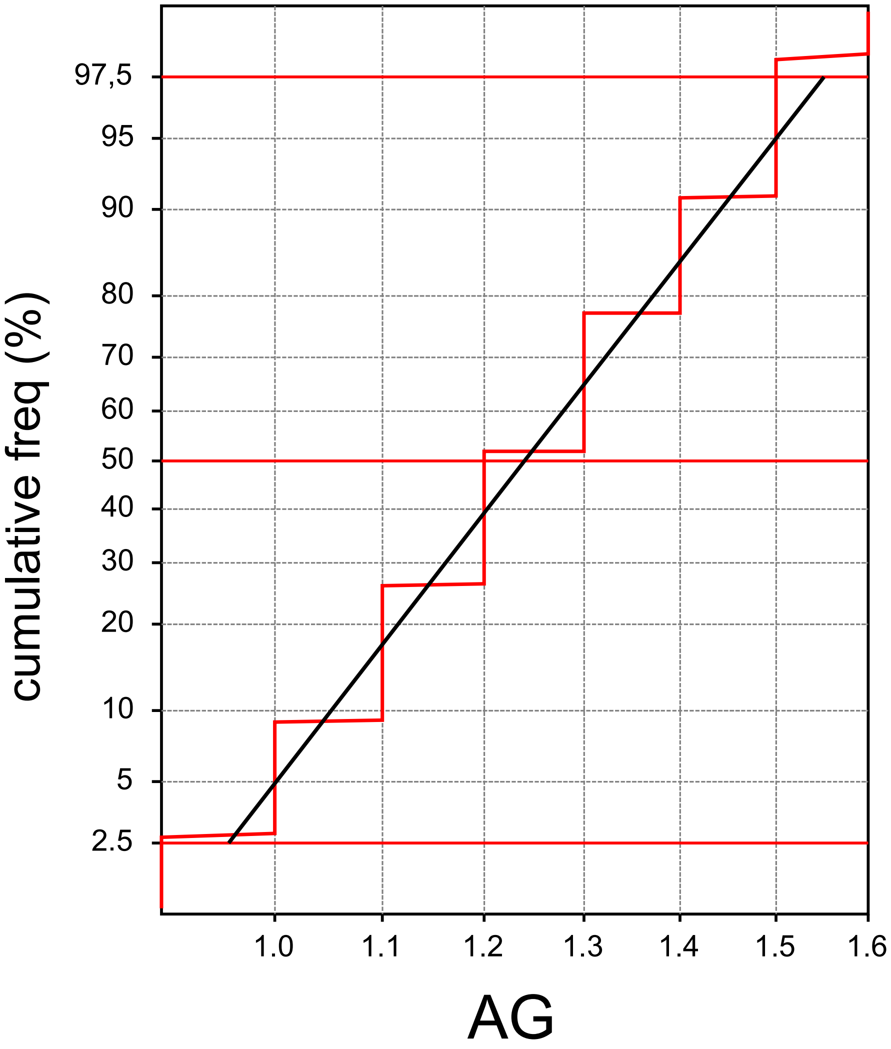

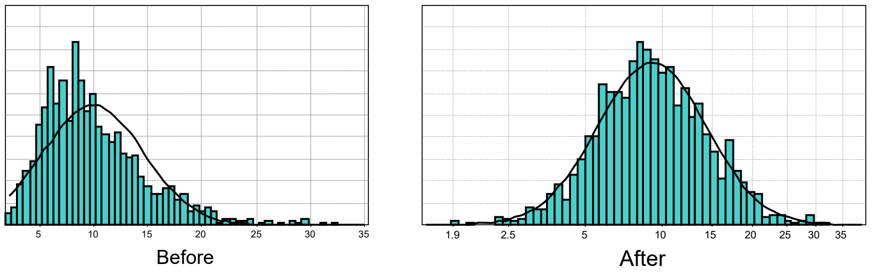

TBIL MF n=1,423
Para: 3.44 ~ 9.20 ~ 20.85
Nonpara: 3.18 ~ 9.02 ~ 21.56
Pow=0.396 TPos=1.848
Kurt=-0.241 Skew=0.073
K-S test for normality: .471 (NS)

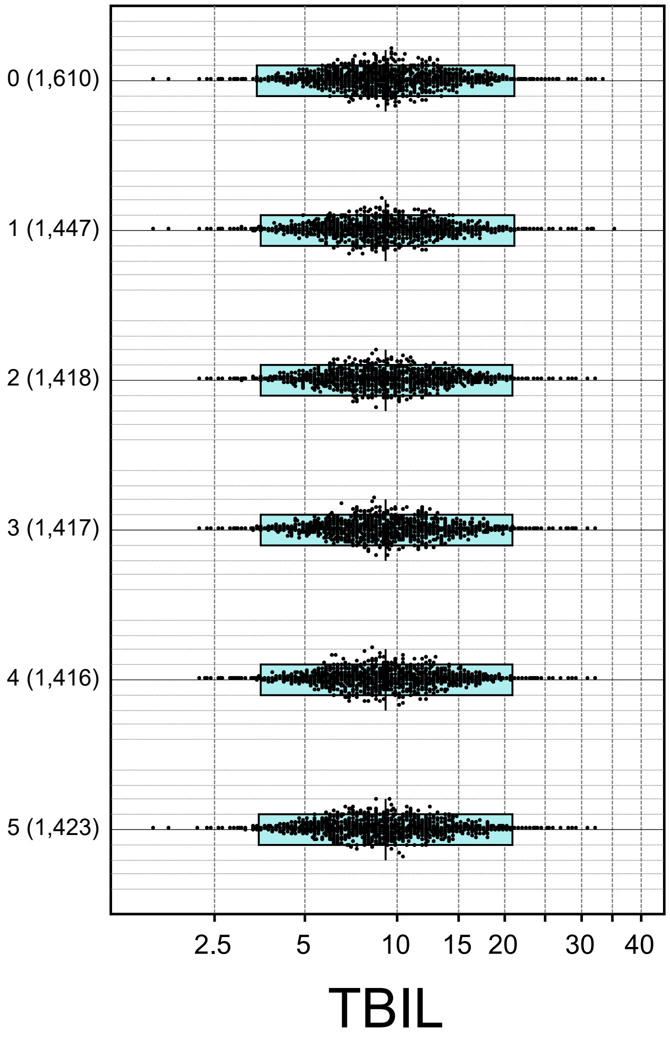

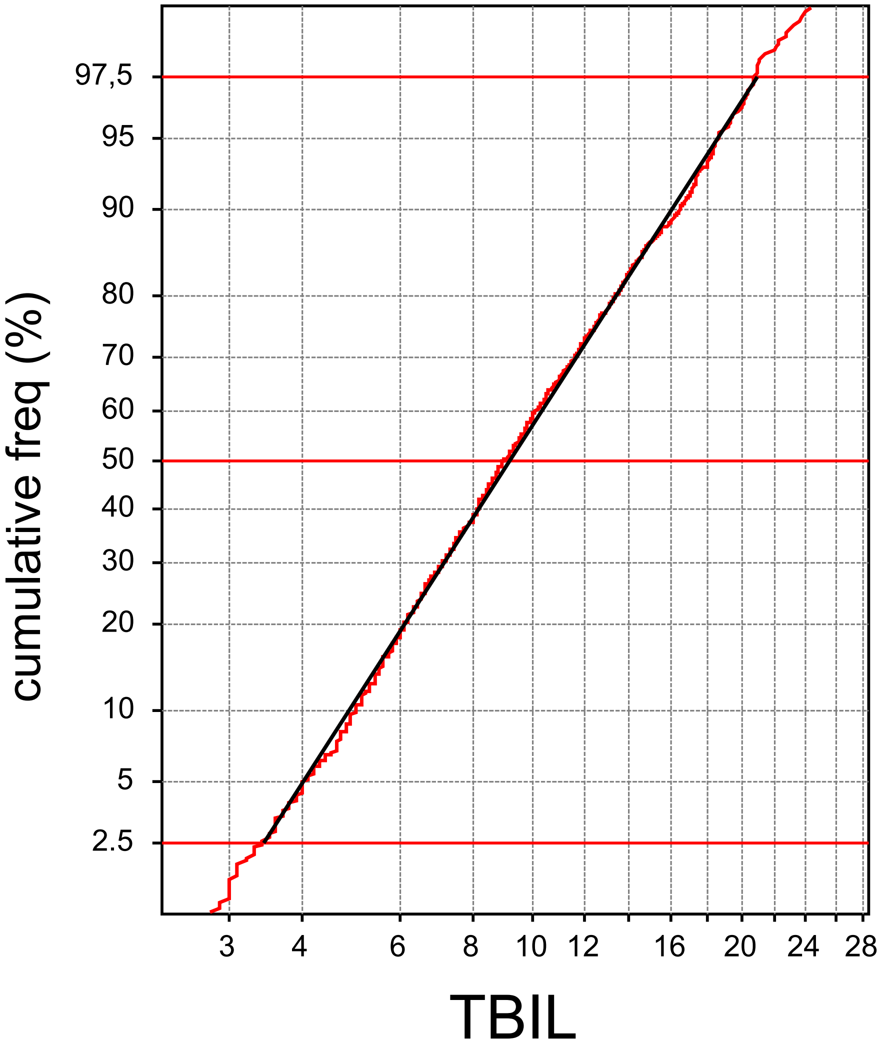

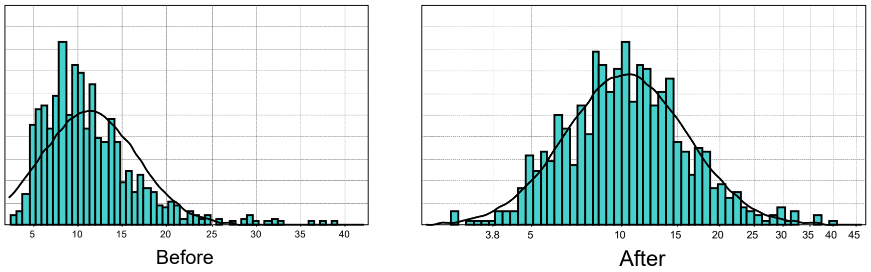

TBIL M n=675
Para: 4.11 ~ 10.27 ~ 23.91
Nonpara: 3.98 ~ 10.16 ~ 25.58
Pow=0.335 TPos=2.358
Kurt=-0.261 Skew=0.083
K-S test for normality: .988 (NS)

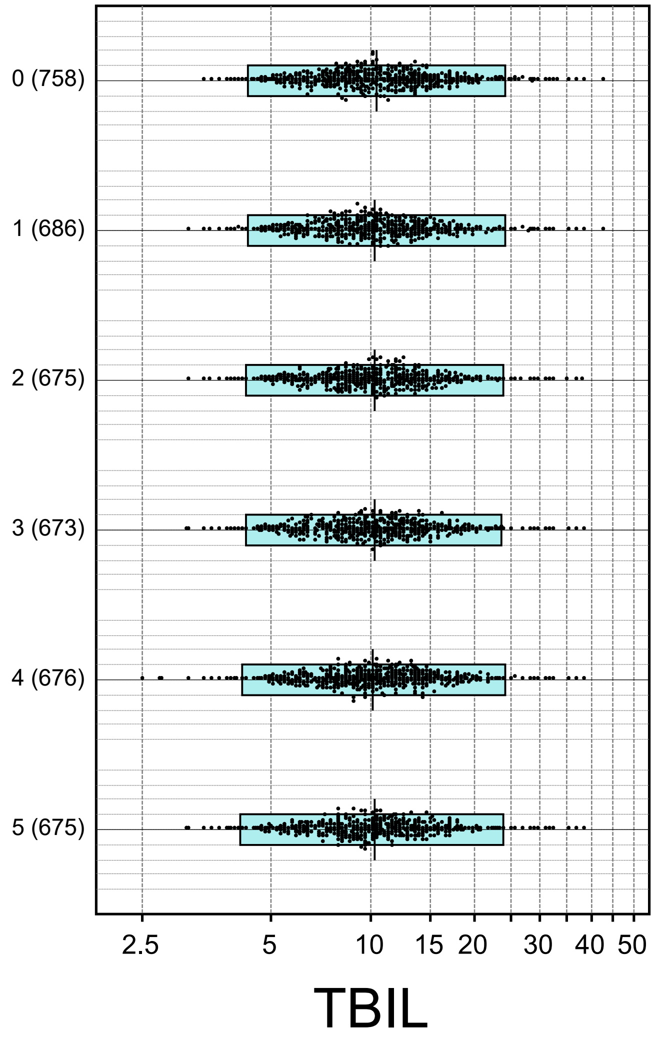

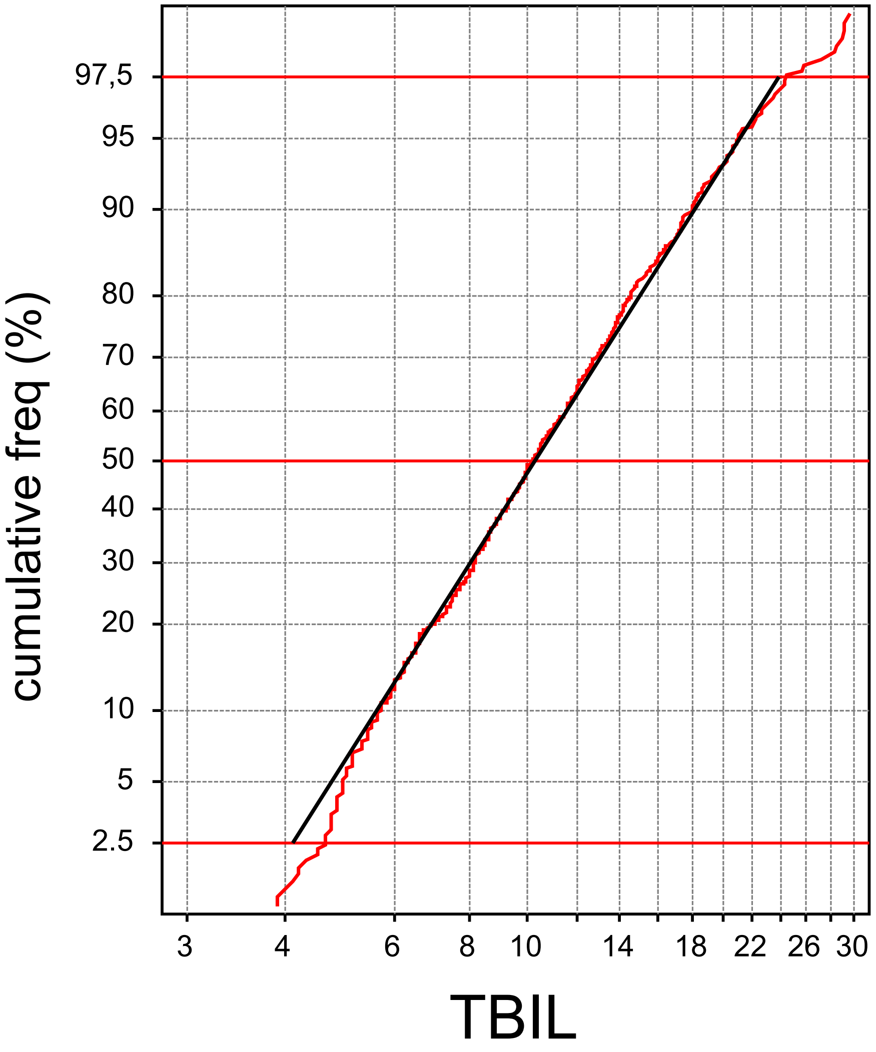

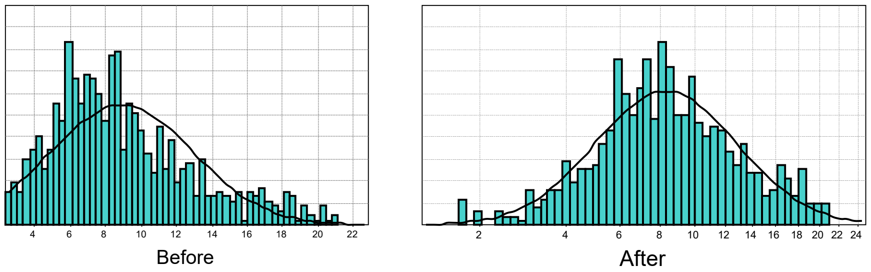

TBIL F n=755
Para: 2.97 ~ 8.27 ~ 18.37
Nonpara: 2.86 ~ 8.22 ~ 18.74
Pow=0.394 TPos=1.085
Kurt=-0.313 Skew=0.118
K-S test for normality: .415 (NS)

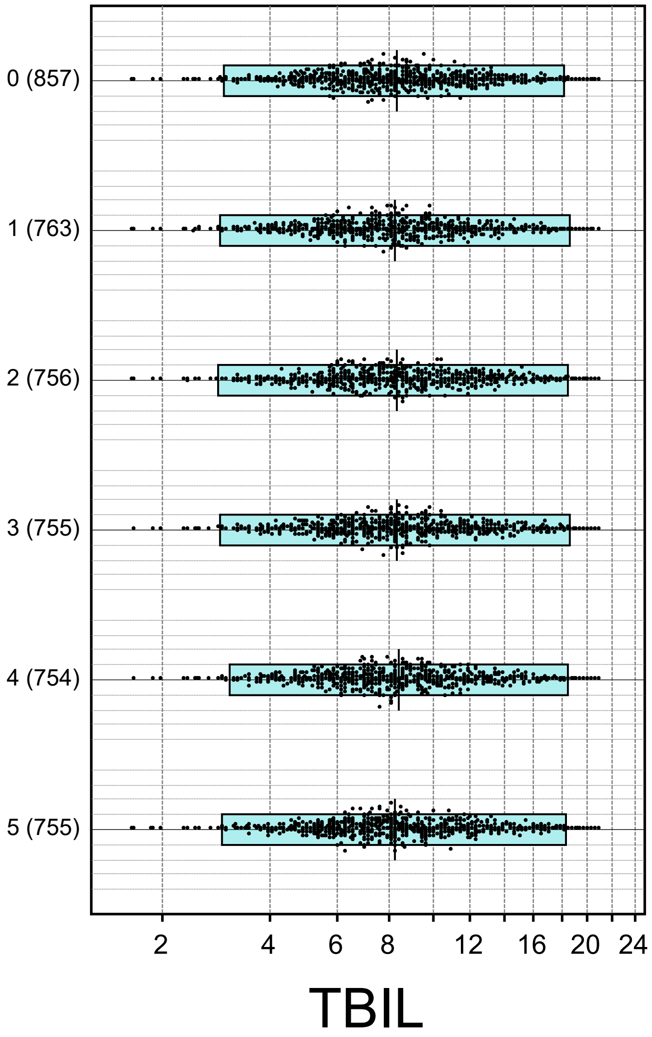

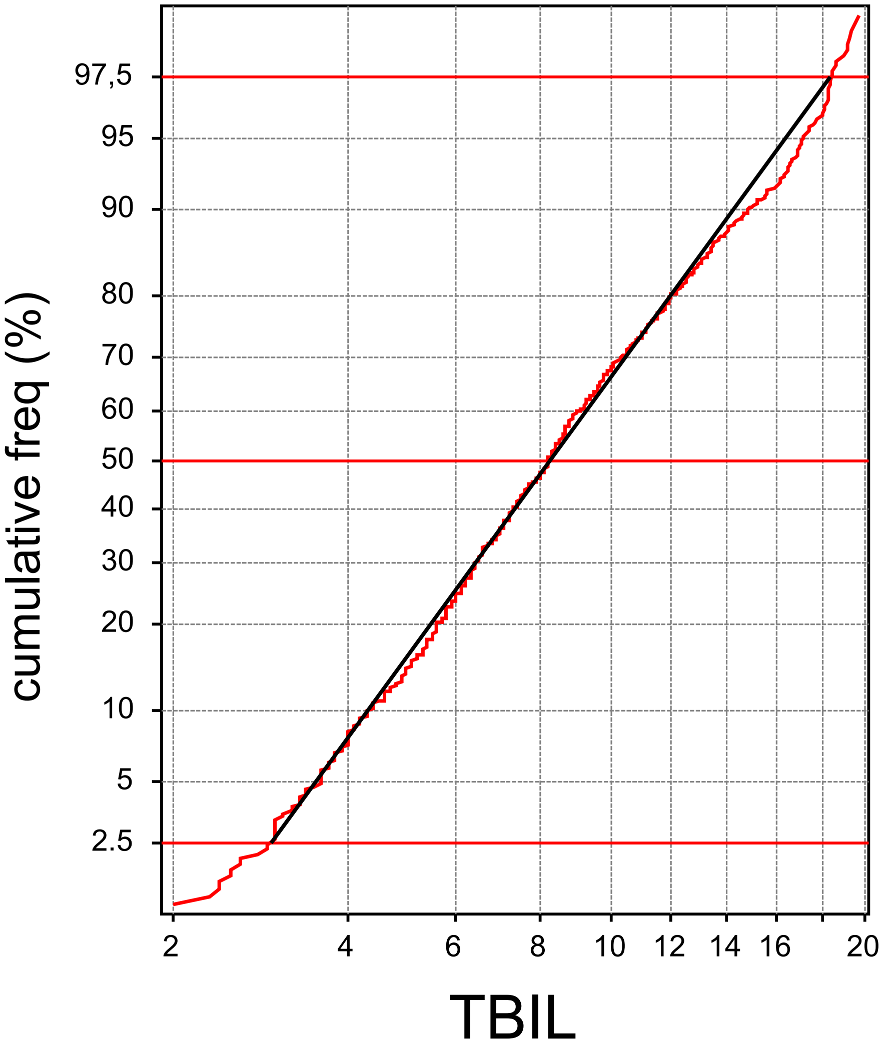

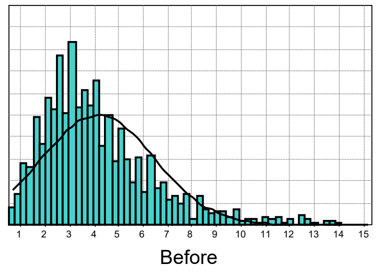

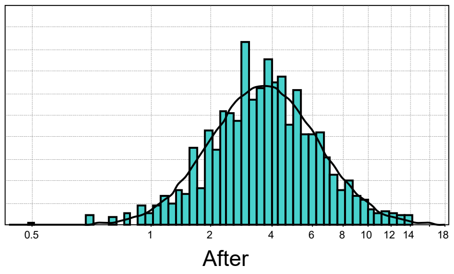

DBIL MF n=1,430
Para: 1.11 ~ 3.70 ~ 10.03
Nonpara: 1.08 ~ 3.70 ~ 10.98
Pow=0.308 TPos=0.497
Kurt=-0.18 Skew=0.034
K-S test for normality: .171 (NS)

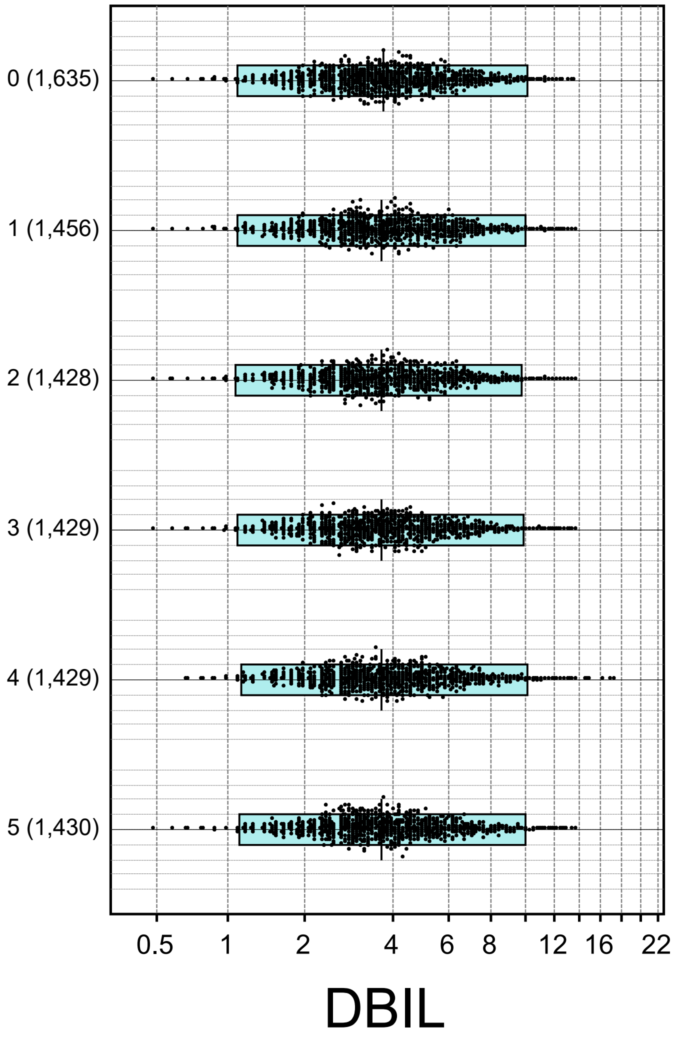

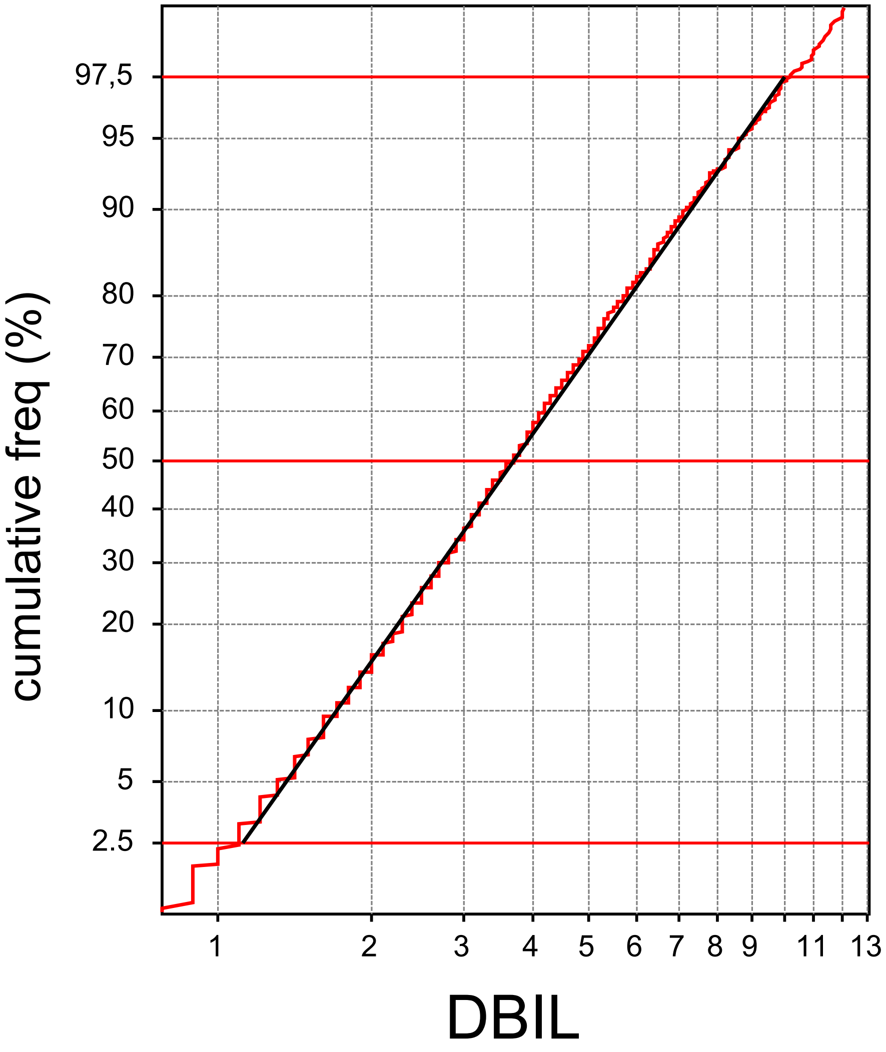

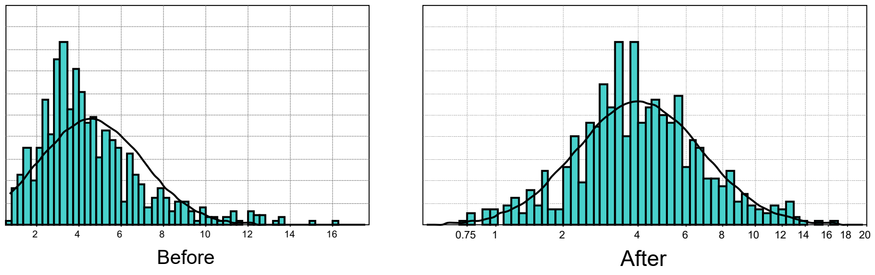

DBIL M n=672
Para: 1.25 ~ 4.06 ~ 10.40
Nonpara: 1.12 ~ 3.99 ~ 11.66
Pow=0.315 TPos=0.41
Kurt=-0.049 Skew=0.131
K-S test for normality: .332 (NS)

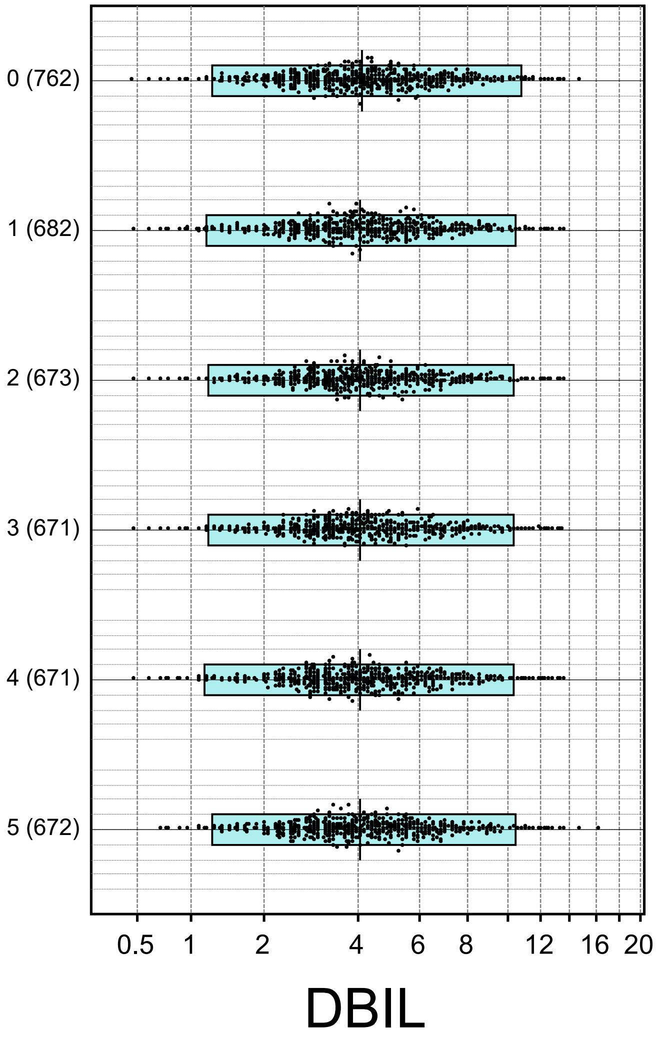

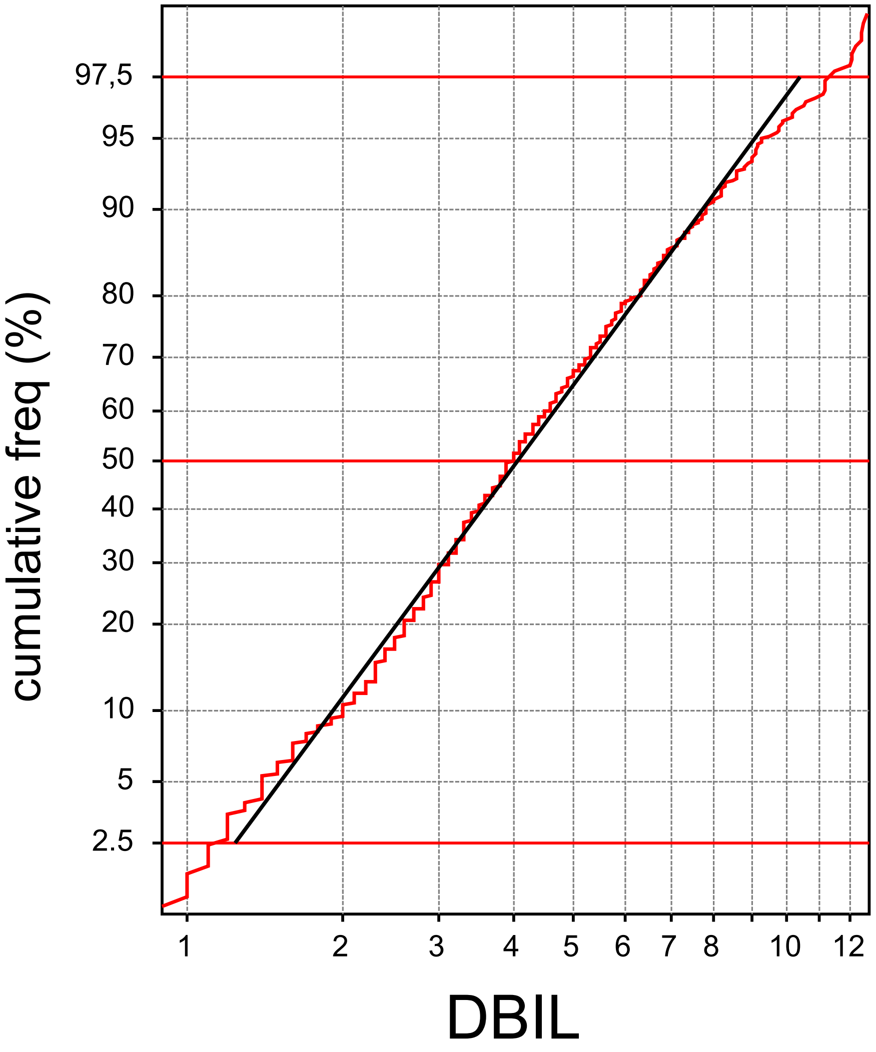

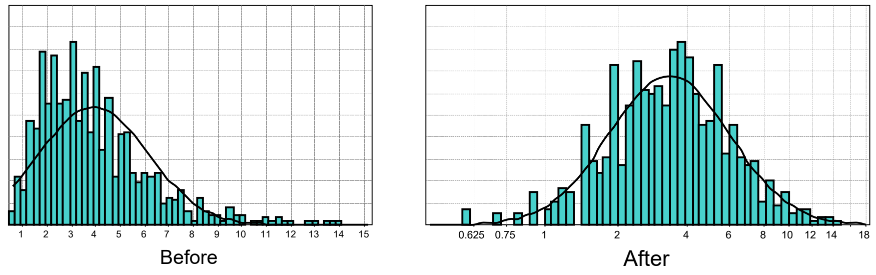

DBIL F n=768
Para: 1.09 ~ 3.38 ~ 9.66
Nonpara: 1.00 ~ 3.42 ~ 10.48
Pow=0.251 TPos=0.528
Kurt=-0.19 Skew=-0.007
K-S test for normality: .703 (NS)

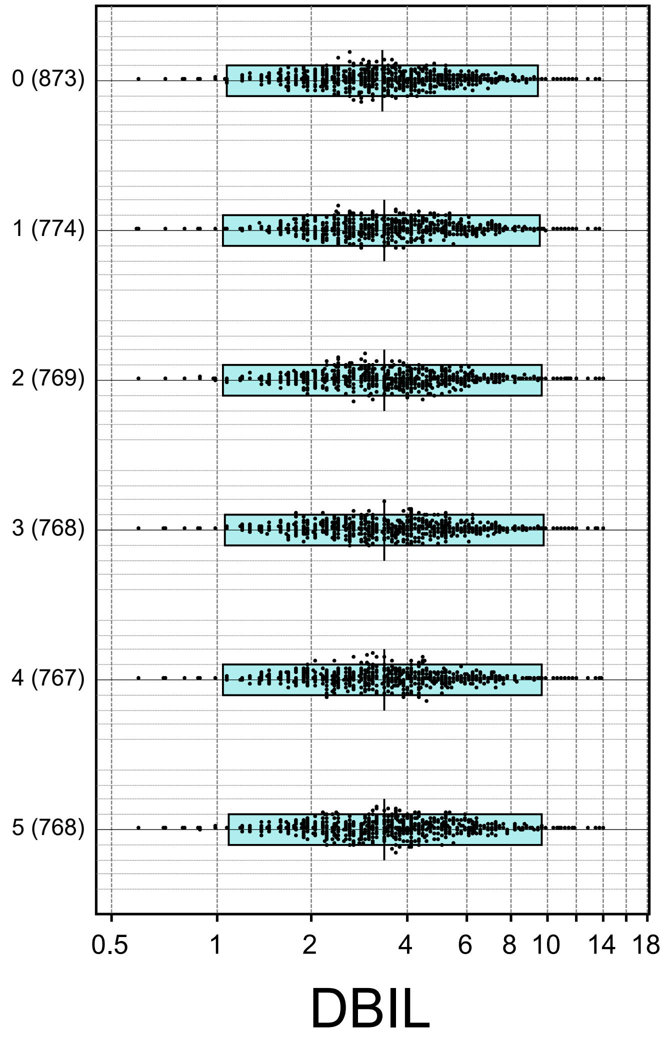

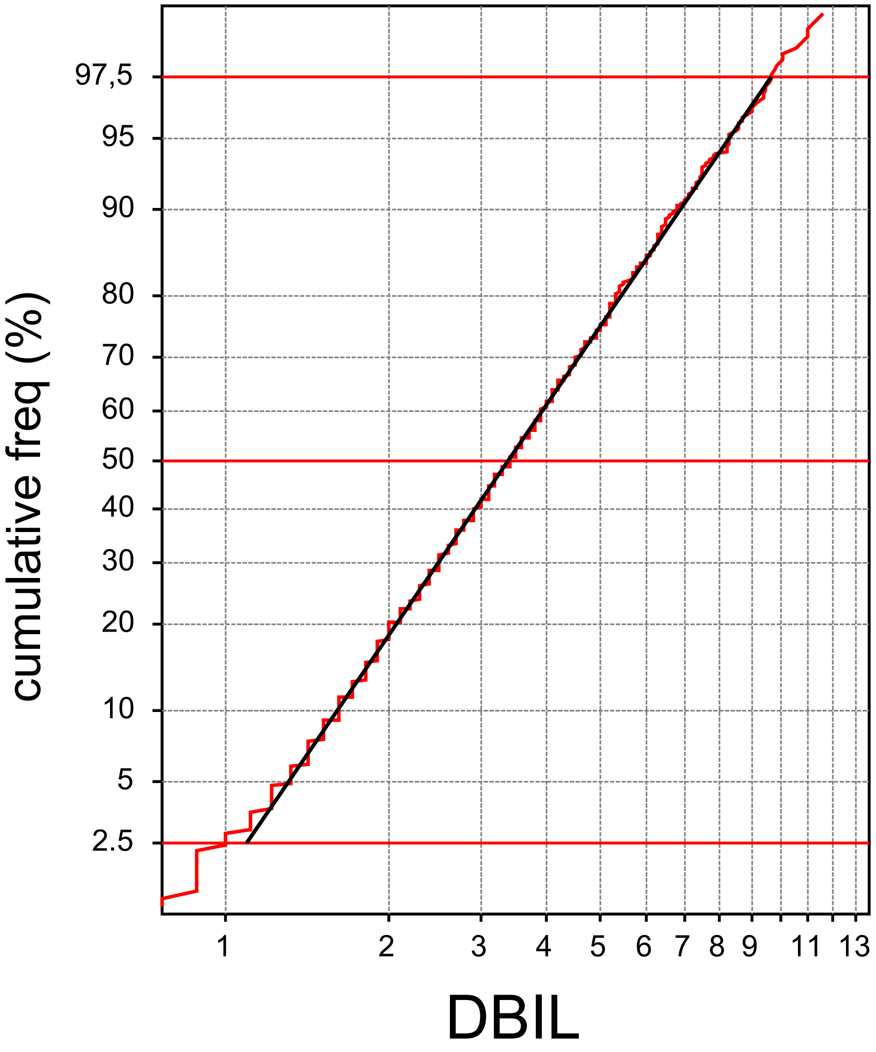

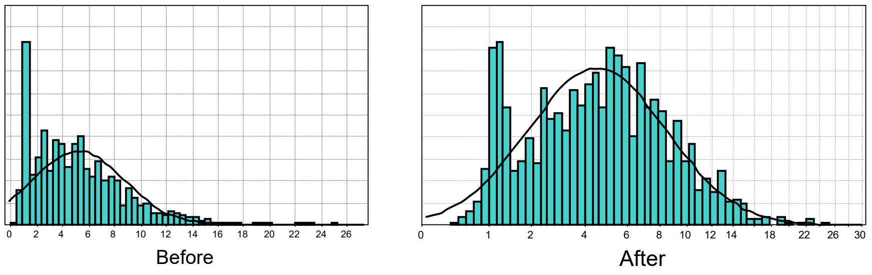

IBIL MF n=1,439
Para: 0.56 ~ 4.47 ~ 13.99
Nonpara: 0.96 ~ 4.64 ~ 13.99
Pow=0.329 TPos=-0.46
Kurt=-0.711 Skew=0.044
K-S test for normality: 0.00021

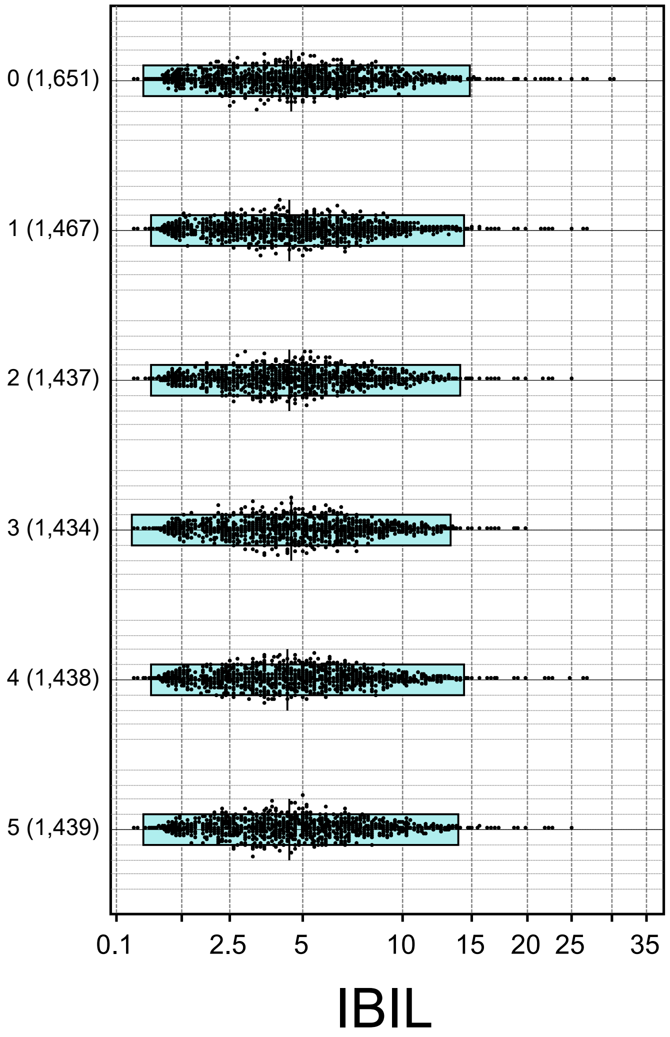

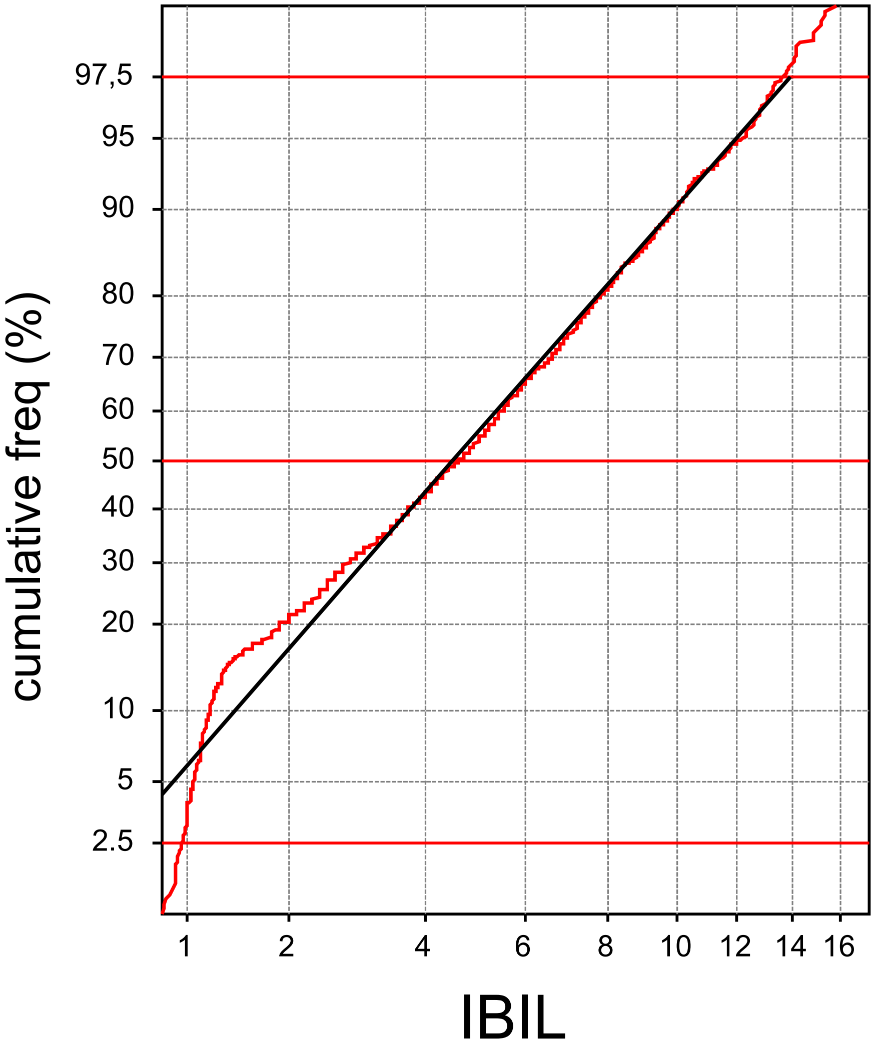

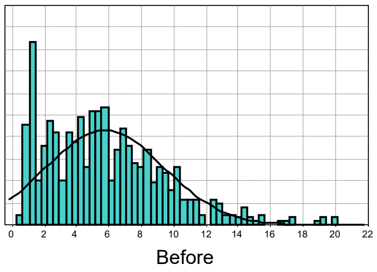

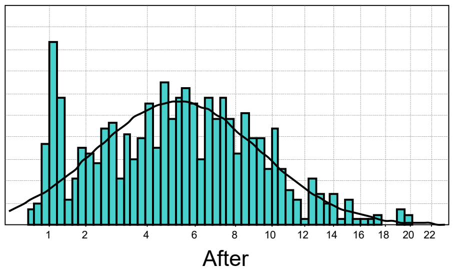

IBIL M n=669
Para: 0.33 ~ 5.21 ~ 14.46
Nonpara: 0.96 ~ 5.27 ~ 15.13
Pow=0.353 TPos=-2.028
Kurt=-0.61 Skew=0.118
K-S test for normality: .069 (NS)

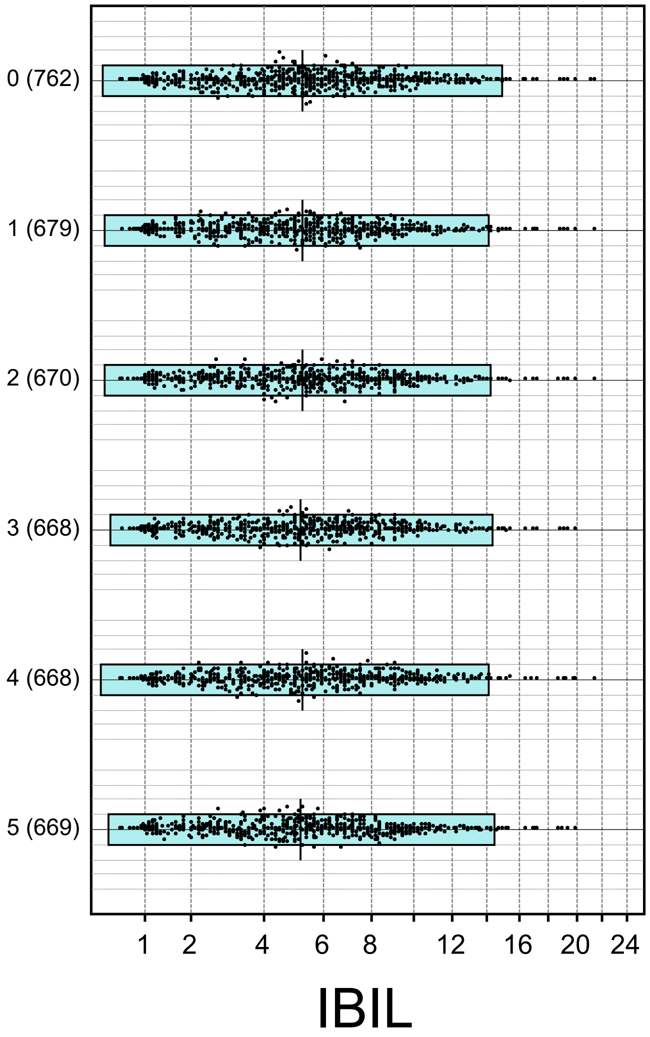

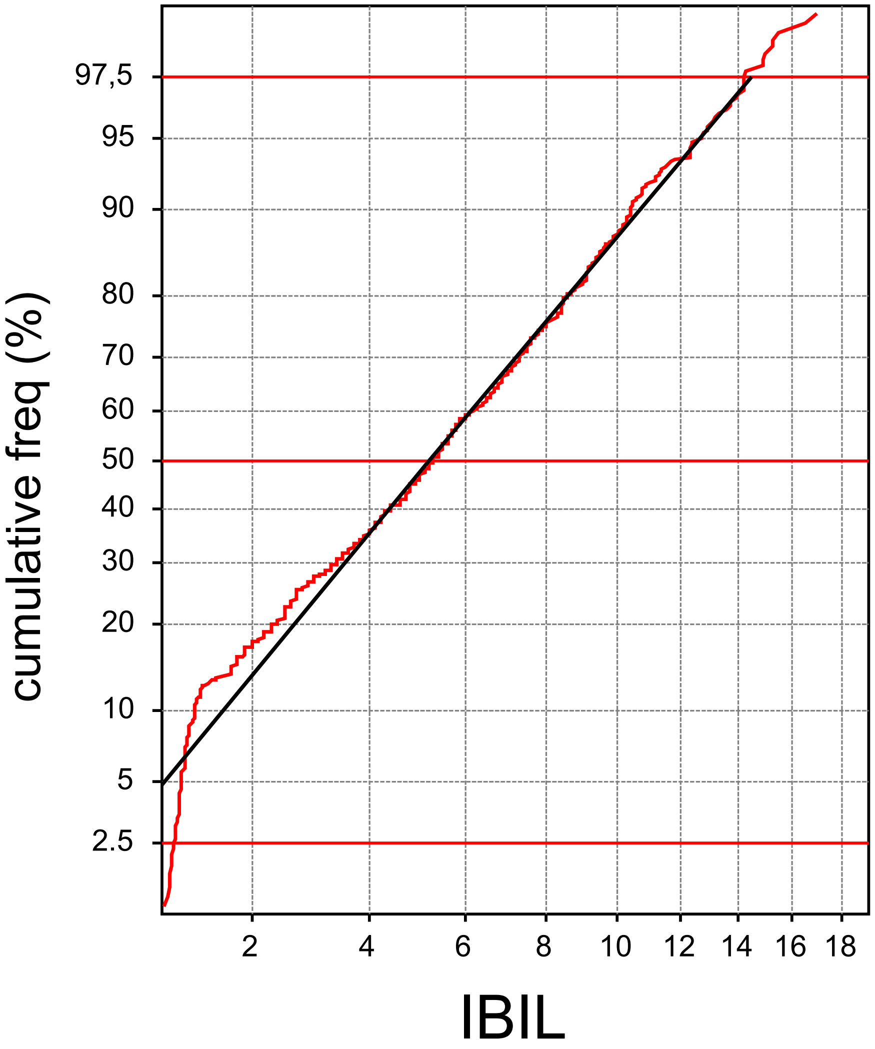

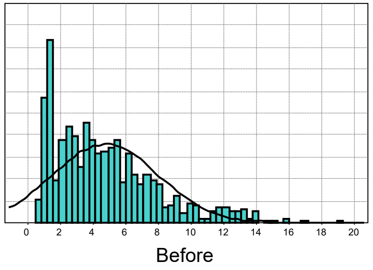

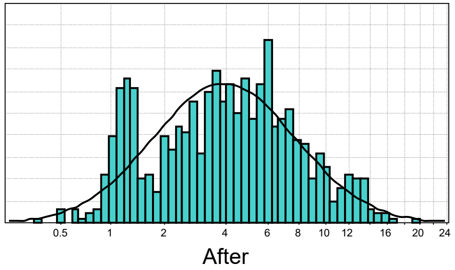

IBIL F n=773
Para: 0.70 ~ 3.92 ~ 14.26
Nonpara: 0.93 ~ 4.12 ~ 13.53
Pow=0.287 TPos=0.219
Kurt=-0.649 Skew=-0.056
K-S test for normality: 0.00234

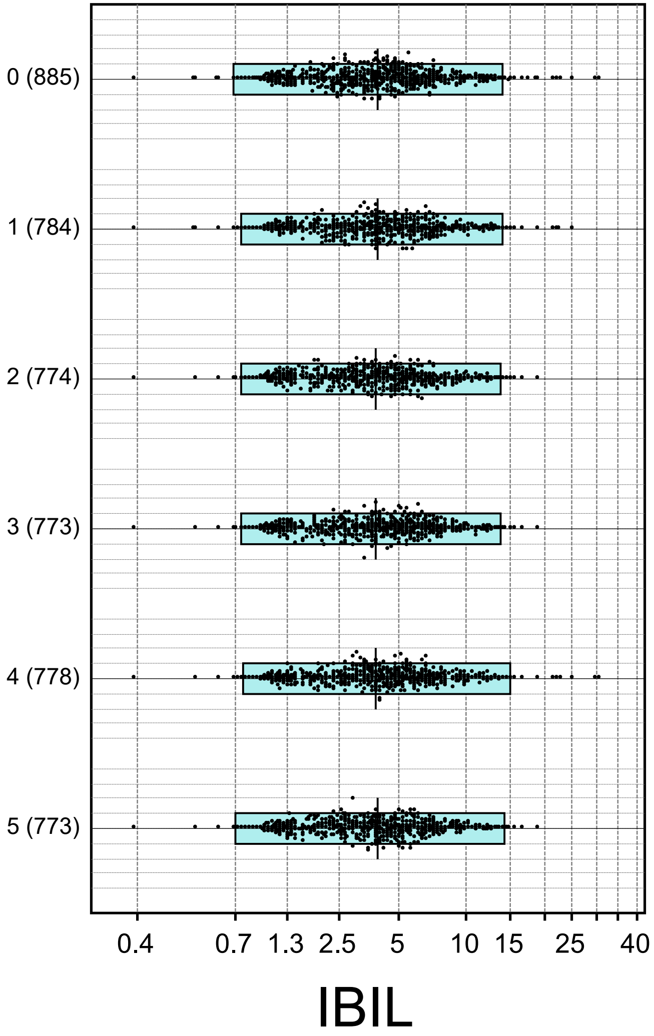

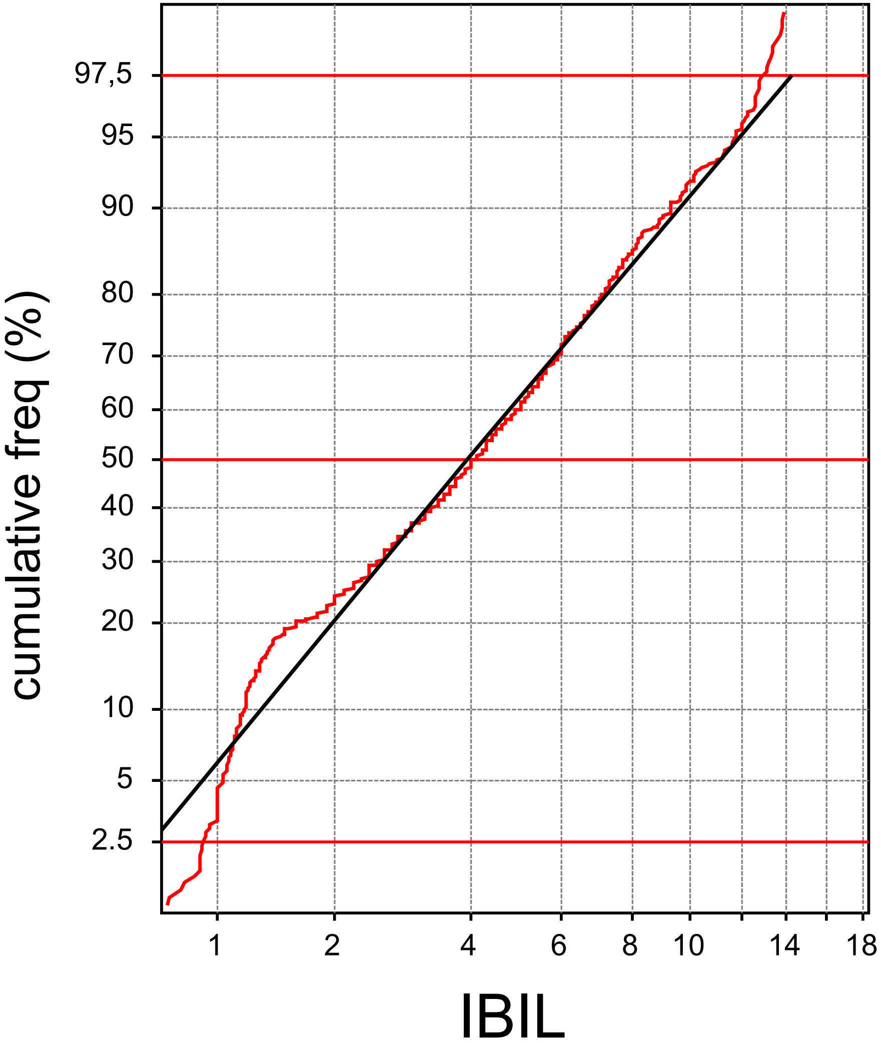

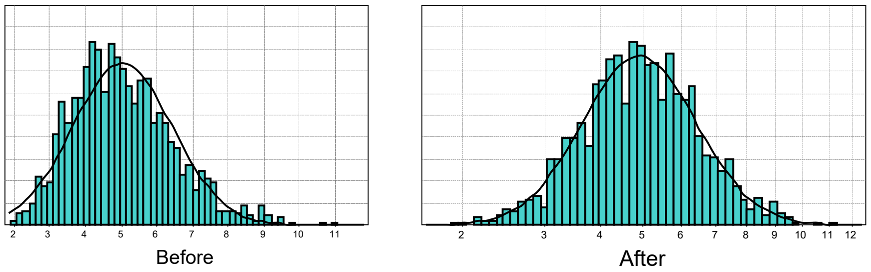

UREA MF n=1,443
Para: 2.73 ~ 4.88 ~ 8.13
Nonpara: 2.66 ~ 4.89 ~ 8.27
Pow=0.539 TPos=1.739
Kurt=-0.243 Skew=0.015
K-S test for normality: P≈1.00 (N.S.)

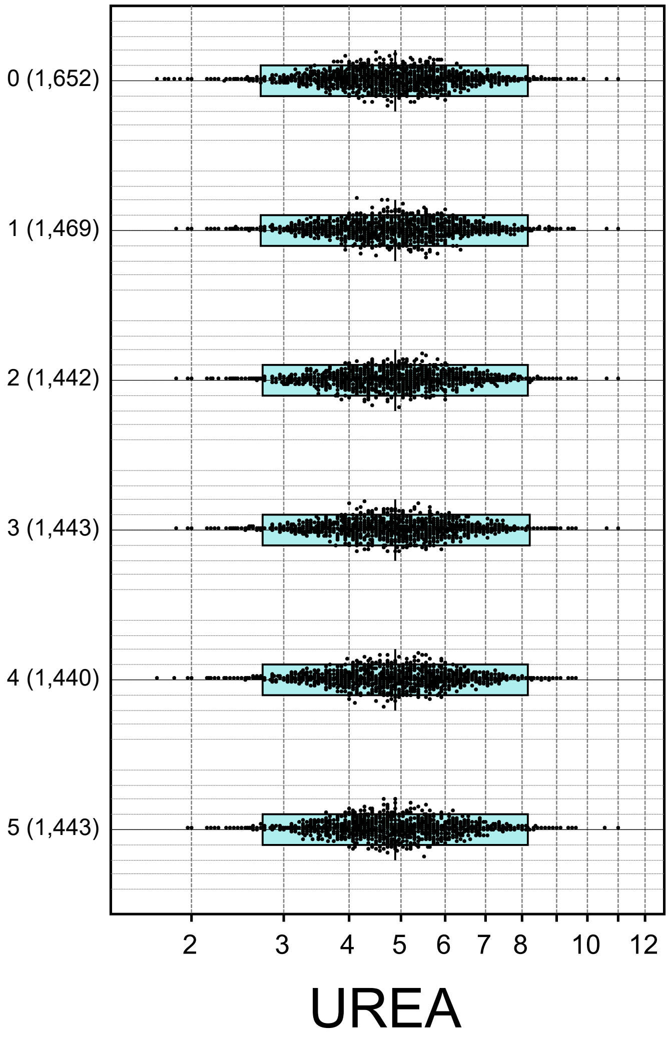

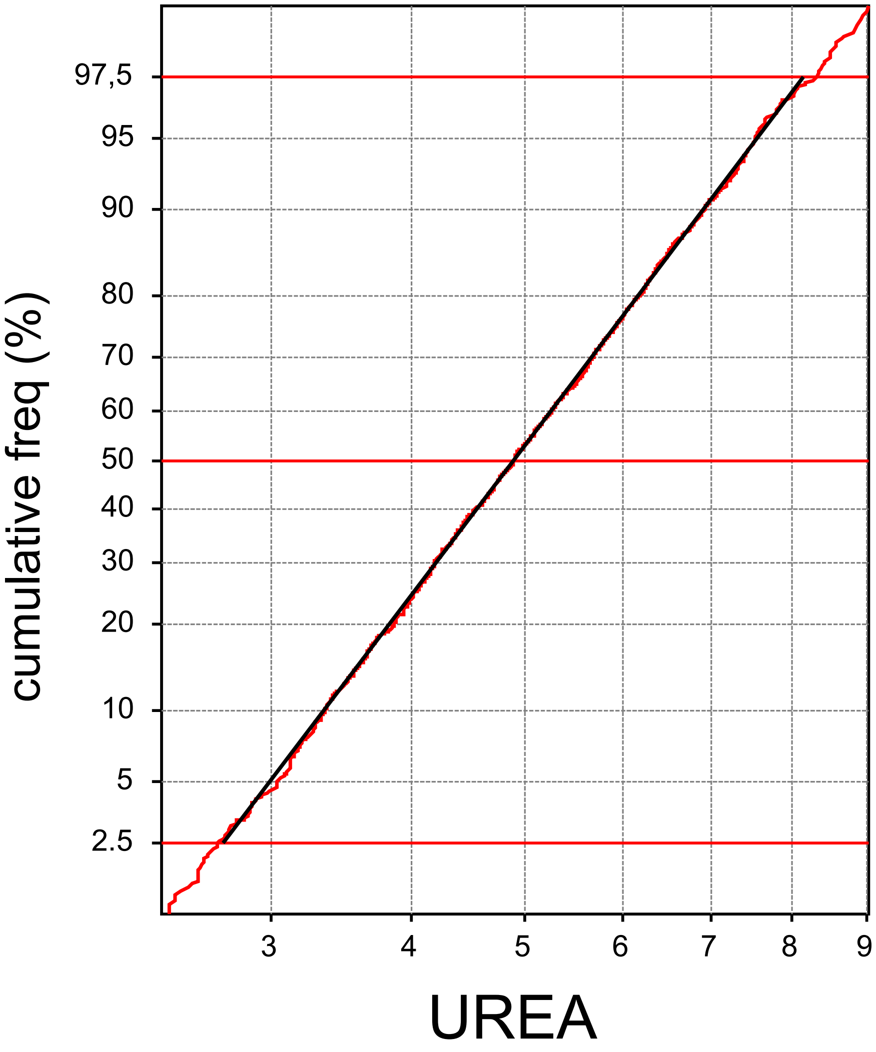

UREA M n=672
Para: 2.93 ~ 5.16 ~ 8.49
Nonpara: 3.03 ~ 5.19 ~ 8.69
Pow=0.519 TPos=1.718
Kurt=-0.279 Skew=-0.025
K-S test for normality: P≈1.00 (N.S.)

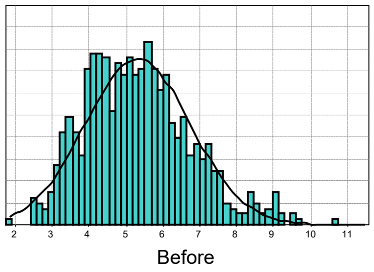

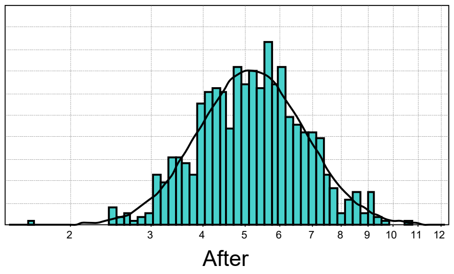

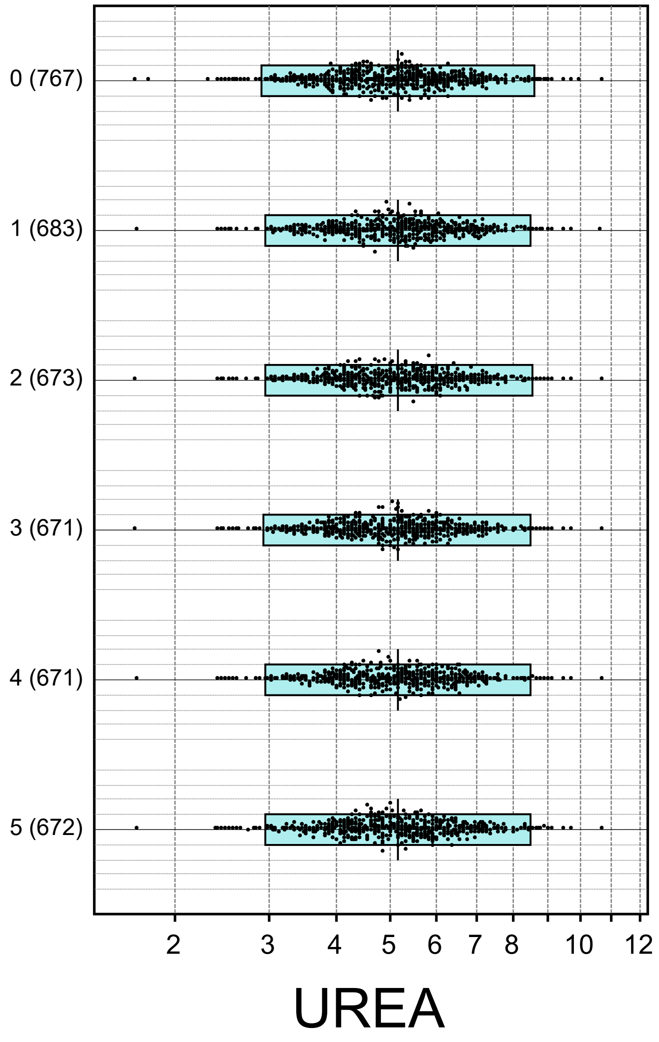

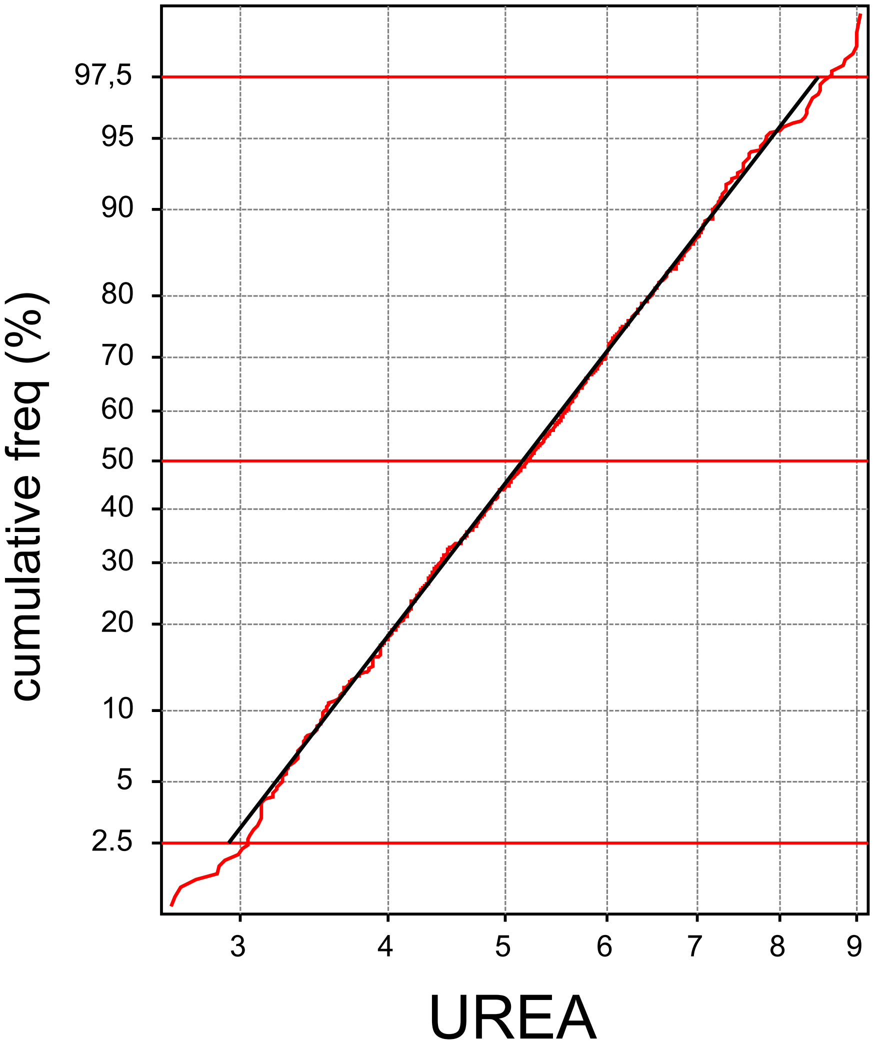

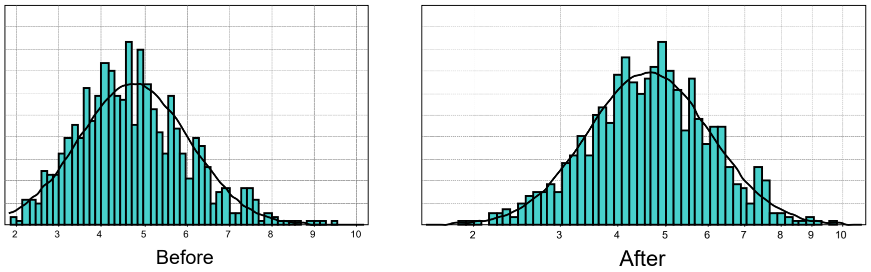

UREA F n=779
Para: 2.59 ~ 4.64 ~ 7.66
Nonpara: 2.55 ~ 4.67 ~ 7.63
Pow=0.574 TPos=1.731
Kurt=-0.231 Skew=-0.034
K-S test for normality: P≈1.00 (N.S.)

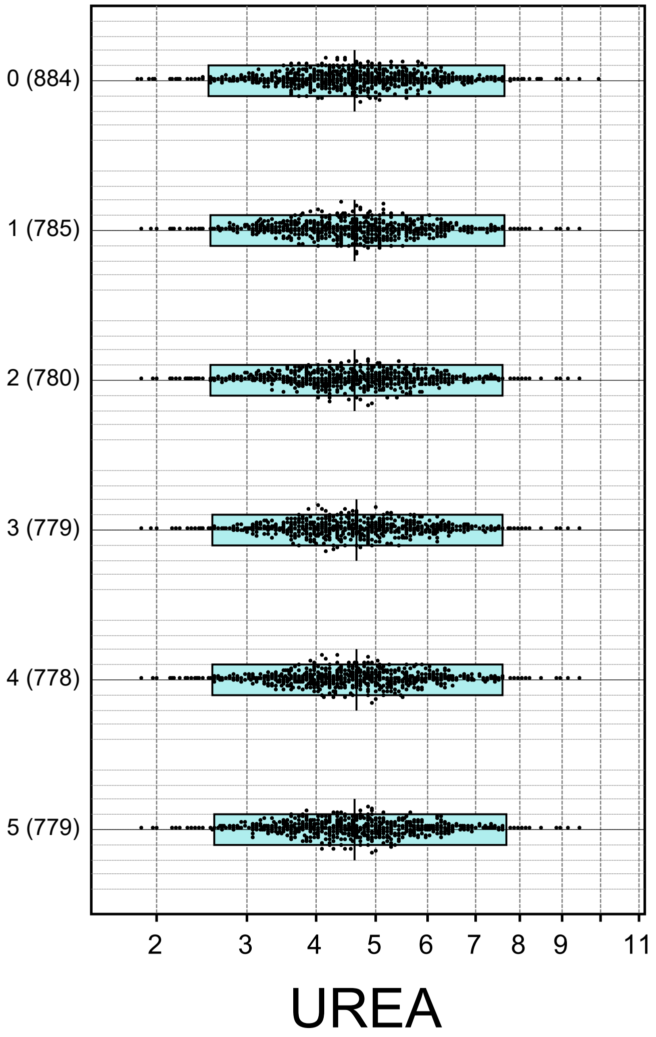

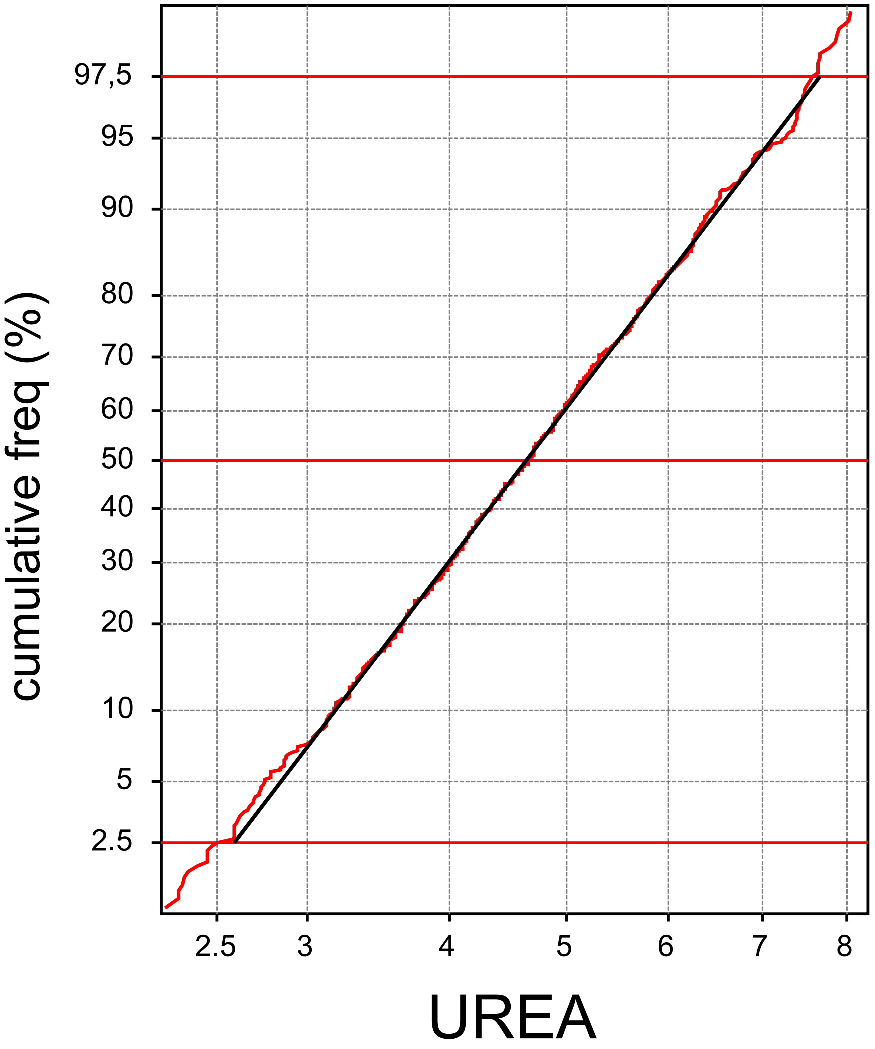

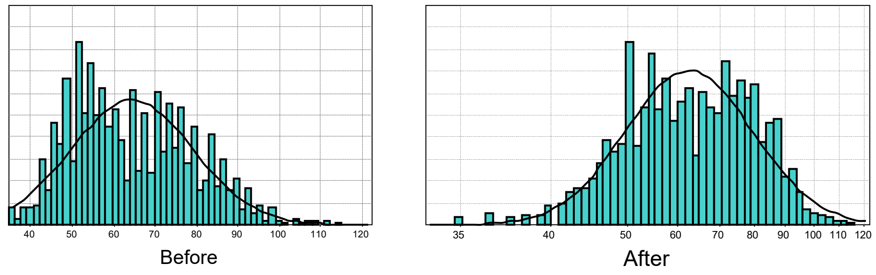

CREA MF n=1,445
Para: 41.4 ~ 62.3 ~ 100.0
Nonpara: 41.1 ~ 62.3 ~ 94.6
Pow=0.453 TPos=34.668
Kurt=-0.588 Skew=-0.129
K-S test for normality: 0.00003

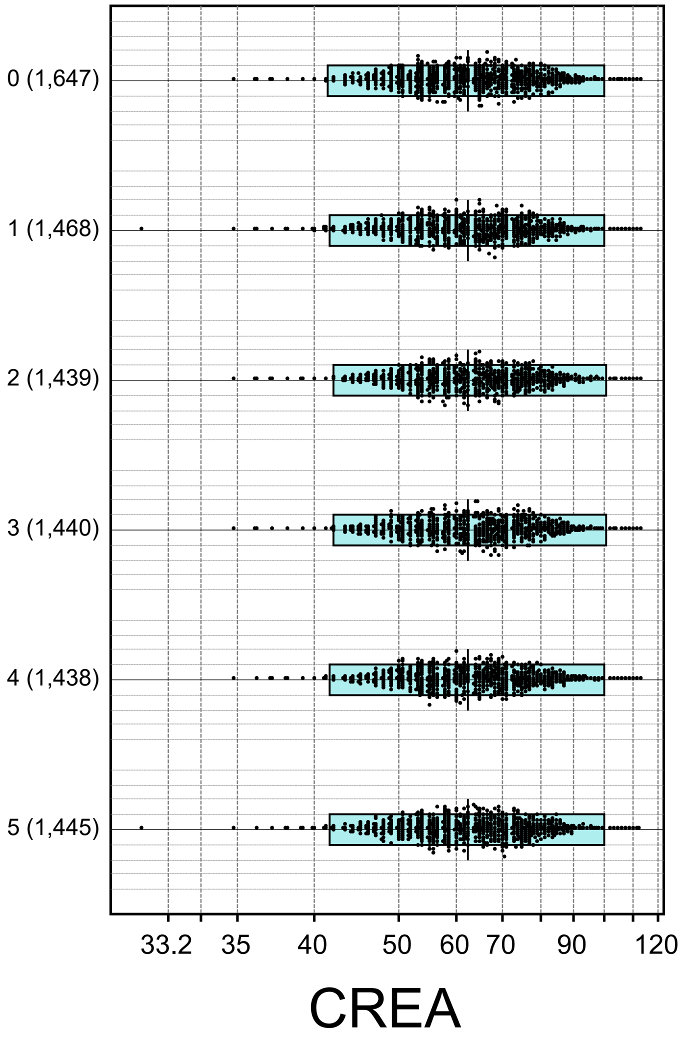

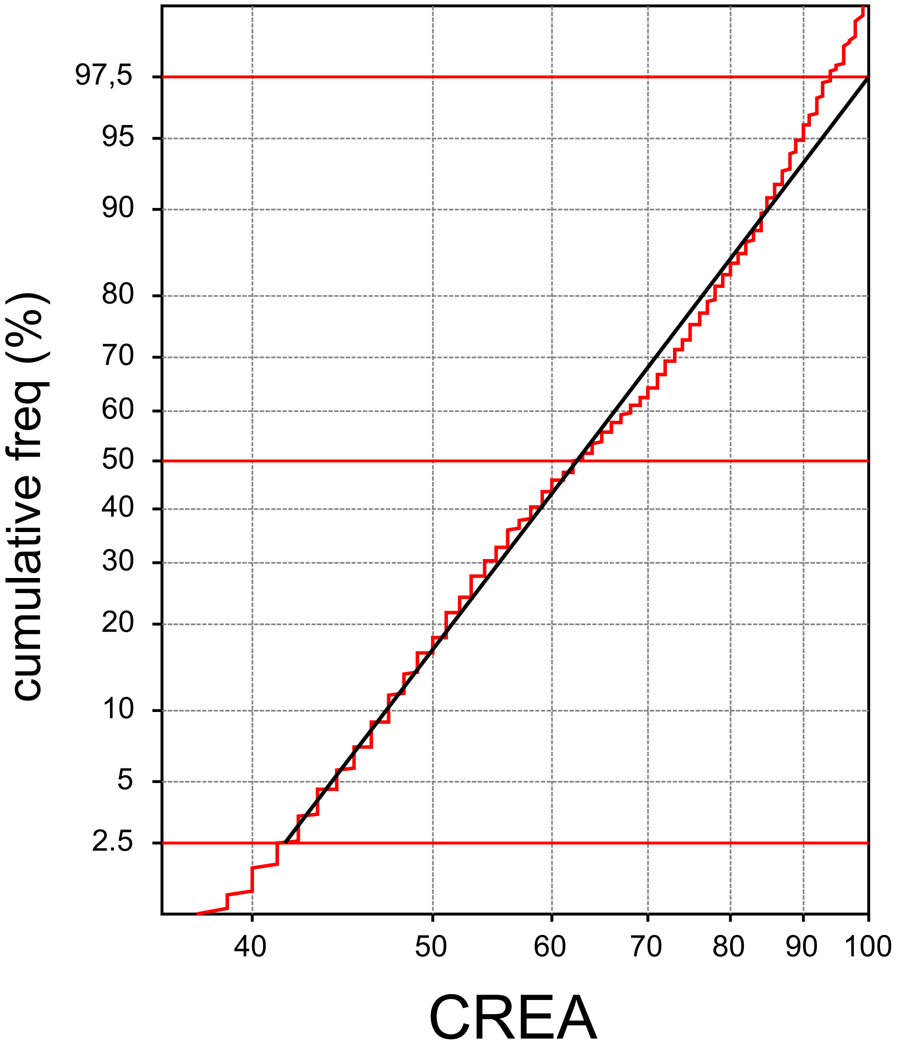

CREA M n=672
Para: 53.2 ~ 76.5 ~ 96.9
Nonpara: 51.8 ~ 76.0 ~ 98.4
Pow=1.217 TPos=39.221
Kurt=-0.102 Skew=0.14
K-S test for normality: .086 (NS)

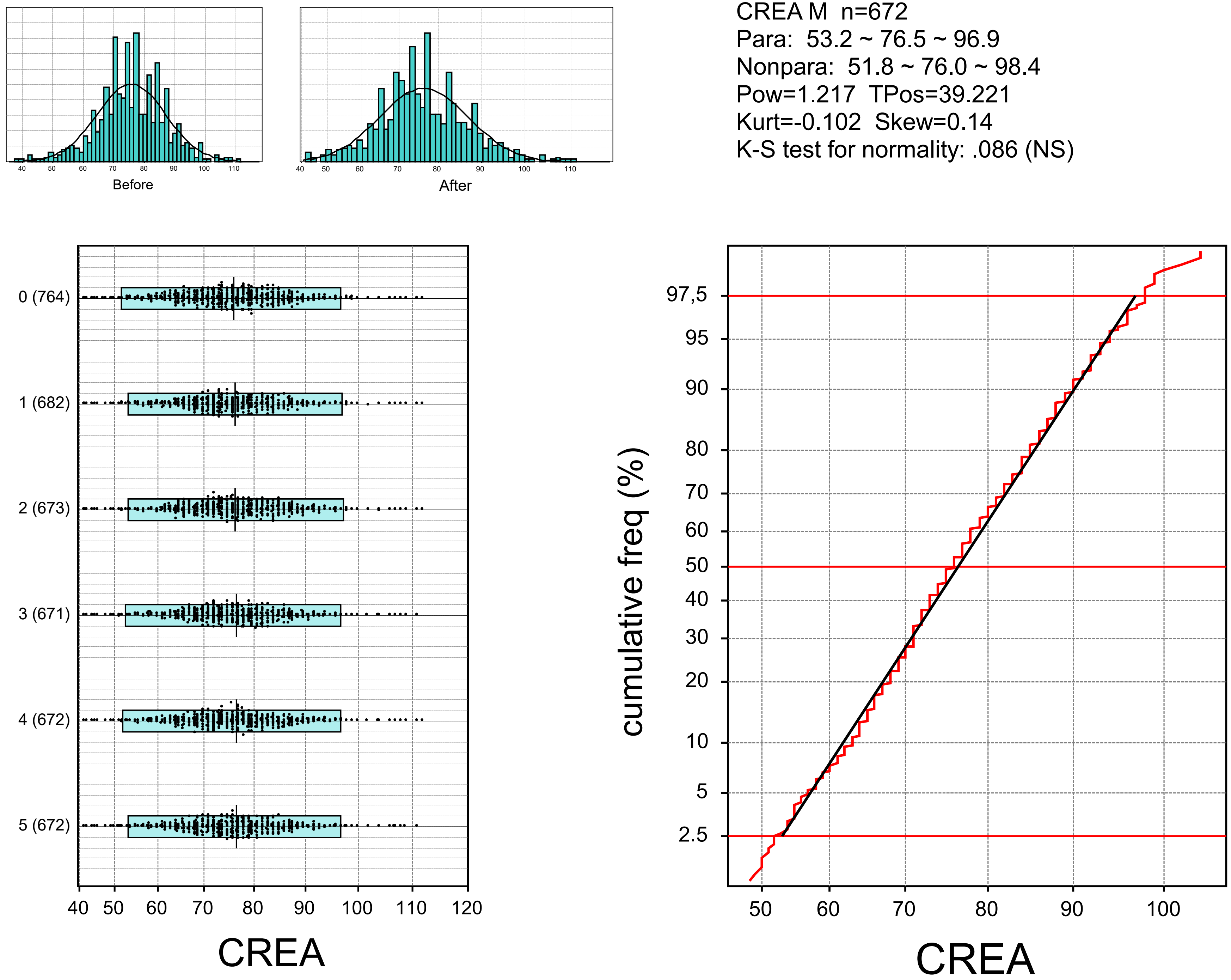

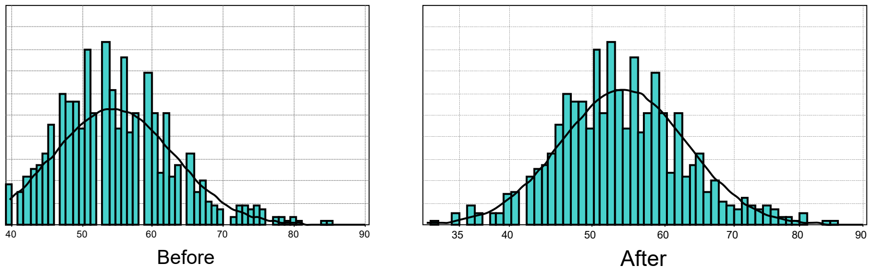

CREA F n=775
Para: 39.18 ~ 54.19 ~ 71.79
Nonpara: 39.13 ~ 53.90 ~ 75.07
Pow=0.809 TPos=32.328
Kurt=-0.108 Skew=0.097
K-S test for normality: .118 (NS)

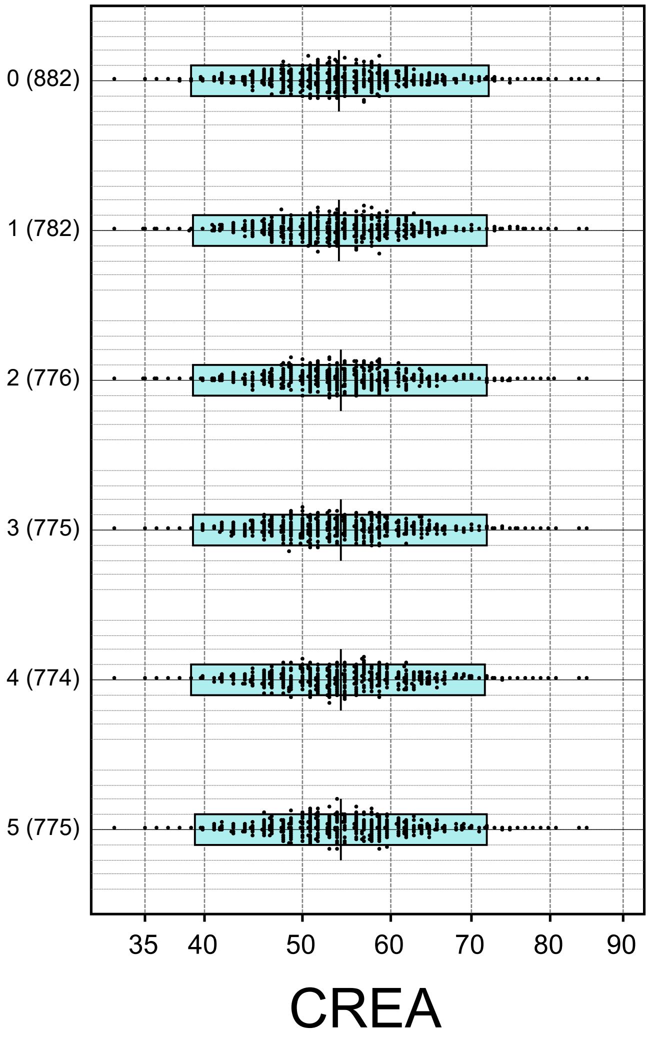

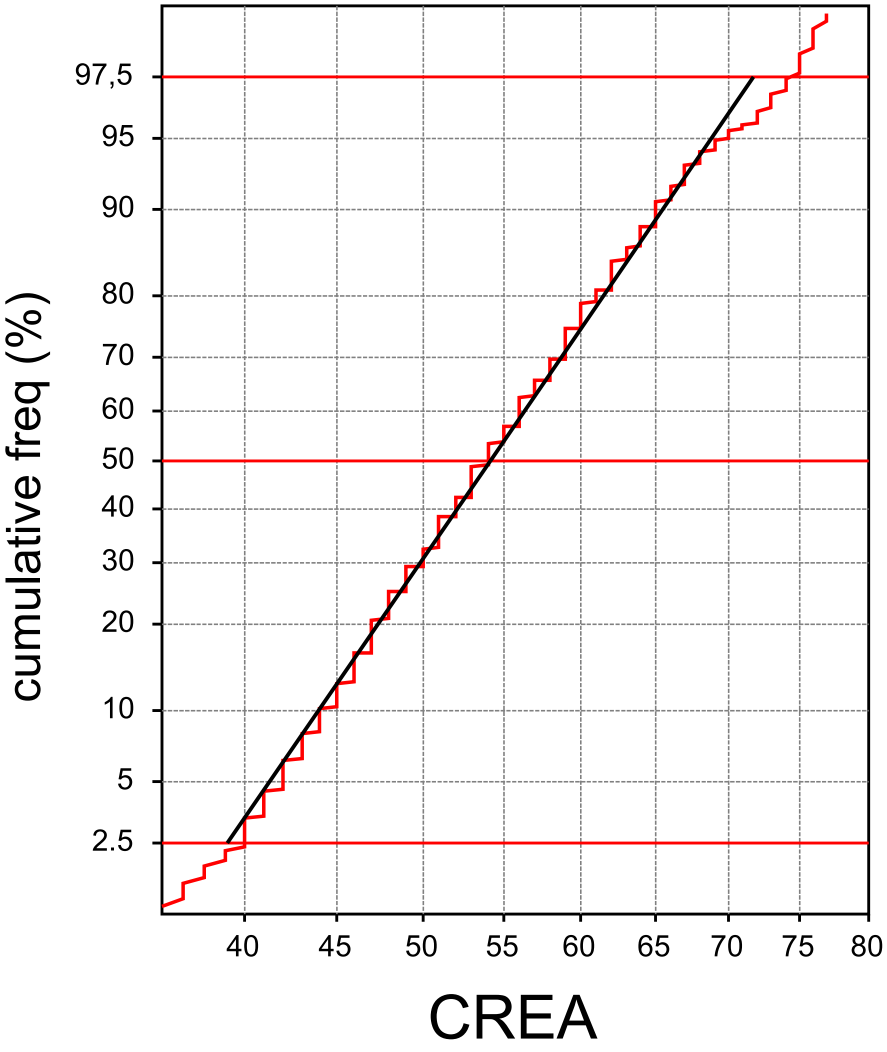

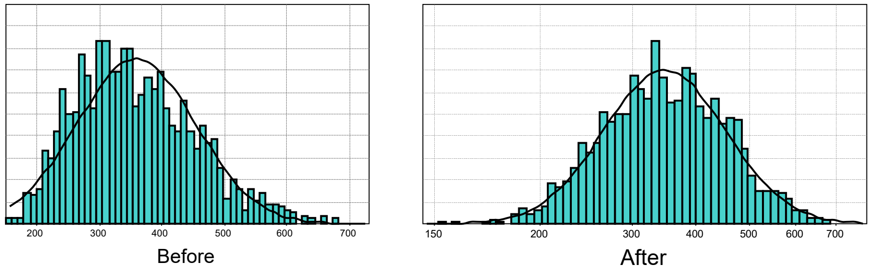

UA MF n=1,445
Para: 204.5 ~ 347.9 ~ 575.1
Nonpara: 204.0 ~ 347.0 ~ 569.7
Pow=0.523 TPos=149.238
Kurt=-0.398 Skew=-0.014
K-S test for normality: .768 (NS)

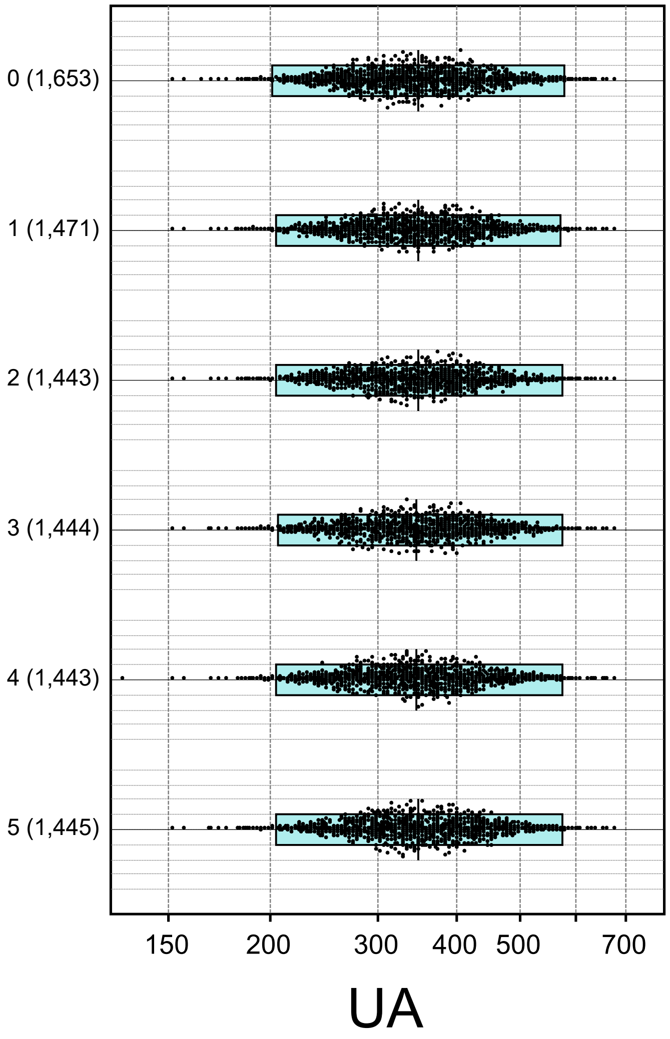

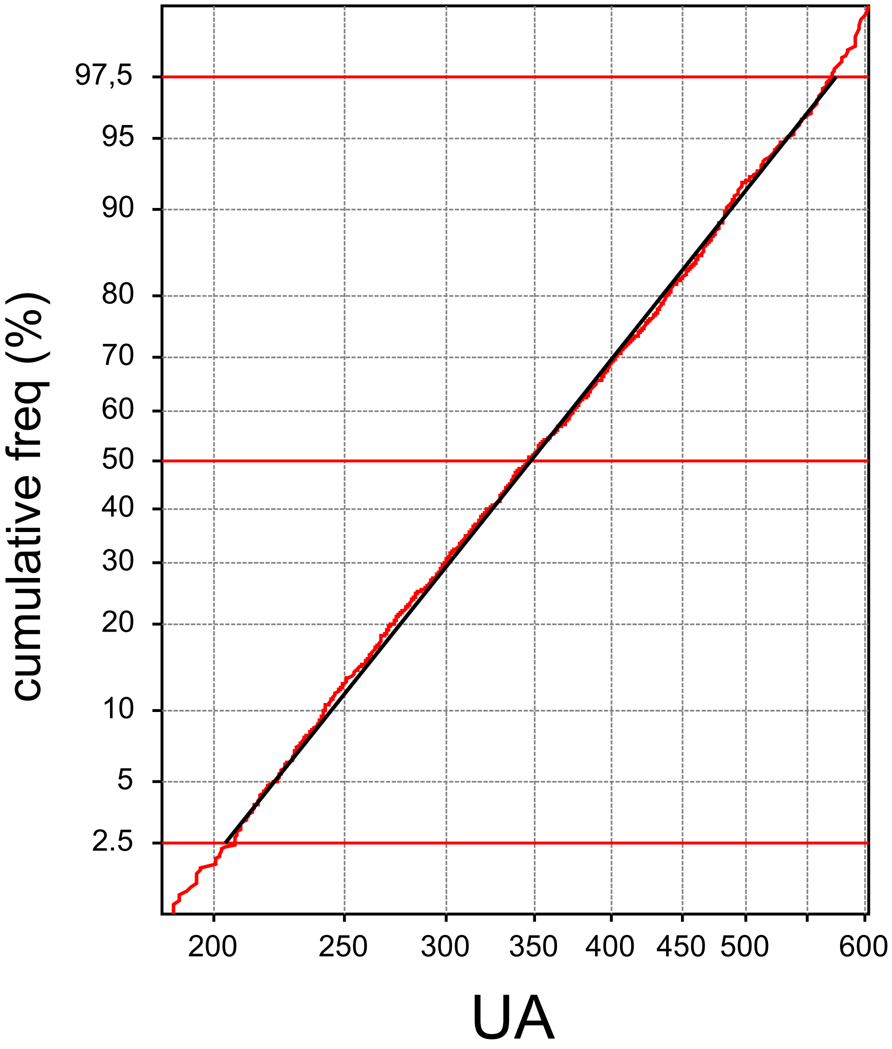

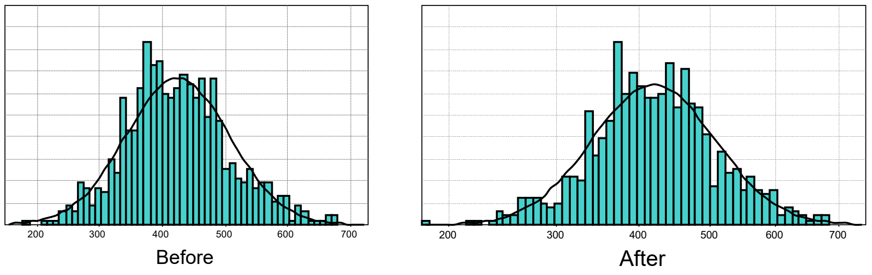

UA M n=673
Para: 275.5 ~ 421.5 ~ 598.2
Nonpara: 265.6 ~ 419.7 ~ 603.6
Pow=0.741 TPos=186.711
Kurt=-0.157 Skew=0.065
K-S test for normality: .952 (NS)

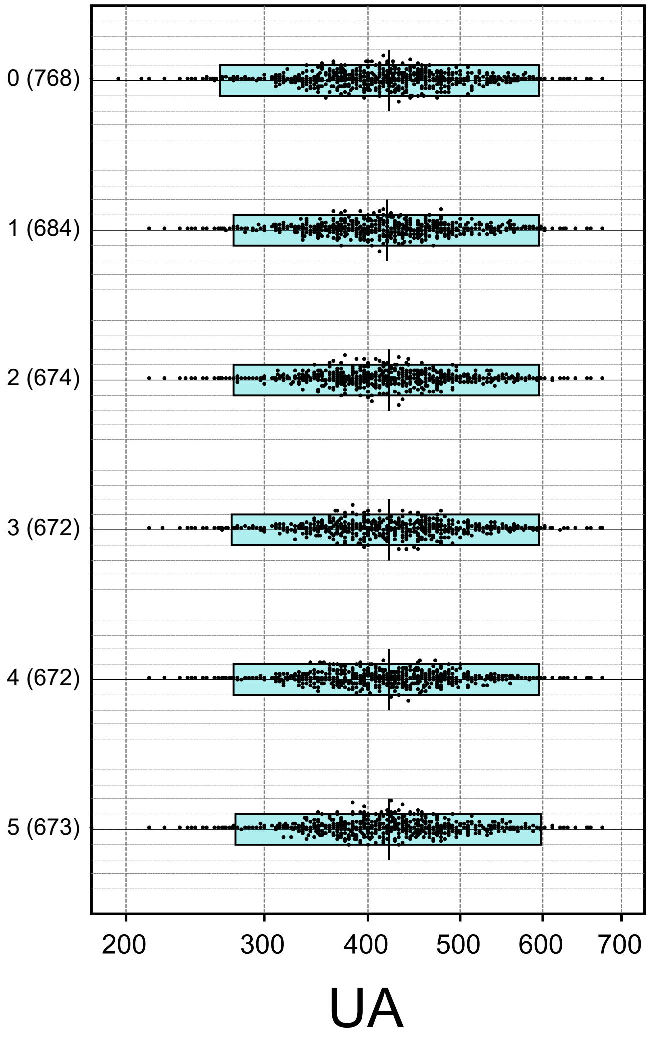

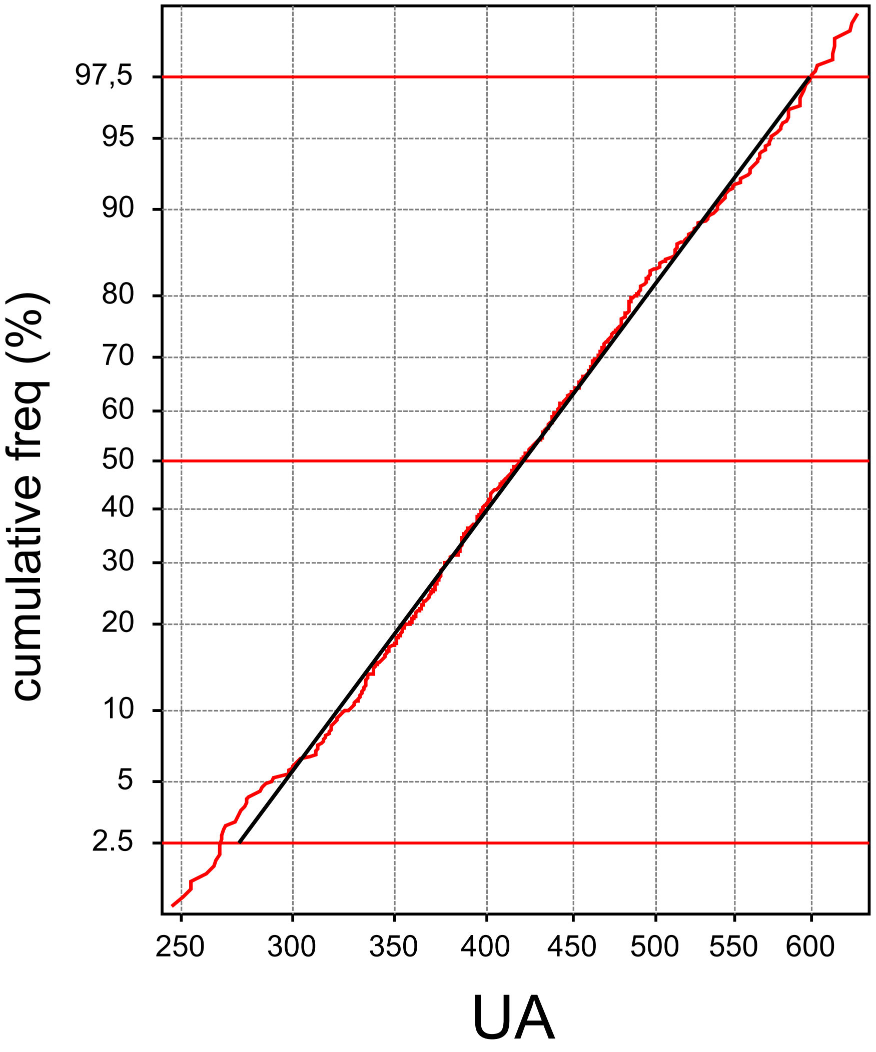

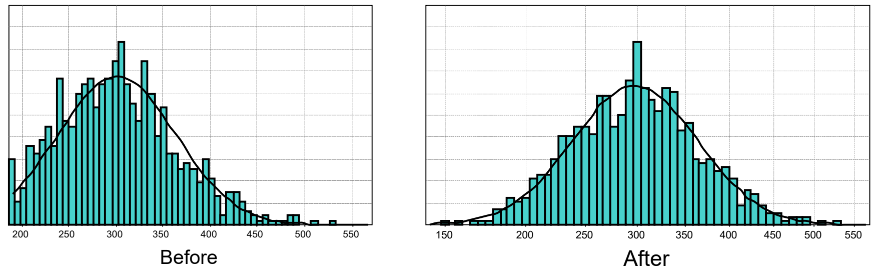

UA F n=775
Para: 190.4 ~ 297.7 ~ 431.9
Nonpara: 191.0 ~ 298.3 ~ 441.6
Pow=0.739 TPos=143.591
Kurt=-0.35 Skew=0.013
K-S test for normality: P≈1.00 (N.S.)

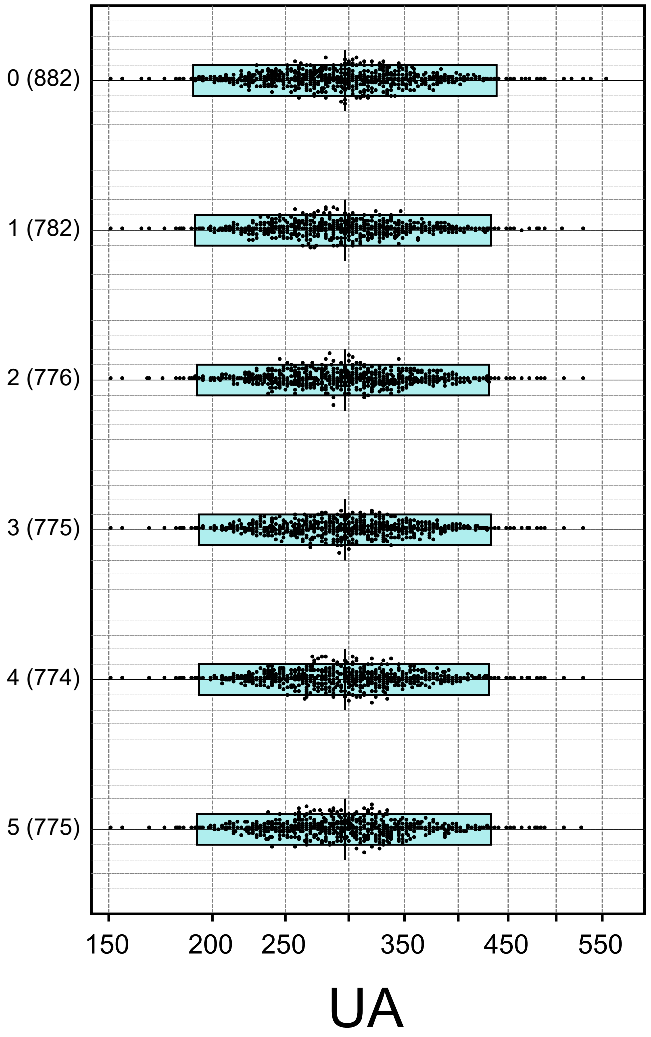

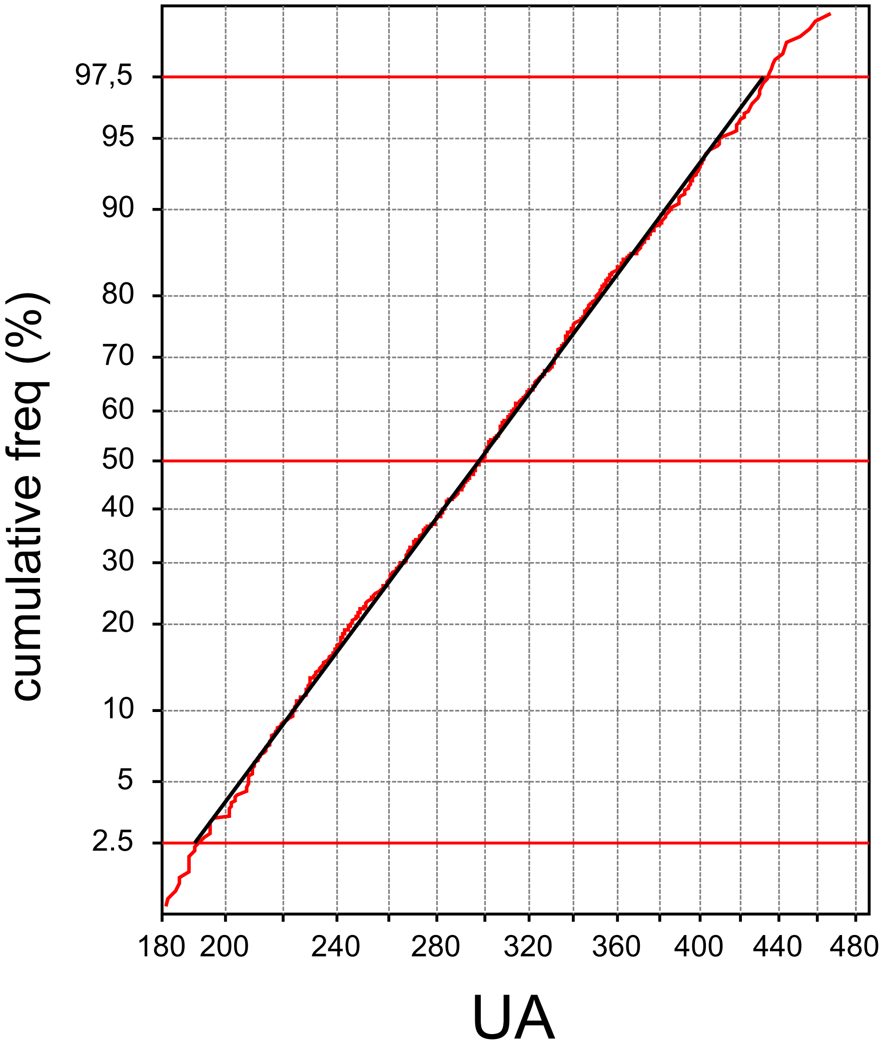

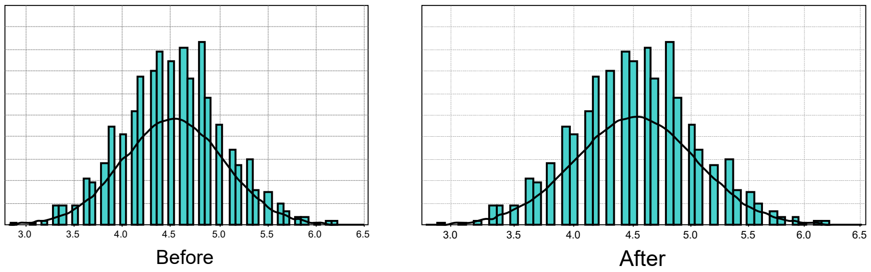

GLU MF n=1,441
Para: 3.52 ~ 4.52 ~ 5.56
Nonpara: 3.51 ~ 4.50 ~ 5.61
Pow=0.939 TPos=2.789
Kurt=-0.3 Skew=0.016
K-S test for normality: 0.00703

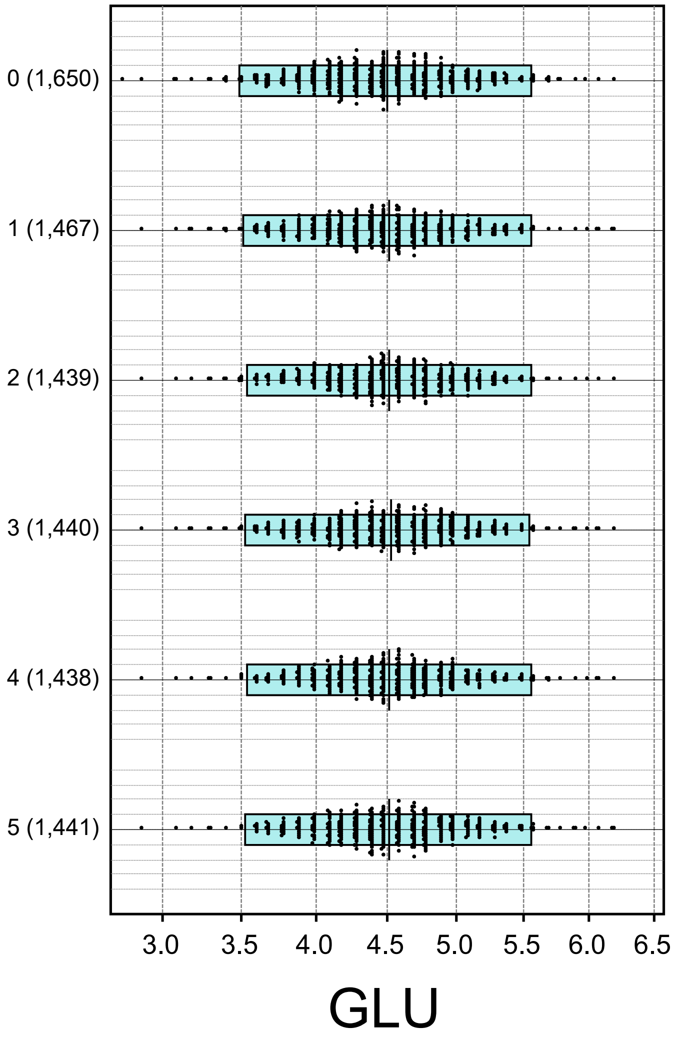

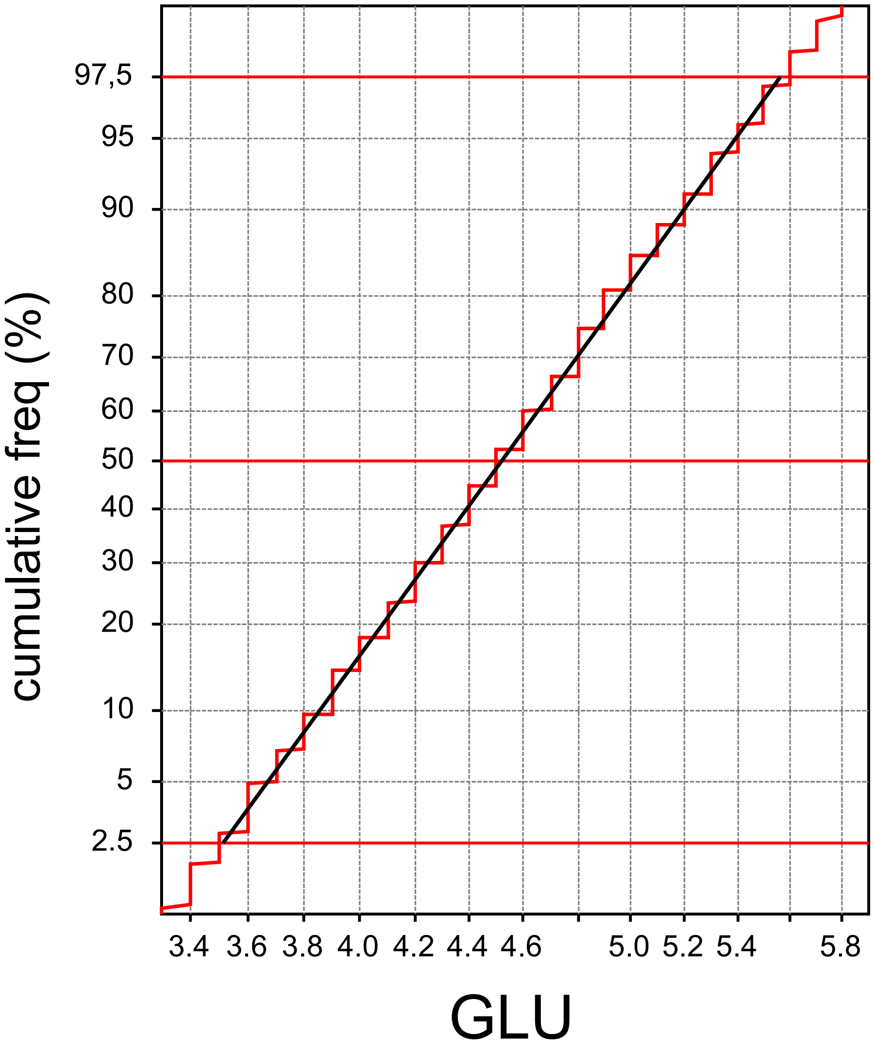

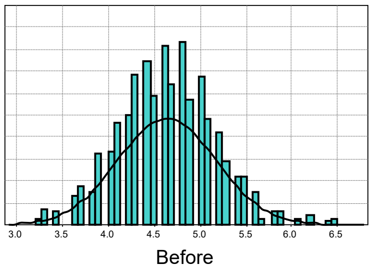

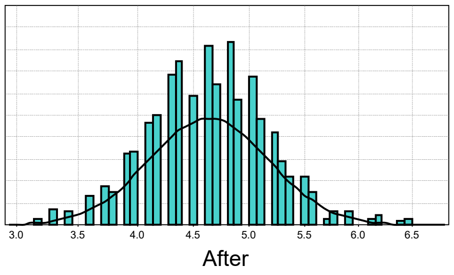

GLU M n=673
Para: 3.61 ~ 4.63 ~ 5.69
Nonpara: 3.61 ~ 4.61 ~ 5.73
Pow=0.936 TPos=2.885
Kurt=-0.19 Skew=0.083
K-S test for normality: .165 (NS)

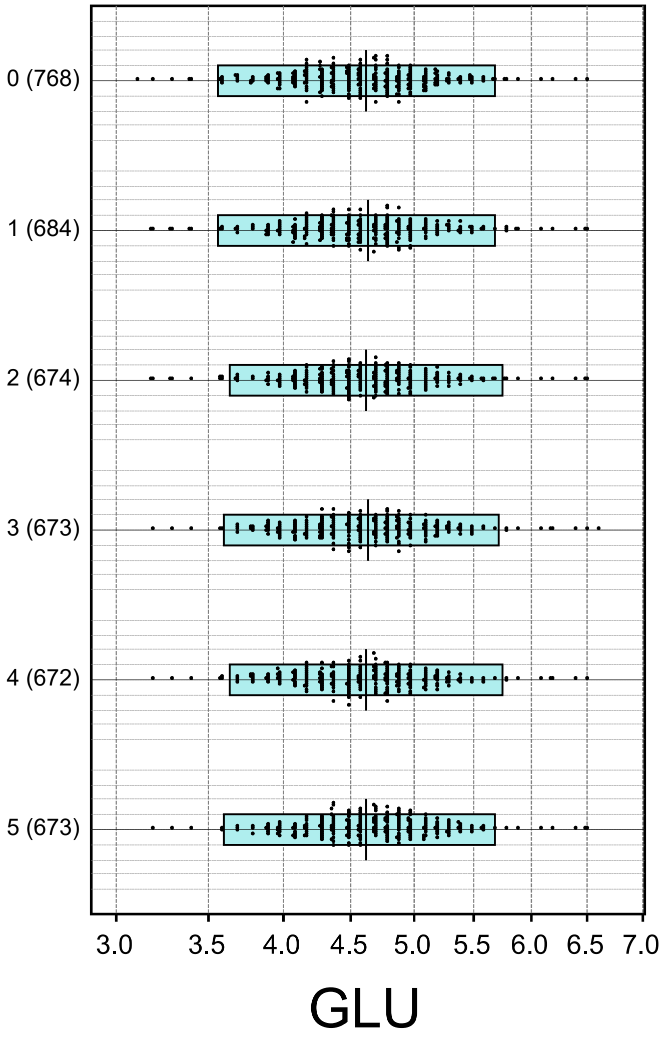

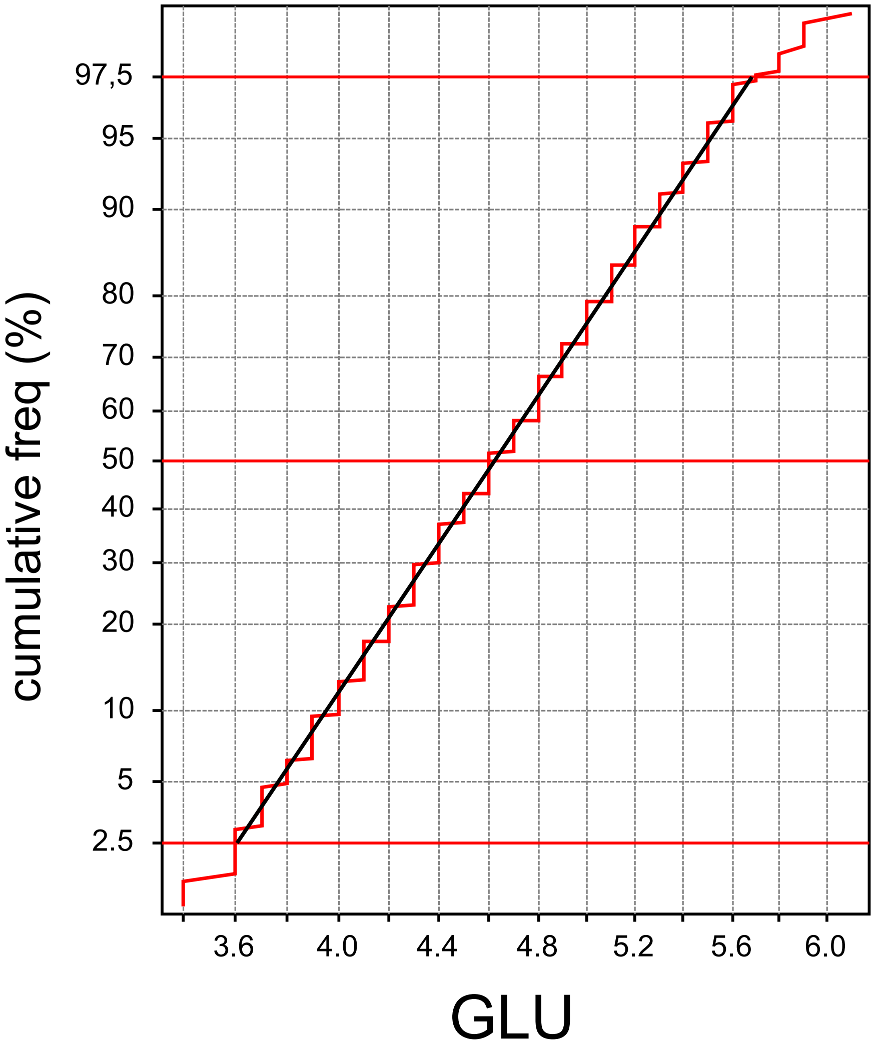

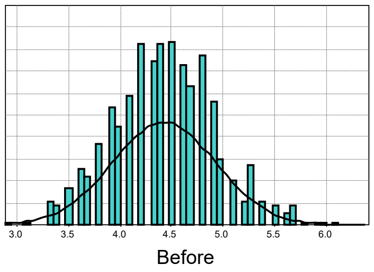

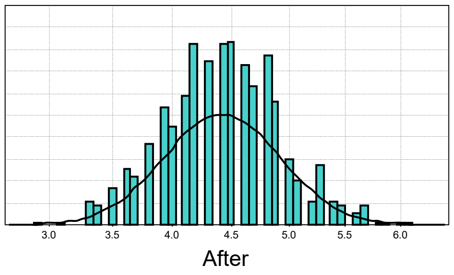

GLU F n=778
Para: 3.46 ~ 4.42 ~ 5.44
Nonpara: 3.45 ~ 4.42 ~ 5.51
Pow=0.92 TPos=2.69
Kurt=-0.164 Skew=0.043
K-S test for normality: .064 (NS)

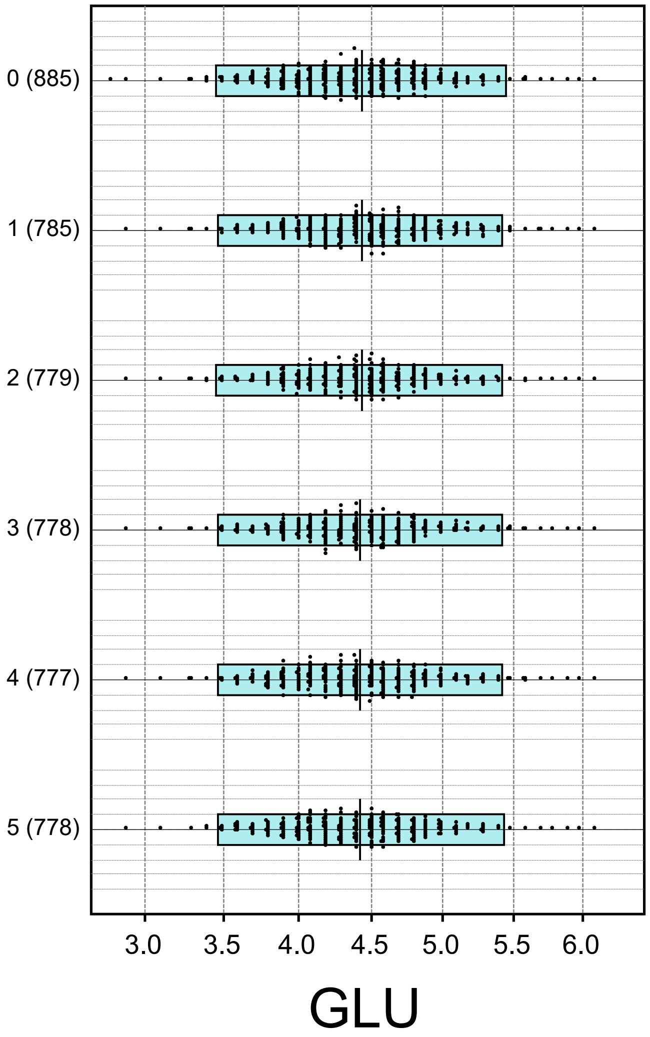

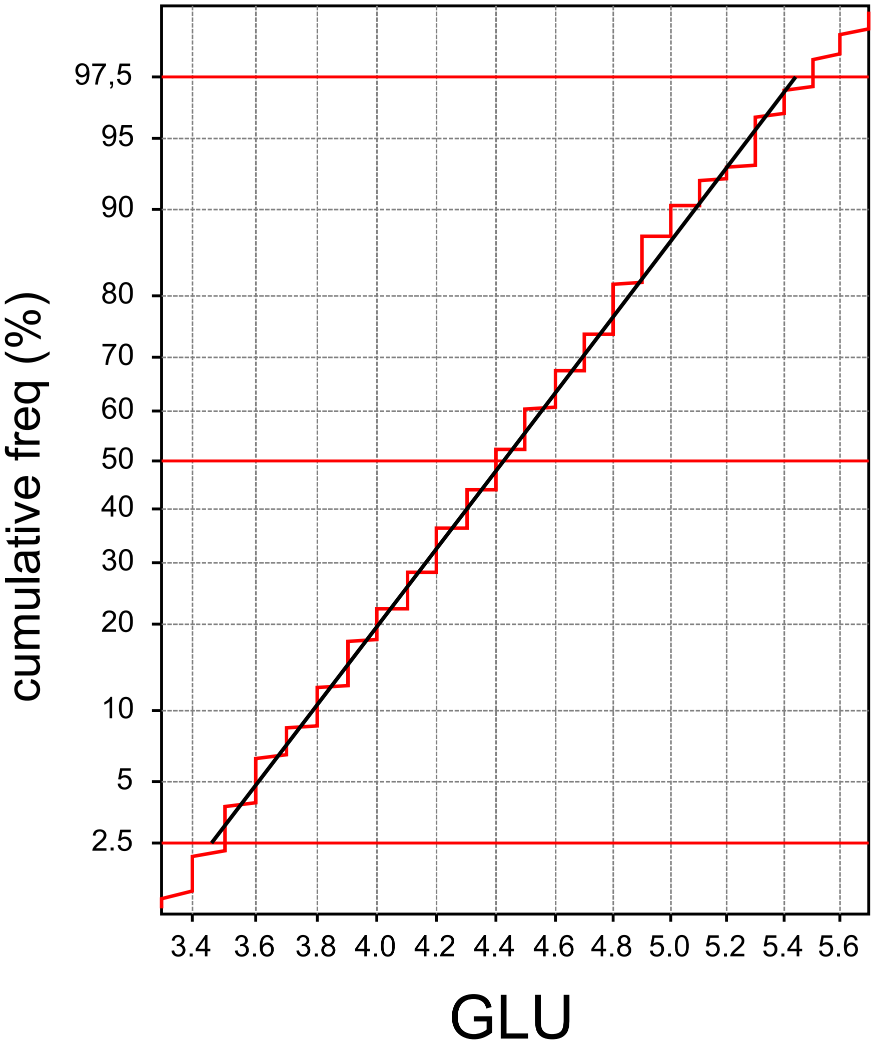

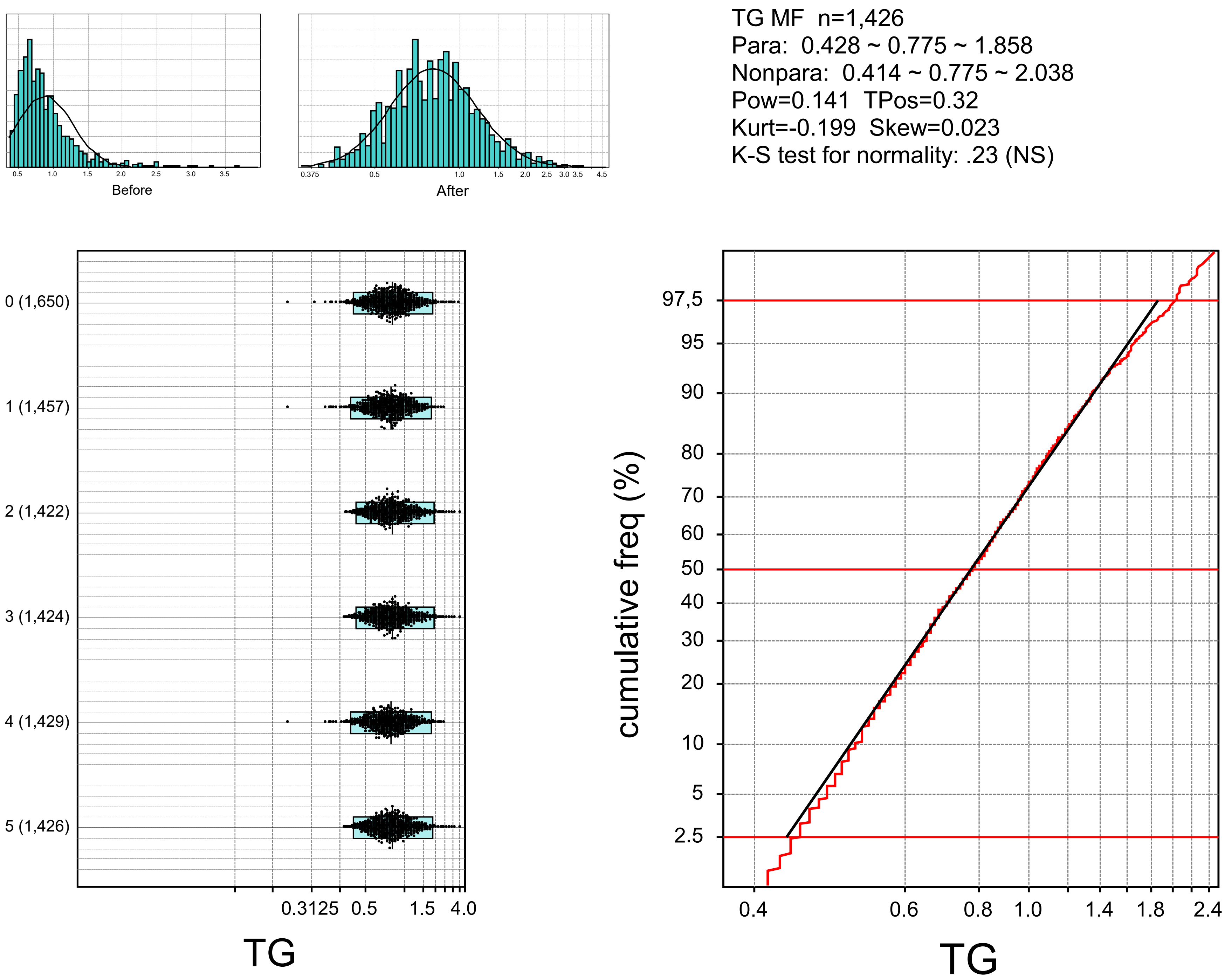
TG MF n=1,426
Para: 0.428 ~ 0.775 ~ 1.858
Nonpara: 0.414 ~ 0.775 ~ 2.038
Pow=0.141 TPos=0.32
Kurt=-0.199 Skew=0.023
K-S test for normality: .23 (NS)
Before
After
0 (1,650)
1 (1,457)
2 (1,422)
3 (1,424)
4 (1,429)
5 (1,426)
0.3125
0.5
1.5
4.0
TG
cumulative freq (%)
97,5
95
90
80
70
60
50
40
30
20
10
5
2.5
0.4
0.6
0.8
1.0
1.4
1.8
2.4
TG

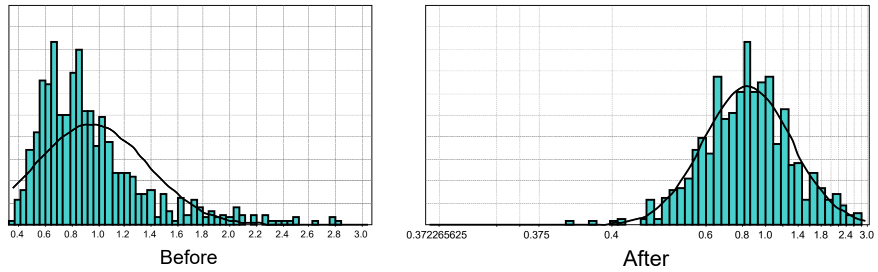

TG M n=669
Para: 0.469 ~ 0.850 ~ 2.095
Nonpara: 0.453 ~ 0.852 ~ 2.213
Pow=0.165 TPos=0.372
Kurt=-0.201 Skew=0.011
K-S test for normality: .782 (NS)

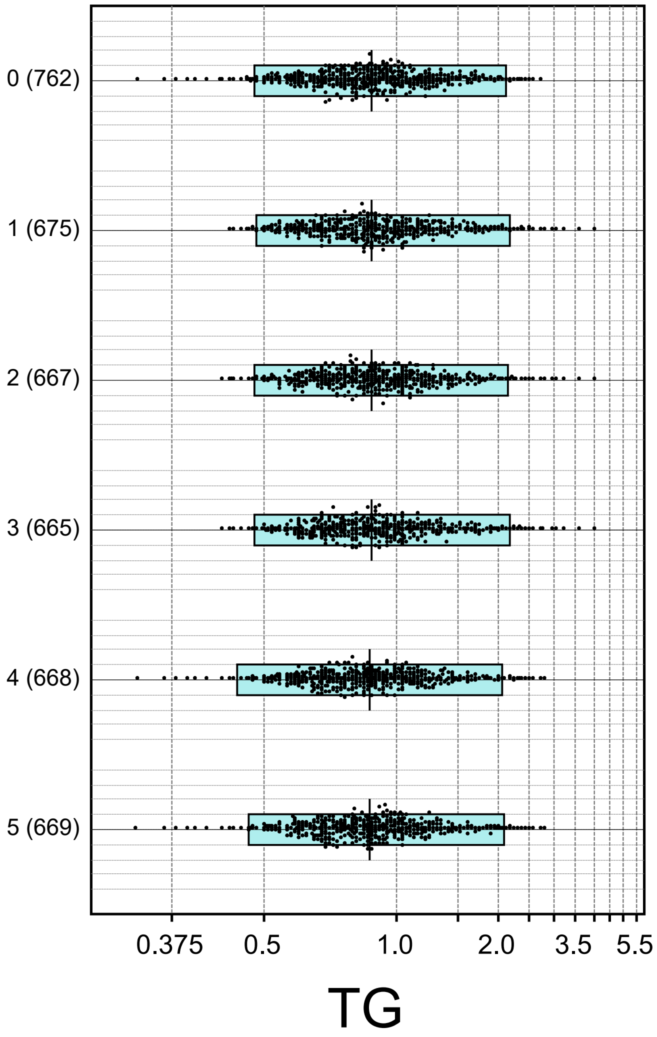

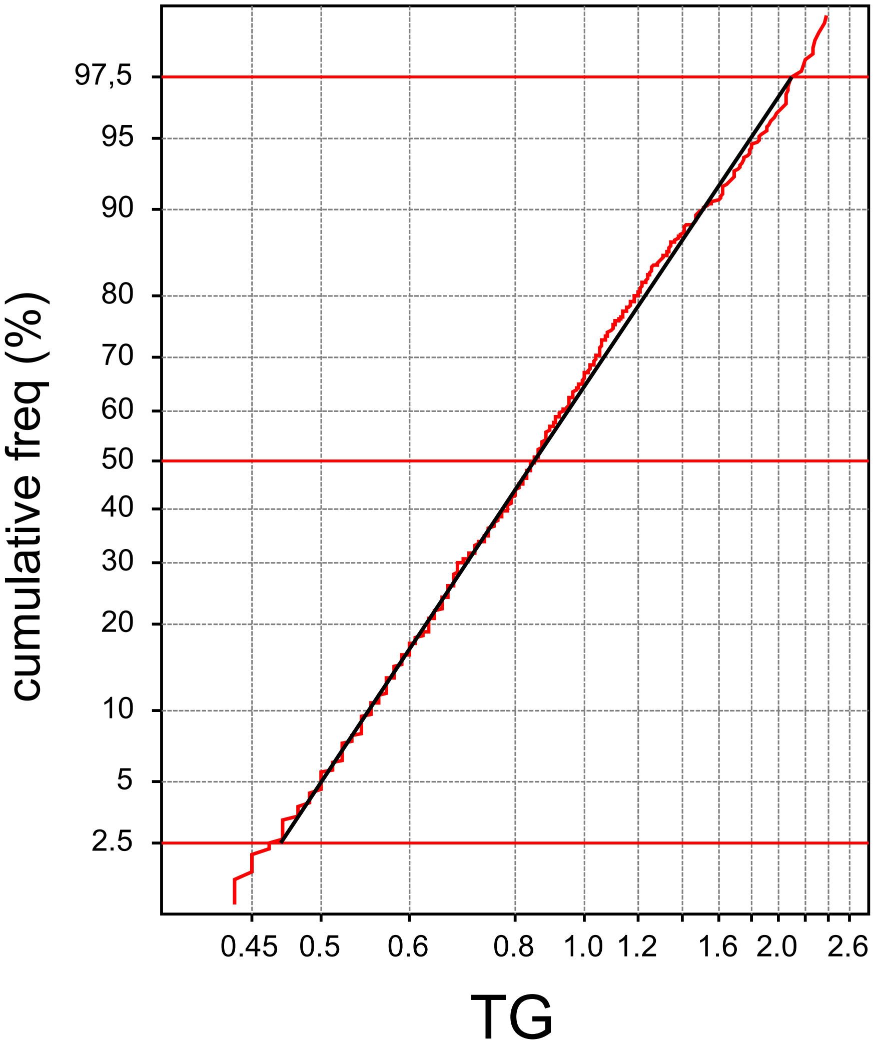

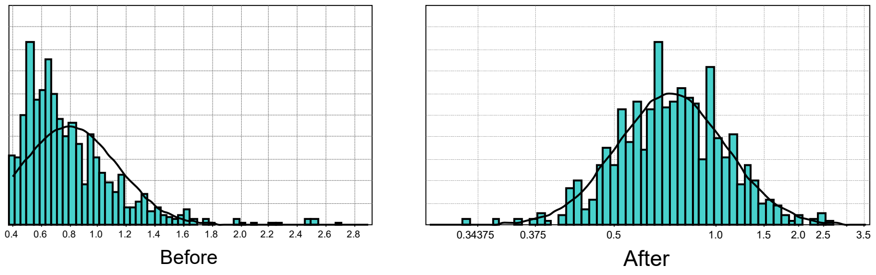

TG F n=776
Para: 0.412 ~ 0.717 ~ 1.656
Nonpara: 0.402 ~ 0.712 ~ 1.636
Pow=0.181 TPos=0.332
Kurt=-0.209 Skew=0.01
K-S test for normality: .635 (NS)

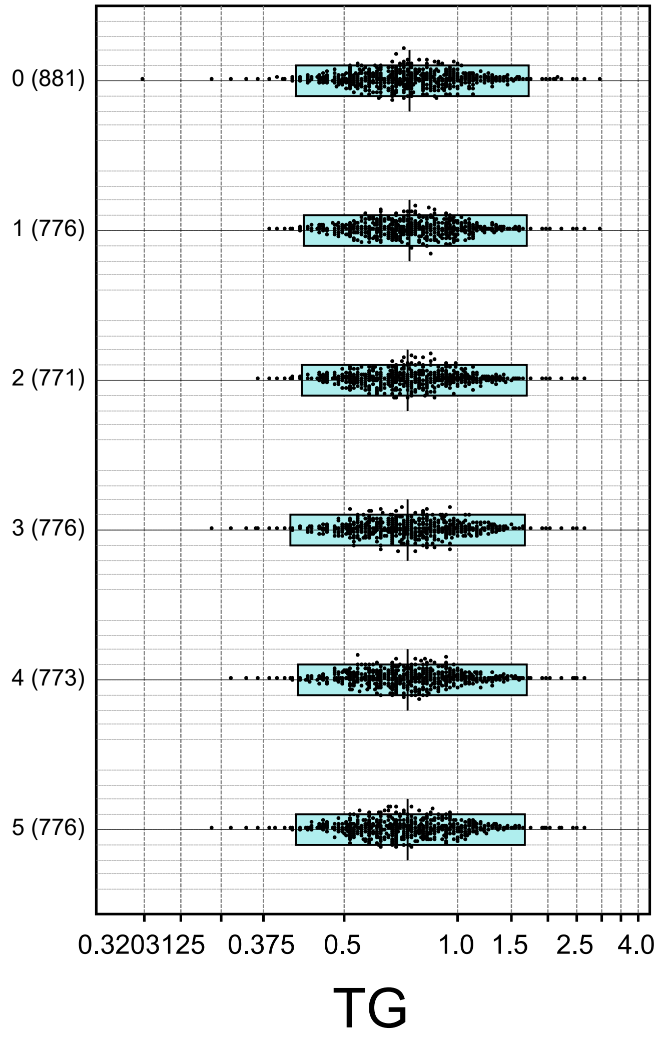

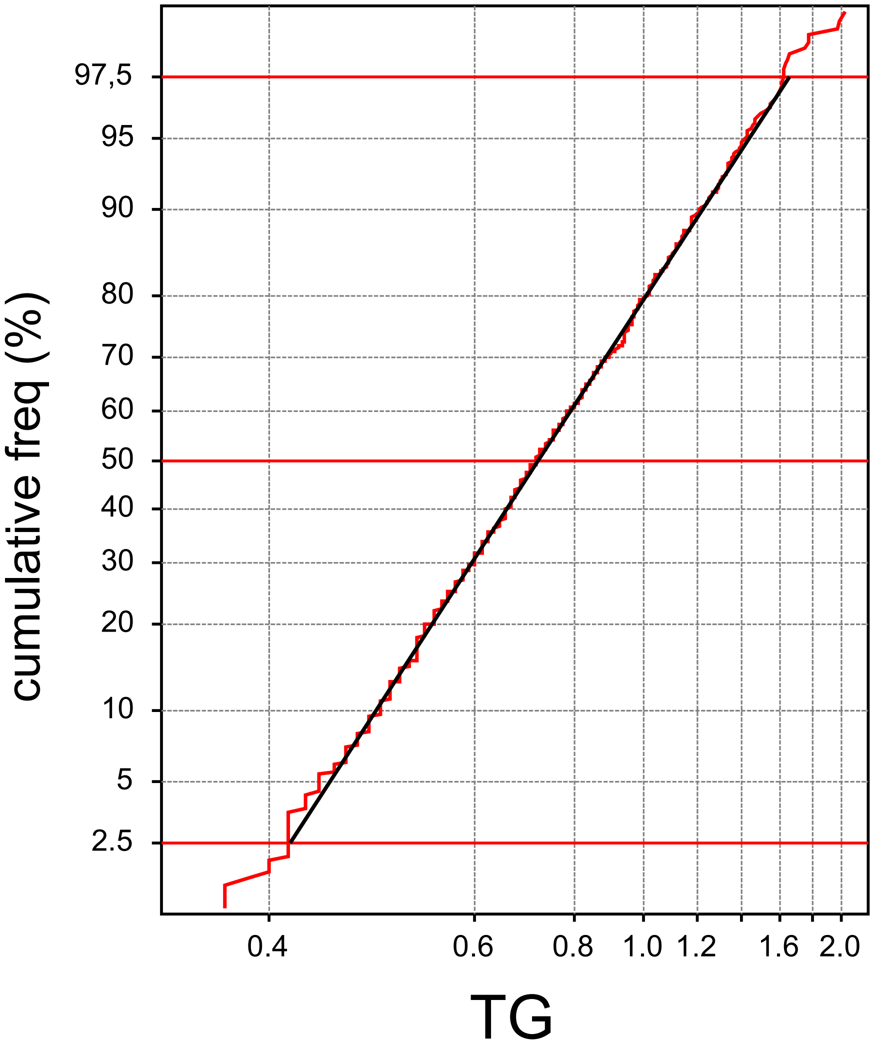

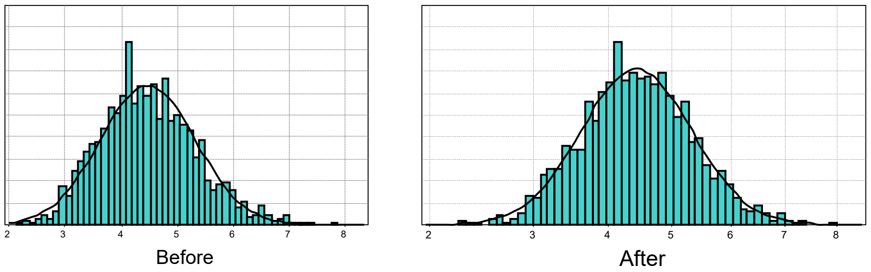

CHOL MF n=1,446
Para: 2.95 ~ 4.43 ~ 6.23
Nonpara: 2.97 ~ 4.42 ~ 6.33
Pow=0.721 TPos=1.922
Kurt=-0.256 Skew=0.056
K-S test for normality: .905 (NS)

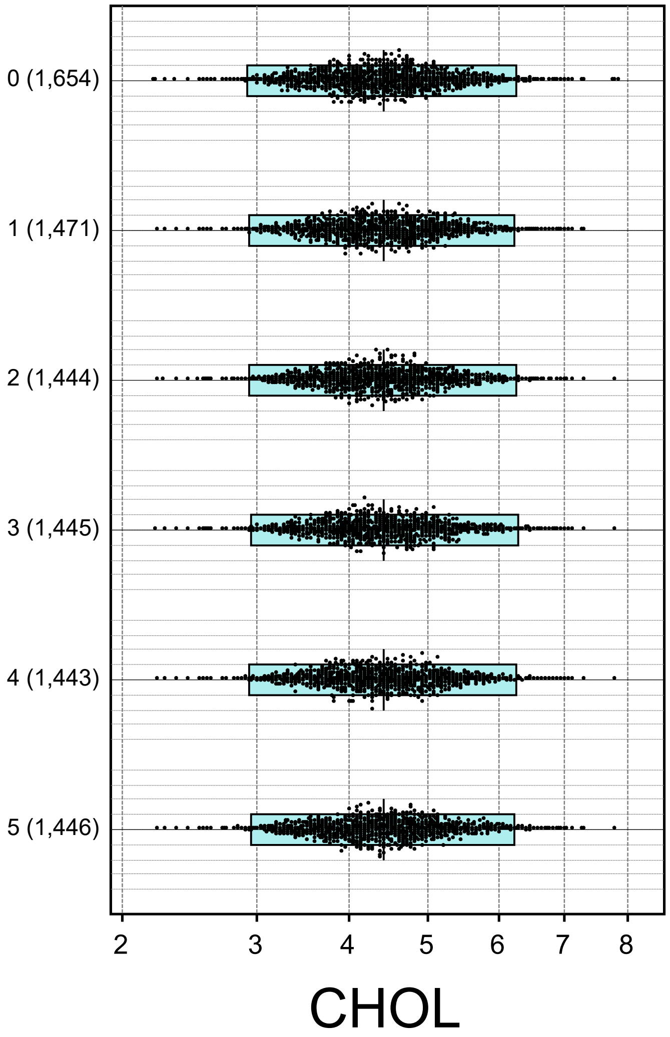

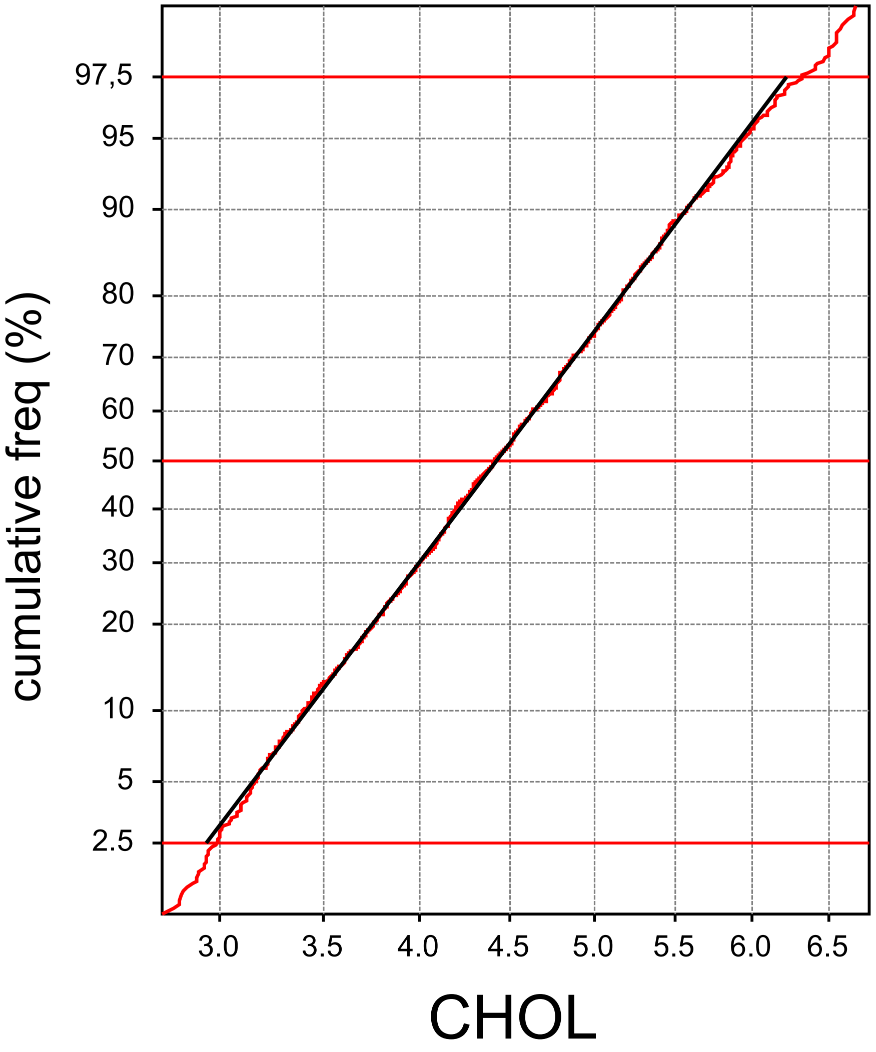

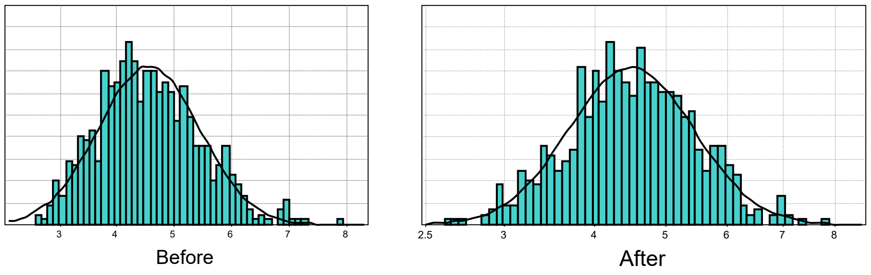

CHOL M n=674
Para: 3.03 ~ 4.49 ~ 6.48
Nonpara: 3.01 ~ 4.50 ~ 6.41
Pow=0.667 TPos=2.493
Kurt=-0.319 Skew=-0.012
K-S test for normality: P≈1.00 (N.S.)

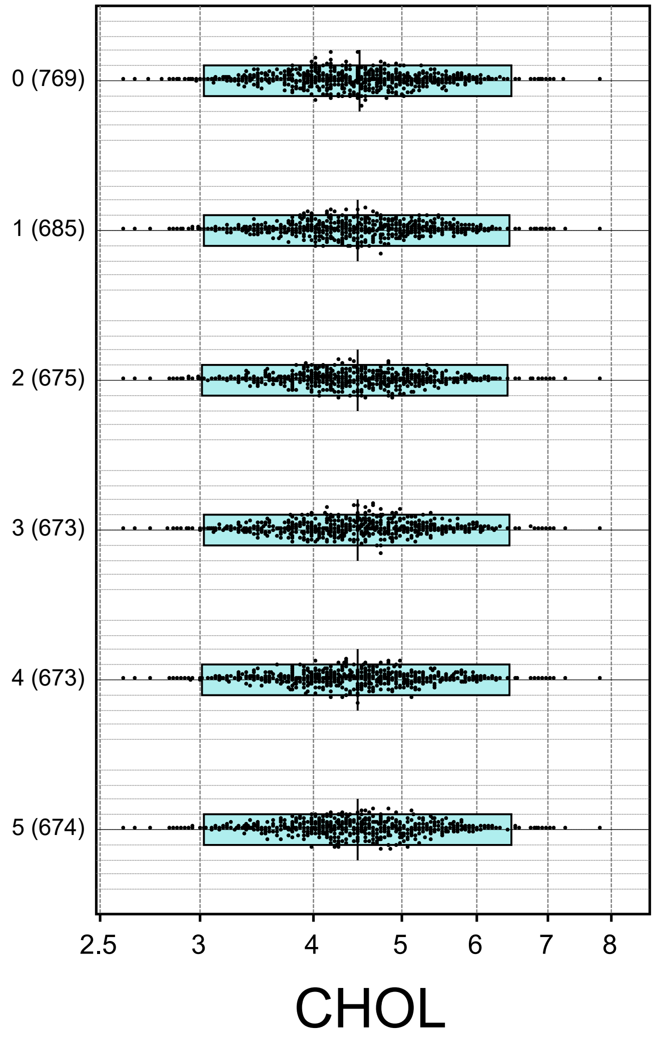

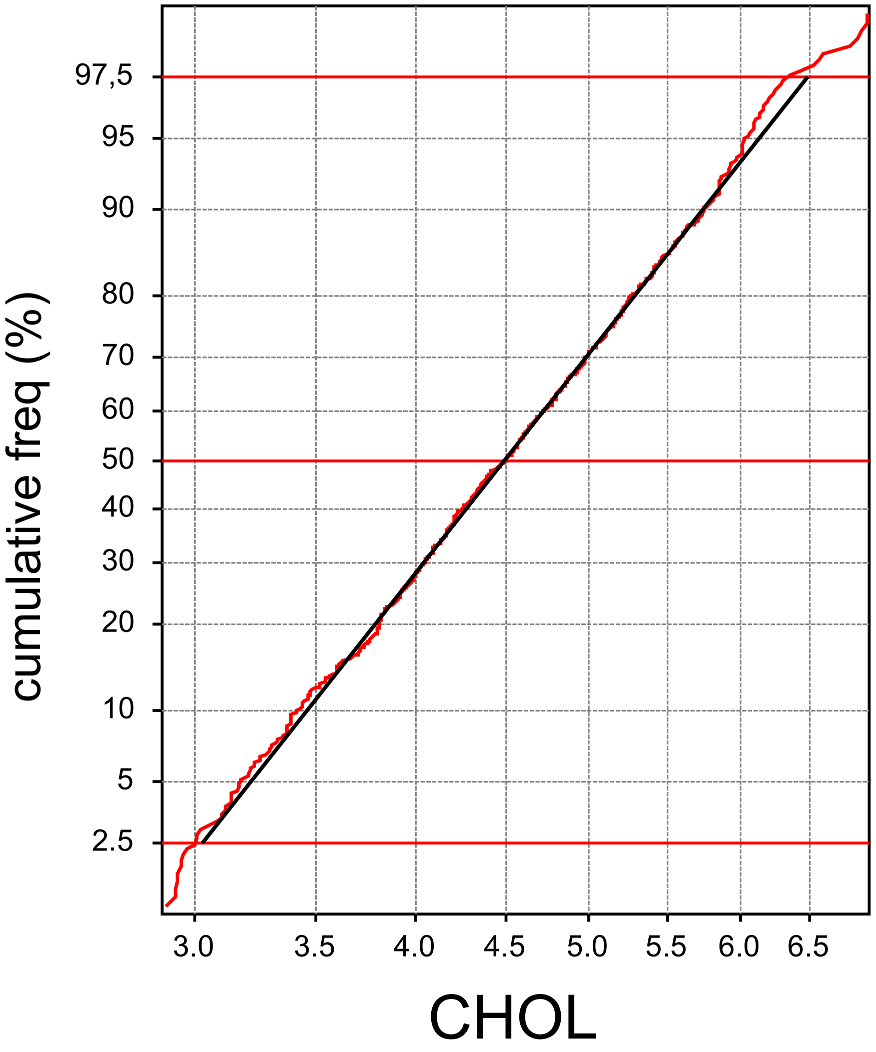

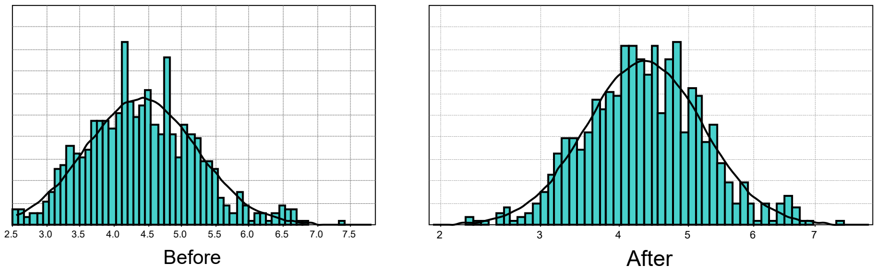

CHOL F n=779
Para: 2.90 ~ 4.36 ~ 6.07
Nonpara: 2.97 ~ 4.36 ~ 6.33
Pow=0.778 TPos=1.87
Kurt=-0.18 Skew=0.033
K-S test for normality: P≈1.00 (N.S.)

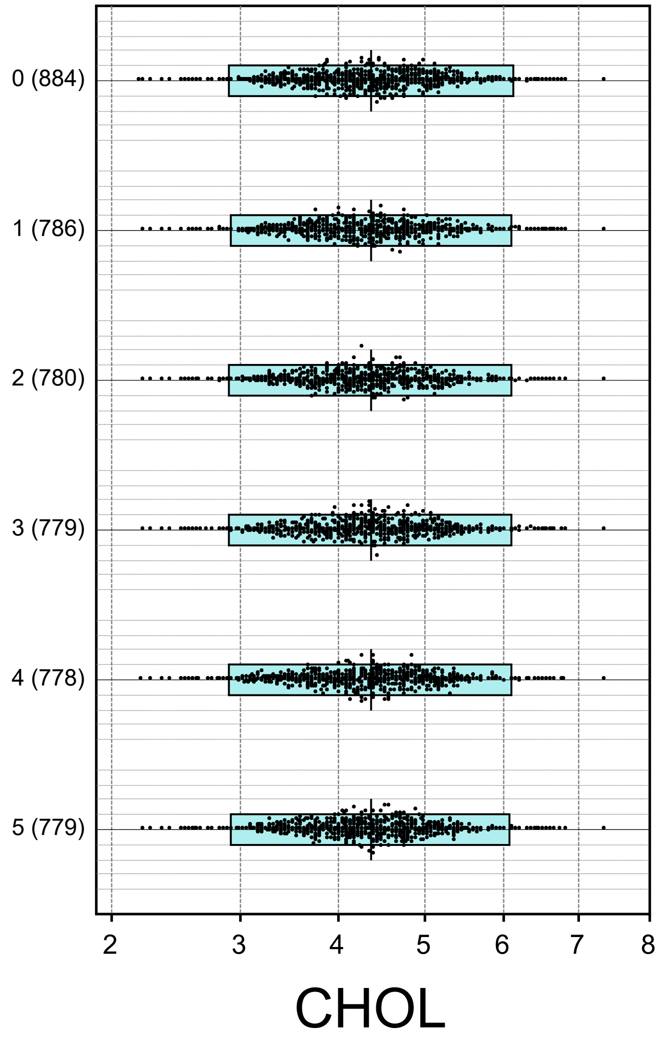

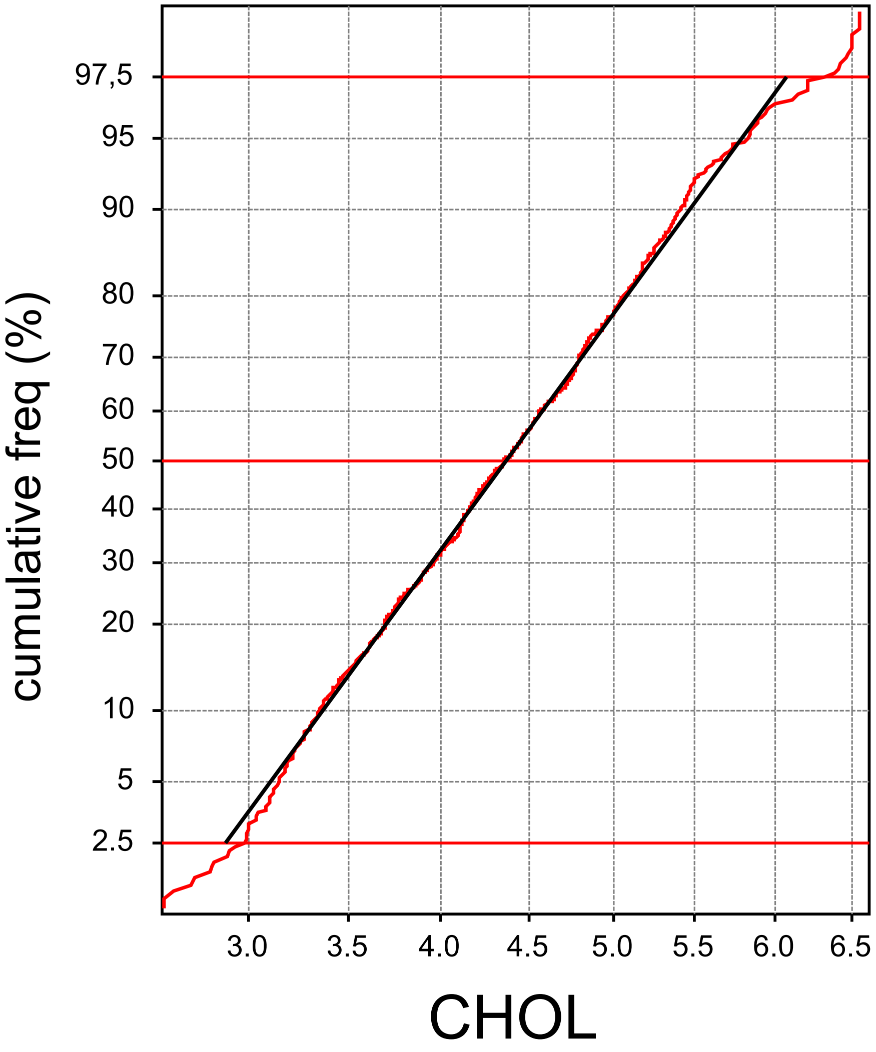

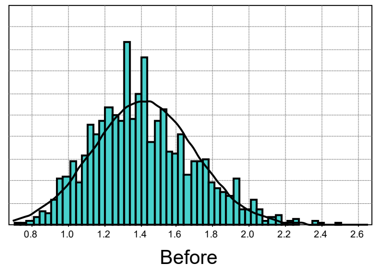

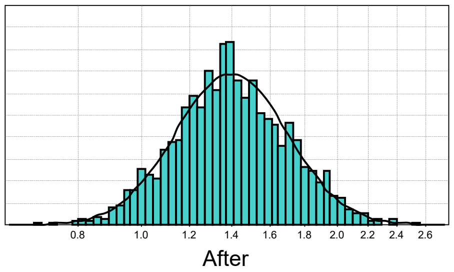

HDLC MF n=1,444
Para: 0.928 ~ 1.394 ~ 2.025
Nonpara: 0.932 ~ 1.389 ~ 2.027
Pow=0.62 TPos=0.672
Kurt=-0.313 Skew=0.064
K-S test for normality: .249 (NS)

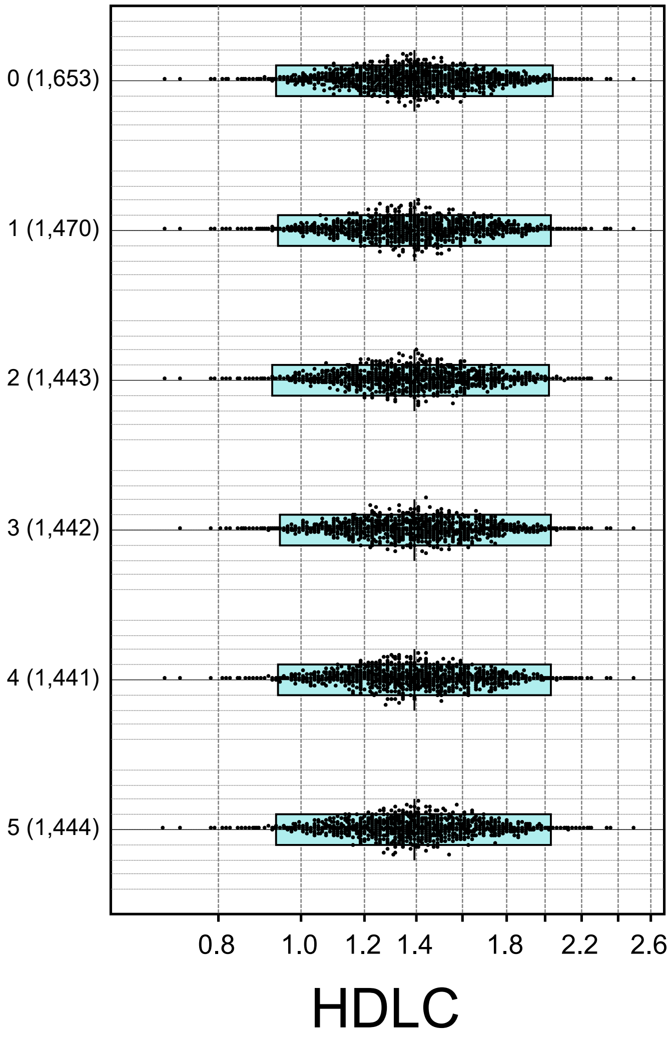

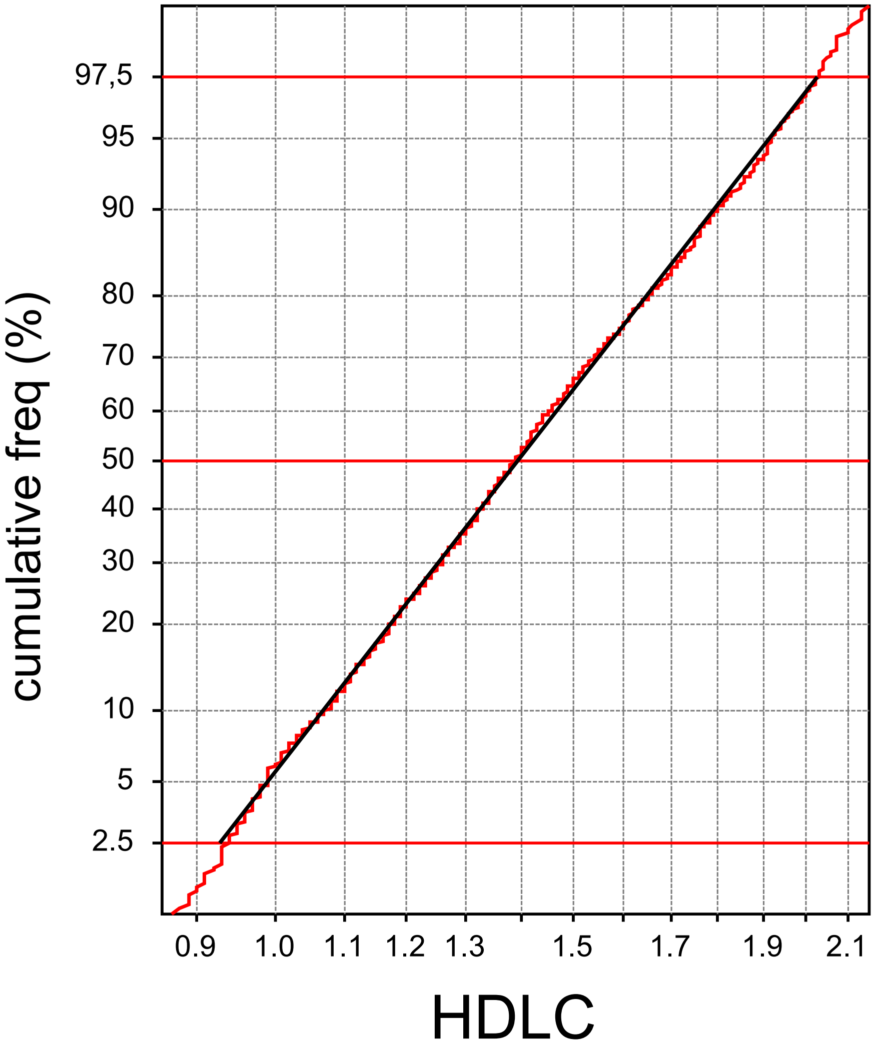

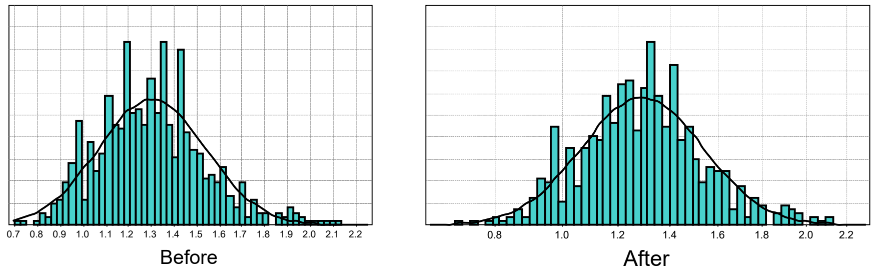

HDLC M n=673
Para: 0.878 ~ 1.287 ~ 1.793
Nonpara: 0.905 ~ 1.291 ~ 1.860
Pow=0.715 TPos=0.615
Kurt=-0.285 Skew=-0.016
K-S test for normality: .419 (NS)

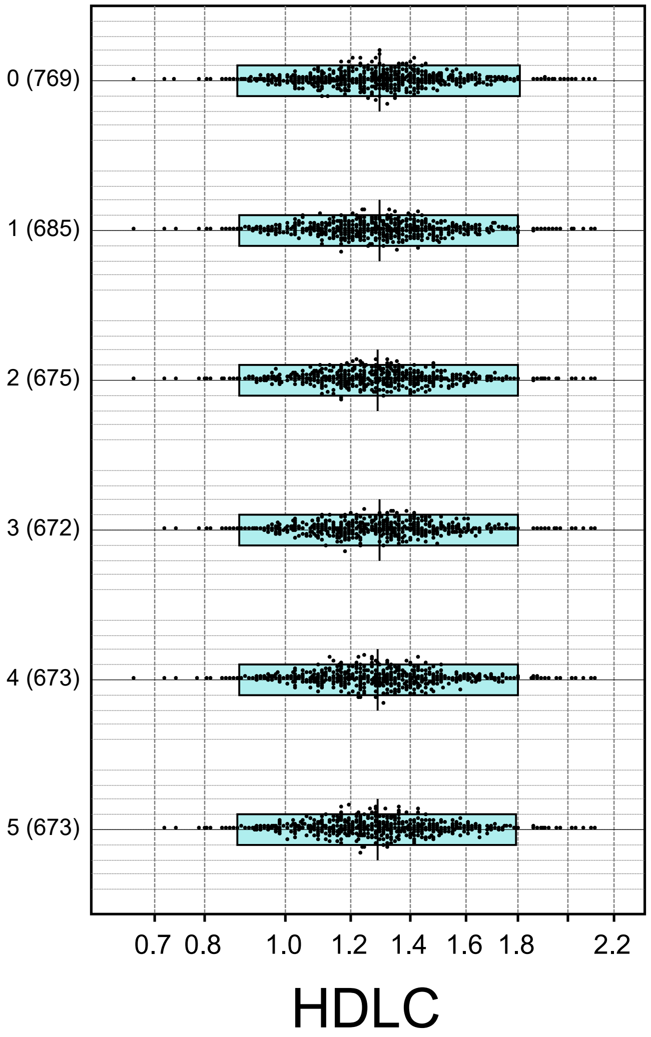

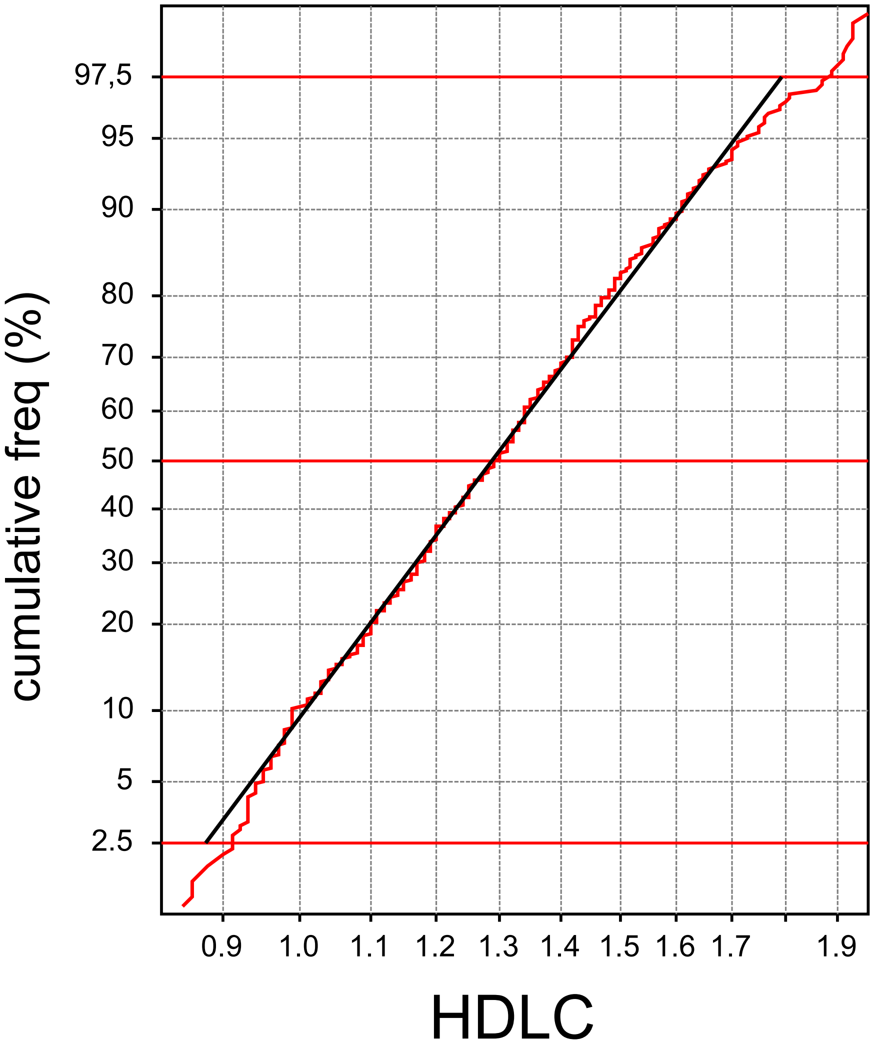

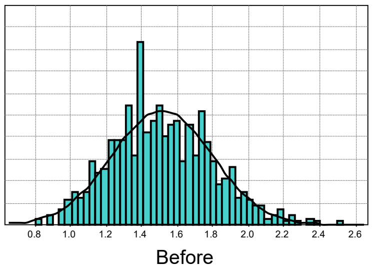

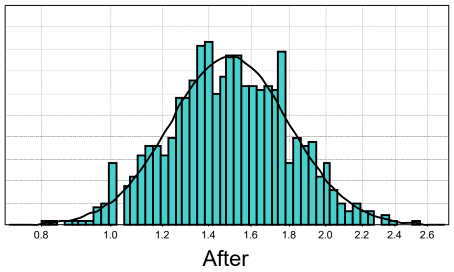

HDLC F n=778
Para: 1.005 ~ 1.491 ~ 2.107
Nonpara: 1.005 ~ 1.493 ~ 2.085
Pow=0.688 TPos=0.721
Kurt=-0.351 Skew=0.006
K-S test for normality: .939 (NS)

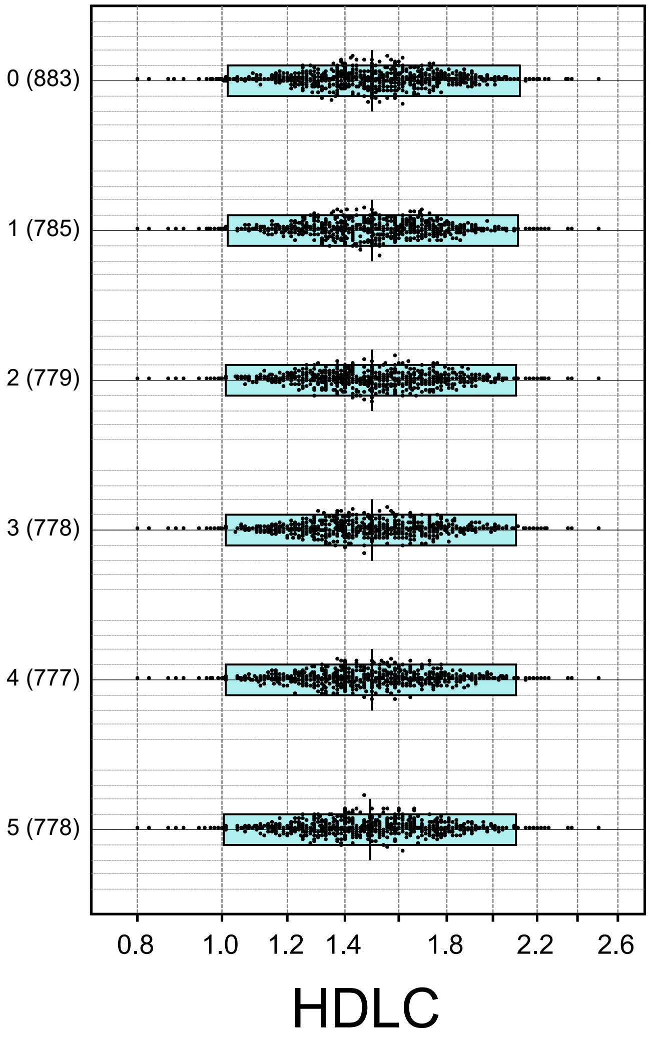

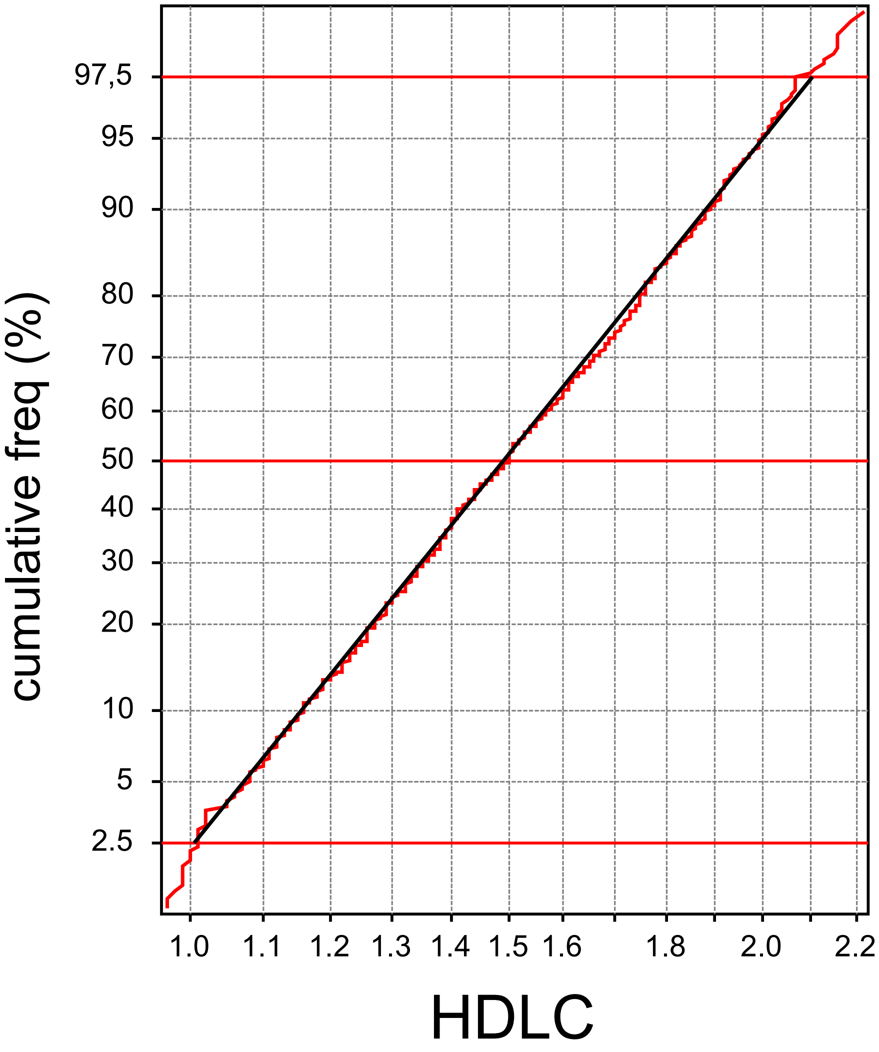

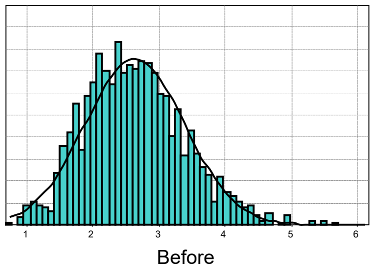

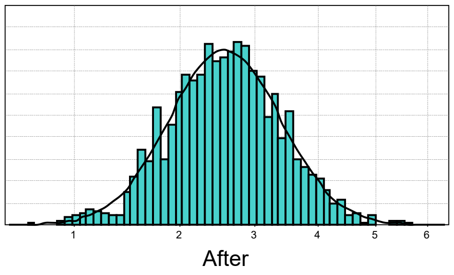

LDLC MF n=1,446
Para: 1.334 ~ 2.573 ~ 4.245
Nonpara: 1.311 ~ 2.579 ~ 4.221
Pow=0.63 TPos=0.692
Kurt=-0.251 Skew=-0.05
K-S test for normality: P≈1.00 (N.S.)

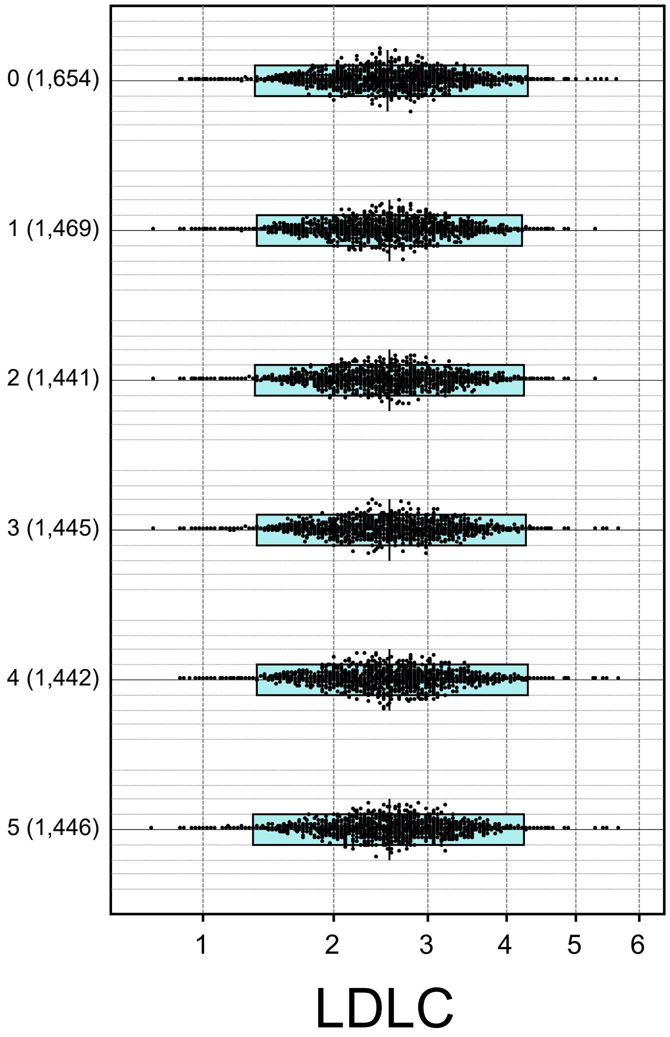

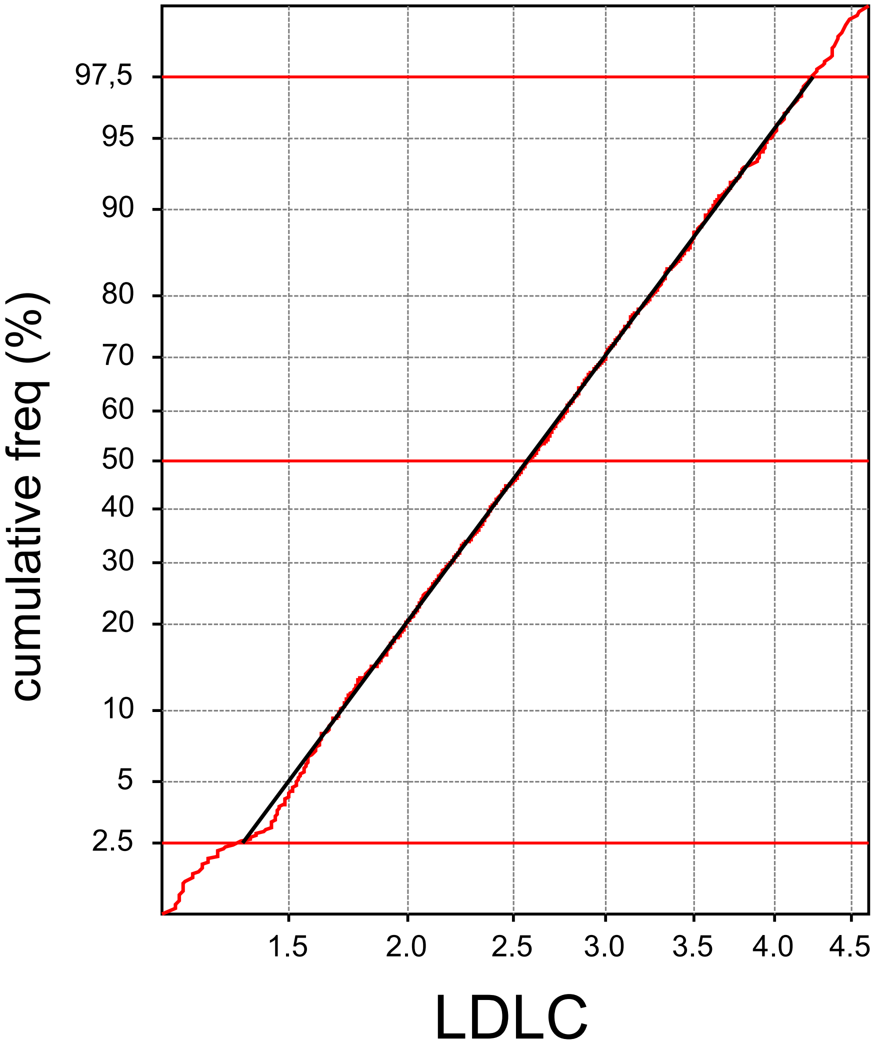

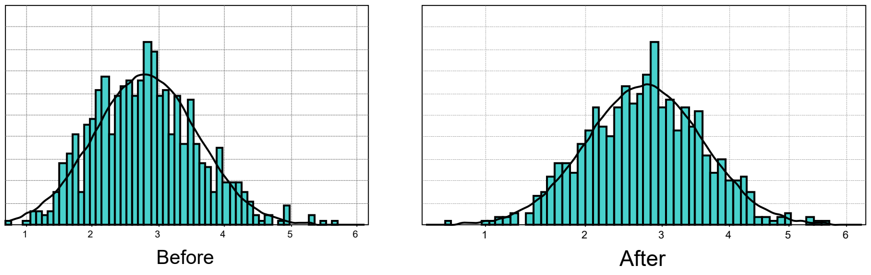

LDLC M n=674
Para: 1.428 ~ 2.766 ~ 4.476
Nonpara: 1.487 ~ 2.788 ~ 4.395
Pow=0.685 TPos=0.655
Kurt=-0.328 Skew=-0.042
K-S test for normality: P≈1.00 (N.S.)

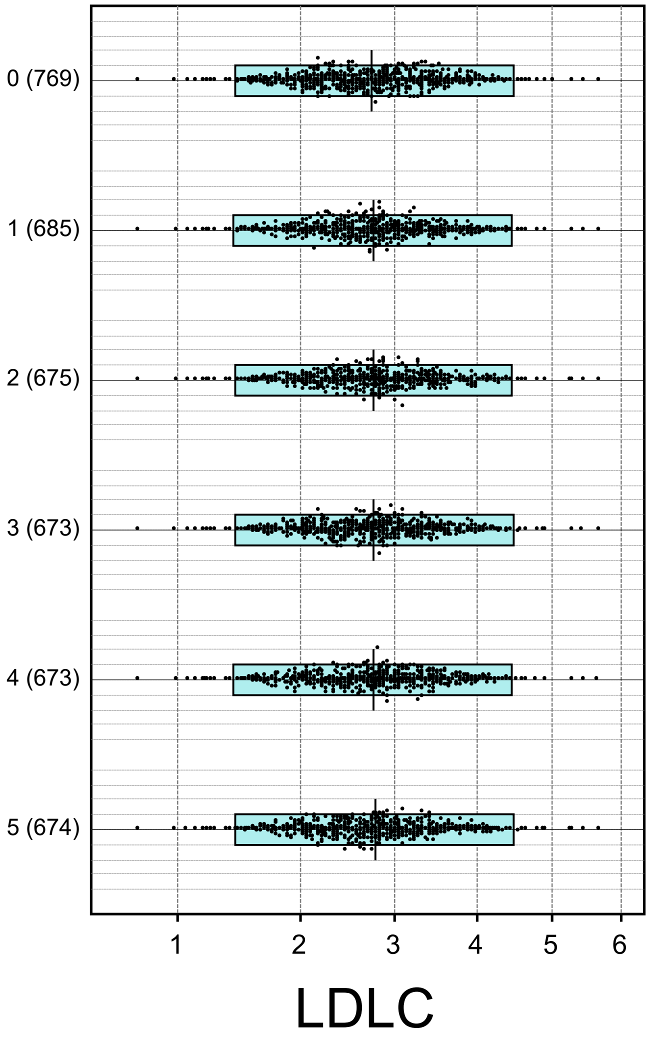

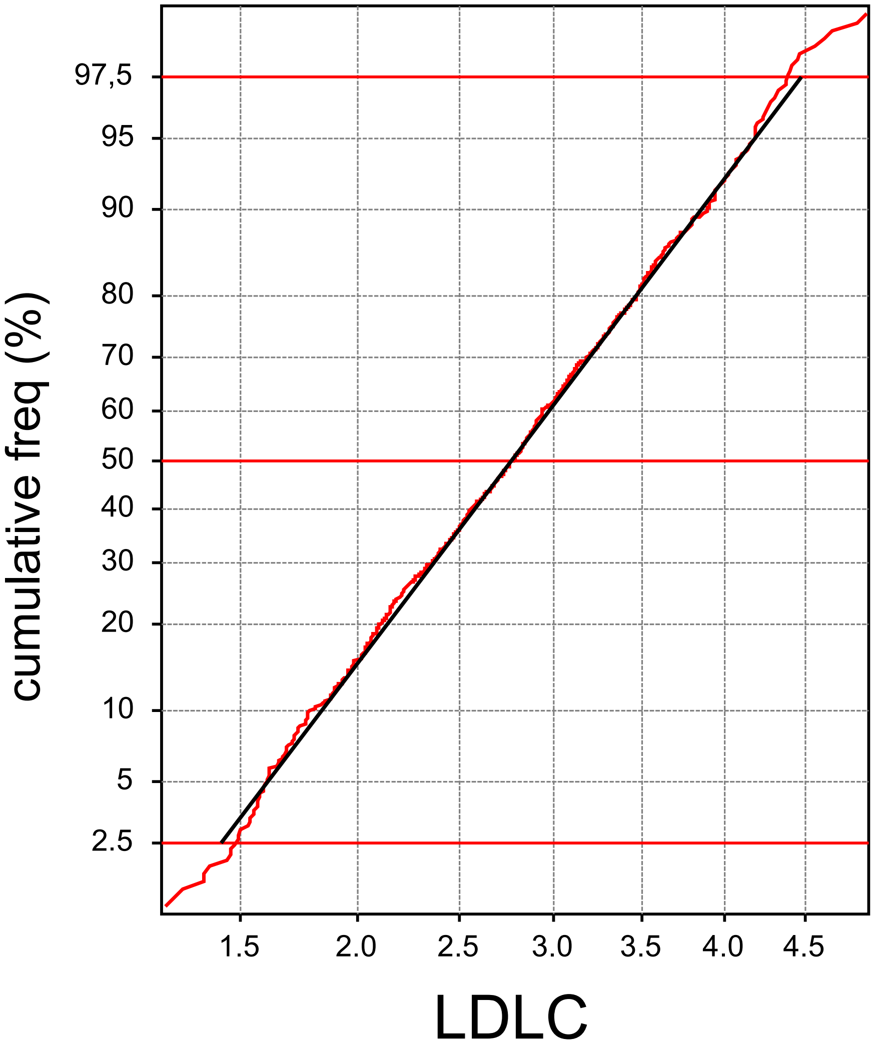

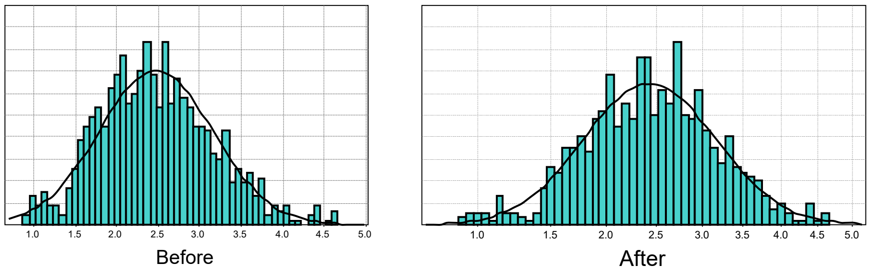

LDLC F n=778
Para: 1.271 ~ 2.426 ~ 3.980
Nonpara: 1.173 ~ 2.425 ~ 3.959
Pow=0.608 TPos=0.567
Kurt=-0.193 Skew=-0.056
K-S test for normality: P≈1.00 (N.S.)

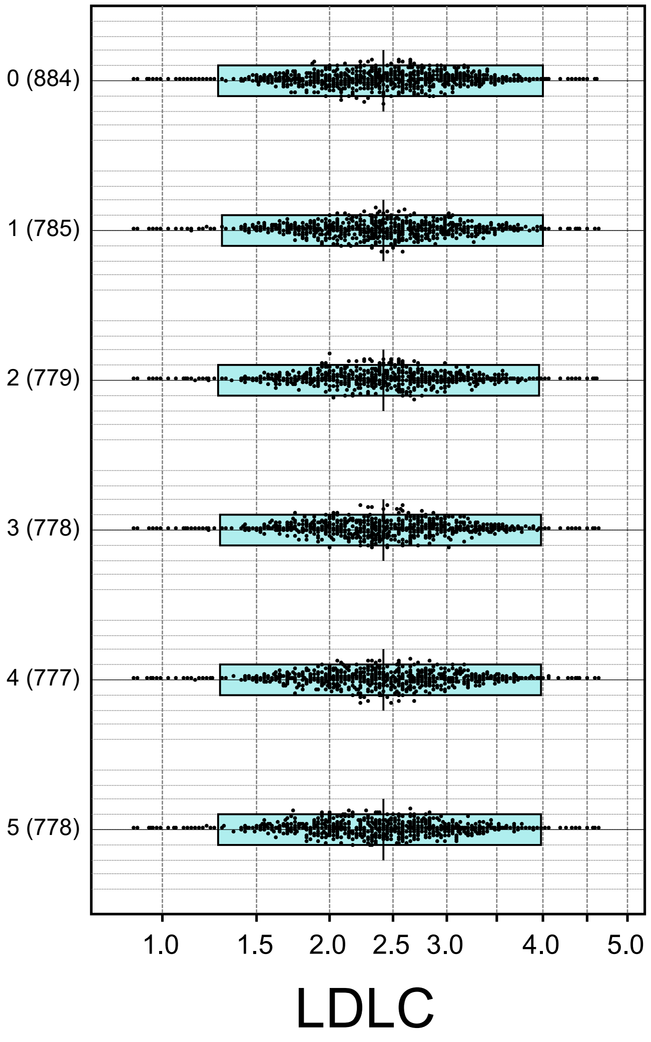

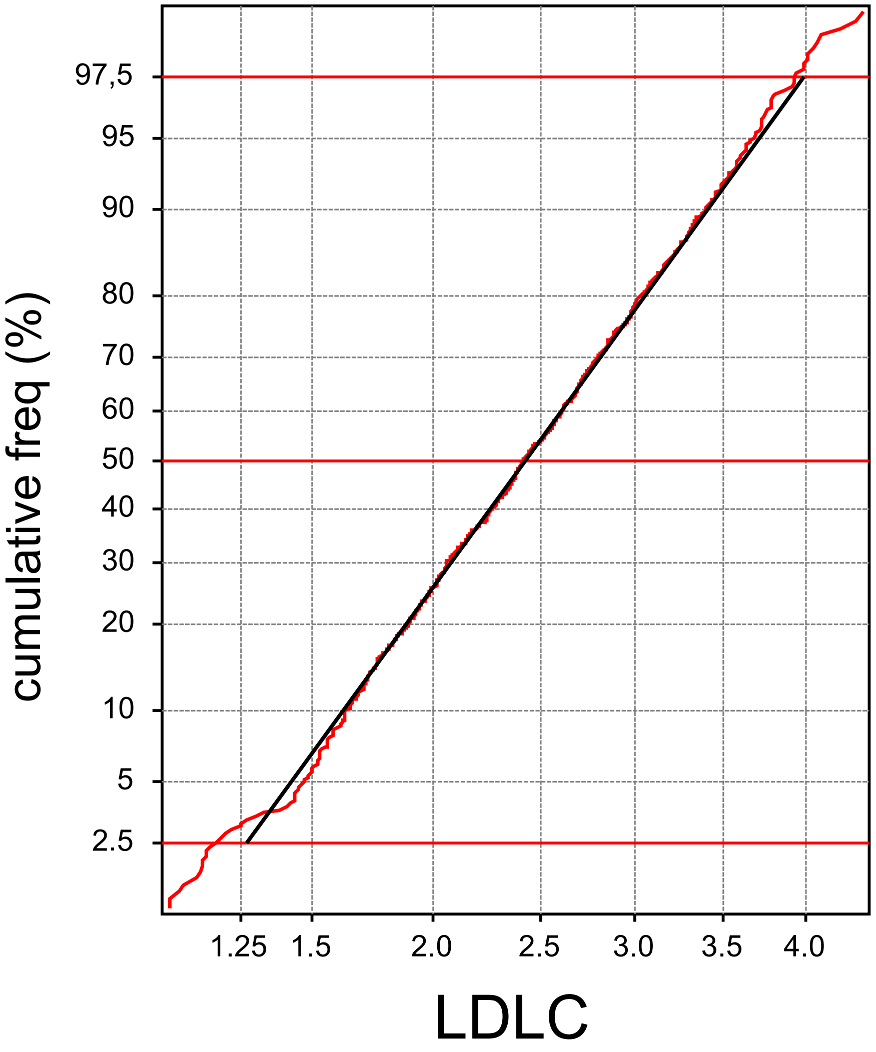

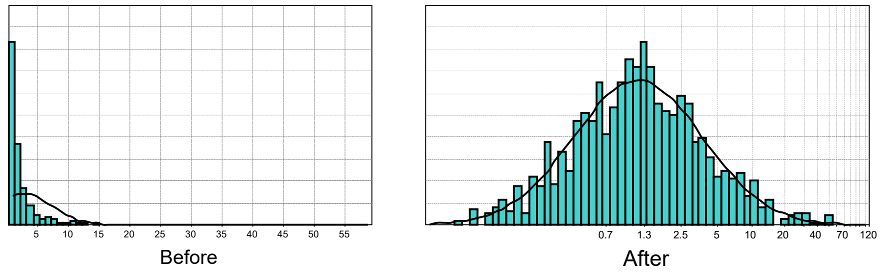

CRP MF n=1,413
Para: 0.26 ~ 1.16 ~ 13.02
Nonpara: 0.25 ~ 1.16 ~ 13.39
Pow=-0.026 TPos=0.163
Kurt=-0.167 Skew=-0.007
K-S test for normality: .545 (NS)

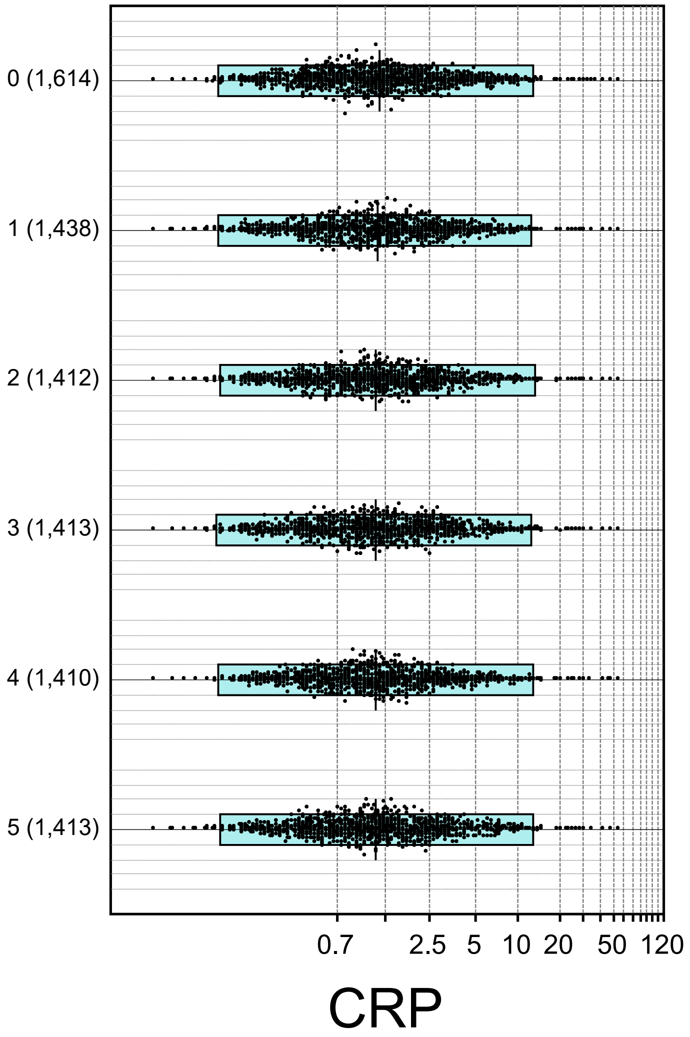

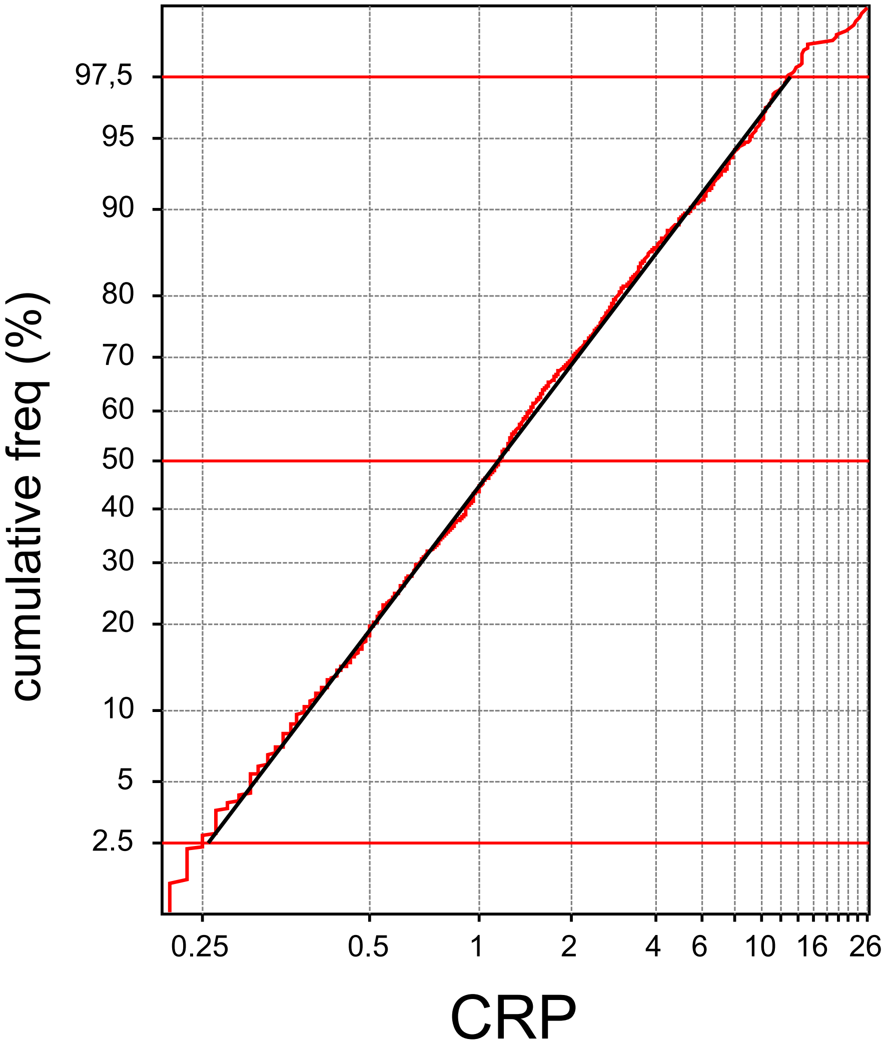

CRP M n=666
Para: 0.27 ~ 1.24 ~ 10.99
Nonpara: 0.26 ~ 1.24 ~ 10.86
Pow=0 TPos=0.156
Kurt=-0.174 Skew=-0.022
K-S test for normality: .979 (NS)

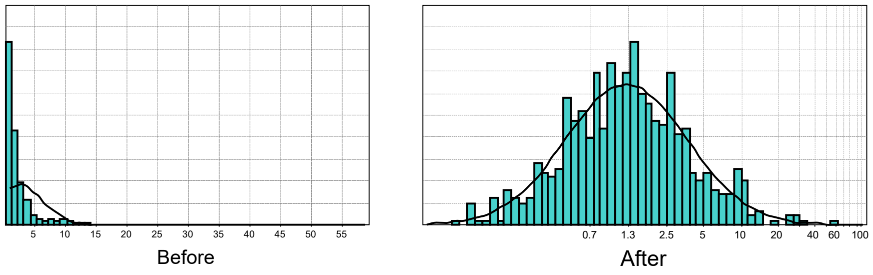

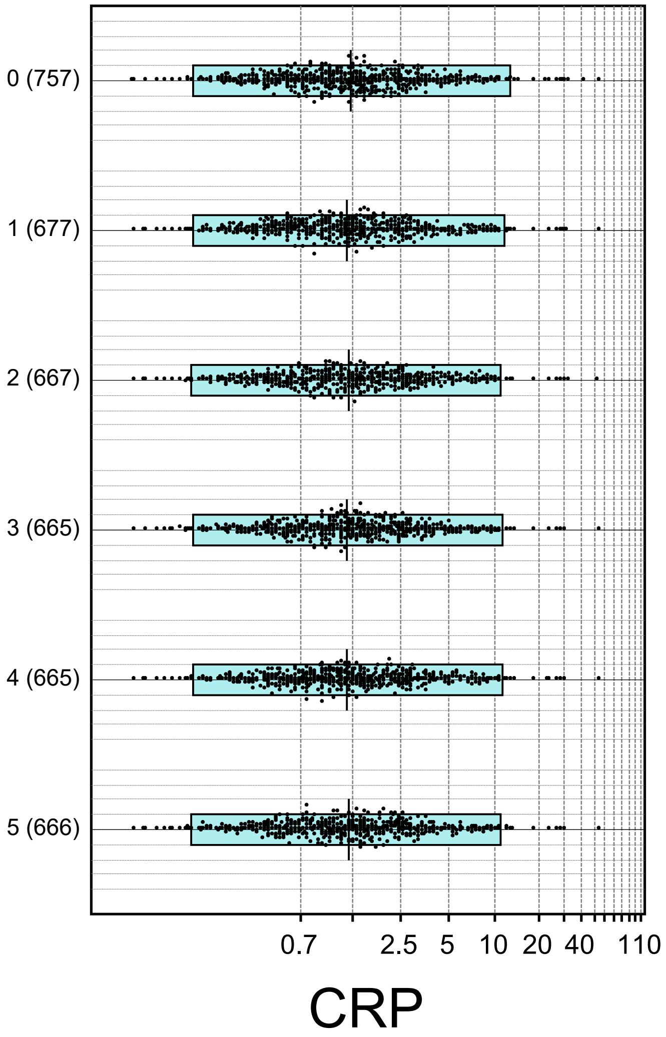

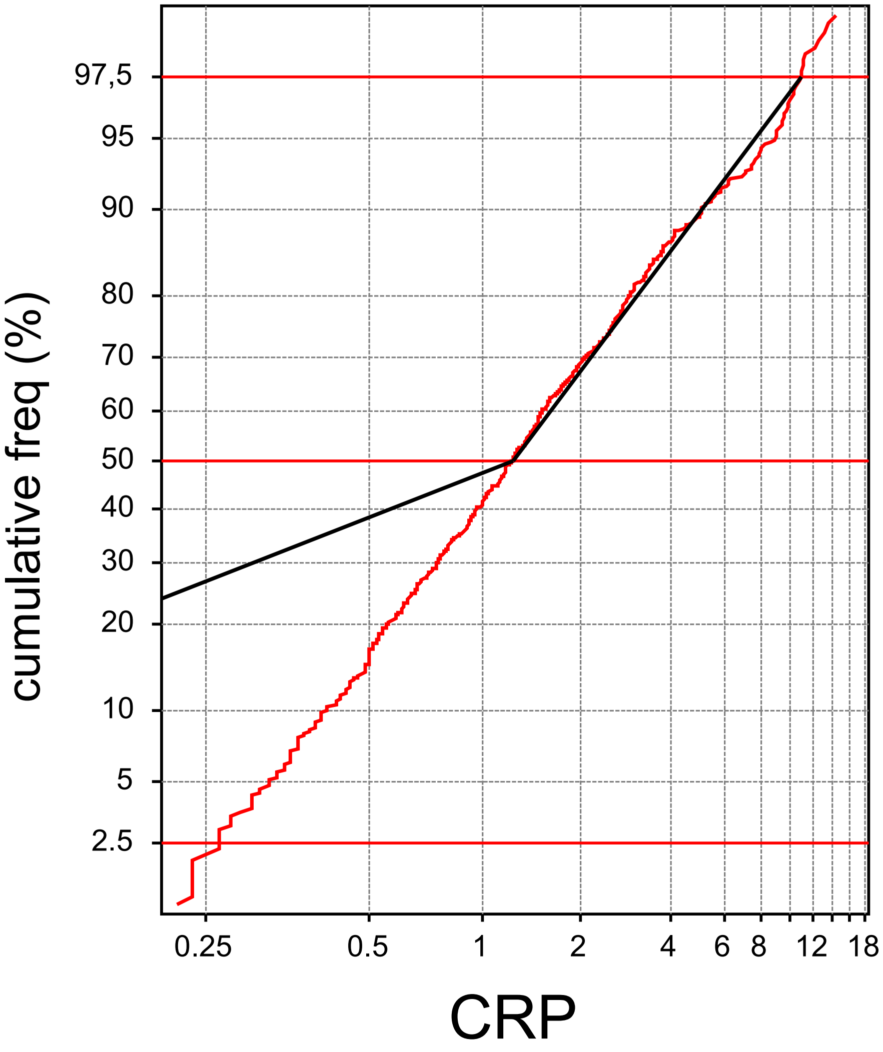

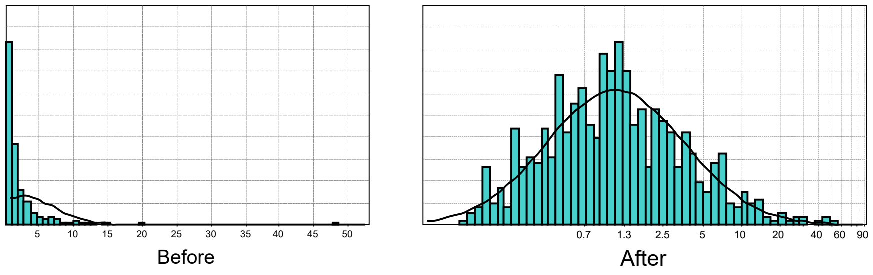

CRP F n=758
Para: 0.22 ~ 1.12 ~ 12.04
Nonpara: 0.24 ~ 1.08 ~ 13.82
Pow=0.005 TPos=0.145
Kurt=-0.268 Skew=0.148
K-S test for normality: .185 (NS)

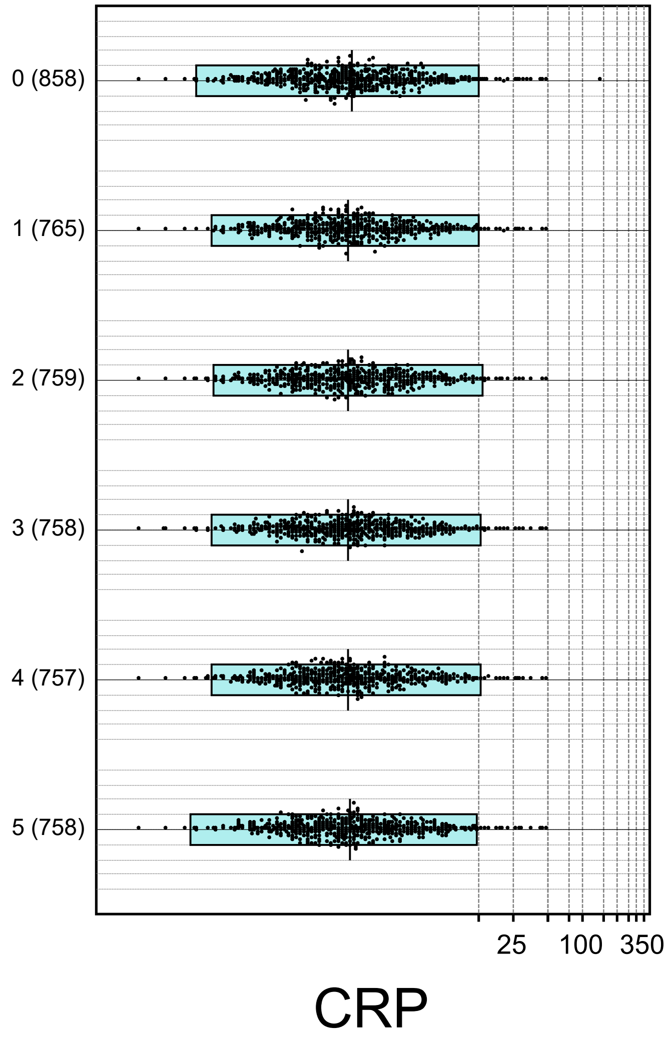

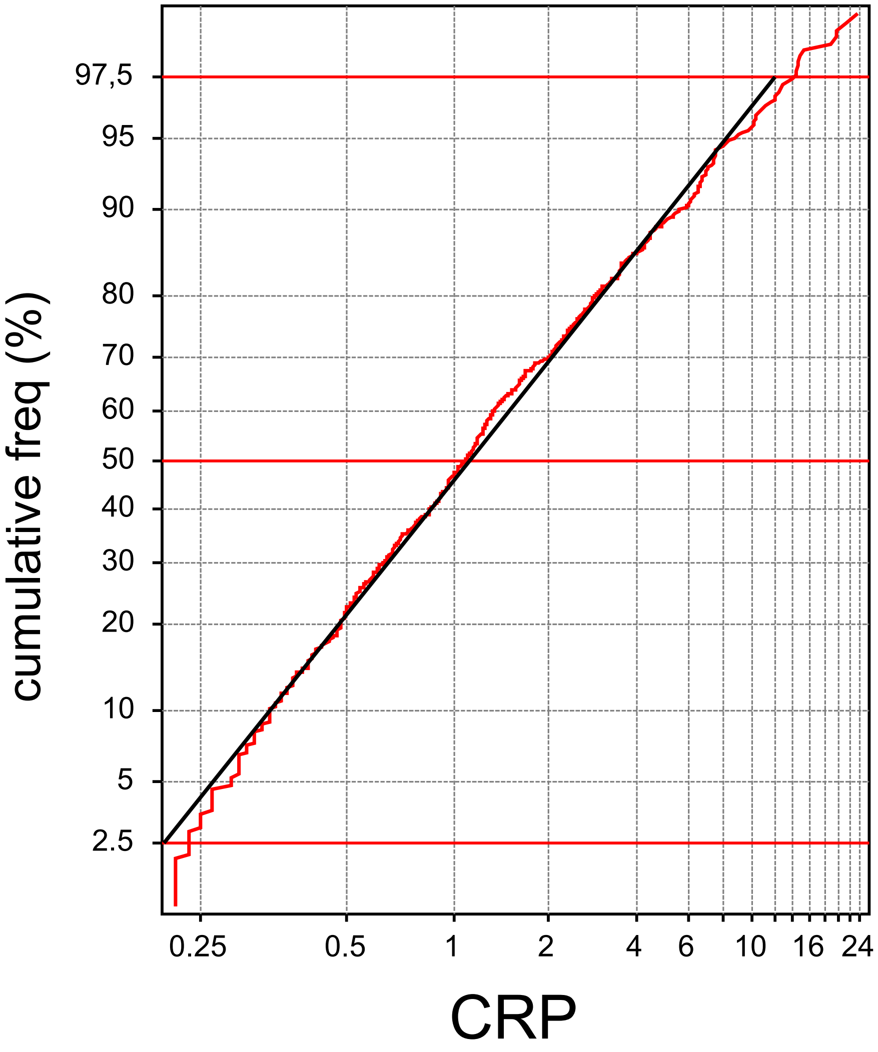

K MF n=1,439
Para: 3.61 ~ 4.17 ~ 4.86
Nonpara: 3.60 ~ 4.20 ~ 4.90
Pow=0.709 TPos=3.236
Kurt=-0.311 Skew=-0.02
K-S test for normality: 0.00001

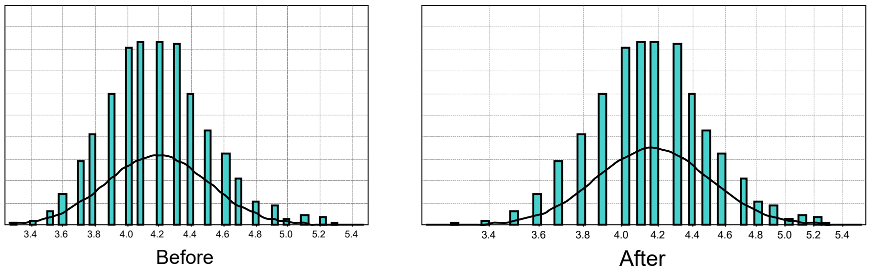

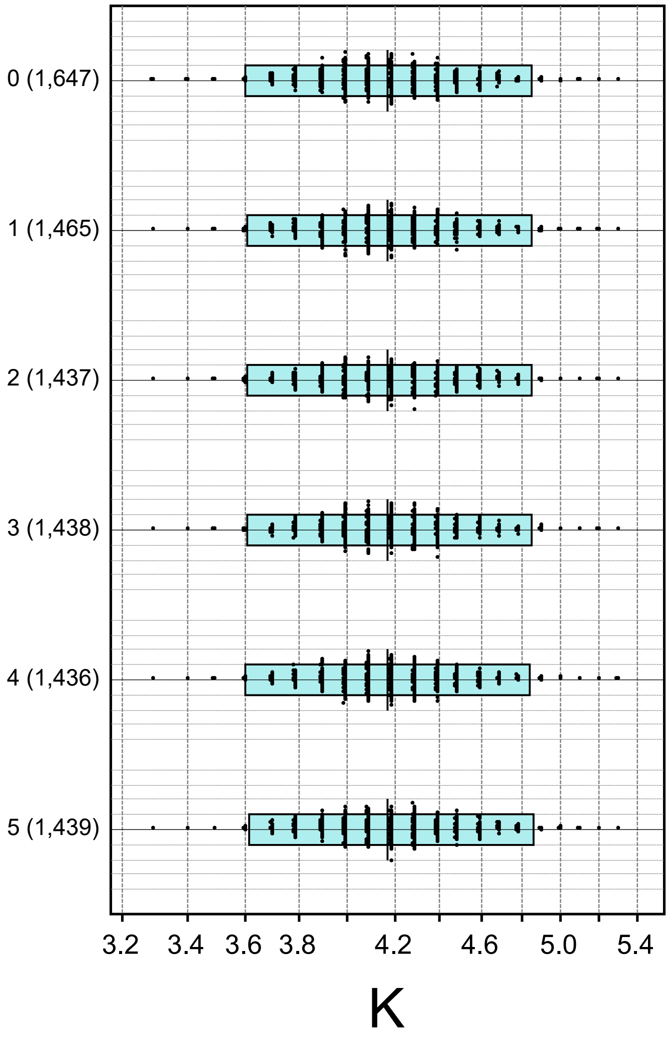

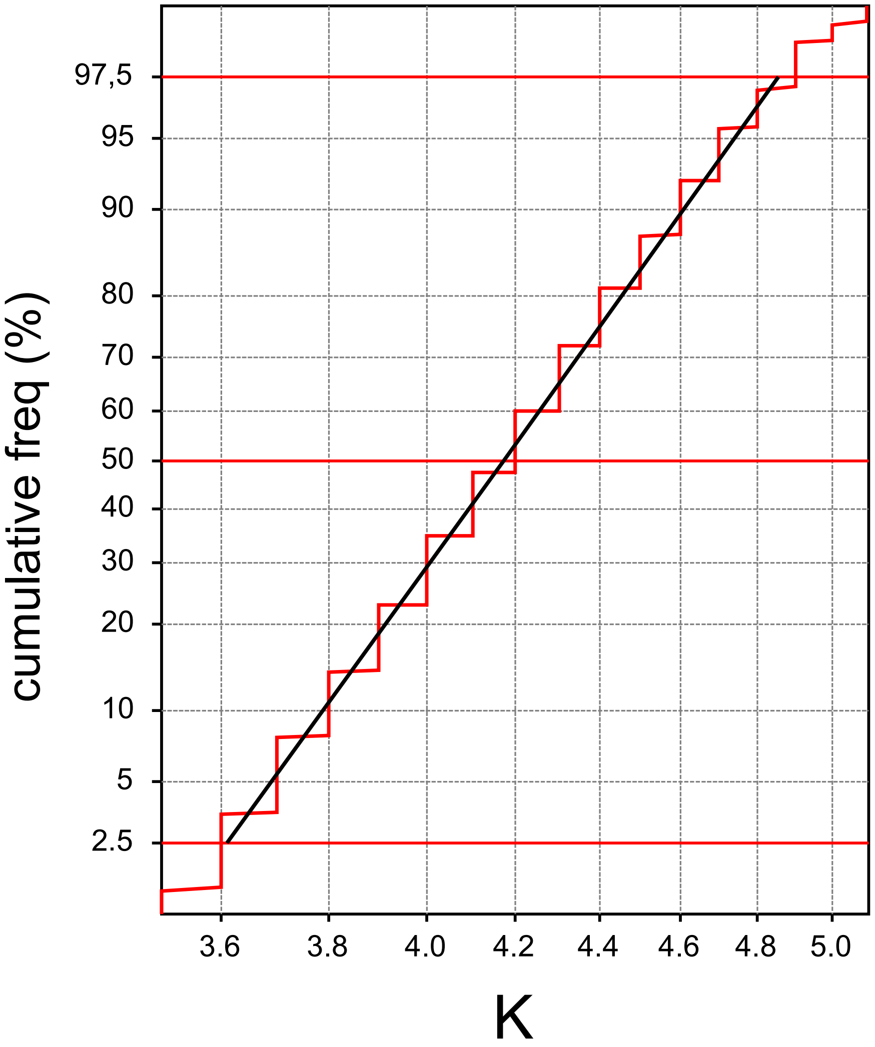

K M n=671
Para: 3.65 ~ 4.22 ~ 4.90
Nonpara: 3.62 ~ 4.20 ~ 5.02
Pow=0.76 TPos=3.271
Kurt=-0.123 Skew=-0.072
K-S test for normality: 0.00048

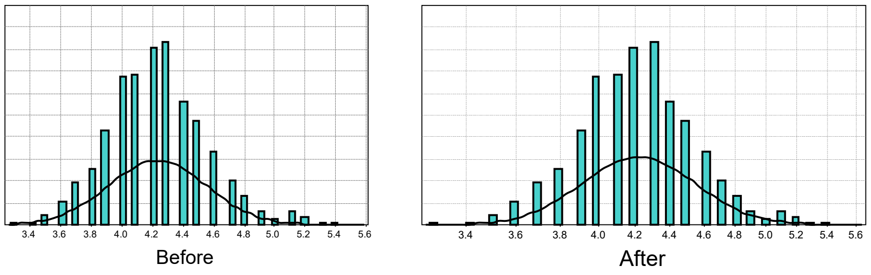

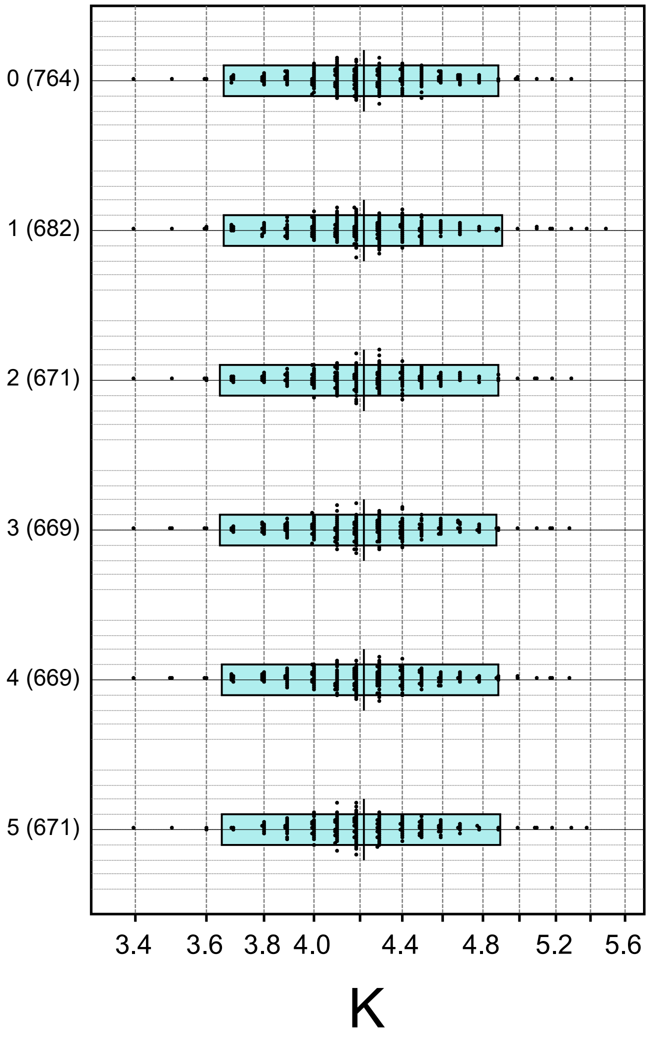

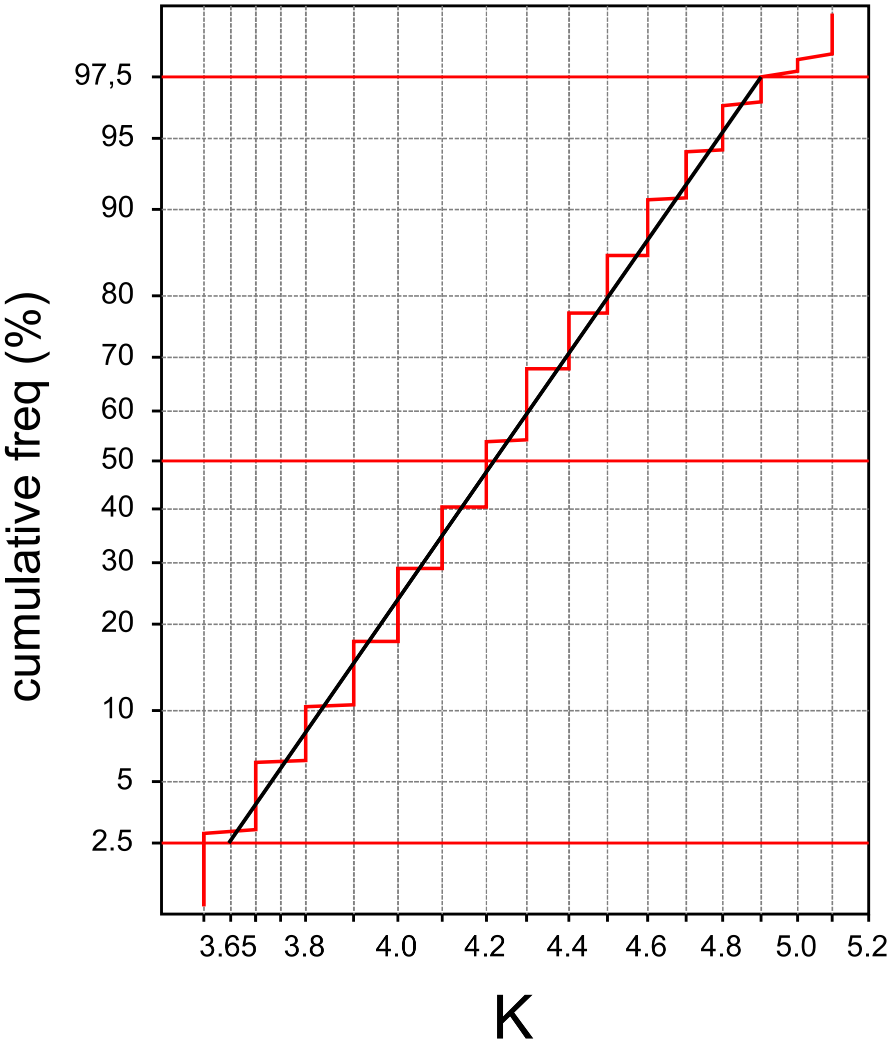

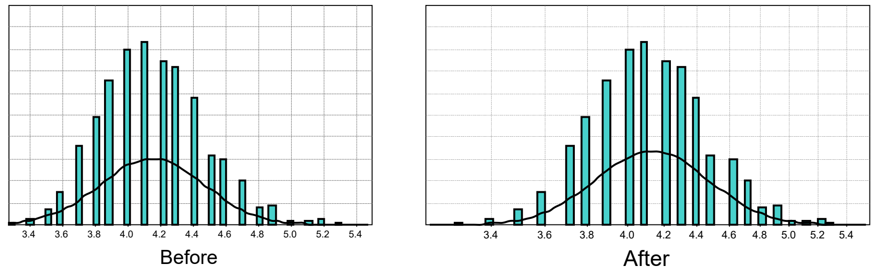

K F n=777
Para: 3.59 ~ 4.13 ~ 4.82
Nonpara: 3.59 ~ 4.11 ~ 4.84
Pow=0.697 TPos=3.251
Kurt=-0.239 Skew=0.067
K-S test for normality: 0.00205

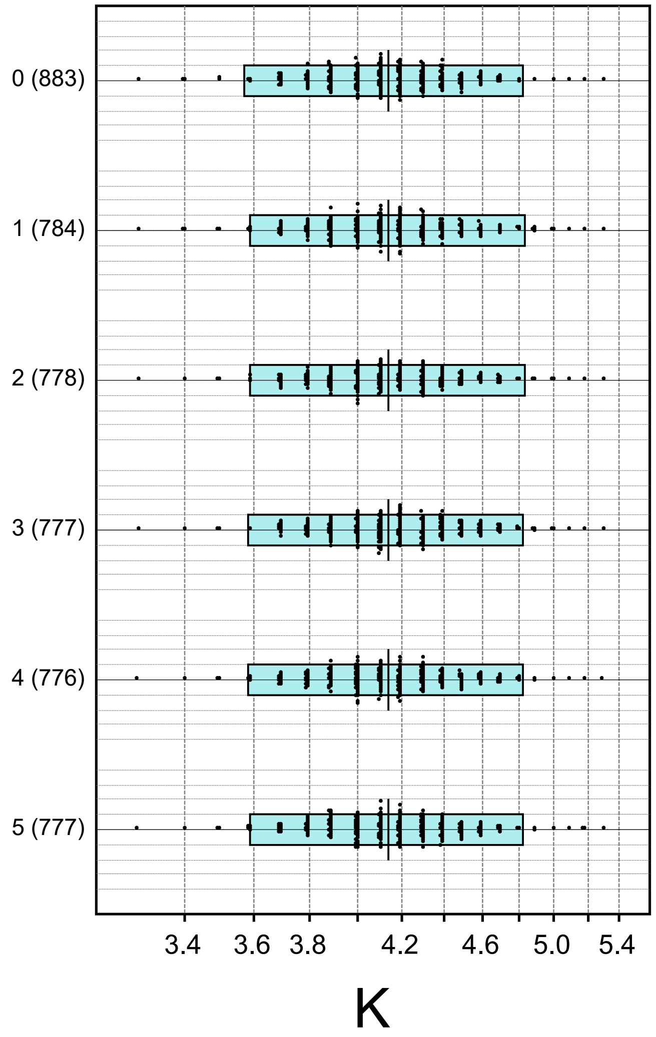

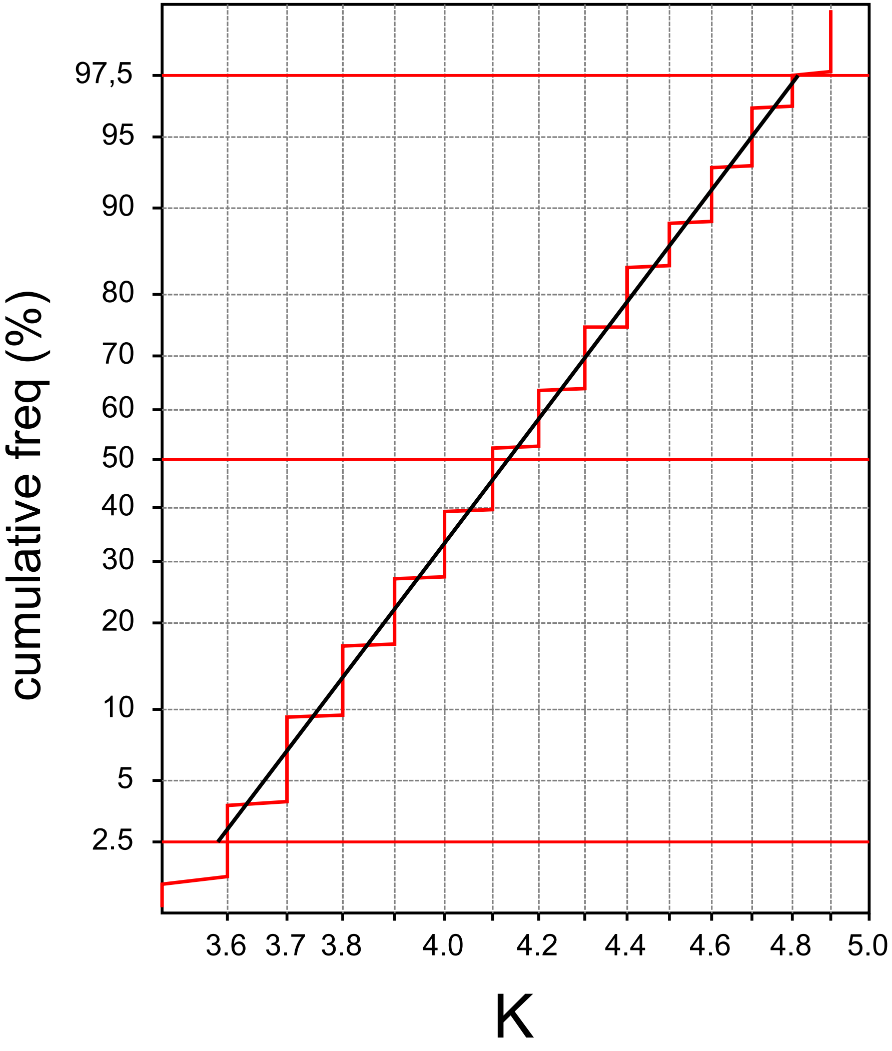

Na MF n=1,437
Para: 137.2 ~ 141.1 ~ 145.8
Nonpara: 137.0 ~ 141.0 ~ 146.0
Pow=0.727 TPos=134.415
Kurt=-0.422 Skew=0.228
K-S test for normality: 0

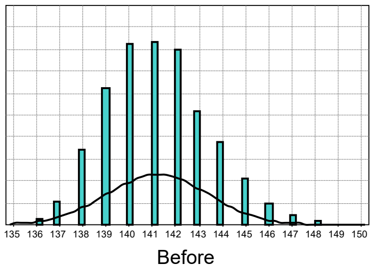

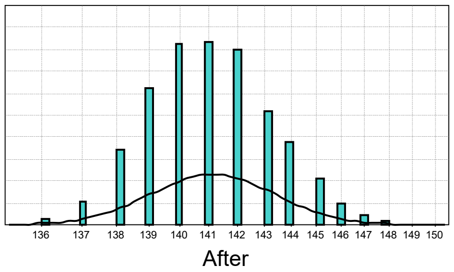

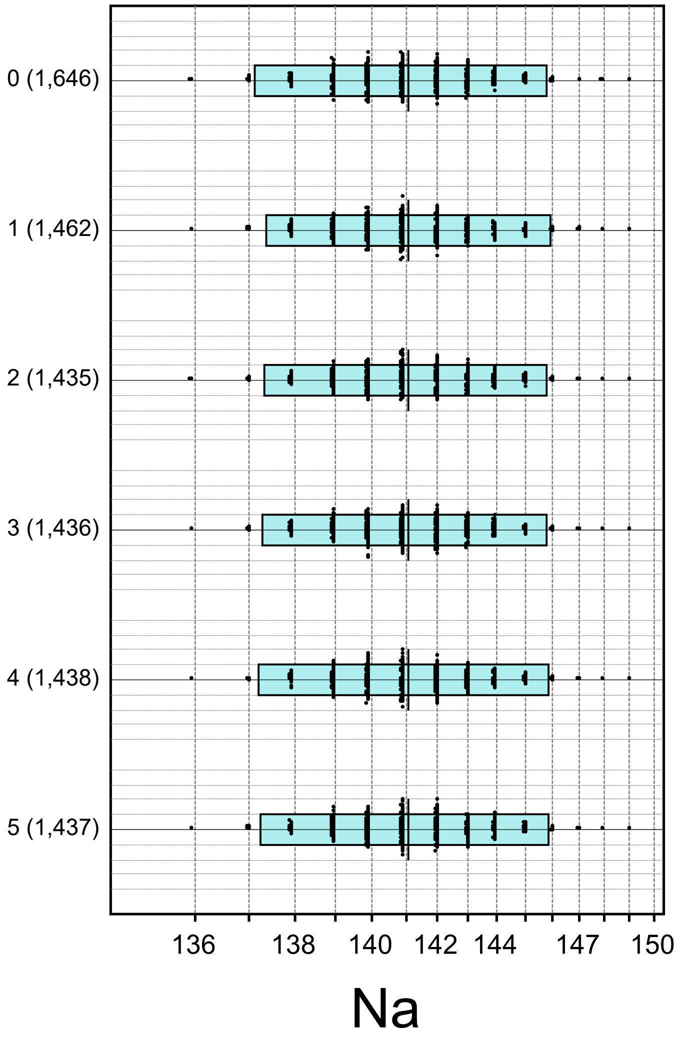

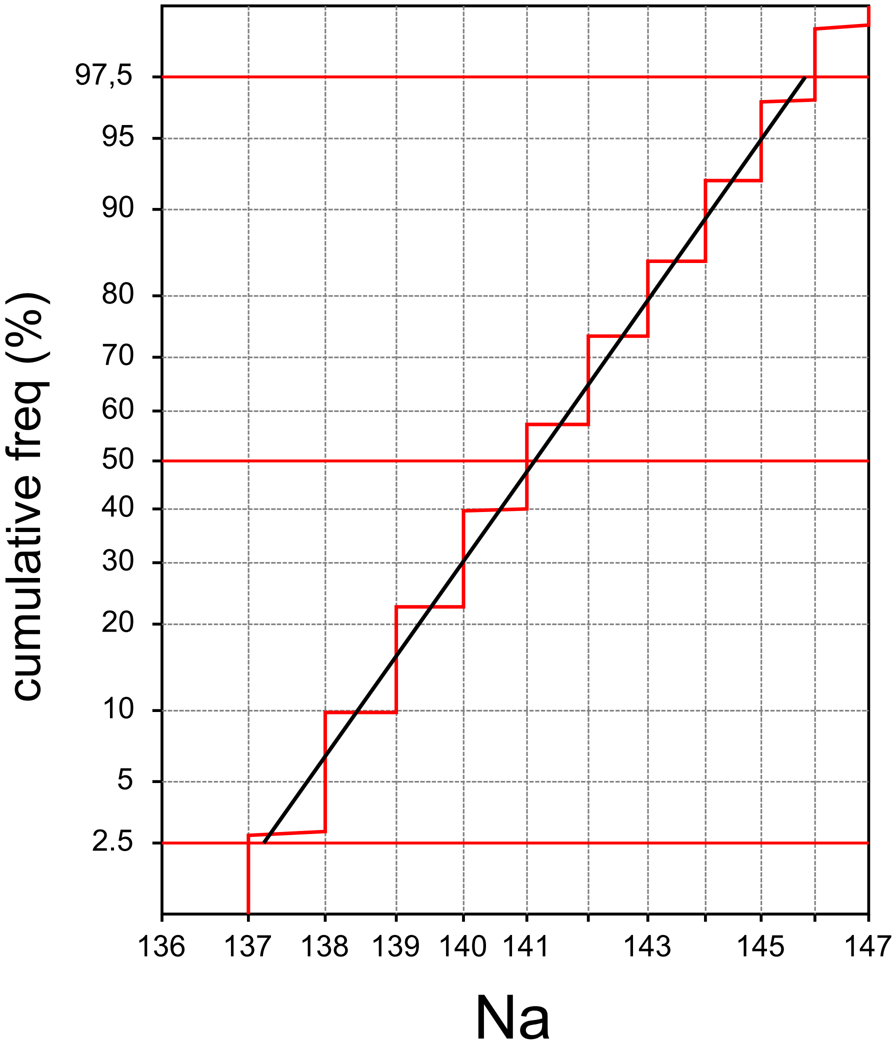

Na M n=668
Para: 137.7 ~ 141.3 ~ 146.0
Nonpara: 137.5 ~ 141.0 ~ 146.0
Pow=0.669 TPos=135.401
Kurt=-0.422 Skew=0.214
K-S test for normality: 0

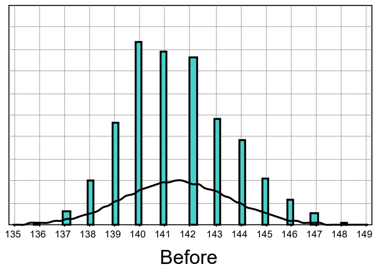

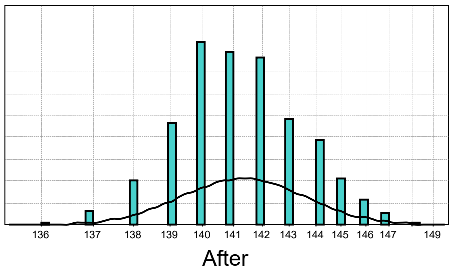

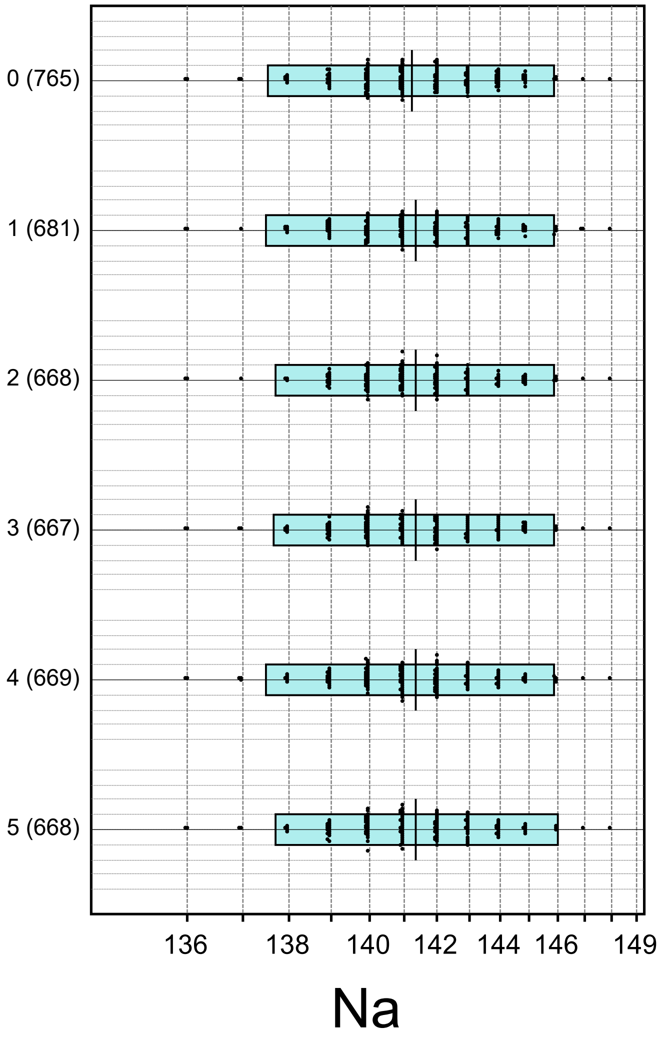

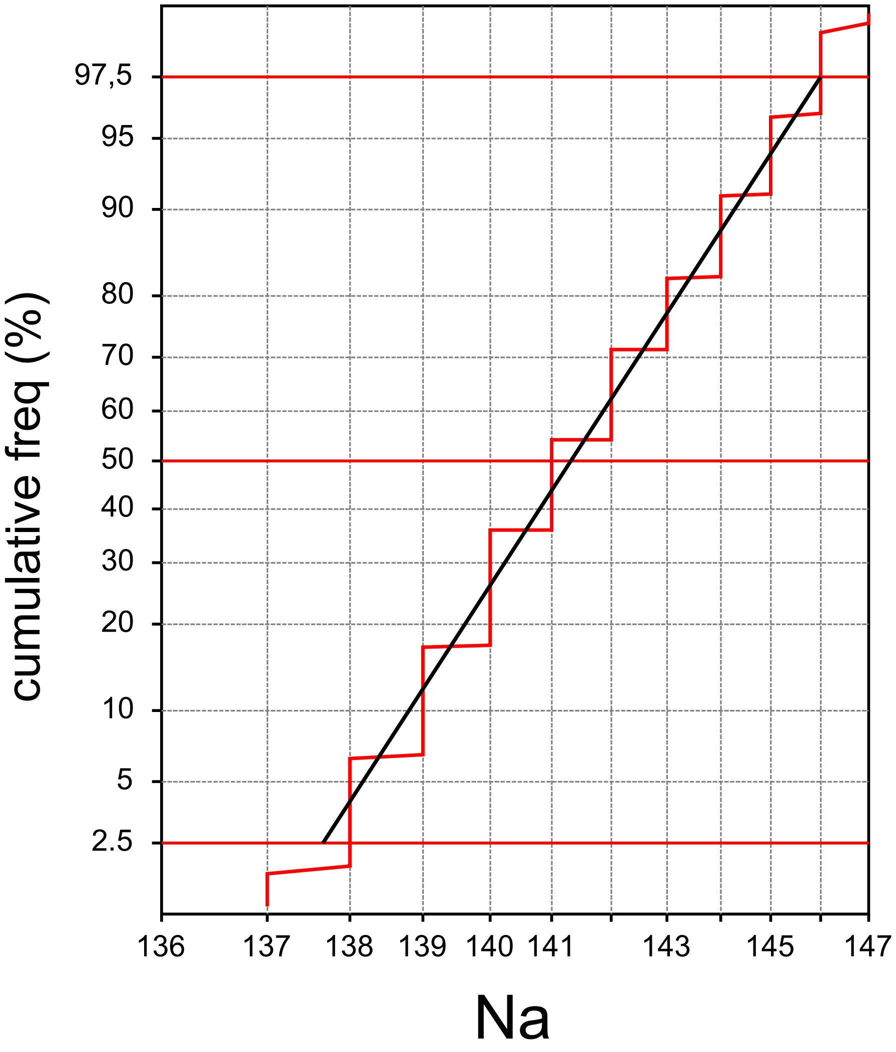

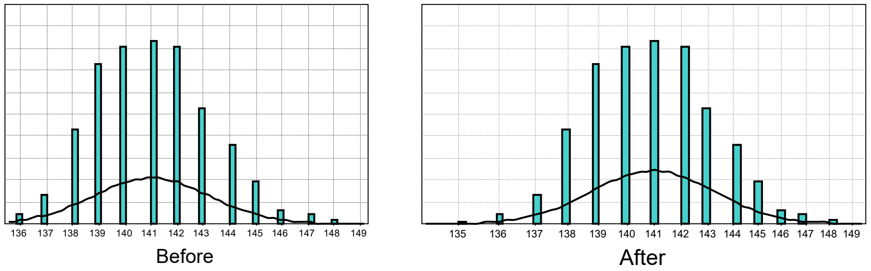

Na F n=776
Para: 136.9 ~ 140.9 ~ 145.7
Nonpara: 137.0 ~ 141.0 ~ 145.7
Pow=0.744 TPos=133.929
Kurt=-0.306 Skew=-0.109
K-S test for normality: 0

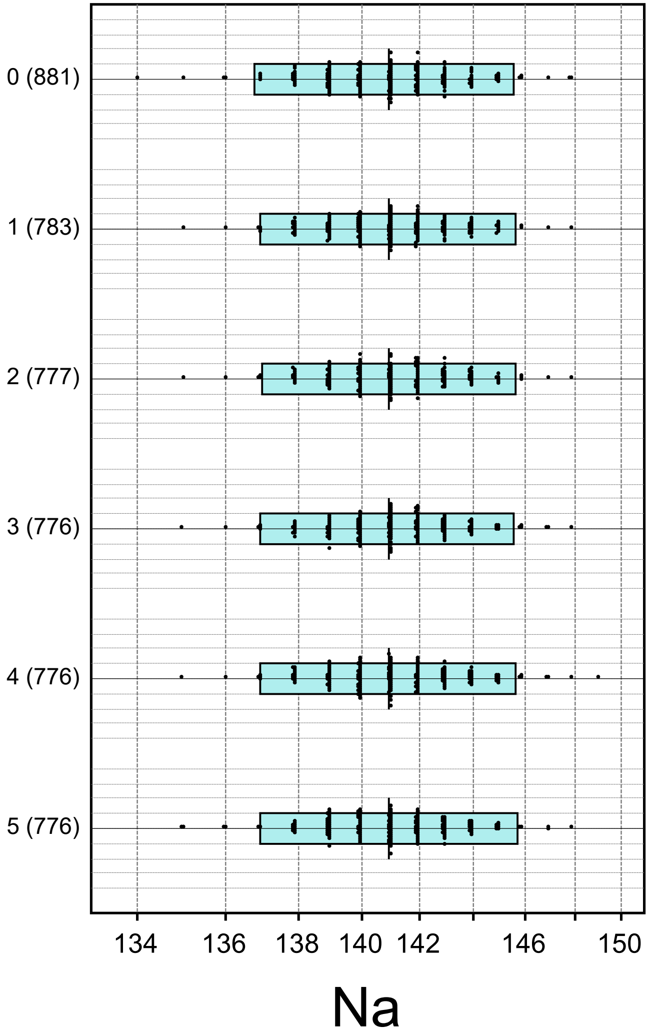

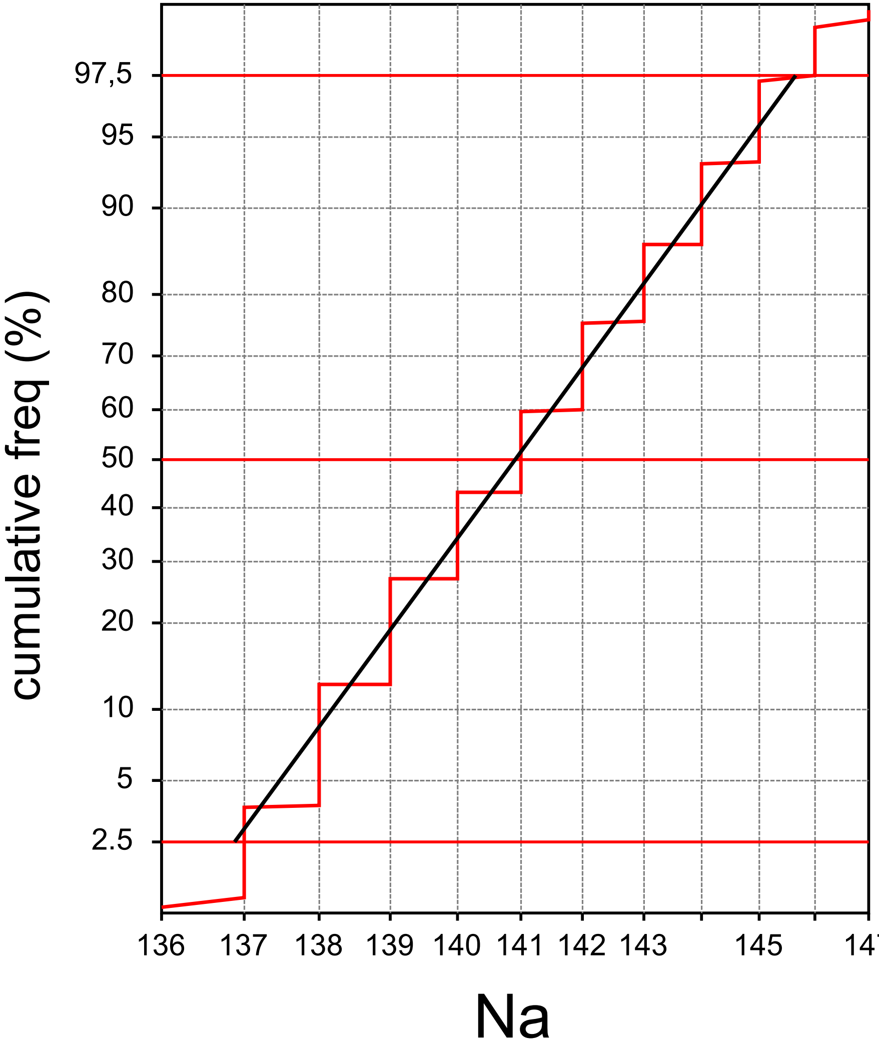

CI MF n=1,441
Para: 108.2 ~ 112.7 ~ 118.0
Nonpara: 108.0 ~ 113.0 ~ 118.0
Pow=0.761 TPos=104.853
Kurt=-0.284 Skew=-0.06
K-S test for normality: 0

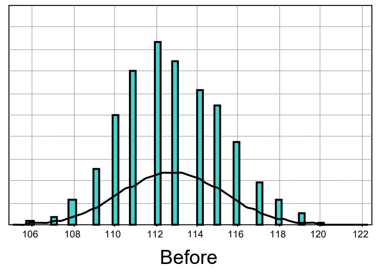

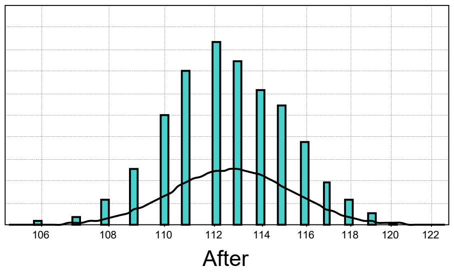

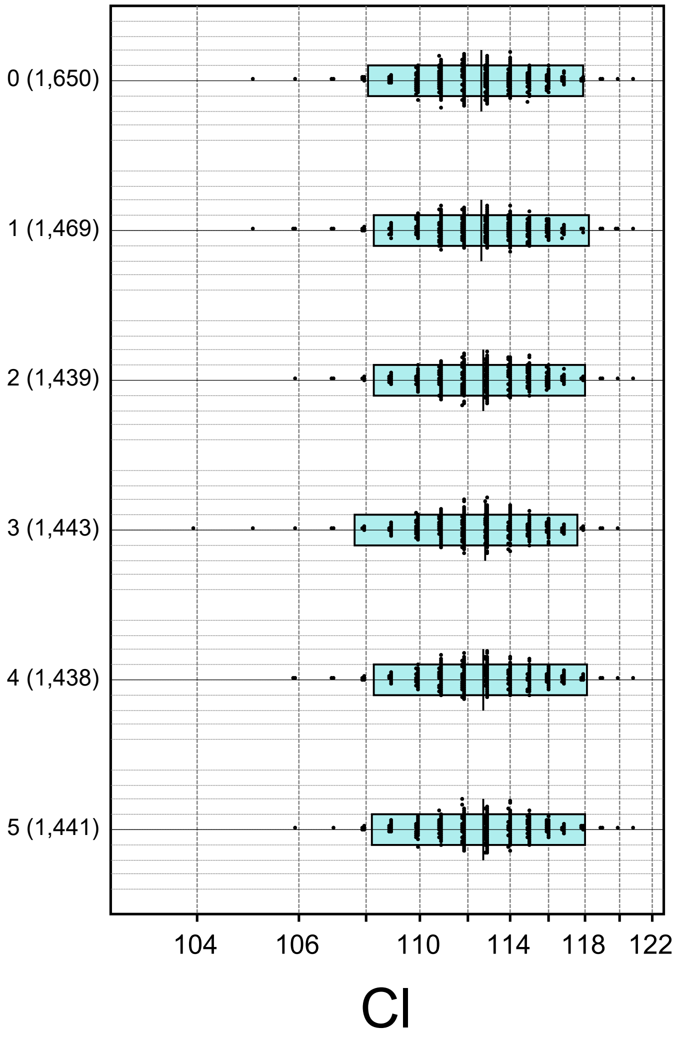

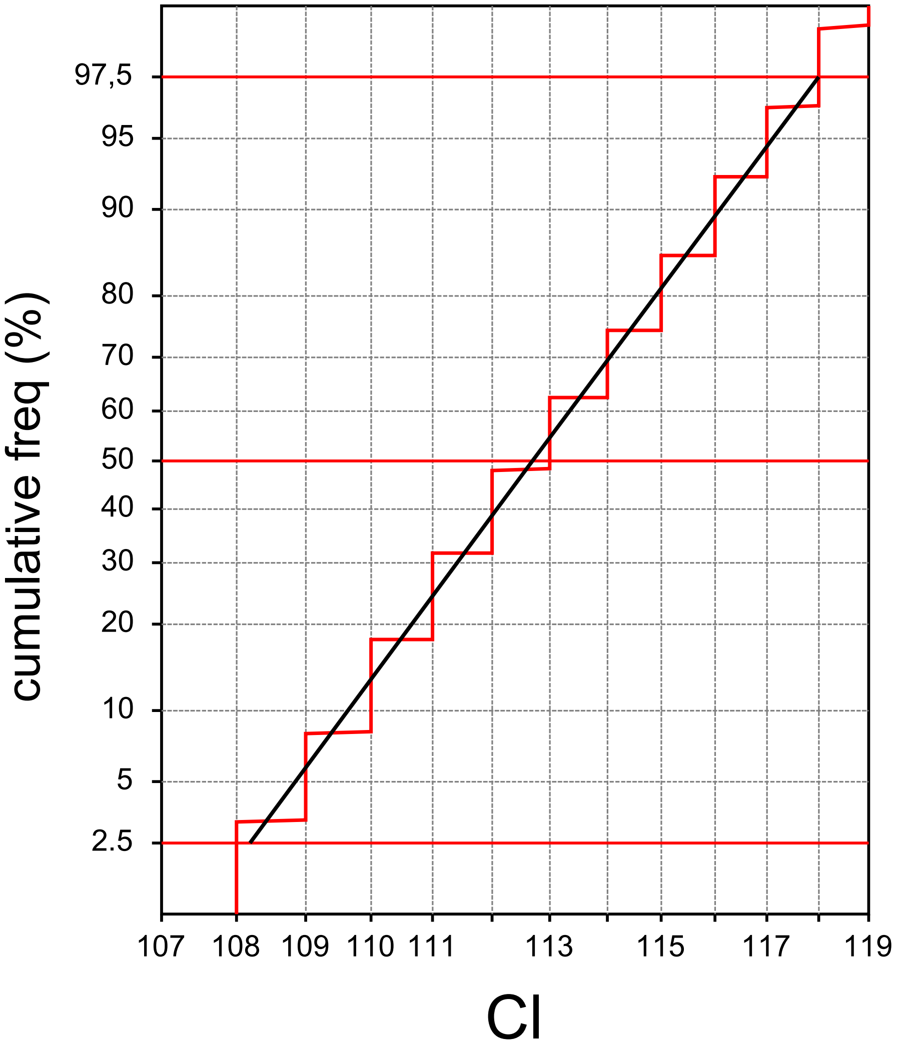

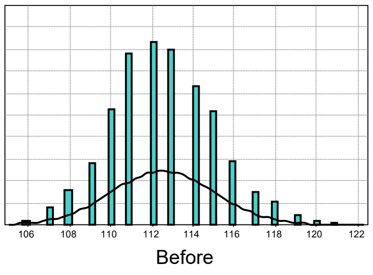

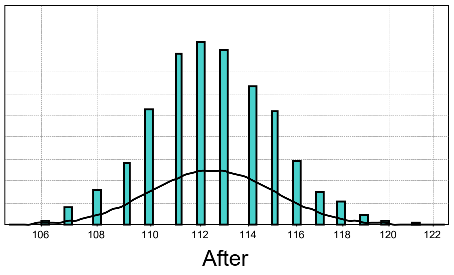

CI M n=670
Para: 107.7 ~ 112.5 ~ 117.6
Nonpara: 107.5 ~ 112.3 ~ 117.9
Pow=0.879 TPos=104.135
Kurt=-0.314 Skew=0.181
K-S test for normality: 0.00005

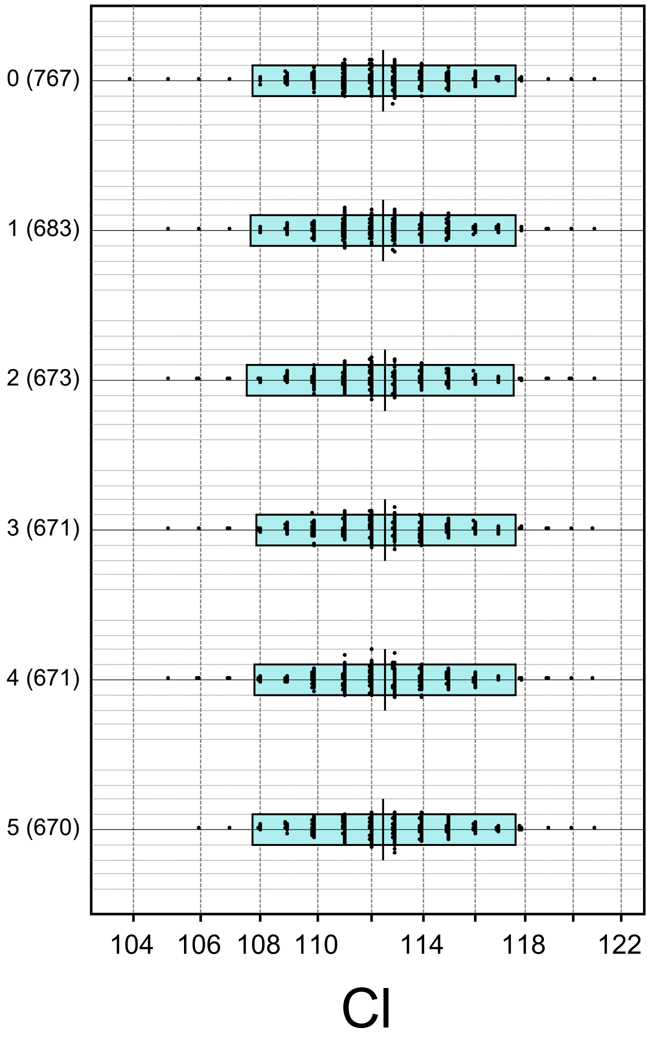

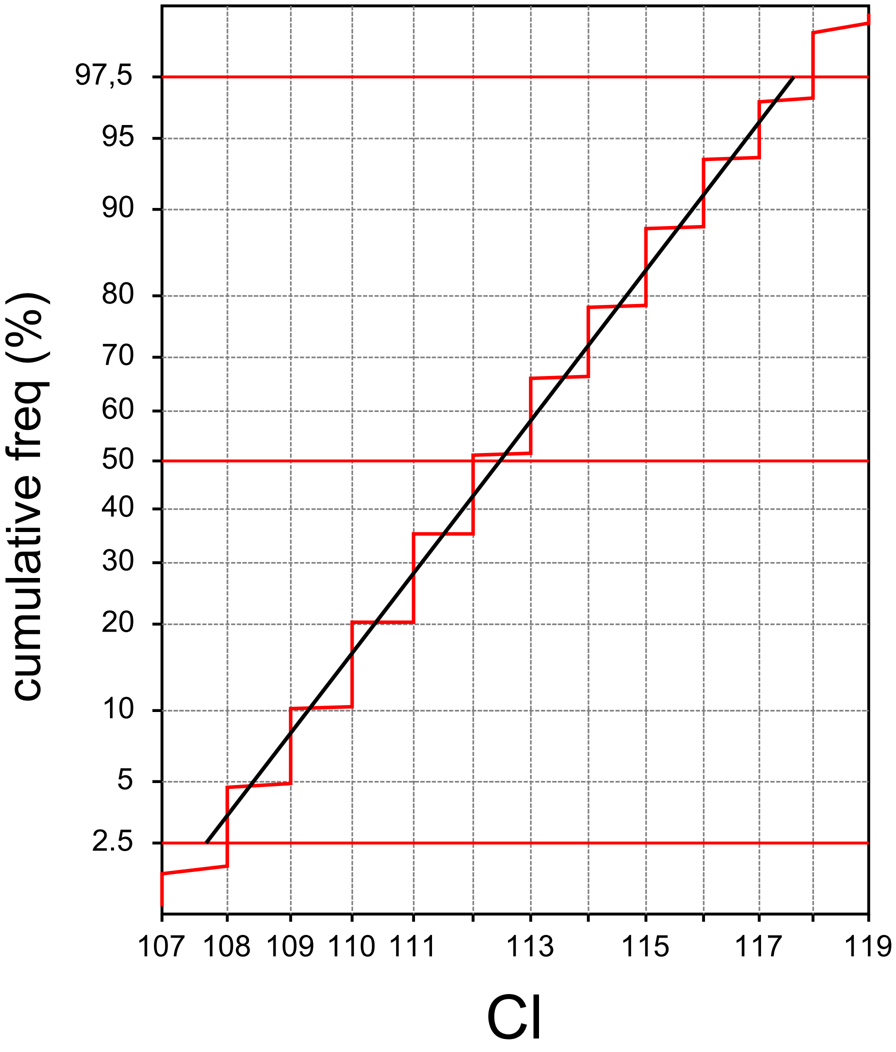

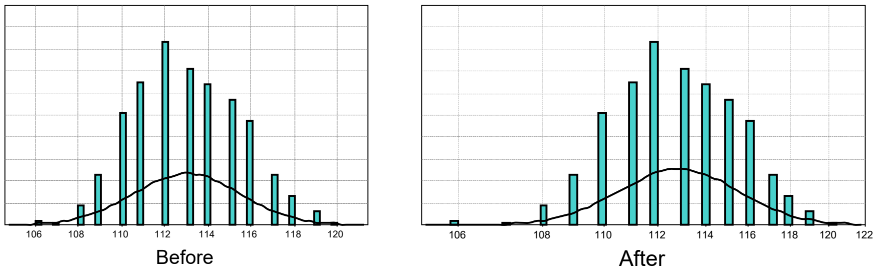

Cl F n=778
Para: 108.6 ~ 112.9 ~ 118.3
Nonpara: 108.7 ~ 113.0 ~ 117.9
Pow=0.635 TPos=105.217
Kurt=-0.467 Skew=-0.093
K-S test for normality: 0.00001

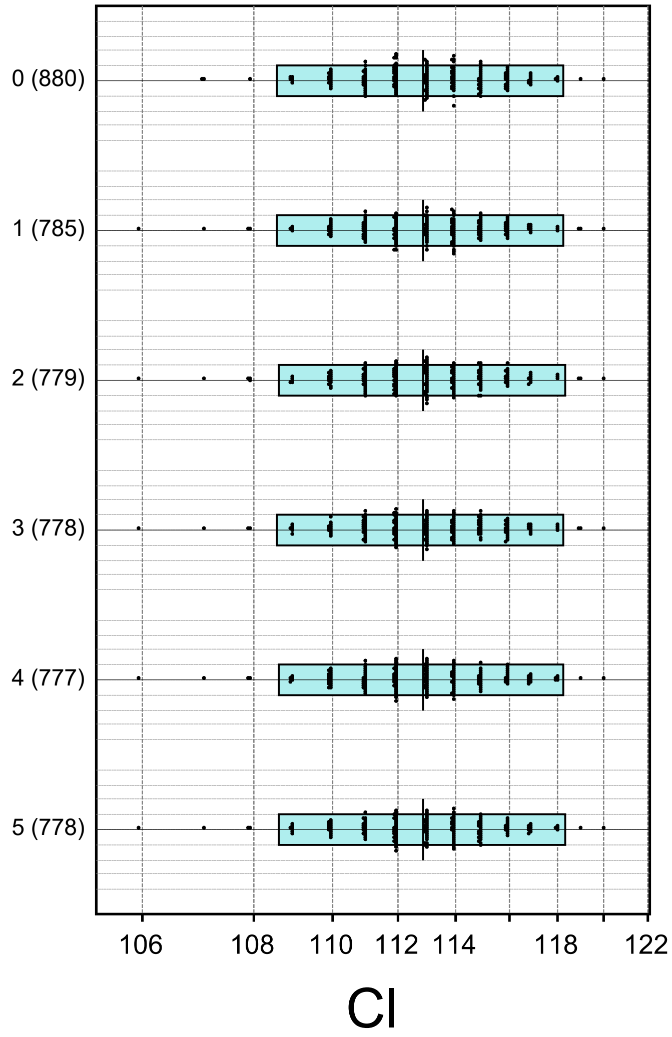

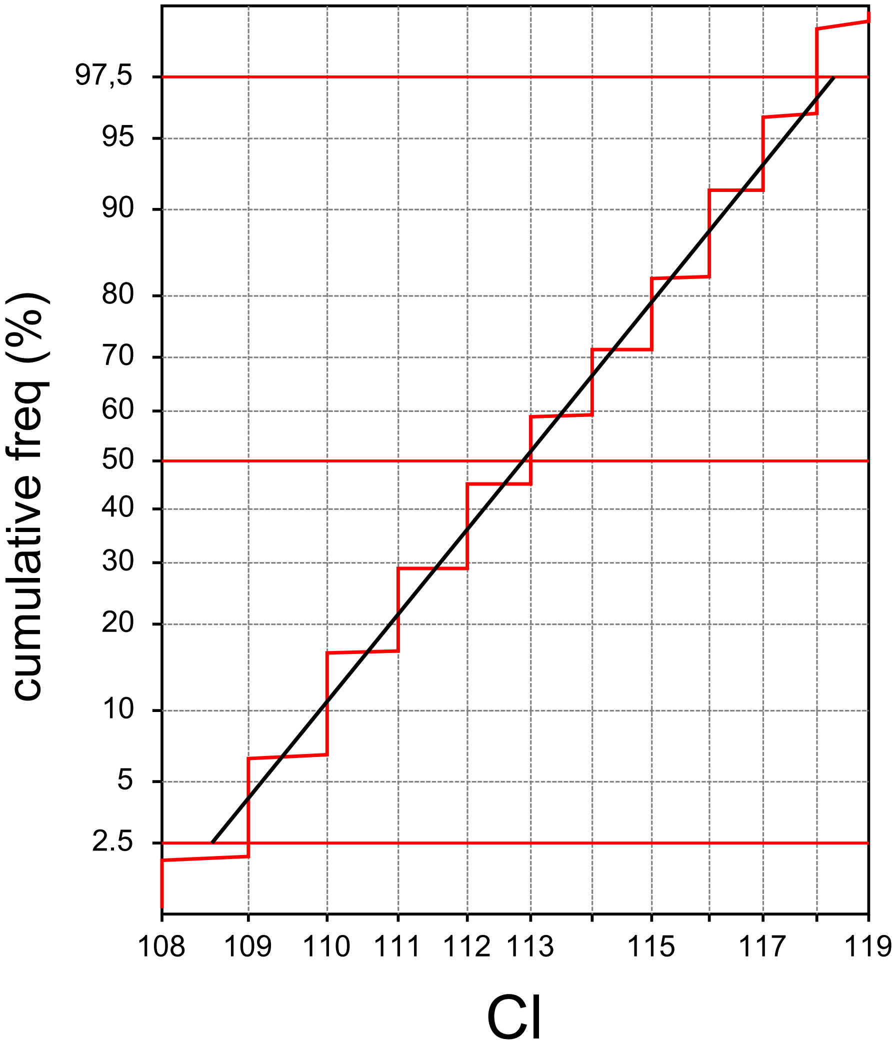

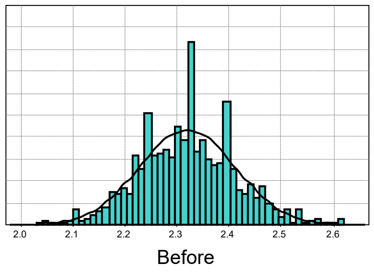

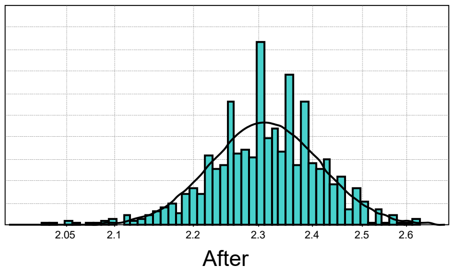

Ca MF n=1,435
Para: 2.151 ~ 2.314 ~ 2.523
Nonpara: 2.116 ~ 2.319 ~ 2.505
Pow=0.515 TPos=1.958
Kurt=-0.179 Skew=-0.116
K-S test for normality: 0.0006

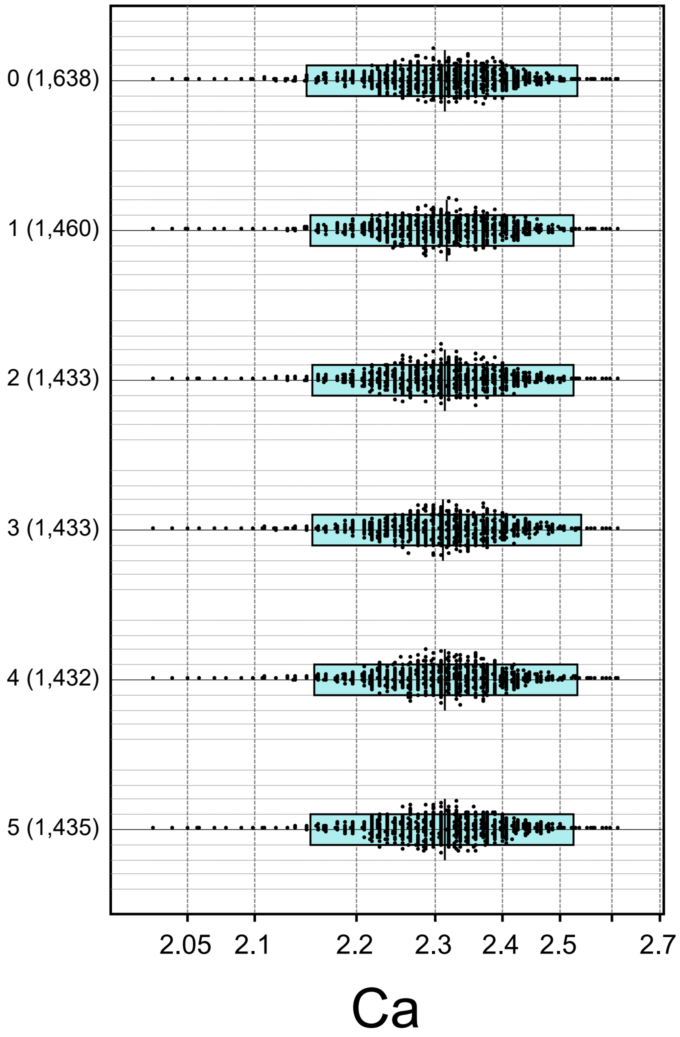

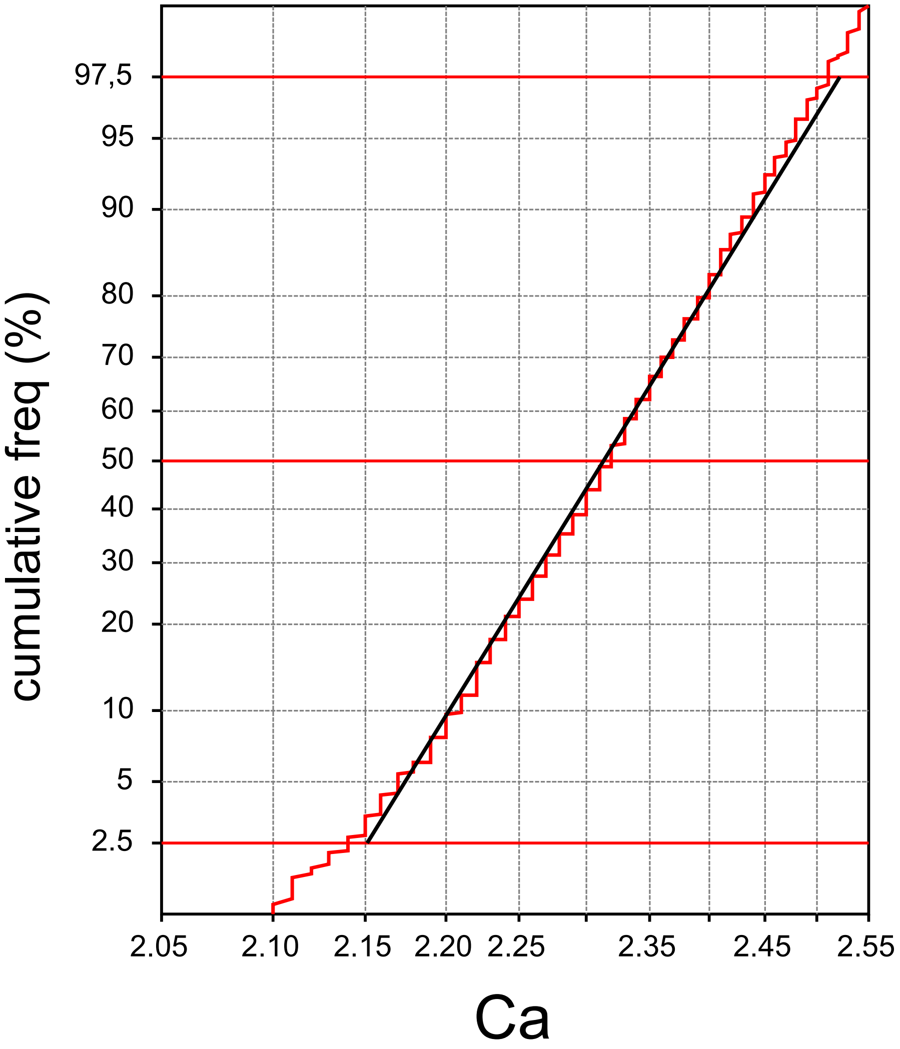

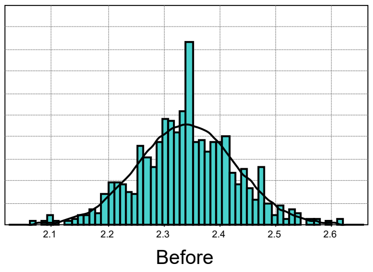

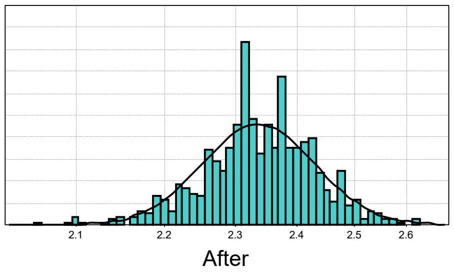

Ca M n=669
Para: 2.178 ~ 2.336 ~ 2.528
Nonpara: 2.154 ~ 2.337 ~ 2.519
Pow=0.631 TPos=2.017
Kurt=-0.189 Skew=-0.047
K-S test for normality: 0.03291

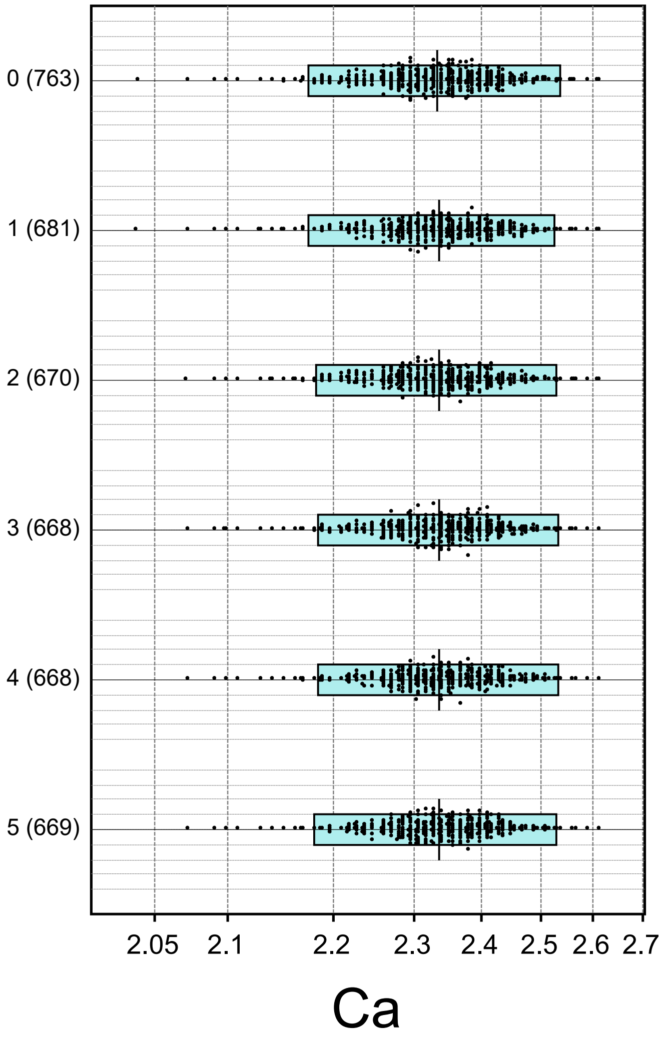

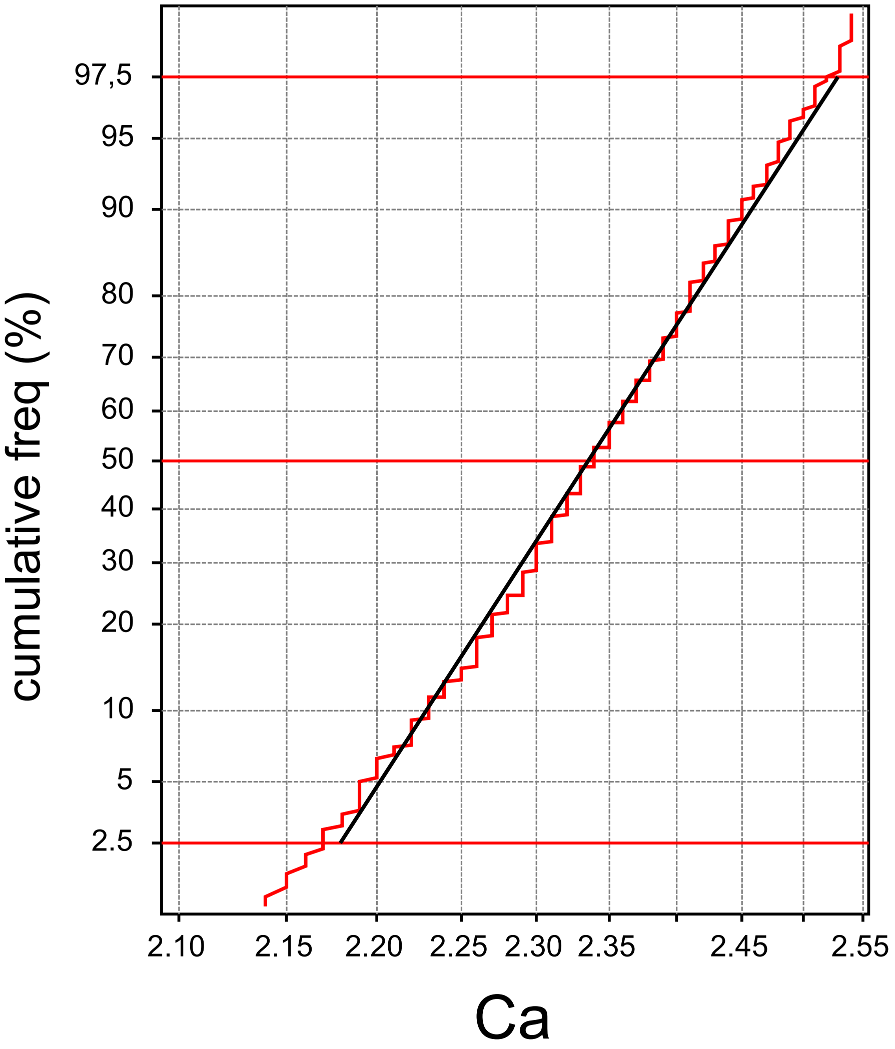

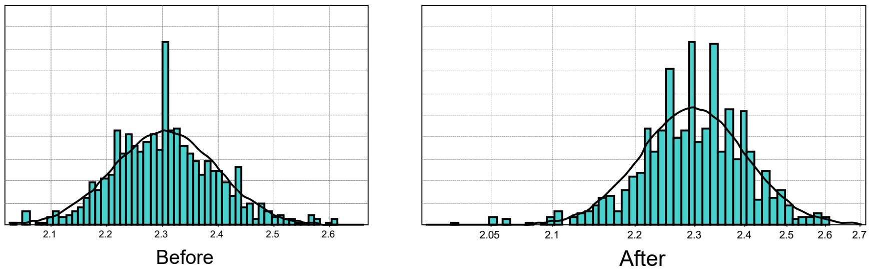

Ca F n=773
Para: 2.146 ~ 2.296 ~ 2.521
Nonpara: 2.106 ~ 2.300 ~ 2.504
Pow=0.373 TPos=1.986
Kurt=-0.107 Skew=-0.158
K-S test for normality: 0.02964

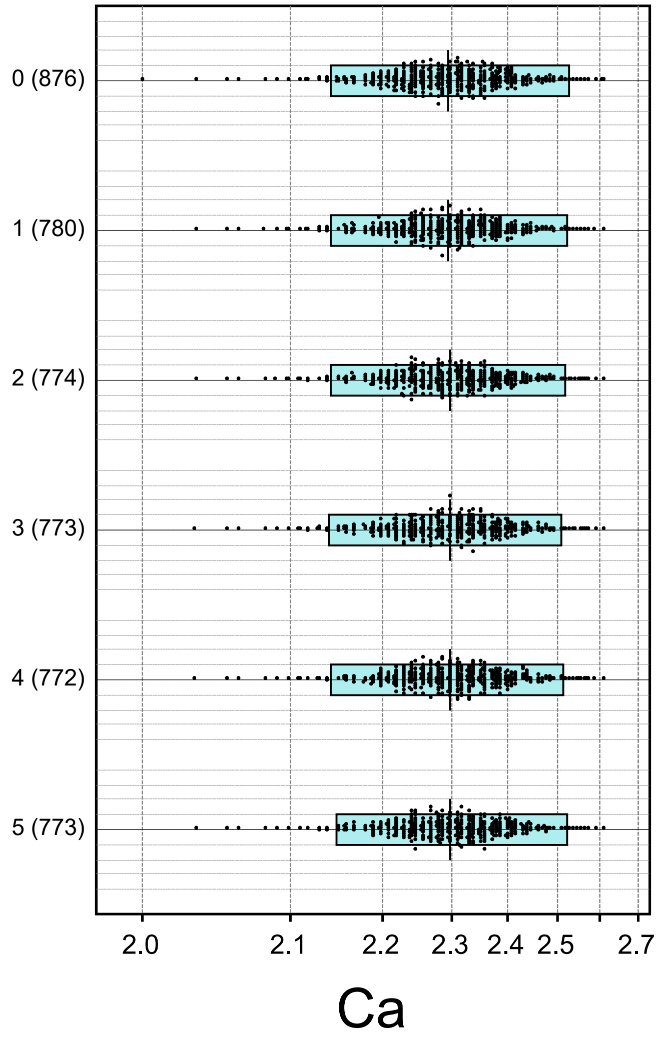

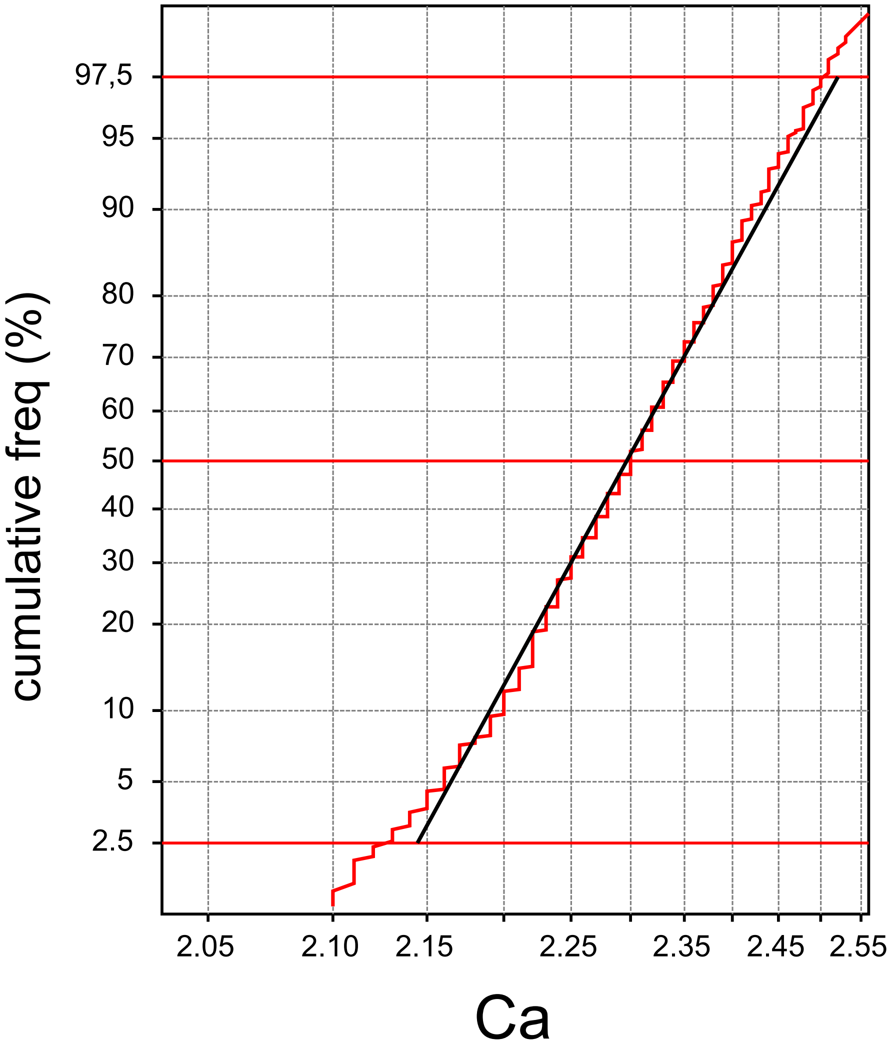

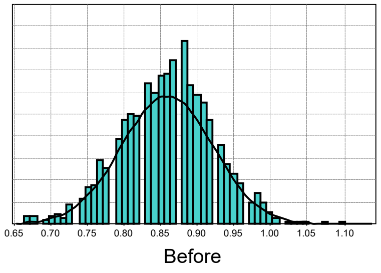

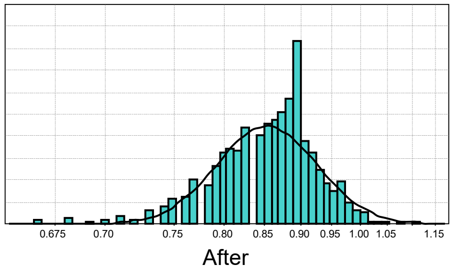

Mg MF n=1,432
Para: 0.749 ~ 0.854 ~ 1.009
Nonpara: 0.719 ~ 0.860 ~ 0.980
Pow=0.419 TPos=0.649
Kurt=-0.132 Skew=-0.395
K-S test for normality: 0

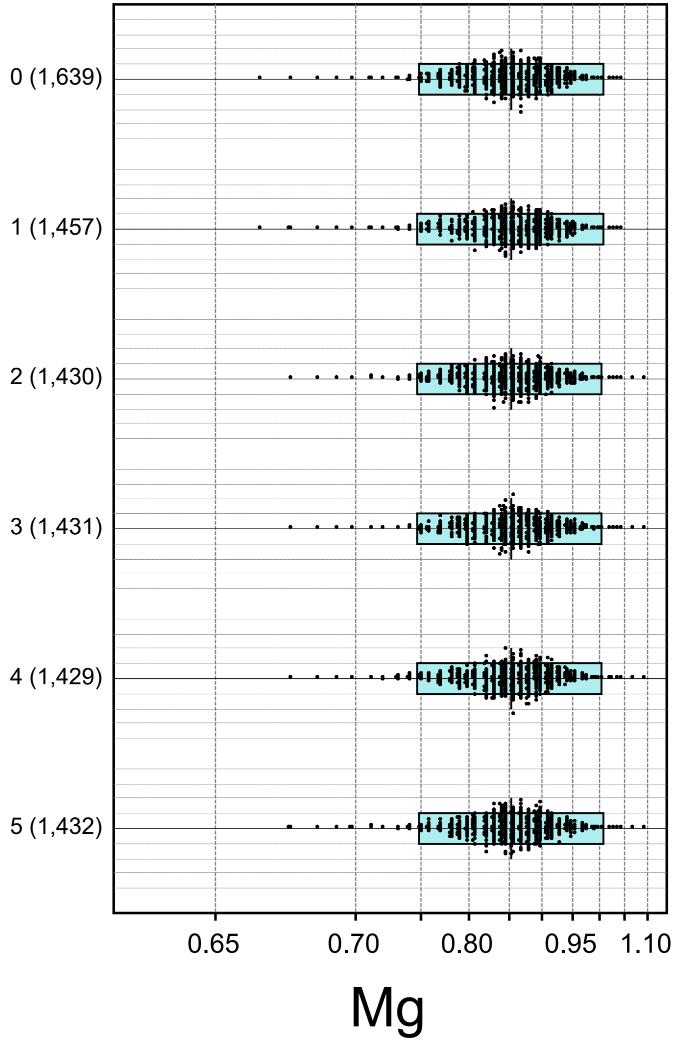

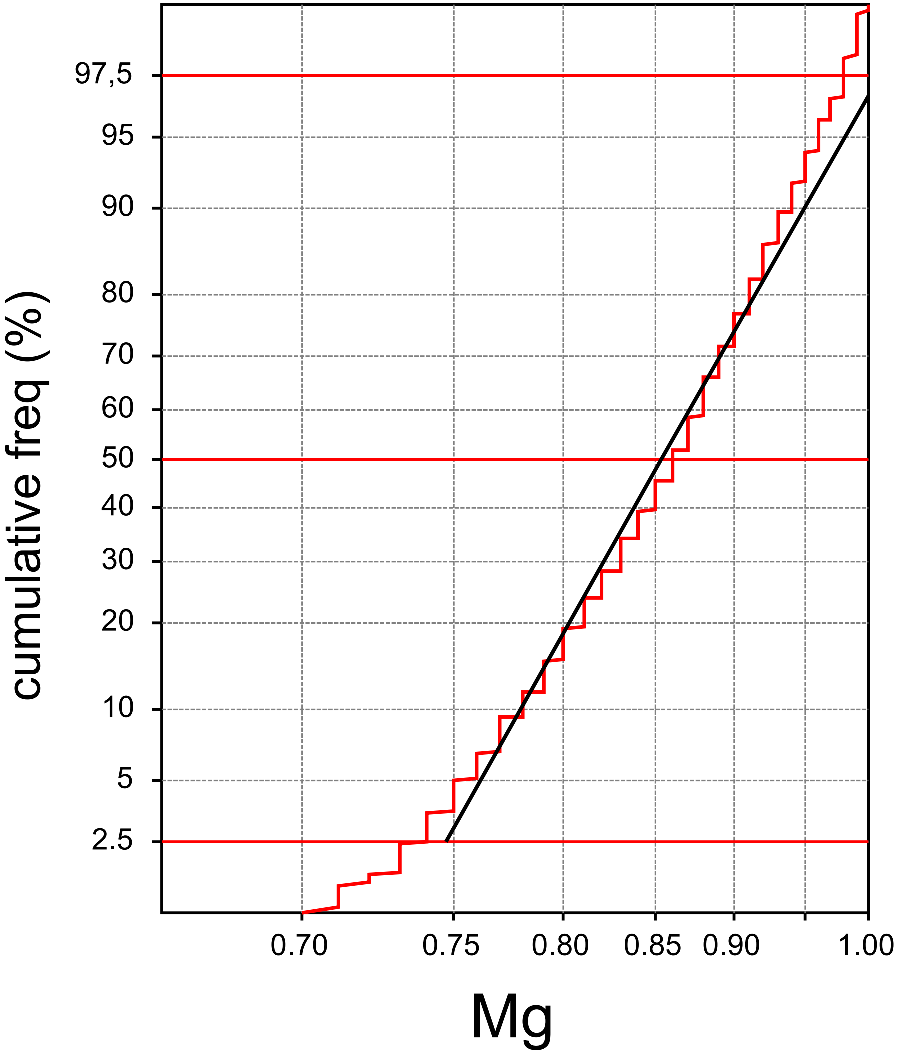

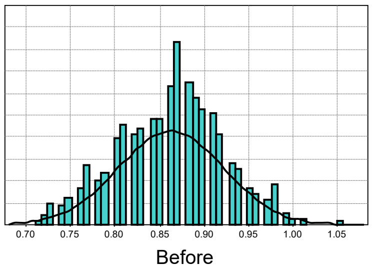

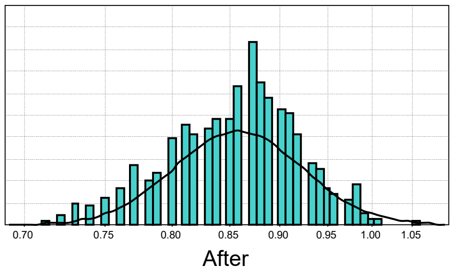

Mg M n=659
Para: 0.748 ~ 0.857 ~ 1.004
Nonpara: 0.722 ~ 0.867 ~ 0.978
Pow=0.488 TPos=0.617
Kurt=-0.029 Skew=-0.451
K-S test for normality: 0.00004

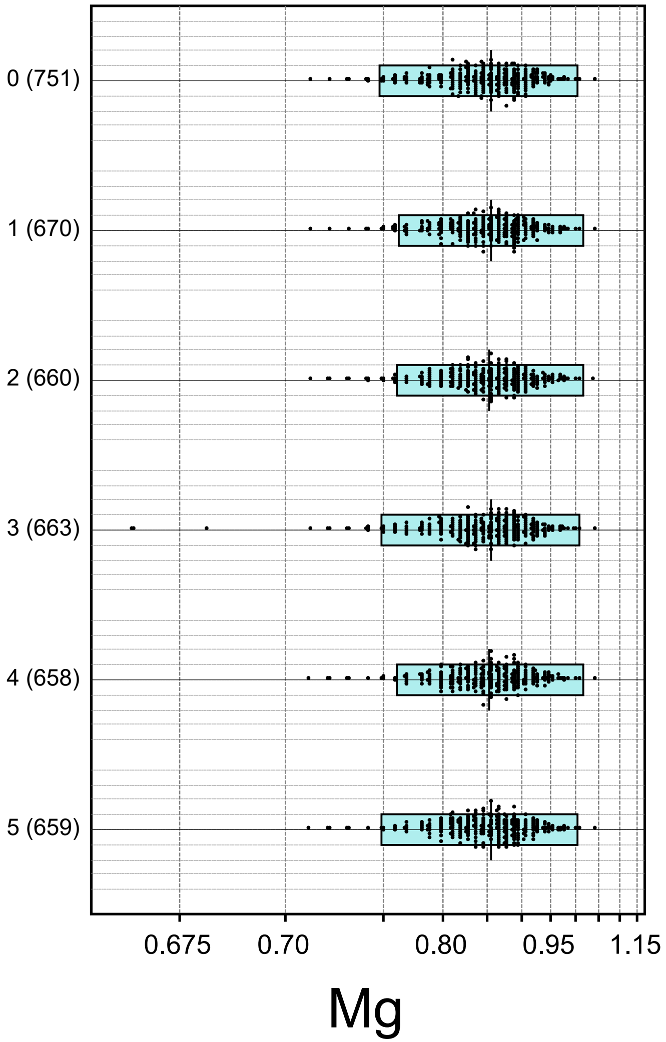

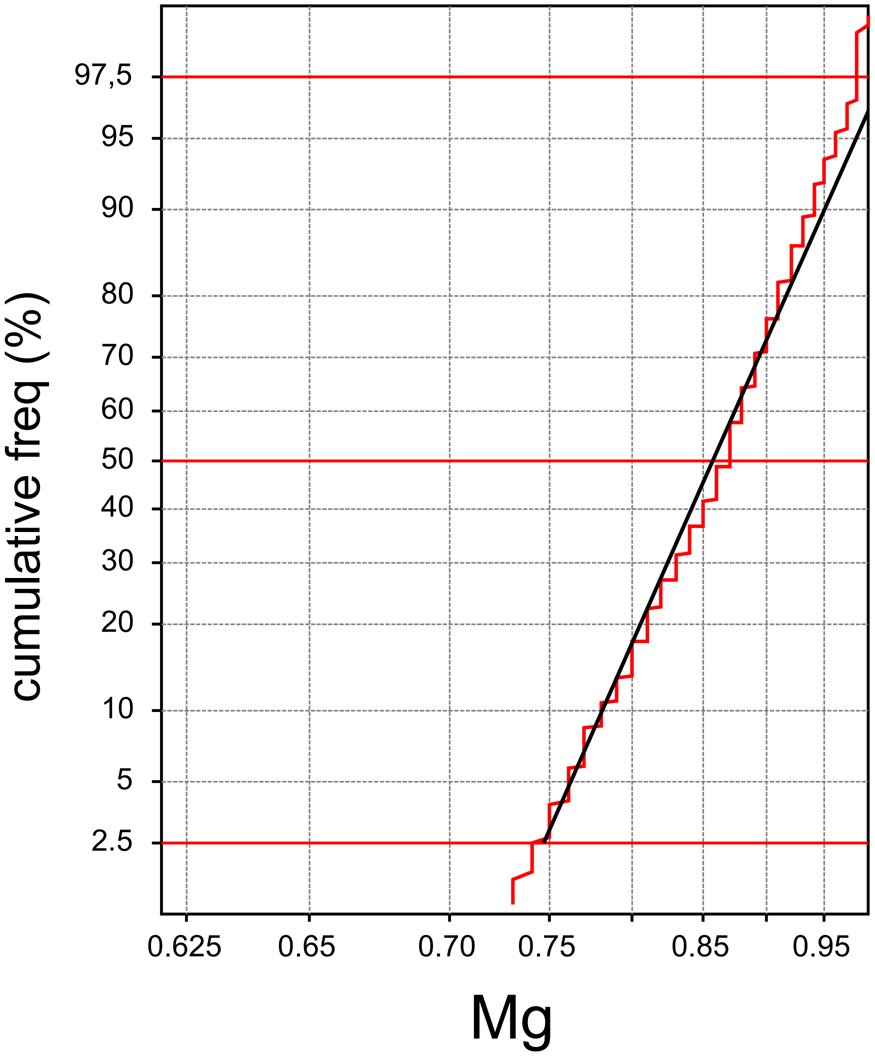

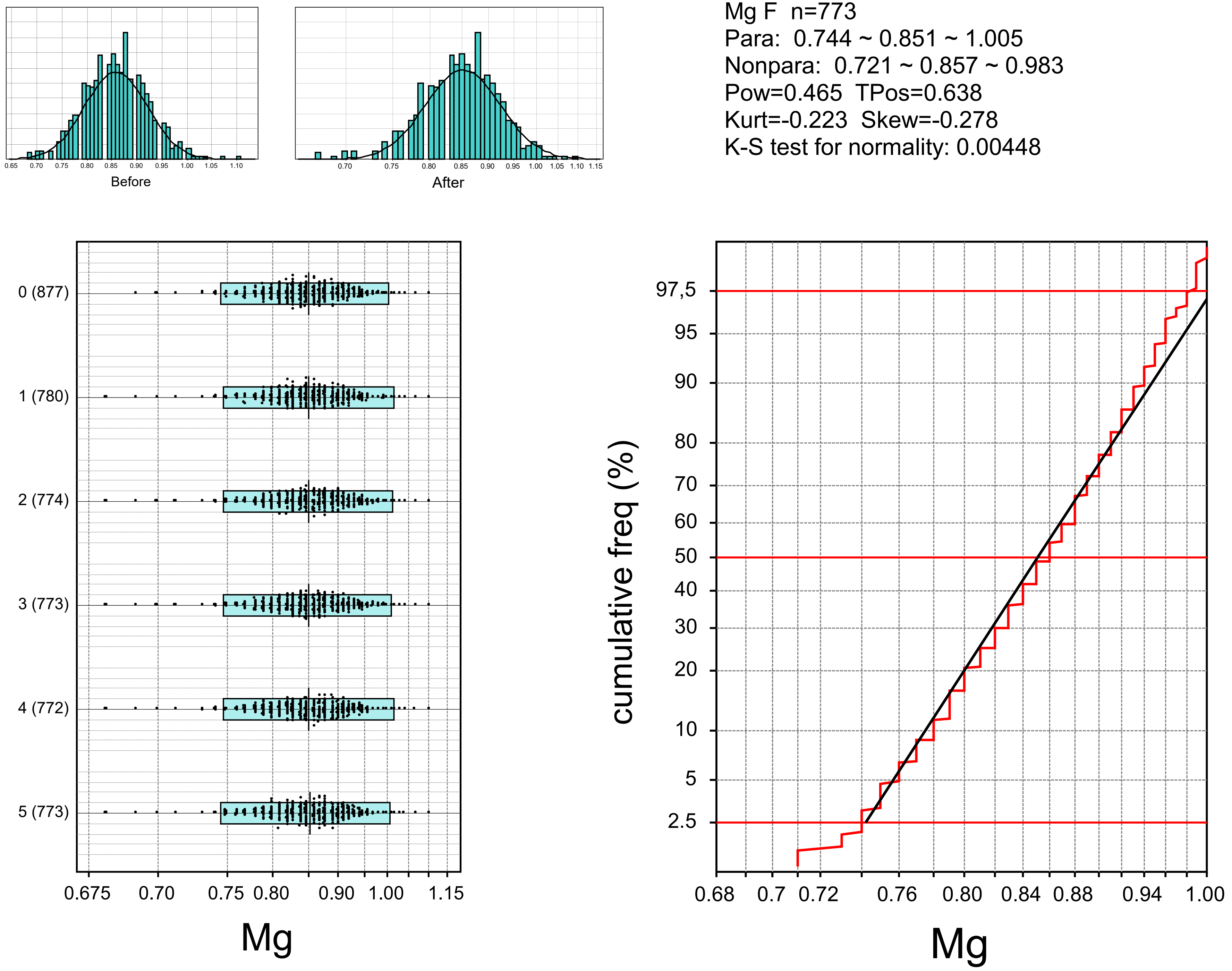

Mg F n=773
Para: 0.744 ~ 0.851 ~ 1.005
Nonpara: 0.721 ~ 0.857 ~ 0.983
Pow=0.465 TPos=0.638
Kurt=-0.223 Skew=-0.278
K-S test for normality: 0.00448

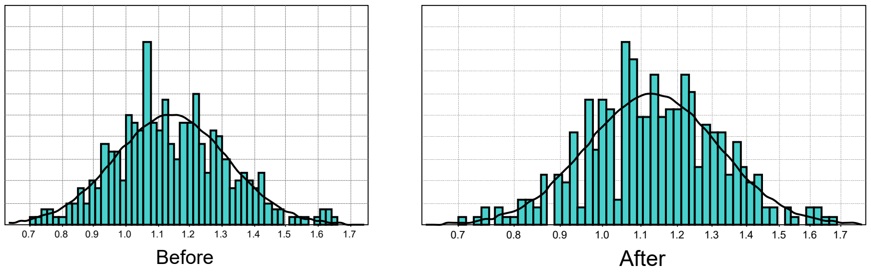

P MF n=351
Para: 0.822 ~ 1.130 ~ 1.507
Nonpara: 0.807 ~ 1.129 ~ 1.526
Pow=0.725 TPos=0.621
Kurt=-0.185 Skew=0.034
K-S test for normality: P≈1.00 (N.S.)

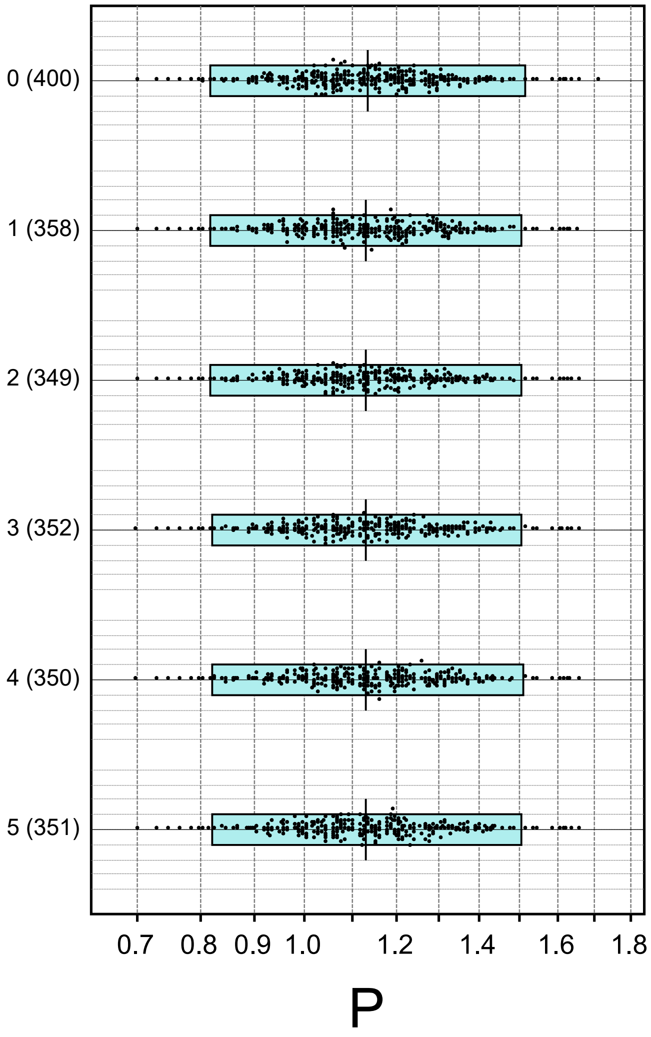

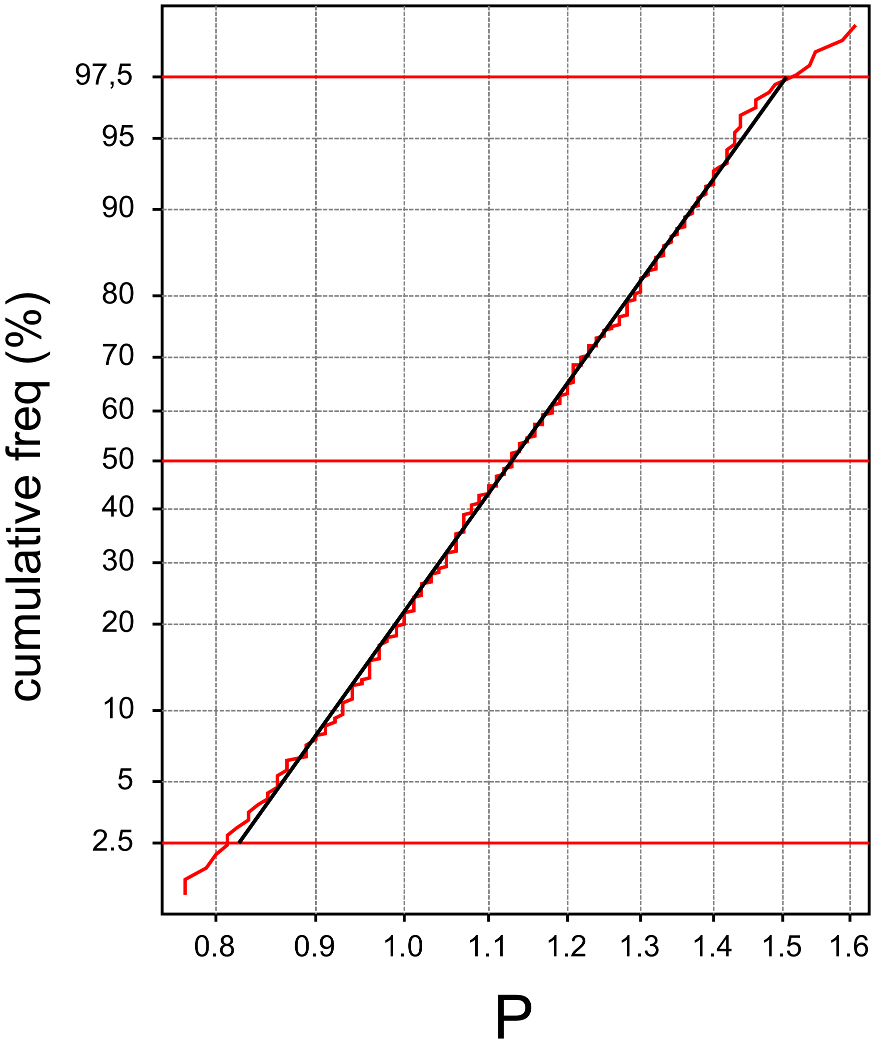

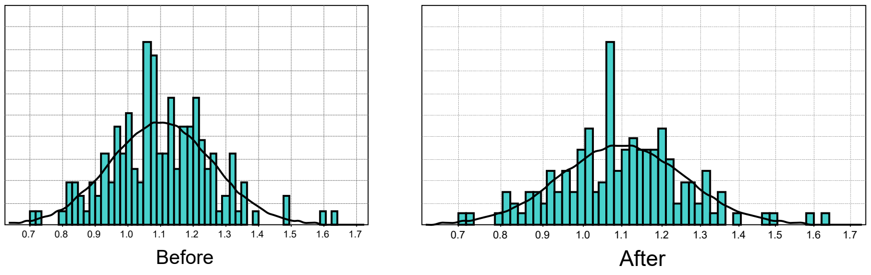

P M n=155
Para: 0.805 ~ 1.098 ~ 1.415
Nonpara: 0.807 ~ 1.096 ~ 1.455
Pow=0.927 TPos=0.577
Kurt=-0.327 Skew=-0.105
K-S test for normality: P≈1.00 (N.S.)

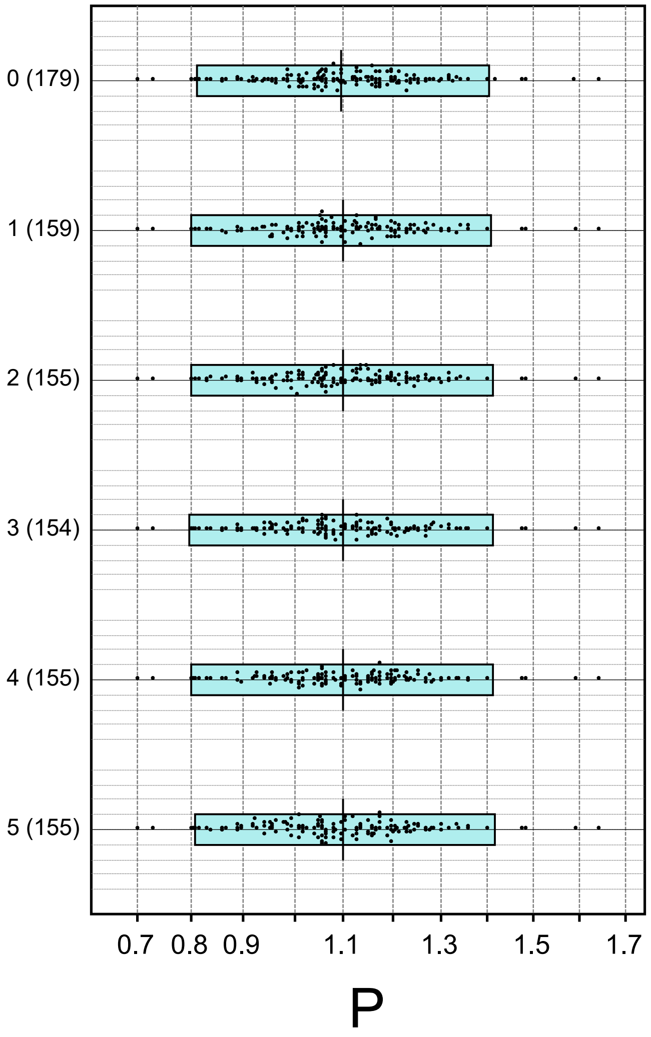

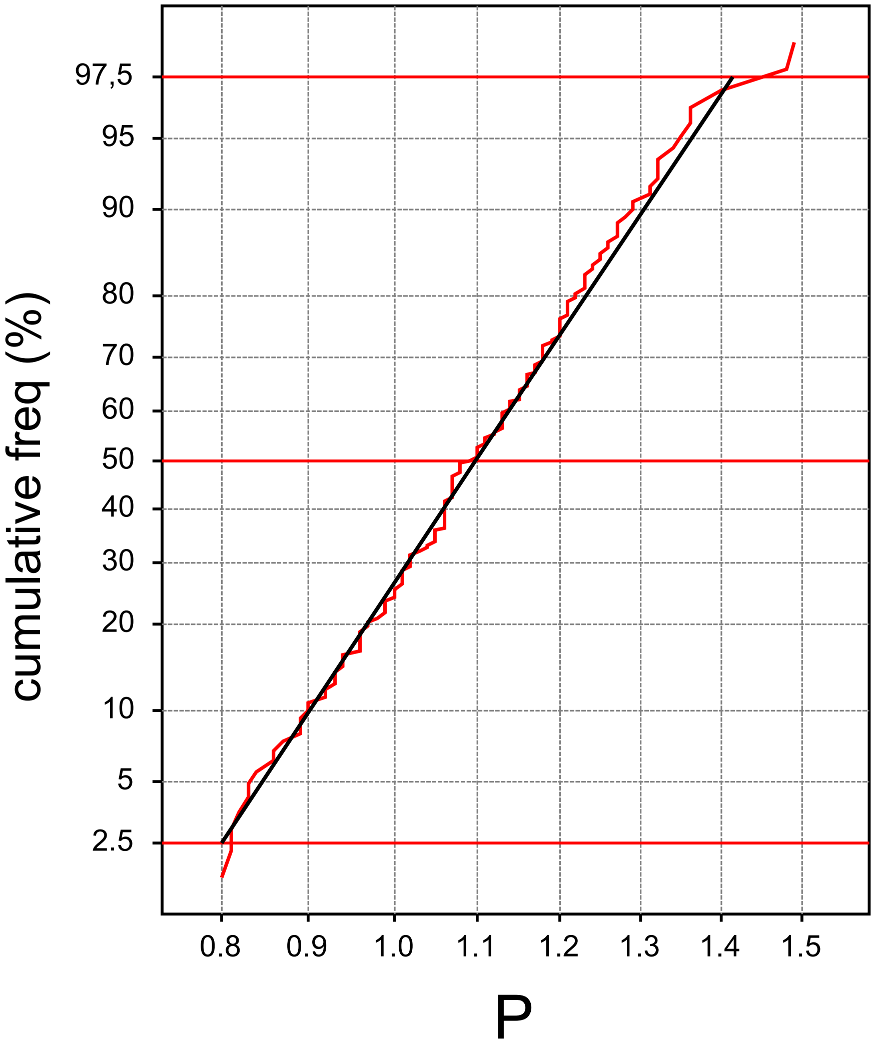

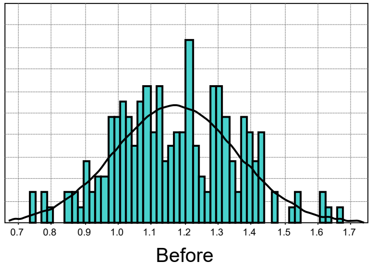

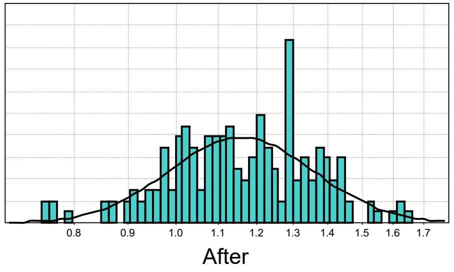

P F n=194
Para: 0.842 ~ 1.160 ~ 1.575
Nonpara: 0.820 ~ 1.165 ~ 1.567
Pow=0.656 TPos=0.658
Kurt=-0.206 Skew=-0.14
K-S test for normality: .559 (NS)

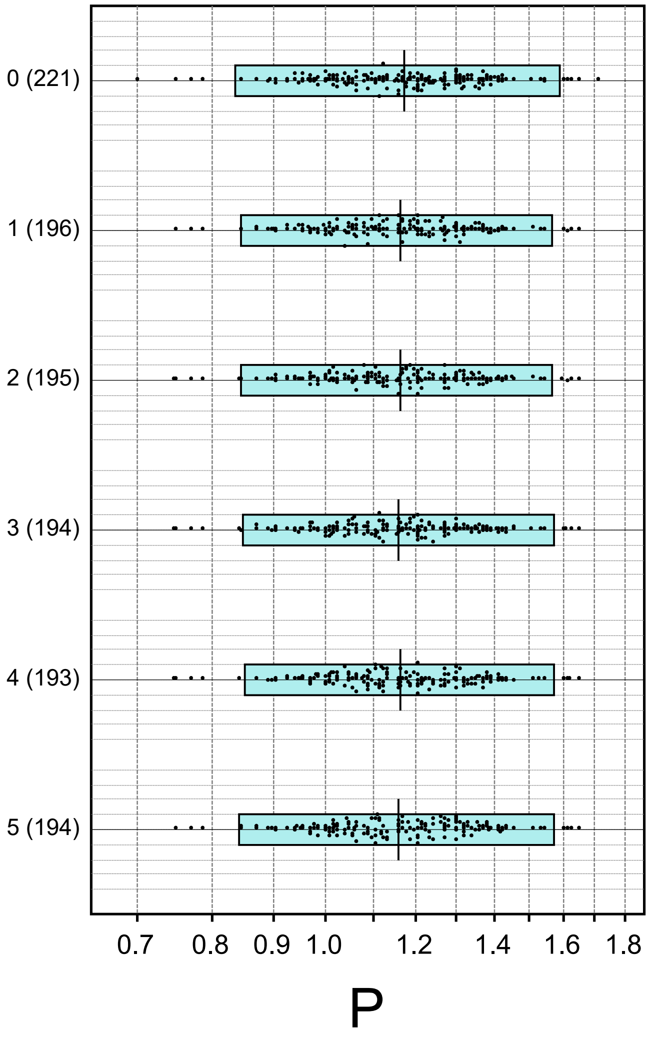

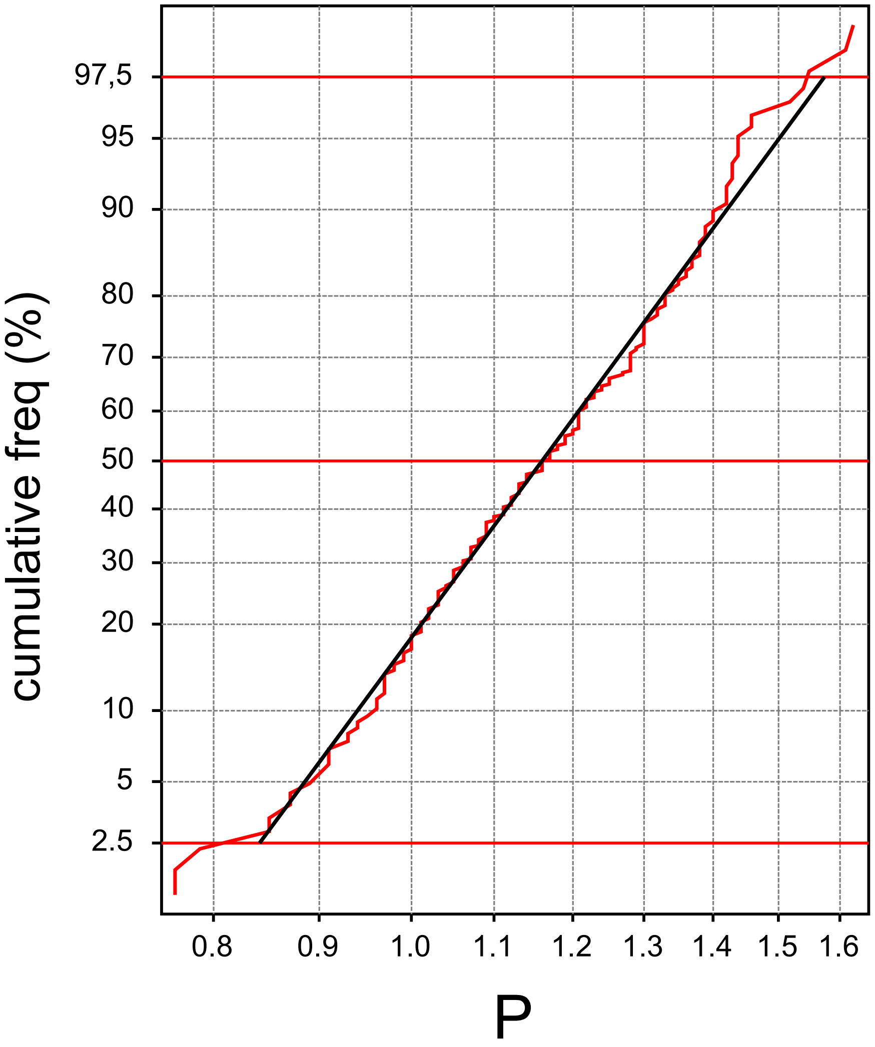

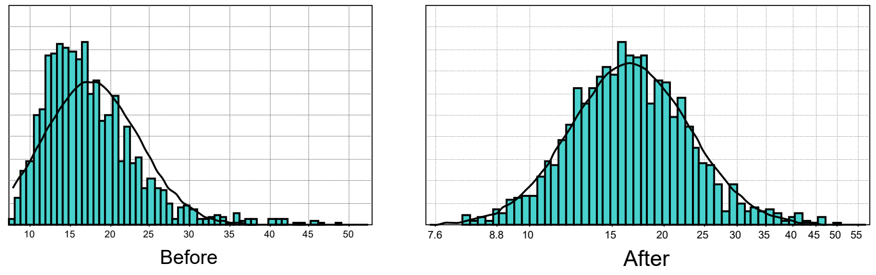

HCY MF n=1,413
Para: 9.31 ~ 16.53 ~ 31.60
Nonpara: 9.01 ~ 16.39 ~ 34.01
Pow=0.377 TPos=7.316
Kurt=-0.119 Skew=0.066
K-S test for normality: .571 (NS)

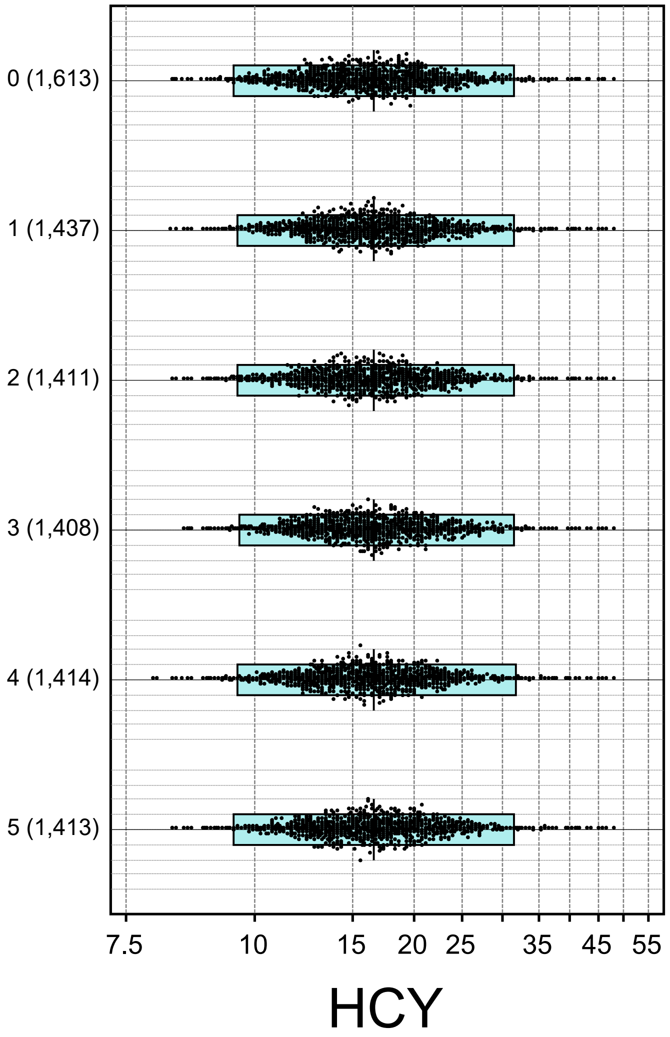

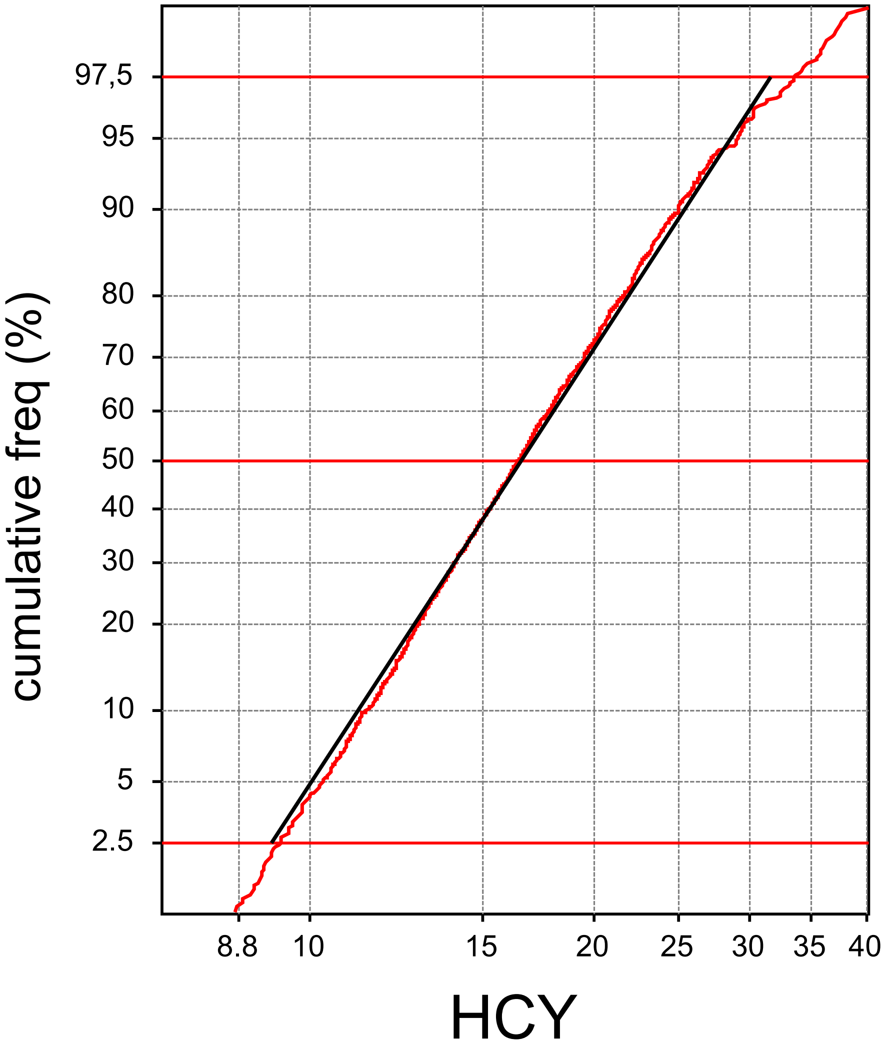

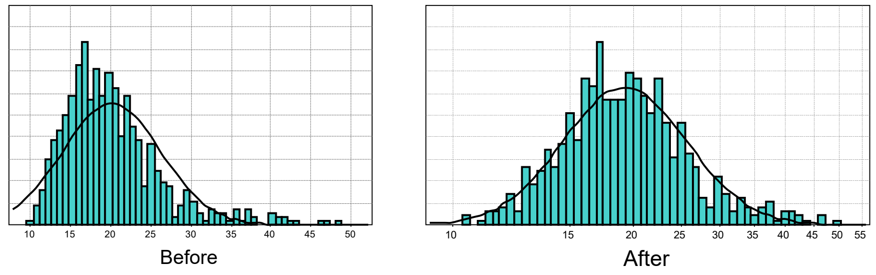

HCY M n=647
Para: 11.33 ~ 19.08 ~ 34.28
Nonpara: 10.93 ~ 18.85 ~ 37.49
Pow=0.392 TPos=8.766
Kurt=-0.104 Skew=0.15
K-S test for normality: .576 (NS)

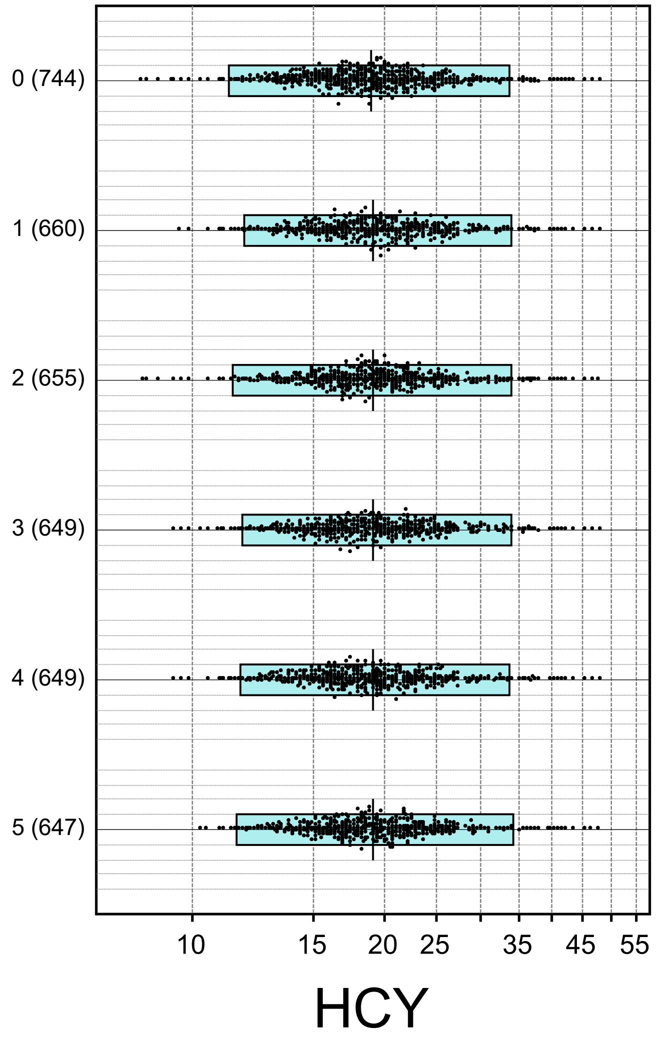

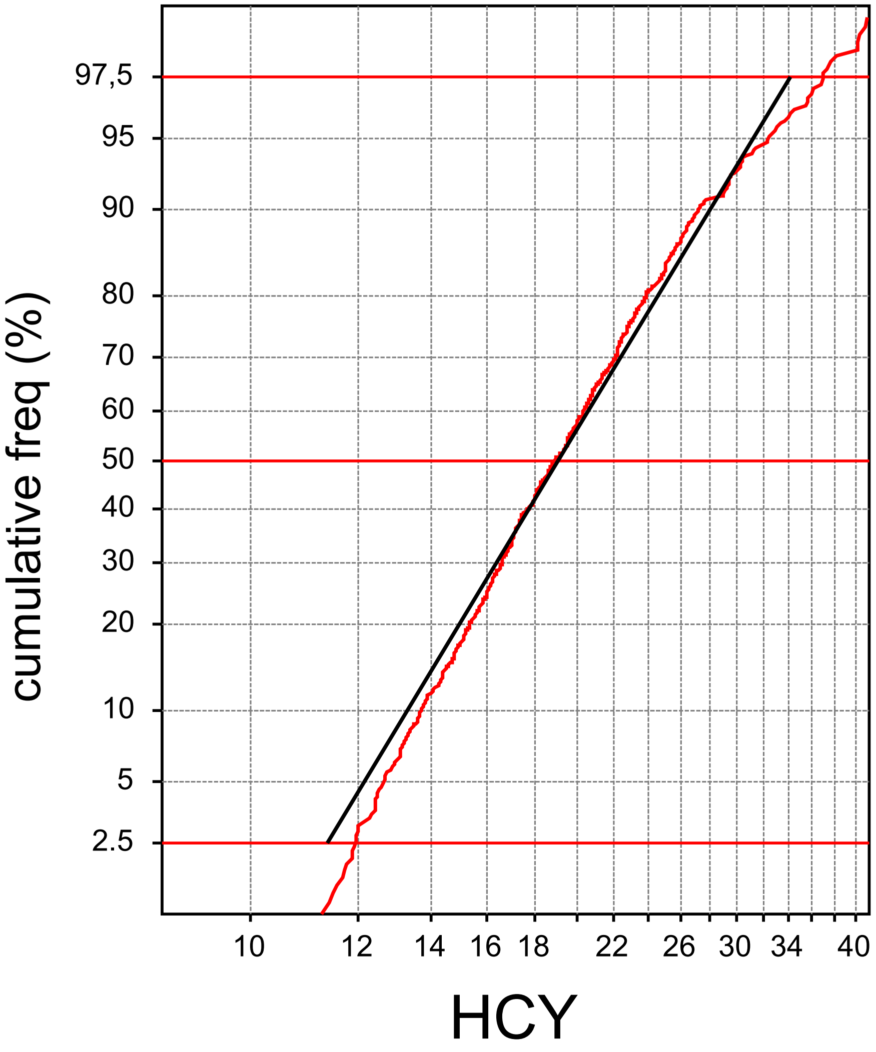

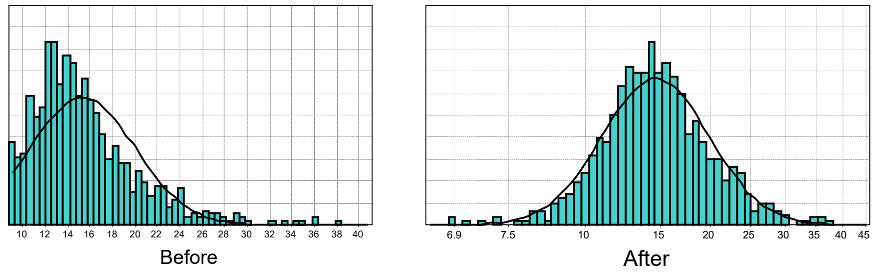

HCY F n=769
Para: 8.70 ~ 14.59 ~ 26.03
Nonpara: 8.49 ~ 14.42 ~ 27.62
Pow=0.406 TPos=6.844
Kurt=-0.237 Skew=0.155
K-S test for normality: .376 (NS)

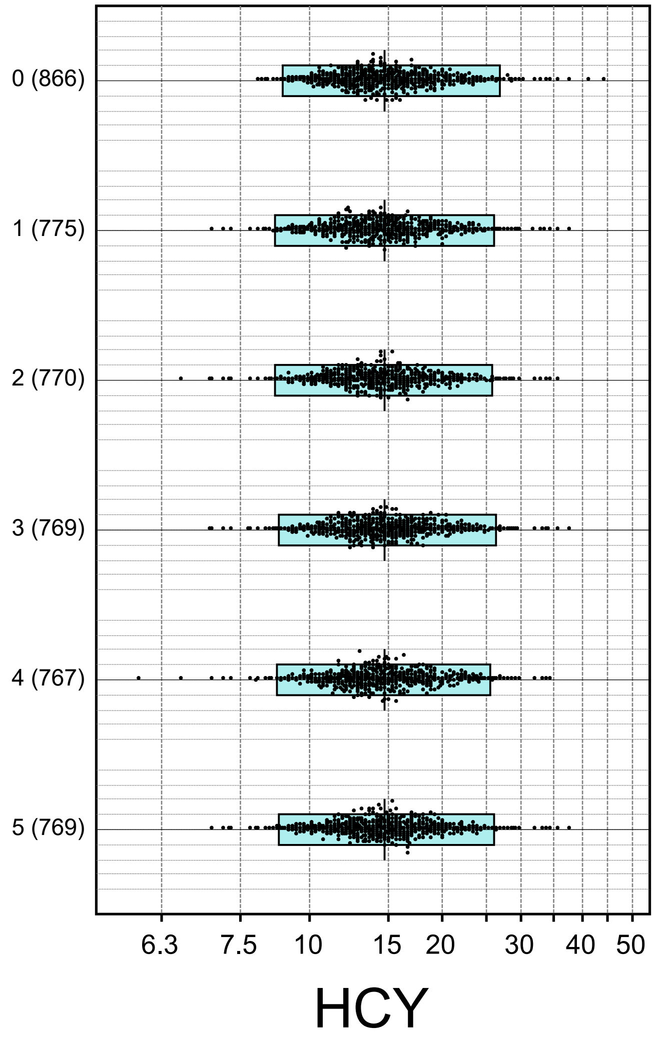

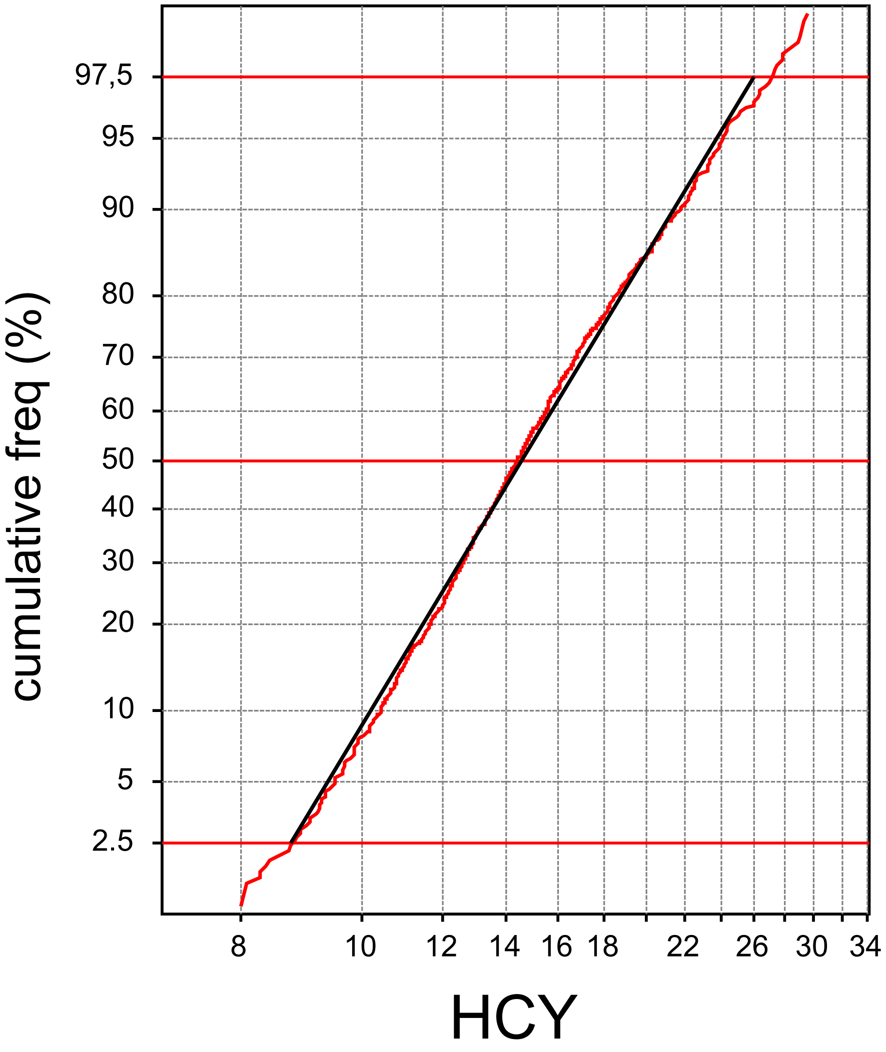

Supplement: Supplementary file 1 — Supplementary Material [file j_med-2025-1285_suppl_001.pdf]
