# Supplementary figures and images for: Establishing reference intervals for 25 common biochemical analytes in Tibetans living at very high altitude
Source: Open Med (Wars). 2026 Jan 19;21(1):20251285. doi: 10.1515/med-2025-1285 (PMC12917553; doi:10.1515/med-2025-1285)

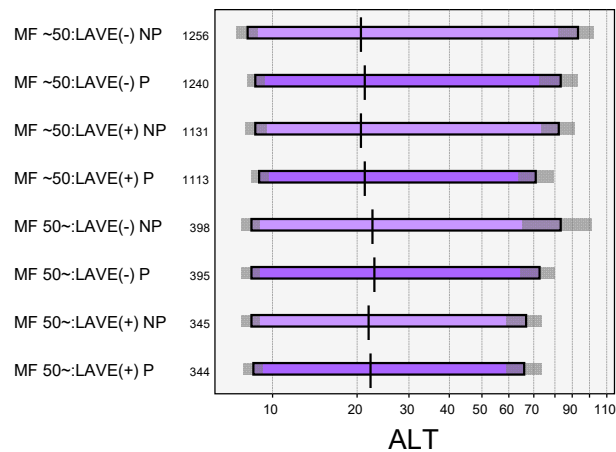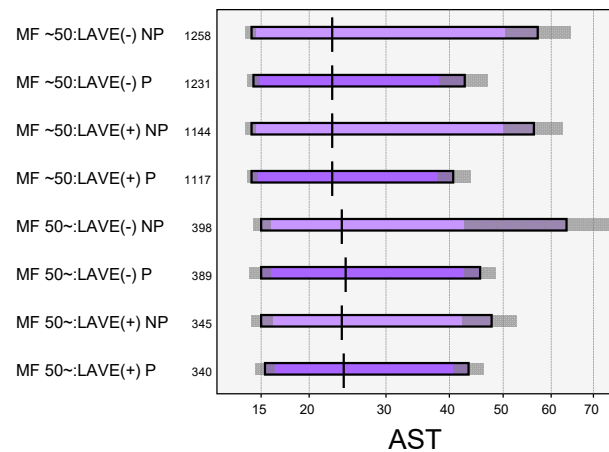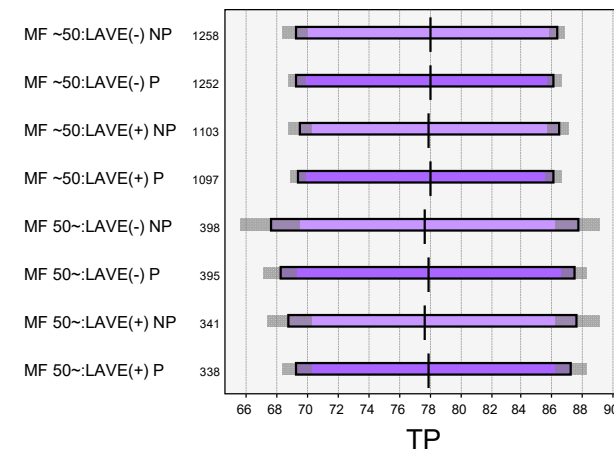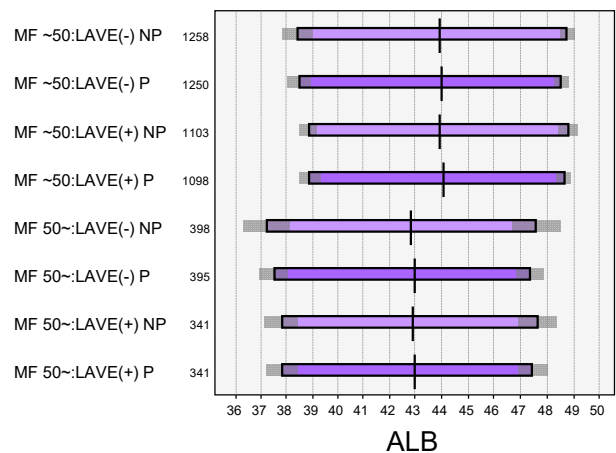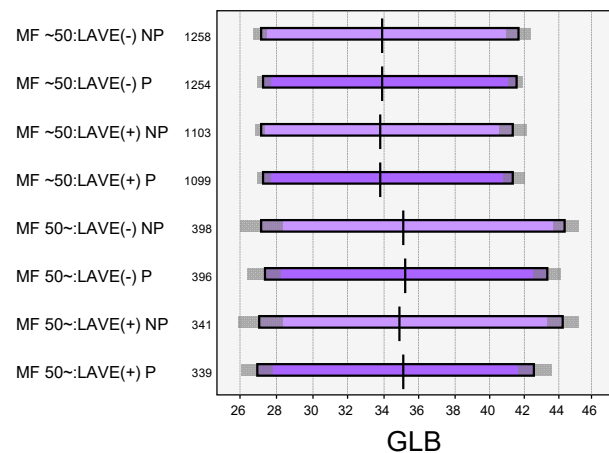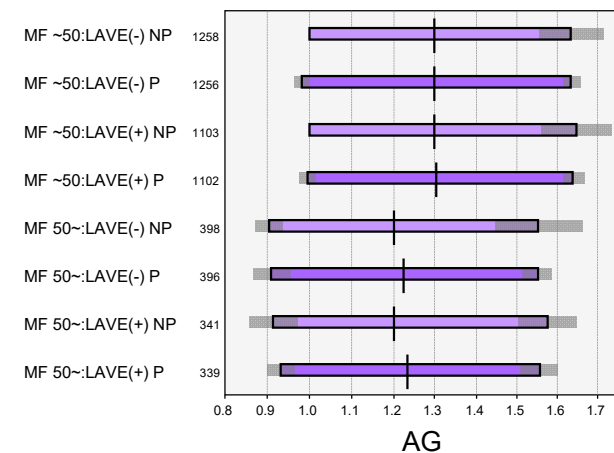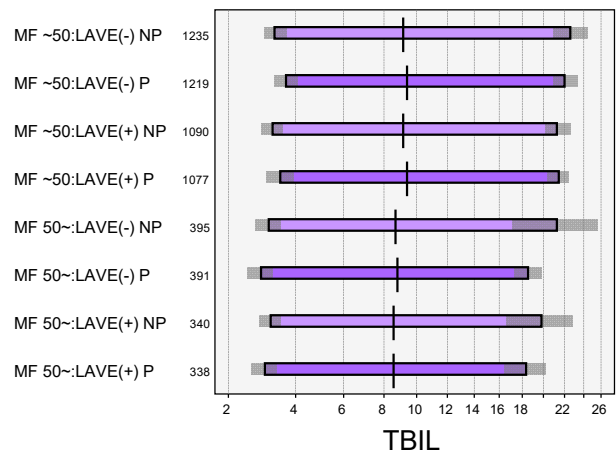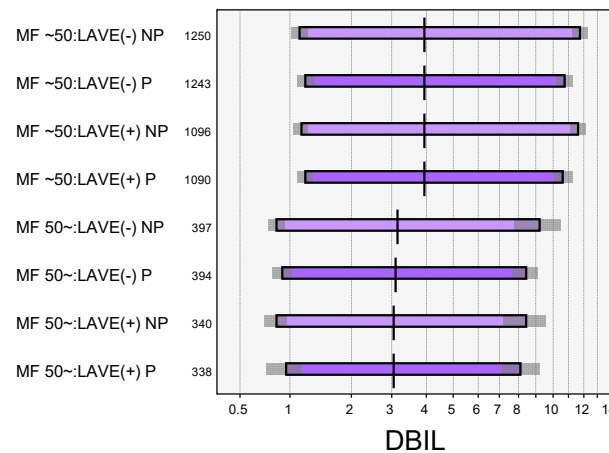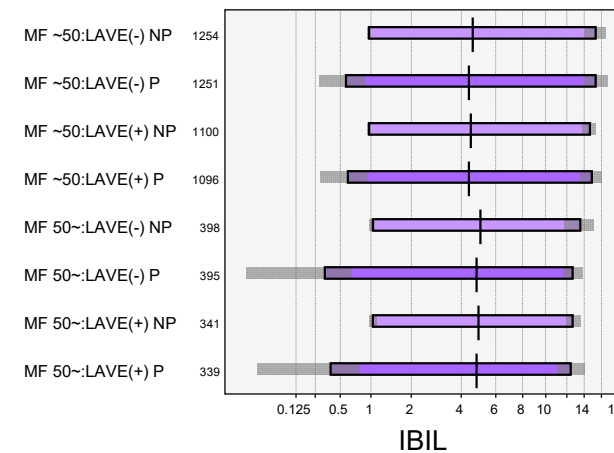

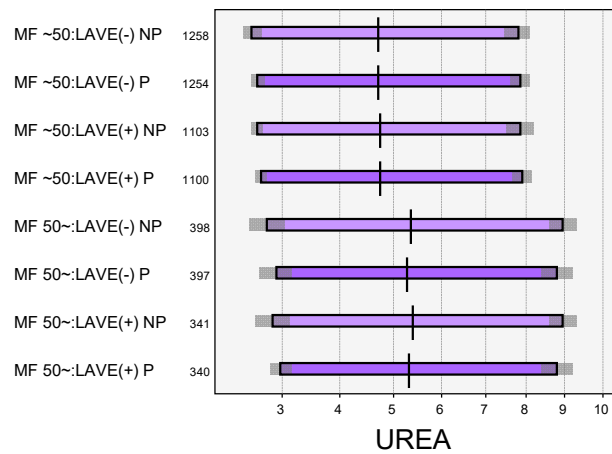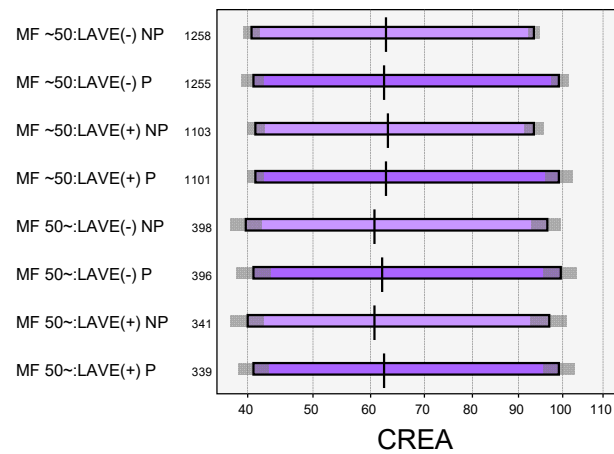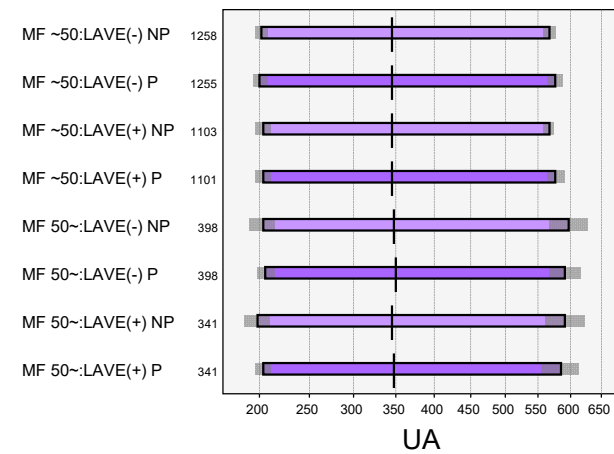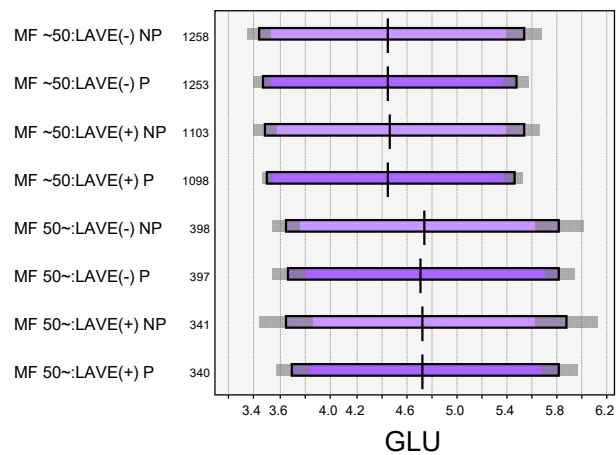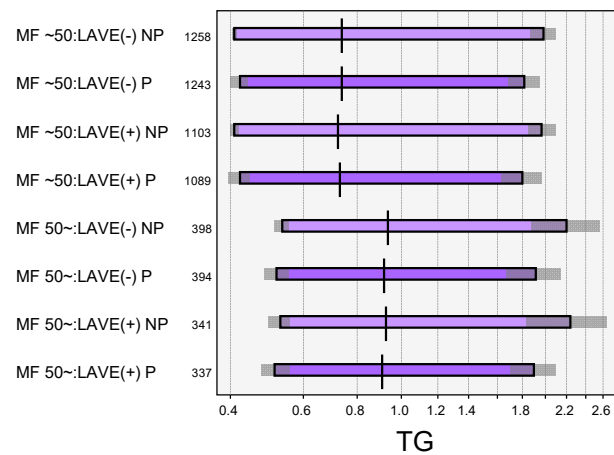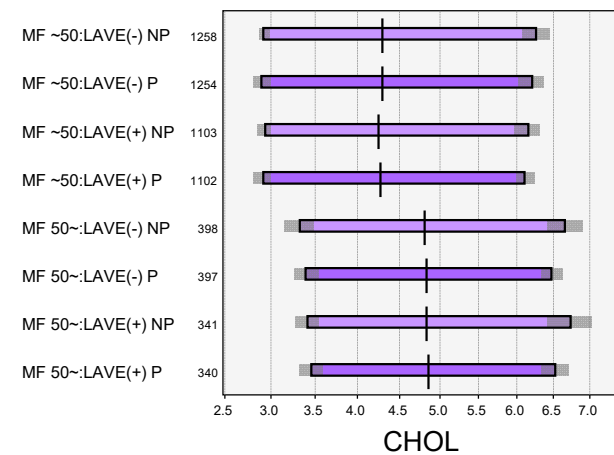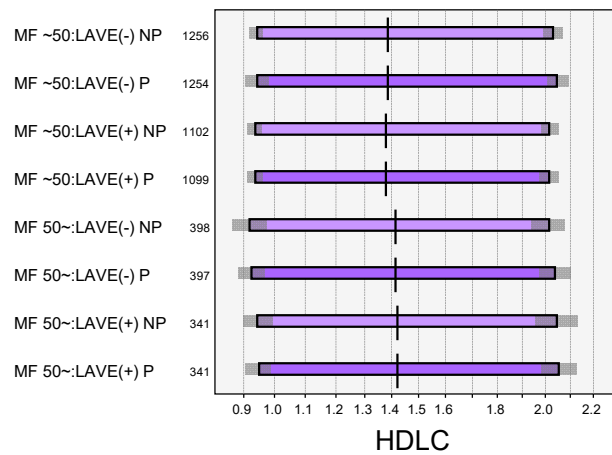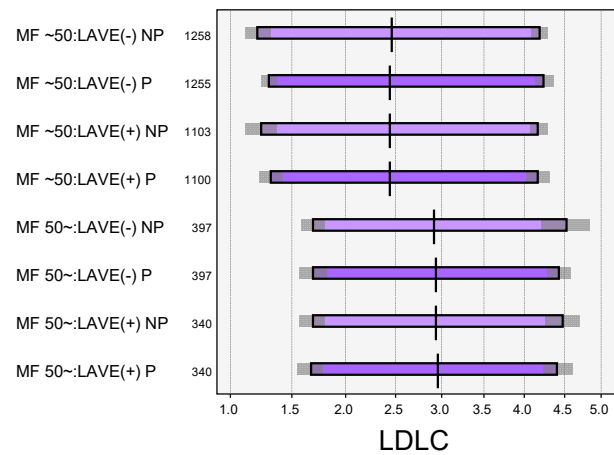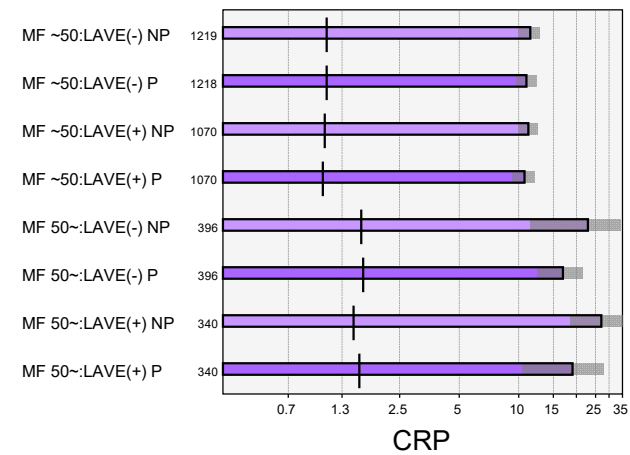

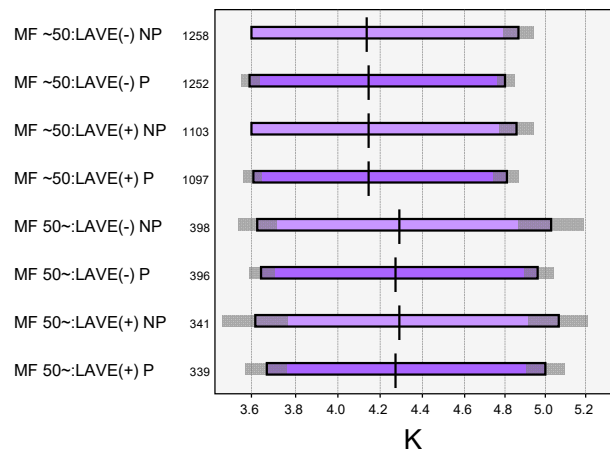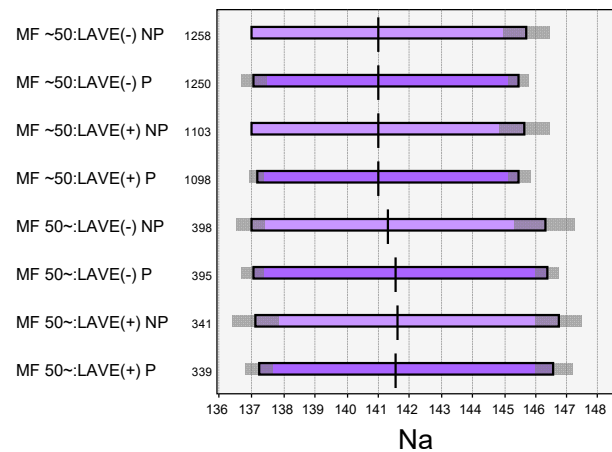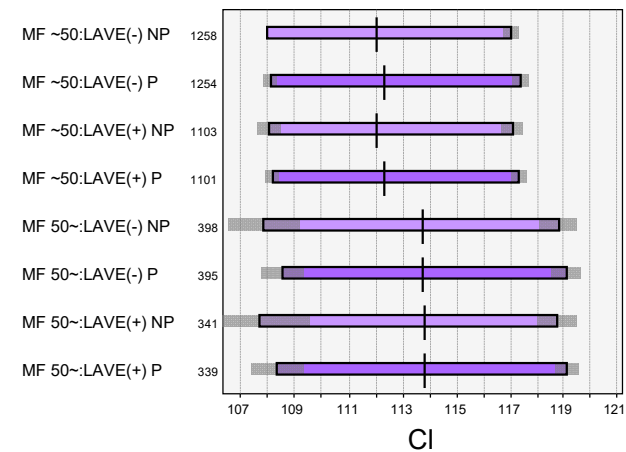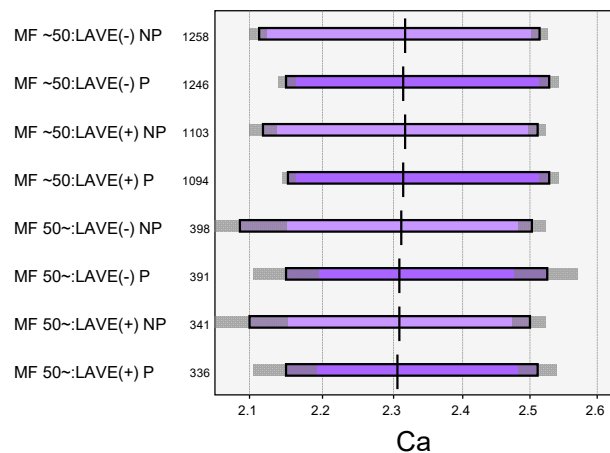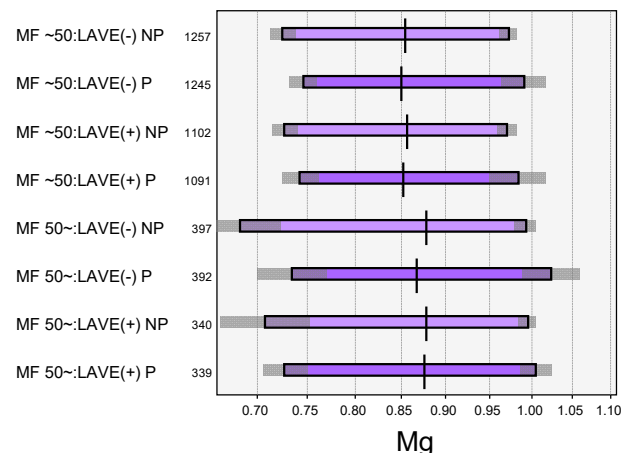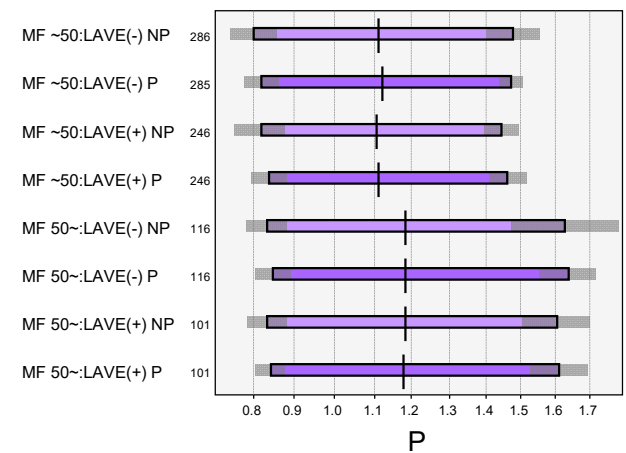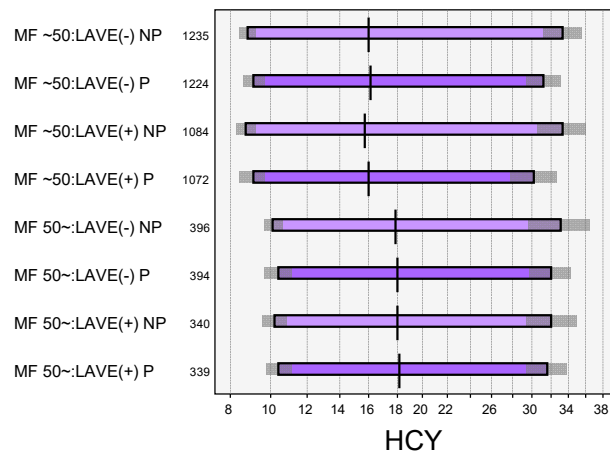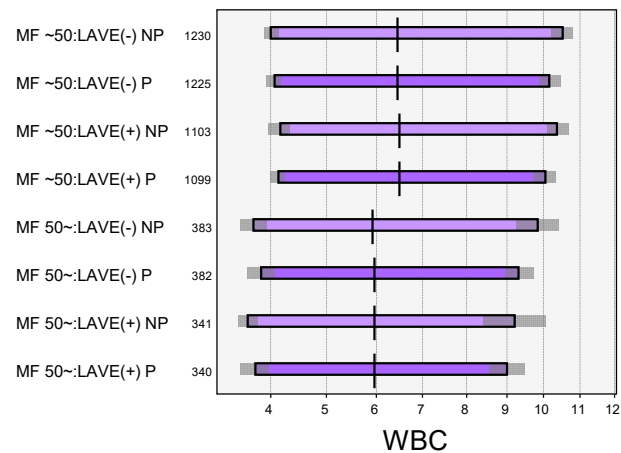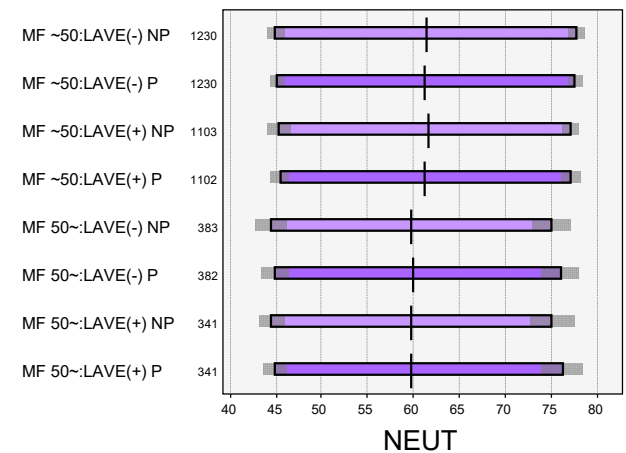

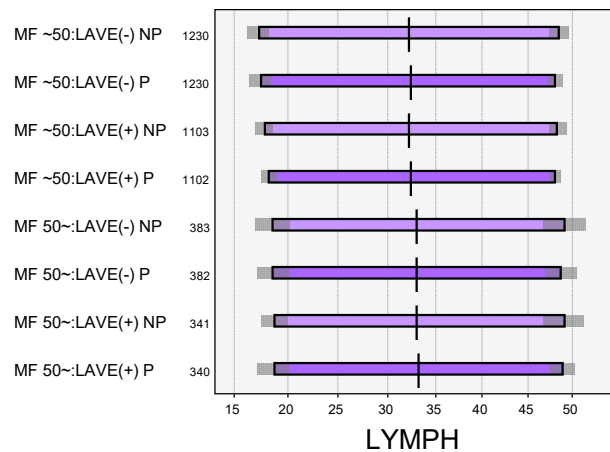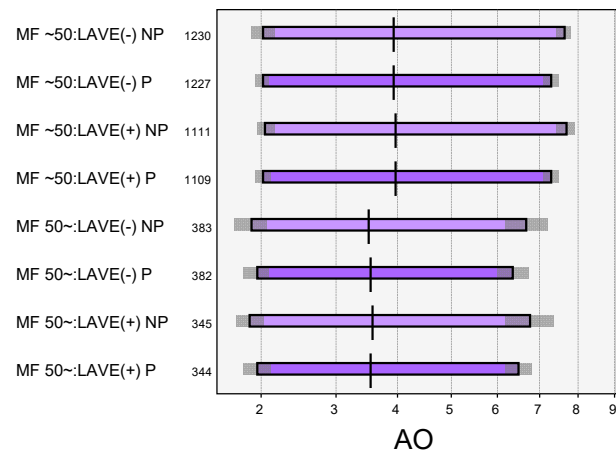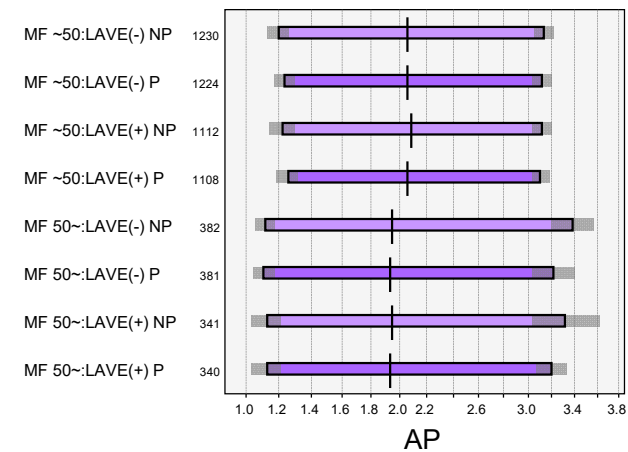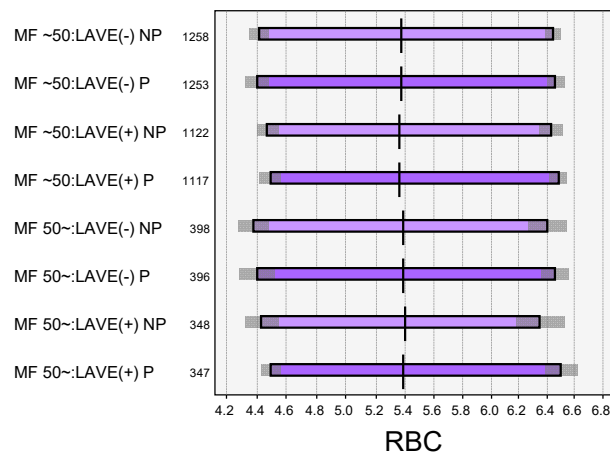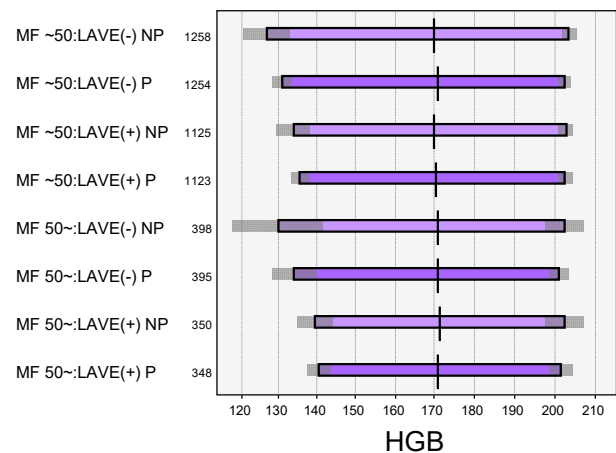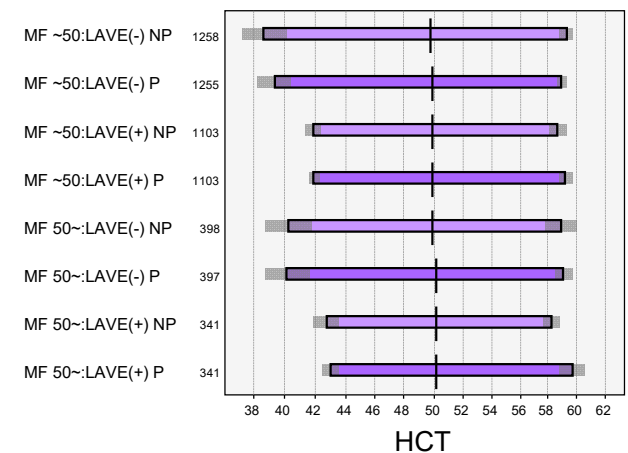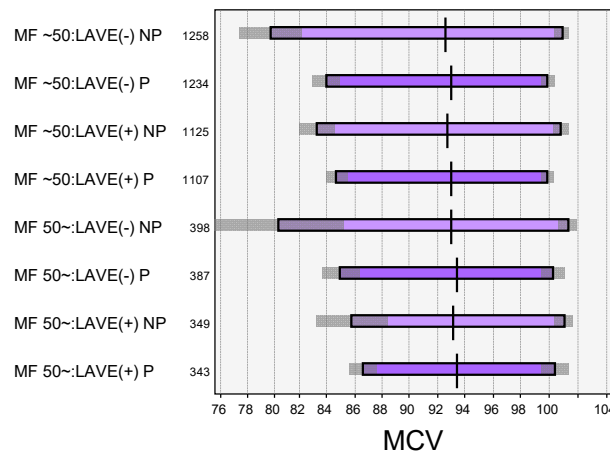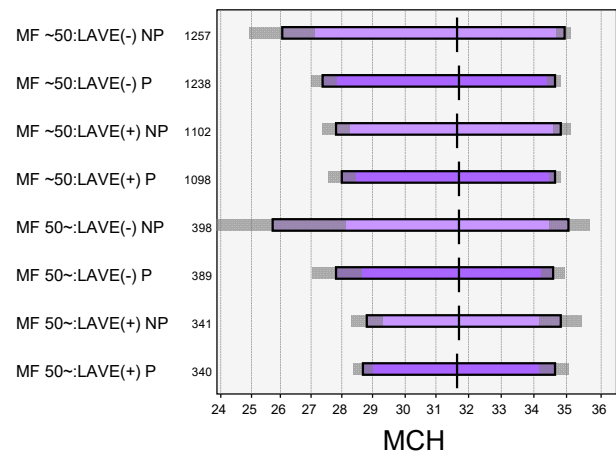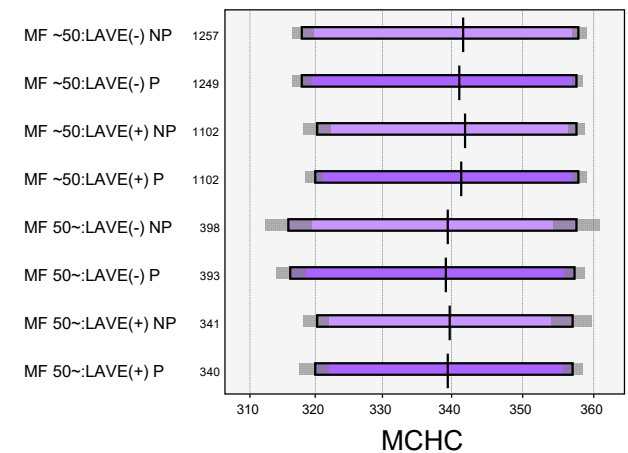

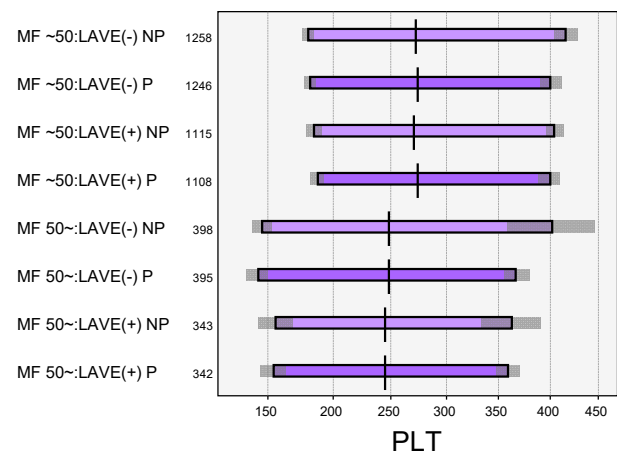

Supplement: Supplementary file 2 — Supplementary Material [file j_med-2025-1285_suppl_002.pdf]
