# Supplementary material for: Establishing reference intervals for 25 common biochemical analytes in Tibetans living at very high altitude
Source: Open Med (Wars). 2026 Jan 19;21(1):20251285. doi: 10.1515/med-2025-1285 (PMC12917553; doi:10.1515/med-2025-1285)
Supplement: Supplementary file 5 — Supplementary Material [file j_med-2025-1285_suppl_005.docx]

**Supplemental Data**

**Supplementary File S1.** Histograms and probability plots before and after Box-Cox transformation

**Supplementary File S2**. Effect of LAVE procedure

**Supplementary Table S1.** Comparisons of RIs for all analytes

**Supplementary Table S2.** Multiple regression analysis [MRA]
